# Development of a cost-effective high-throughput process of microsatellite analysis involving miniaturized multiplexed PCR amplification and automated allele identification

**DOI:** 10.1186/1479-7364-7-6

**Published:** 2013-03-05

**Authors:** Truc TM Nguyen, Shaheen E Lakhan, Barry A Finette

**Affiliations:** 1 Department of Pediatrics, Vermont Cancer Center, University of Vermont, Burlington, VT, 05401, USA; 2 Medicine Institute, Cleveland Clinic, Cleveland, OH, 44195, USA; 3 Neurological Institute, Cleveland Clinic, 9500 Euclid Ave, Cleveland, OH S100A, USA; 4 Biosciences Department, Global Neuroscience Initiative Foundation, Beverly Hills, CA 91412, USA

**Keywords:** Microsatellite instability, Loss of heterozygosity, Multiplexed PCR

## Abstract

**Background:**

Microsatellites are nucleotide sequences of tandem repeats occurring throughout the genome, which have been widely used in genetic linkage analysis, studies of loss of heterozygosity, determination of lineage and clonality, and the measurement of genome instability or the emergence of drug resistance reflective of mismatch repair deficiency. Such analyses may involve the parallel evaluation of many microsatellite loci, which are often limited by sample DNA, are labor intensive, and require large data processing.

**Results:**

To overcome these challenges, we developed a cost-effective high-throughput approach of microsatellite analysis, in which the amplifications of microsatellites are performed in miniaturized, multiplexed polymerase chain reaction (PCR) adaptable to 96 or 384 well plates, and accurate automated allele identification has been optimized with a collective reference dataset of 5,508 alleles using the GeneMapper software.

**Conclusions:**

In this investigation, we have documented our experience with the optimization of multiplex PCR conditions and automated allele identification, and have generated a unique body of data that provide a starting point for a cost-effective, high-throughput process of microsatellite analysis using the studied markers.

## Background

Microsatellites are nucleotide sequences of tandem repeat units ranging from 1 to 6 nucleotides that occur throughout the genome. Their polymorphisms, primarily exhibited as variations in length from the expansion or contraction of repeat units, reflect the tendency of DNA polymerase to slip during replication of repeat tracts. Although polymorphic through evolution, microsatellites are generally stably inherited between closely related individuals [1]. These attributes have led to their wide use in genetic linkage analysis, studies of loss of heterozygosity (LOH) in cancer, and in the determination of lineage and clonality [2].

On the other hand, microsatellite instability (MSI) in tumors, first observed in a proportion of sporadic colon cancers [3,4] and most colon cancers of hereditary nonpolyposis colorectal cancer (HNPCC) families [5], may indicate genetic instability as a result of mismatch repair (MMR) deficiency [6,7]. The MMR system corrects post-replication base-base mismatches and insertion/deletion loops, and has also been implicated in the cytotoxicity of some DNA-damaging agents [8,9]. Mismatch repair deficiency has been observed to exhibit a high mutation rate [10] and confer tolerance to methylating agents [11,12], cisplatin [13,14], and 6-thioguanine or 6-mercaptopurine [14,15]. Since the findings of MSI in colon cancer, the measurement of microsatellite instability has been extended to other solid tumors [16] and hematological malignancies [17-19], as an indication of the potential contribution of mismatch repair deficiency in tumor development, transformation [20-22], and drug resistance.

Depending on the type of analysis, microsatellite markers are selected based on their chromosomal locations and frequency of heterozygosity. In genetic linkage analysis, studies of LOH and the determination of lineage, polymorphic markers are utilized to distinguish and follow alleles on homologous chromosomes, while sensitive and specific measurements of microsatellite instability reflective of mismatch repair deficiency in isolated colon cancer samples are better achieved with quasimonomorphic mononucleotide markers, including Bat25, Bat26, NR22, and NR24 [23,24].

In some cases, these studies may involve the parallel evaluation of many microsatellite loci that are often limited by sample DNA, can be labor-intensive depending on the number of loci and samples being examined, and require the processing of numerous data. To address these limitations, we demonstrate a cost effective, high-throughput, and reproducible process of microsatellite analysis from polymerase chain reaction (PCR) amplification to automated data processing using model studies of microsatellite instability and lineage determination. Performed in miniaturized and multiplexed PCR format, our assay evaluates 11 microsatellite loci consisting of the NCI-recommended panel [25,26] of mononucleotide markers, Bat25 and Bat26, and dinucleotide markers (D2S123, D5S2346, and D17S250), and 6 additional dinucleotide markers (D3S1262, D3S3623, D6S262, D7S481, D9S171, and D18S61), that altogether samples 9 chromosomes. We have designed the assay to also simultaneously evaluate for LOH of mismatch repair components commonly associated with HNPCC by including polymorphic markers, with the exception of Bat26, that are in close proximity to *MLH1* (D3S3623), *MSH2* (Bat26), *MSH6* (D2S123), and *PMS2* (D7S481) gene regions. For other applications, markers may be replaced or added to the existing assay format with some optimization. Following the separation of amplified products by capillary electrophoresis, accurate automated allele identification is performed with optimized peak detection algorithm and sizing method using the GeneMapper software.

## Results

A quality high-throughput process of microsatellite analysis requires specific high-throughput PCR amplification of microsatellite regions and automated specific peak detection, and precise sizing of amplified fragments. In the following, we present optimized conditions for the amplification of 11 microsatellite loci in two miniaturized, multiplexed PCR reactions. We also present the optimization of analysis methods and marker parameters of the GeneMapper software to detect specific peaks of amplified products from background signals and precisely size microsatellite fragments in order to achieve reproducible automated allele identification.

### Performance of multiplex PCR

**Figure 1:**
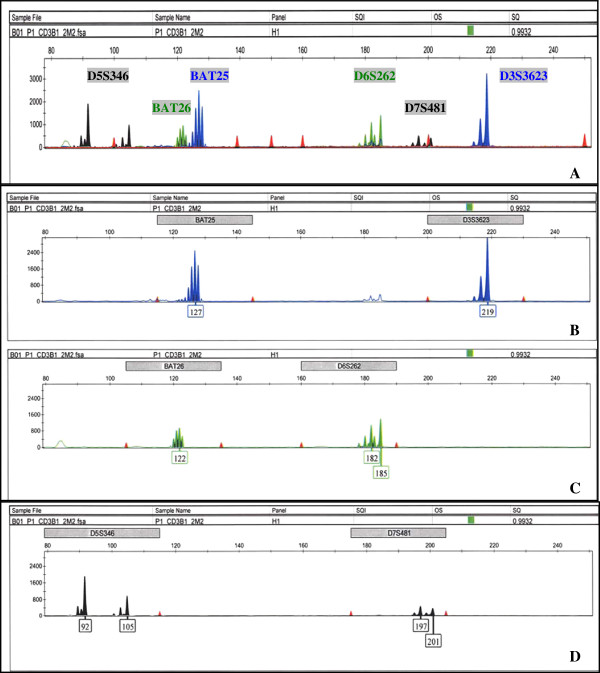
**Electropherograms of group I markers. **The *x*-axis represents DNA fragment size in base pairs, and the *y*-axis represents fluorescence units. (**A**) Complete spectrum of group I markers labeled with specific fluorescent tags. D5S346 and D7S481 labeled with Ned, Bat26 and D6S262 labeled with Hex, and Bat25 and D3S3623 labeled with Fam. Similarly labeled fragments are distally spaced in size by assay design to accommodate population distribution and possible microsatellite instability. Nonspecific signals are kept at a minimum; however, there is overlap in spectral emission of Hex into Fam, observed as similar stutter peaks with lower signals at Bat26 and D6S262 that are labeled as Fam. (**B**) Allele identification of Fam-labeled fragments, Bat25 and D3S3623. Background peaks from the spectral overlap of Hex into Fam were excluded from identification due to either low nonspecific signals compared to specific signals as in Bat25 or by filtering out with specified marker and analysis method parameters as in D3S3623. (**C**) Allele identification of Hex-labeled fragments, Bat26 and D6S262. (**D**) Allele identification of Ned-labeled fragments, D5S346 and D7S481.

**Figure 2:**
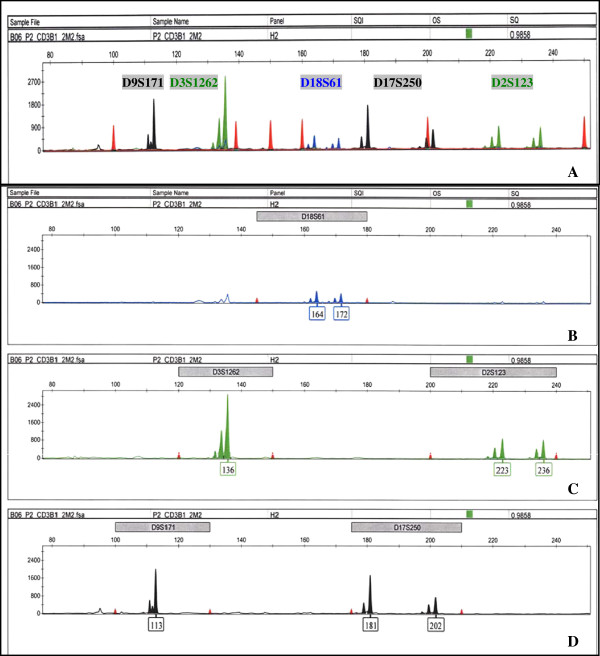
**Electropherograms of group II markers. **The *x*-axis represents DNA fragment size in base pairs, and the *y*-axis represents fluorescence units. (**A**) Complete spectrum of group II markers labeled with specific fluorescent tags. D9S171 and D17S250 labeled with Ned, D3S1262 and D2S123 labeled with Hex, and D18S61 labeled with Fam. Similar to group I, the spectral overlap of Hex into Fam is observed at D3S1262 and D2S123. (**B**) Allele identification of Fam-labeled fragment, D18S61. Nonspecific stutters from the spectral overlap of Hex into Fam were excluded from identification by filtering out with specified marker and analysis method parameters. (**C**) Allele identification of Hex-labeled fragments, D3S1262 and D2S123. (**D**) Allele identification of Ned labeled fragments, D9S171 and D17S250. Nonspecific peaks outside of marker size range were excluded from identification by filtering out with specified marker and analysis method parameters.

*In silico* design and optimization to minimize hairpin and primer-dimer formation as well as cross-dimerization among possible primer combinations were performed prior to *in vitro* testing. Predicted compatible primer sets were tested and evaluated for optimal assay performance of low background signals, high fluorescence of specific PCR products that fall within the detectable dynamic range, and signal and size resolution among amplified products. We found that multiplexing Bat25, Bat26, D3S3623, D5S346, D6S262, and D7S481 into one PCR reaction (group I) and D2S123, D3S1262, D9S171, D17S250, and D18S61 into another (group II) met these criteria. The compatibility of primer sets in our experimental conditions is overall consistent with *in silico* predictions. Figure 1 shows the electropherograms of group I markers amplified with human T-cell lysates in a 10 μL PCR reaction. Figure 1A displays the complete spectrum of an individual’s microsatellite profile for group I markers with internal size standards represented as red peaks. Bat25 and D3S3623 are labeled with Fam (blue), Bat26 and D6S262 with Hex (green), and D5S346 and D7S481 with Ned (black). Similarly labeled fragments are distally spaced in size by assay design to accommodate both population size distribution and possible microsatellite instability. Background signals from nonspecific priming in multiplex PCR are kept to a minimum for each fluorescent tag. However, there is an overlap in the spectral emission of Hex into Fam, which is most evident at D6S262. These parameters are examined in detailed for each dye in Figure 1B (Fam), 1C (Hex), and 1D (Ned). Red peaks in Figure 1B–D mark the size range for each microsatellite marker. In all three panels, signals from nonspecific products for each dye are quite low compared to specific signals of amplified microsatellites. Bleed through of Hex into Fam is evident in Figure 1B as background microsatellite fragments labeled with Fam that have similar stutter patterns and size of Bat26 and D6S262. However, this is not concerning for the genotyping of either Bat25 or D3S3623 since the nonspecific signal from Bat26 is extremely low compared to the specific signal of Bat25, and the expected fragment size range of D3S36S3 is approximately 40 nucleotides away from the background peaks from D6S262, which can be excluded from automated identification by the adjustment of marker and analysis method parameters in the GeneMapper software. Alternatively, using narrower band filters for detecting Fam or exchanging labels to tag markers that are farther apart in size can minimize this spectral overlap. Figure 2 shows the electropherograms of group II markers also amplified with cell lysates in a 10 μL PCR reaction. The complete spectrum of group II markers is shown in Figure 2A, consisting of D18S61 labeled with Fam, D2S123, and D3S1262 labeled with Hex, and D9S171 and D17S250 labeled with Ned; the amplified products of individual dye are analyzed in Figure 2B (Fam), C (Hex), and D (Ned). Similar to group I, fragments labeled with the same fluorescent tags are distally spaced apart, and background signals from nonspecific priming in multiplex PCR are minimized within the relevant marker size ranges. The spectral overlap of Hex into Fam is again observed in Figure 2B as microsatellite fragments with the same stutter patterns and size as D3S1262 and D2S123. Since these background peaks are present outside the size range of D18S61, they will not interfere with the genotyping of D18S61. The nonspecific peak outside of the lower limit of D9S171 size range in Figure 2D can also be excluded from identification with specified marker and analysis parameters. Overall, PCR conditions for groups I and II markers produce data with clear resolution of specific signals and sizes of similarly labeled amplified fragments, which are suitable for high-throughput microsatellite analysis.

### Specific peak detection and precise sizing

Automated data processing was performed with the GeneMapper software. A reference dataset of 5508 alleles from 27 subjects and 1 cancer cell line (Additional file 1) was used to optimize parameters for specific peak detection, precise fragment sizing, and accurate allele identification of amplified microsatellites, which minimize the recognition of nonspecific artifacts of PCR amplifications and dye bleed through. Several peak detection algorithms and sizing methods were tested. Superior genotyping quality was observed using the advanced peak detection algorithm in combination with the Local Southern sizing method (parameters listed in Table 1), which determines the best-fit line fragment size for an unknown fragment from the four nearest sizing standards based on the reciprocal relationship between fragment length and mobility ([27] Applied Biosystems, Part Number 4366831 Rev. A 2005). We also tested different sizing methods, including the third-order least squares and Global Southern methods, and did not observe comparable precision as the Local Southern method. This is consistent with what Ghosh and colleagues had observed with fragment sizing [28]. We further improved specific peak detection by increasing the minimum peak half width to 4 points for group I markers to minimize the recognition of nonspecific spikes observed in some samples.

**Table 1:** Analysis method parameters

**Analysis method**	**Multiplex PCR group 1**	**Multiplex PCR group 2**
Peak detector tab		
Peak detection algorithm	Advanced	Advanced
Size calling method	Local Southern	Local Southern
Minimum peak half width	4 points and	2 points
Allele tab		
Mono cut-off value	0.95	N/A
Range filter		
Blue	146-194	181-250
Green	136-159	151-199
Yellow	116-174	131-174

Optimized parameters for automated specific peak detection, precise sizing, and accurate allele identification.

Along with peak detection and fragment sizing, parameters were also optimized for accurate allele calling of amplified microsatellites with unique nucleotide repeats. Specific allele identification was promoted by specifying the size range for each marker (Table 2), using previously determined values of a population sampling from the GDB human genome database that have been adjusted for primer locations and product size in our assay conditions (Table 3), and also those determined from 27 additional subjects and 1 cell line in our studies (Additional file 1). Nonspecific peaks outside the defined size range for each marker that may interfere with allele identification of similarly labeled marker, including bleed through of Hex into Fam, were additionally filtered out by specified range filters in the analysis method parameters (Table 1). Allele identification of quasimonomorphic microsatellites with mononucleotide repeats, including Bat25 and Bat26, was optimized to recognize the highest peak among stutter peaks as allele 1, and peaks that have heights >95% of the maximum peak height or height of allele 1 by setting the mono cut-off value at 0.95 as additional alleles (Table 1). This setting reproducibly identified at most two alleles for Bat25 and Bat26 per PCR amplification of a sample without microsatellite instability in our dataset.

**Table 2:** Marker parameters

**Multiplex PCR reaction**	**Markers**	**Minimal size**	**Maximum size**	**Marker repeat**
1	BAT25	115	145	1
1	BAT26	105	135	1
1	D3S3623	200	230	2
1	D5S346	70	115	2
1	D6S262	160	190	2
1	D7S481	175	205	2
2	D2S123	200	240	2
2	D3S1262	120	150	2
2	D9S171	100	130	2
2	D17S250	175	210	2
2	D18S61	145	180	2

Defined size ranges of markers, which include the GDB human genome database population distribution and additional allele sizes observed in our studies for optimal automatic allele identification.

**Table 3:** Microsatellite markers size ranges and primer design and concentrations in multiplex PCR reactions

**PCR reaction**	**Marker**	**Range of PCR product size (bases)**	**Dye**	**Primer sequence (5**^′^**→3**^′^)	**Primer (μM)**
1	BAT25	130	Fam	FamTCGCCTCCAAGAATGTAAGTgtgtcttTCTGCATTTTAACTATGGCTC	0.25
1	BAT26	120	Hex	HexTGACTACTTTTGACTTCAGCCgtgtcttAACCATTCAACATTTTTAACCC	2.00
1	D3S3623	207–223	Fam	FamCCATGTTGGTTAAAGGCAAGgtgtcttCTCTGAACTGAAGTGACCTCC	0.25
1	D6S262	167–183	Hex	HexATTCTTACTGCTGGAAAACCATgtgtcttGGAGCATAGTTACCCTTAAAATC	0.50
1	D7S481	181–199	Ned	NedTTCTCATTCTCACCCCCATgtgtcttATCCCCCACTGTCTCCAA	0.50
2	D2S123	203–233	Hex	gtgtcttAACAGGATGCCTGCCTTTAHexGGACTTTCCACCTATGGGAC	1.00
2	D3S1262	132–146	Hex	HexCAGTTGTGAGCCACCATGTCgtgtcttCAGTTTTTATGGACGGGGT	2.00
2	D9S171	102–122	Ned	NedGTGAACCTCATCTCTGTCTGCTgtgtcttACTTTATTAACAATCAGTATTTTCC	0.25
2	D17S250	188–203	Ned	gtgtcttGTAAGCATAAAAAGGAAGAATCANedTTACAGGCATGAGCCACTC	0.50
2	D18S61	150–176	Fam	FamATTTCTAAGAGGACTCCCAAACgtgtcttGAAACTCAGGAGCATGGTTATG	0.50

Summary of microsatellite markers, population size ranges for amplified products with specific primer sets, and primer sequences, labels, and concentrations used in multiplex PCR amplifications.

The reference dataset was also utilized to define bins for each marker, which are rounded off to specified integer values that represent different categories of fragment sizes of amplified products for a particular microsatellite, to enable automatic allele identification of detected peaks that fall within the bin settings. The maximum offset of each bin was set at +/-0.5 base pair to allow up to one nucleotide range of experimental variations for each fragment size. Day-to-day inter-experimental and across time variations related to reagent preparations, PCR amplifications, and electrophoretic mobility differences between experiments resulting in large bin ranges ≥ 1 bp have been observed by others [28]. Some of these variations can be adjusted by applying a correction factor based on differences in performance of control samples included on every run [28], which have been shown to reduce bin ranges (to ≤ 0.8 bp) and increase interbin distances that would improve identification of alleles differing in size by 1 bp. We did not perform this correction prior to binning our initial data set to examine the total variation of our experiments, and our initial dataset also did not require 1 bp resolution.

### Quality of high-throughput microsatellite analysis

We next assessed the performance of the entire high-throughput process from PCR amplification to automated data analyses for potential sources of variations and process accuracy and reproducibility over the course of 5 months. Subsets of the reference dataset including studies of microsatellite instability among peripheral T cell clones from different subjects and lineage determination of an unknown family, which have been automatically analyzed with optimized parameters and bin settings, were utilized in these assessments since the observed outcome from those studies provided the appropriate controls for comparison.

In those studies, PCR amplifications were performed in 10 μL reactions with either purified DNA or cell lysates prepared from peripheral T cell clones isolated from human subjects, which expressed varying growth rates and were cultured at different times. The inclusion of different conditions captured the potential variation associated with sample preparation, including the contribution of background matrix from tissue culture and DNA isolation. Along with every analysis of unknown samples, we included the amplification of a known control sample to distinguish process reproducibility from sample integrity as the source of variation. Not included in our comparisons were PCR amplifications of samples that resulted in no signals or very low signals that do not display distinguishable marker peaks when our controls were performing. These may be the result of interferences from sample matrix and/or low DNA content since the use of higher sample volumes resulted in the successful amplifications of some samples.

The reproducibility of each day’s processing as well as over the course of 5 months was assessed by examining the performance of the control samples. Additional files 2 and 3 provide the sizing values for identified alleles of group I and II markers, which have been amplified with cell lysates in 23 and 18 different runs, respectively, and analyzed with optimized parameters using the GeneMapper software. The sizing values of alleles for control samples, which reflect processing from PCR amplifications of microsatellite regions, fragment separation by capillary electrophoresis, and specific peak detection to sizing of fragments were very reproducible over the course of 5 months showing cumulative %CV ranging from 0.05% to 0.12% among identified alleles. In addition, automatic allele identification with indicated bins was accurate in 360 out 364 alleles analyzed for markers with dinucleotide repeat units. Although peak detection and sizing may be accurate, problems with automatic allele calling of markers with dinucleotide repeats occur in cases where signals from nonspecific products generated during PCR amplifications have surpassed pre-set threshold values. This was observed on two occasions for D5S346 as highlighted in blue (Additional file 2).

We observed the most ambiguity with allele calling of mononucleotide markers, Bat25 and Bat26 (also shown in Additional file 4). The algorithm for automatic allele identification of markers with mononucleotide repeats was optimized to reproducibly call peaks as alleles by recognizing the tallest peak as allele 1 and peaks that meet the preset cut-off value of 95% of the maximum peak height or allele 1 among stutter peaks as additional alleles. By this algorithm, Bat25 alleles were identified as homozygous for 127 bp in 18 of 23 runs of a pooled control sample and as heterozygous for 127 and 128 nucleotides in 5 runs. Bat26 was identified as 122 bp in all 23 runs. The variations in allele calling of mononucleotide markers reflect the variability in biochemistry of amplifying regions of mononucleotide repeats due to DNA polymerase slippage, and relative consistency appears to depend on pre-set criteria of tolerance for variations. When these markers were analyzed manually, the variability is similar or may be subject to more inter-observer variation in the estimation of differences in peak heights when compared to automated allele identification. As these variations likely reflect the tendency of slippage of DNA polymerase during the amplification of mononucleotide repeats rather than process variability, we accepted a deviation of +1 nucleotide as normal for these markers. This tolerance does not negate the usefulness of these markers and/or our methodology. Other groups considered variations of ≥3 bp for Bat25 and ≥4 bp for Bat26 as true polymorphisms or somatic alterations of these markers [23].

Process accuracy and reproducibility were further examined in an analysis of 4,440 identified alleles of groups I and II markers from the amplifications of 240 and 156 T cell clones from 13 and 10 subjects, respectively, in a microsatellite instability study. This dataset is unique in that different clones with unique T cell receptor for antigen recognition from the same subjects exhibited identical patterns of microsatellite profiles or microsatellite stability, providing the appropriate internal controls for measuring process deviations. Additional files 4 and 5 provide summaries of identified alleles for groups I and II markers, respectively, organized in chronological order by dates of processing, subjects and respective number of clones analyzed, and the percent deviation from expected values. Percent deviation from expected values connotes the percent of alleles that were miscalled, indicated by asterisk. All discordant samples were manually analyzed to confirm that deviations were unrelated to microsatellite instability. Misidentifications can represent failure in any part of the process from sample preparation, PCR amplification, fragment separation, peak detection, fragment sizing to automated allele calling. The analysis of 1,920 alleles of dinucleotide markers in group I (Additional file 4) showed percent deviations from expected values ranging from 0.0% to 4.69% for day to day processing and of 0.63% for all days. When discordant samples were analyzed manually, most deviations from expected allele calls were related to the presence of higher nonspecific signals or unequal amplifications of alleles. On the day showing the highest percent deviation, three independent amplifications of control samples were performed optimally, indicating that the deviations were likely related to sample integrity. There was no trend of higher deviations among any of the markers. As previously mentioned, mononucleotide markers Bat25 and Bat26 are more difficult to call whether performed manually or automatically even though their fragment sizing is very precise (Additional file 2). We accepted a deviation of +1 nucleotide as normal and did not calculate a percent deviation for these markers since all alleles were automatically called within this range.

The analysis of 1,560 alleles for group II dinucleotide markers (Additional file 5) showed percent deviations from expected values ranging from 0.0% to 3.57% for day to day processing and of 0.77% for all days. Most of discordant samples showed deviations related to the presence of higher nonspecific signals and unequal amplifications of alleles as observed with group I markers. On the day showing the highest percent deviation, our control sample performed optimally, suggesting that sample integrity is the cause of poor PCR amplifications. There was also no apparent trend of higher deviations among any of the markers in group II.

**Figure 3:**
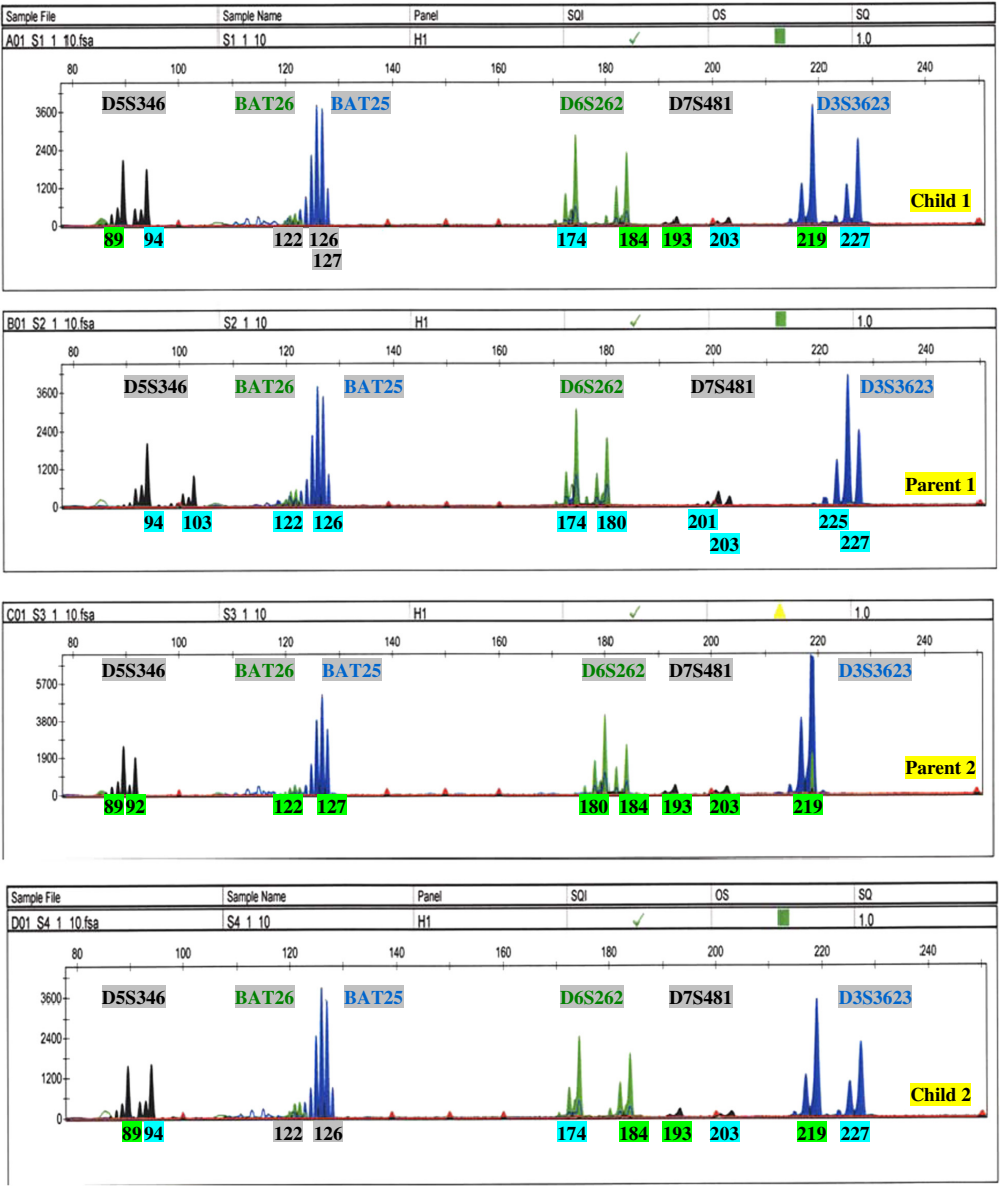
**Microsatellite profiles of group I markers of family members in lineage determination. **The *x*-axis represents the DNA fragment size in base pairs, and the *y*-axis represents the fluorescence units. Relationships among family members were predicted based on the patterns of inheritance of microsatellite alleles. Child 1 and 2 are monozygotic twins. (**A**) Microsatellite profile of child 1. Automated identification accurately called inherited microsatellite alleles of dinucleotide markers, which can be traced back to the microsatellite profiles of parent 1 and parent 2. (**B**) Microsatellite profile of parent 1. Alleles have been automatically called with optimized methods. (**C**) Microsatellite profile of parent 2. Alleles have been automatically called with optimized methods. (**D**) Microsatellite profile of child 2. Automated identification accurately called inherited microsatellite alleles of dinucleotide markers, which can be traced back to the microsatellite profiles of parent 1 and parent 2.

Thus far, we have demonstrated the accuracy and reproducibility of our high throughput process with control samples and clones from the same subjects in which deviations from expected patterns of microsatellites were analyzed. The accuracy of our methods was also examined in a lineage determination study of a family of four members unknown to us. Figure 3 shows the microsatellite profiles of group I markers in four panels that we have determined as child 1, parent 1, parent 2, and child 2 based on the patterns of segregation of microsatellite alleles. Alleles inherited from each parent have been appropriately color coded in each child’s microsatellite profiles. As previously discussed with the exception of the ambiguity in identification of Bat25 and Bat26, all alleles of dinucleotide markers were accurately automatically identified in Child 1 and Child 2 and can be traced back to Parent 1 and Parent 2. Furthermore, the microsatellite profiles of Child 1 and Child 2 were identical, suggesting that the children are monozygotic twins. The parents later confirmed these determinations.

## Discussion

We have developed a reproducible high-throughput process of microsatellite analysis in which the amplifications of microsatellite regions are performed in miniaturized, multiplexed PCR format; automated allele identification has been optimized to overcome many challenges encountered in genetic studies of linkage analysis, LOH, lineage determination, or microsatellite instability. Our microsatellite assay minimizes sample requirement using approximately 2–4 ng of DNA in PCR amplifications of 11 microsatellite loci in two 10 μL reactions that can be adapted to a 96- or 384-well high-throughput assay format. Our optimized conditions produced clear resolution of specific signals and sizes of amplified microsatellites, allowing for reproducible peak detection and fragment sizing. The current assay is amenable to the incorporation of more markers to increase throughput without compromising size resolution of additional amplified products.

Using the Advanced Peak Detection Algorithm with specific optimized parameters in combination with the Local Southern sizing method, our control samples were precisely sized with %CV ranging from 0.05% to 0.12% for all markers in groups I and II in 23 and 18 independent runs, respectively, over the course of 5 months. These values included the variation from sample preparation, PCR amplification, fragment separation, peak detection to fragment sizing. For dinucleotide markers, these data also indicate that the addition of the GTGTCTT tail to our primer sets in conjunction with optimized extension time have successfully promoted consistent amplifications of fragment size using a basic PCR protocol. The observed precision demonstrates the reproducibility of this process over time.

From a collective reference dataset of 5,508 alleles from 27 subjects and 1 cell line, we have defined bins or categories representing different fragment sizes for each marker to enable automatic allele identification in future studies with these markers. This dataset is unique in that sufficient replicates of the same allele have been amplified using samples from different T cell isolates that have been cultured and lysed at different times, thus testing a wide spectrum of variation during sample preparation. Allowing a bin offset of +/-0.5 base pairs, the performance of the entire process showed percents deviation from expected values of 0.63% and 0.77% in 1,920 and 1,560 alleles analyzed for groups I and II dinucleotide markers, respectively. Most misidentifications were of nonspecific signals above our preset threshold values or from the unequal amplifications of alleles, which were related to poor sample quality rather than process quality since control samples were performed optimally on the same runs. We actually expected higher deviations since all samples from this dataset were amplified with crude lysates of T cells. In the analysis of control samples, similarly amplified with crude lysates, we had leaned towards a higher estimation of the percent deviation by counting both alleles as being miscalled for marker D5S346 when background signals were also identified as alleles. Automated allele identification of quasimonomorphic mononucleotide markers, Bat25 and Bat26, was highly reproducible using the algorithm of >95% of maximum height as the cut-off value for identification of additional alleles and an allowable +1 nucleotide deviation due to the variable biochemistry of amplifying these markers. These data support the reproducibility and accuracy of this high-throughput process.

We hope that others will benefit from reading about our experiences and further improve their process. In our dataset, we have shown two extreme examples: (1) the analyses of microsatellite stability/reproducibility in the evaluation of a pooled control sample and clones derived from individual subjects, and (2) the contrary analyses of microsatellite instability or divergence of microsatellites through evolution (accelerated in tumors with mismatch repair deficiency) when we examined lineage relationship in the family with monozygotic twins. We expect that these extreme analyses would reflect the feasibility of our process in the analyses of tumor cells that exhibit functional deficiency of mismatch repair and other types of samples, especially since we have also included the five NCI-proposed markers for the evaluation of MSI.

## Conclusions

In this investigation, we have documented our experience with the optimization of multiplex PCR conditions and automated allele identification, and have generated a unique body of data that provide a starting point for a cost-effective, high-throughput process of microsatellite analysis using the studied markers. For specific applications, markers may be exchanged or added with some optimization to the existing assay format and analysis methods. For microsatellite instability studies to test the functional deficiency of mismatch repair, the placement of polymorphic markers in proximity to mismatch repair components in this assay may provide additional information on LOH.

## Methods

### Study population and sample collection

T-cells were isolated from healthy controls and a subset of study participants with inflammatory bowel disease (*n* = 12 with Crohn’s disease and *n* = 7 with ulcerative colitis) and B cell acute lymphoblastic leukemia (*n* = 4) previously recruited from the pediatric and adult gastroenterology clinics and pediatric oncology units at Fletcher Allen Health Care, University of Vermont, and other participating Pediatric Oncology Groups/Children’s Oncology Group institutions [29,30]. Informed consent was obtained from all subjects following procedures approved by the Committee on Human Research at the University of Vermont and participating institutions of the cooperative Pediatric Oncology Group/Children’s Oncology Groups.

### Microsatellite assay

The amplifications of 11 microsatellite loci are performed in two multiplex PCR reactions, consisting of 6 and 5 sets of forward and reverse primers that are either labeled with fluorescent tags Hex, Fam or Ned, or tailed with GTGTCTT nucleotides. Previous research demonstrated that Taq polymerase can catalyze non-templated 3^′^ terminal nucleotide addition, primarily adenosine, to amplified DNA fragments. This activity depends on adjacent DNA sequence context [31-33], with preferential adenylation following 3^′^-cytidine or thymidine, resulting in genotyping errors due to variations in the fractions of adenylated products. Such variations were improved by performing PCR reactions under conditions that either favored the production of the true allele (two-step PCR protocols) or conditions that produced primarily the adenylated products (three-step PRC protocols with longer extension times up to 90 min; true allele plus A) for consistent genotyping. Brownstein further showed that adding specific generic tails of 6–7 nucleotides to the 5^′^ end of reverse primers more consistently promoted the adenylation of amplified DNA products [34]. In our experiments, we have added a GTGTCTT tail, previously tested by Brownstein, to the 5^′^ ends of all unlabeled primers and performed PCR reactions under favorable conditions to promote the adenylation of the majority of PCR products.

To accommodate variations in microsatellite size among individuals (range listed in Table 3) and possible instability, the analyses of multiple loci within the same PCR reaction are made possible by designing primers, when labeled with similar fluorescent dyes to prime products that are at least 40 nucleotides apart in size. Finally, sample requirement is minimized by the miniaturization of PCR reactions in 10 μL volume.

### DNA samples

DNA templates from both purified DNA as well as crude cell lysates have been utilized in PCR reactions. Adequate fluorescence units have been achieved with amplifications of as low as 0.75 ng of purified DNA and 0.5 μL of lysates of 10,000 T cell pellets. Cell lysates are prepared by adding 10 μL of lysis reagent containing 80% Qiagen EB buffer (10 mM Tris–HCl pH 8.5; Qiagen, Inc., Valencia, USA), 0.5% Tween 20, 0.5% NP40, and 0.1 mg/mL of proteinase K to cell pellets, followed by incubation at 56°C for 1 h and heat inactivation at 96°C for 10 min.

### PCR conditions

PCR reactions are performed with 10 mM Tris–HCl pH 8.3, 50 mM KCl, 1.5 mM MgCl2, 0.25U or 0.025 U/μL of platinum (Invitrogen Corporation, Grand Island, USA) or HotStart-It Taq polymerase (USB Corporation, Cleveland, USA), primers at concentrations listed in Table 3, and DNA templates. Reaction mixtures are heated to 94°C for 2 min for enzyme activation and then for 35 cycles (94°C for 30 s, 55°C for 30 s, and 72°C for 40 s) with a final extension at 72°C for 10 min.

### Microsatellite detection and analysis

Amplified products from PCR reactions 1 and 2 are diluted with water. A volume of 1 μL of diluted products are mixed with 10 μL of formamide and 0.4 μL of GeneScan 500 Rox size standards (Invitrogen Corporation), ranging from 35–500 nucleotides, and heat denatured at 95°C for 5 min. PCR products and internal standard mixtures are then electrophoresed using the ABI 3100 Avant Genetic Analyzer (Invitrogen Corporation). Subsequently, automated allele identification is performed with the GeneMapper software, in which markers and method parameters (Tables 1 and 2, respectively) have been empirically optimized for specific peak detection and precise sizing of amplified products, using the Advanced Peak Detection Algorithm and Local Southern sizing method, respectively.

## Abbreviations

HNPCC: Hereditary nonpolyposis colorectal cancer; LOH: Loss of heterozygosity; MSI: Microsatellite instability.

## Competing interests

The authors declare that they have no competing interests.

## Authors’ contributions

All authors participated in the preparation of the manuscript, and read and approved the final manuscript.

## Supplementary Material

Additional file 1 **Summary of all samples and clones that have been amplified with optimized PCR conditions using groups I and II microsatellite markers. **Raw data from these samples have been used to optimize marker and method parameters to achieve specific peak detection, precise fragment sizing, and accurate automated allele identification. Sizing values and allele calls for 5,508 alleles have been automatically analyzed with optimized parameters and bin settings, using the Advanced Peak Detection Algorithm in combination with the Local Southern sizing method. Samples or clones with asterisk represent those that have been miscalled by automated allele identification. Click here for file

Additional file 2 **Process reproducibility with group I markers. **Summary of sizing values of identified alleles for each marker from the amplification of control samples in 23 independent runs over the course of 5 months. Averages, standard deviations, and %CVs have been calculated for each allele. Process variation includes sample preparation, PCR amplification, fragment separation, peak detection, and fragment sizing. Click here for file

Additional file 3 **Process reproducibility with group II markers. **Summary of sizing values of identified alleles for each marker from the amplification of control samples in 18 independent runs over the course of 1 month. Averages, standard deviations, and %CVs have been calculated for each allele. Process variation include sample preparation, PCR amplification, fragment separation, peak detection, and fragment sizing. Click here for file

Additional file 4 **Process accuracy with group I markers. **Summary of results from automated allele identification of 1,920 dinucleotide markers and 960 mononucleotide markers from 239 T cell clones from different subjects organized by dates of processing. Click here for file

Additional file 5 **Process accuracy with group II markers. **Summary of results from automated allele identification of 1,560 dinucleotide markers from 156 T cell clones from different subjects organized by dates of processing. Percent deviation from expected values connotes the percent of alleles that have been miscalled, indicated by an asterisk. Misidentification can represent failure in any part of the process from sample preparation, PCR amplification, fragment separation, peak detection, fragment sizing, to automated allele calling. We manually analyzed all discordant samples and found failures to be related to either the interferences of nonspecific signals above threshold values or from the unequal amplification of alleles. These failures were likely related to poor sample quality since control samples were performed optimally in the same runs. Click here for file
