# Supplementary material for: Development of a cost-effective high-throughput process of microsatellite analysis involving miniaturized multiplexed PCR amplification and automated allele identification
Source: Hum Genomics. 2013 Mar 5;7(1):6. doi: 10.1186/1479-7364-7-6 (PMC3600708; doi:10.1186/1479-7364-7-6)
Supplement: Additional file 1 — Summary of all samples and clones that have been amplified with optimized PCR conditions using groups I and II microsatellite markers. Raw data from these samples have been used to optimize marker and method parameters to achieve specific peak detection, precise fragment sizing, and accurate automated allele identification. Sizing values and allele calls for 5,508 alleles have been automatically analyzed with optimized parameters and bin settings, using the Advanced Peak Detection Algorithm in combination with the Local Southern sizing method. Samples or clones with asterisk represent those that have been miscalled by automated allele identification. [file 1479-7364-7-6-S1.docx]

**Additional file 1. Summary of all samples and clones that have been amplified with optimized PCR conditions using groups I and II microsatellite markers.**

**Group I Microsatellite Markers Bat25, Bat26, D3S3623, D5S346, D6S262, D7S481**

| **Day** | **Count** | **Run Date** | **Sample Name** | **Marker** | **Allele 1** | **Allele 2** | **Size 1** | **Size 2** | **Height 1** | **Height 2** | **Peak Area 1** | **Peak Area 2** | **Data Point 1** | **Data Point 2** |
| --- | --- | --- | --- | --- | --- | --- | --- | --- | --- | --- | --- | --- | --- | --- |
| **1** | **1**  **1**  **1**  **1**  **1**  **1** | **6/20/05**  **6/20/05**  **6/20/05**  **6/20/05**  **6/20/05**  **6/20/05** | **UC001B1WT1**  **UC001B1WT1**  **UC001B1WT1**  **UC001B1WT1**  **UC001B1WT1**  **UC001B1WT1** | **BAT25**  **BAT26**  **D3S3623**  **D5S346**  **D6S262**  **D7S481** | **127**  **122**  **219**  **94**  **170**  **193** | **225**  **182**  **203** | **127.0**  **121.9**  **218.7**  **94.1**  **170.5**  **193.2** | **225.0**  **182.1**  **203.0** | **1265**  **645**  **1032**  **1058**  **955**  **525** | **798**  **754**  **448** | **9380**  **5477**  **8861**  **8760**  **7746**  **4557** | **6879**  **5951**  **3578** | **3489**  **3422**  **4616**  **3048**  **4025**  **4314** | **4689**  **4173**  **4435** |
| **1** | **1**  **2**  **3**  **4**  **5** | **6/30/05**  **6/30/05**  **6/30/05**  **6/30/05**  **6/30/05** | **UC002B1M10**  **UC002B1M13**  **UC002B1M18**  **UC002B1M19**  **UC002B1M15** | **BAT25**  **BAT25**  **BAT25**  **BAT25**  **BAT25** | **128**  **128**  **128**  **127**  **128** | **128** | **128.0**  **127.9**  **127.9**  **126.9**  **127.9** | **127.9** | **2069**  **3356**  **3983**  **3089**  **2680** | **3230** | **14692**  **22927**  **27636**  **21129**  **19046** | **22839** | **3416**  **3392**  **3374**  **3354**  **3387** | **3367** |
| **1** | **1**  **2**  **3**  **4**  **5** | **6/30/05**  **6/30/05**  **6/30/05**  **6/30/05**  **6/30/05** | **UC002B1M10**  **UC002B1M13**  **UC002B1M18**  **UC002B1M19**  **UC002B1M15** | **BAT26**  **BAT26**  **BAT26**  **BAT26**  **BAT26** | **122**  **122**  **122**  **122**  **122** |  | **121.9**  **121.8**  **121.8**  **121.8**  **121.8** |  | **834**  **1152**  **1347**  **1210**  **1110** |  | **7061**  **10490**  **12492**  **11217**  **10722** |  | **3338**  **3314**  **3296**  **3290**  **3310** |  |
| **1** | **1**  **2**  **3**  **4**  **5** | **6/30/05**  **6/30/05**  **6/30/05**  **6/30/05**  **6/30/05** | **UC002B1M10**  **UC002B1M13**  **UC002B1M18**  **UC002B1M19**  **UC002B1M15** | **D3S3623**  **D3S3623**  **D3S3623**  **D3S3623**  **D3S3623** | **219**  **219**  **219**  **219**  **219** | **225**  **225**  **225**  **225**  **225** | **218.7**  **218.6**  **218.5**  **218.6**  **218.6** | **225.0**  **224.8**  **224.8**  **224.8**  **224.8** | **1972**  **3247**  **4747**  **3857**  **3186** | **1418**  **2373**  **3209**  **2696**  **2153** | **17181**  **27308**  **40702**  **32835**  **27366** | **12244**  **20529**  **27494**  **22756**  **18228** | **4504**  **4477**  **4453**  **4443**  **4468** | **4575**  **4546**  **4523**  **4512**  **4537** |
| **1** | **1**  **2**  **3**  **4**  **5** | **6/30/05**  **6/30/05**  **6/30/05**  **6/30/05**  **6/30/05** | **UC002B1M10**  **UC002B1M13**  **UC002B1M18**  **UC002B1M19**  **UC002B1M15** | **D5S346**  **D5S346**  **D5S346**  **D5S346**  **D5S346** | **103**  **103**  **103**  **103**  **103** | **105**  **105**  **105**  **105**  **105** | **102.7**  **102.7**  **102.6**  **102.5**  **102.7** | **104.8**  **104.7**  **104.7**  **104.6**  **104.7** | **1139**  **1524**  **1828**  **1555**  **1404** | **658**  **959**  **1115**  **905**  **875** | **8820**  **11588**  **13843**  **12080**  **10932** | **5005**  **6808**  **8038**  **6525**  **6222** | **3085**  **3062**  **3044**  **3038**  **3058** | **3113**  **3089**  **3072**  **3066**  **3085** |
| **1** | **1**  **2**  **3**  **4**  **5** | **6/30/05**  **6/30/05**  **6/30/05**  **6/30/05**  **6/30/05** | **UC002B1M15**  **UC002B1M10**  **UC002B1M13**  **UC002B1M18**  **UC002B1M19** | **D6S262**  **D6S262**  **D6S262**  **D6S262**  **D6S262** | **182**  **182**  **182**  **182**  **182** |  | **181.9**  **182.0**  **182.0**  **181.9**  **181.9** |  | **3645**  **2409**  **3971**  **5329**  **4451** |  | **29399**  **19505**  **33101**  **42627**  **35784** |  | **4038**  **4072**  **4046**  **4024**  **4015** |  |
| **1** | **1**  **2**  **3**  **4**  **5** | **6/30/05**  **6/30/05**  **6/30/05**  **6/30/05**  **6/30/05** | **UC002B1M15**  **UC002B1M10**  **UC002B1M13**  **UC002B1M18**  **UC002B1M19** | **D7S481**  **D7S481**  **D7S481**  **D7S481**  **D7S481** | **201**  **201**  **201**  **201**  **201** |  | **200.7**  **200.9**  **200.7**  **200.7**  **200.7** |  | **1421**  **960**  **1496**  **2003**  **1801** |  | **12935**  **8721**  **13725**  **18085**  **16079** |  | **4268**  **4304**  **4276**  **4254**  **4244** |  |
| **1** | **1**  **1**  **1**  **1**  **1**  **1** | **6/20/05**  **6/20/05**  **6/20/05**  **6/20/05**  **6/20/05**  **6/20/05** | **UC003B1WT1**  **UC003B1WT1**  **UC003B1WT1**  **UC003B1WT1**  **UC003B1WT1**  **UC003B1WT1** | **BAT25**  **BAT26**  **D3S3623**  **D5S346**  **D6S262**  **D7S481** | **127**  **122**  **221**  **94**  **172**  **187** | **225**  **103**  **195** | **126.9**  **121.8**  **220.7**  **94.0**  **172.4**  **187.3** | **224.9**  **102.7**  **195.1** | **1170**  **565**  **1861**  **422**  **3171**  **785** | **1430**  **296**  **599** | **8159**  **4867**  **15404**  **3203**  **27136**  **7091** | **11551**  **2052**  **5370** | **3468**  **3401**  **4611**  **3029**  **4026**  **4214** | **4659**  **3144**  **4311** |
| **1** | **1**  **2**  **3** | **6/30/05**  **6/30/05**  **6/30/05** | **UC005B1M18**  **UC005B1M3**  **UC005B1M5** | **BAT25**  **BAT25**  **BAT25** | **126**  **127**  **126** | **127**  **127** | **125.8**  **126.8**  **125.7** | **126.9**  **126.8** | **2651**  **2910**  **2963** | **2783**  **3093** | **19352**  **21789**  **21597** | **20126**  **23465** | **3321**  **3351**  **3331** | **3334**  **3344** |
| **1** | **1**  **2**  **3** | **6/30/05**  **6/30/05**  **6/30/05** | **UC005B1M18**  **UC005B1M3**  **UC005B1M5** | **BAT26**  **BAT26**  **BAT26** | **123**  **123**  **123** |  | **122.8**  **122.7**  **122.6** |  | **589**  **777**  **729** |  | **4864**  **6286**  **6486** |  | **3282**  **3299**  **3291** |  |
| **1** | **1**  **2**  **3** | **6/30/05**  **6/30/05**  **6/30/05** | **UC005B1M18**  **UC005B1M3**  **UC005B1M5** | **D3S3623**  **D3S3623**  **D3S3623** | **225**  **225**  **225** | **227**  **227**  **227** | **224.7**  **224.7**  **224.7** | **226.8**  **226.8**  **226.7** | **2957**  **2719**  **2978** | **1778**  **1672**  **1803** | **23983**  **21709**  **23775** | **13700**  **12754**  **13874** | **4491**  **4511**  **4499** | **4514**  **4534**  **4522** |
| **1** | **1**  **2**  **3** | **6/30/05**  **6/30/05**  **6/30/05** | **UC005B1M18**  **UC005B1M3**  **UC005B1M5** | **D5S346**  **D5S346**  **D5S346** | **92**  **92**  **92** | **103**  **103**  **103** | **91.7**  **91.7**  **91.6** | **102.6**  **102.6**  **102.5** | **1539**  **1802**  **1880** | **663**  **736**  **753** | **13041**  **14988**  **16220** | **5061**  **5564**  **6028** | **2879**  **2895**  **2889** | **3019**  **3036**  **3029** |
| **1** | **1**  **2** | **6/30/05**  **6/30/05** | **UC005B1M18**  **UC005B1M3** | **D6S262**  **D6S262** | **172**  **172** | **182**  **182** | **172.2**  **172.3** | **181.8**  **181.9** | **2026**  **1928** | **1199**  **1233** | **16767**  **15244** | **9312**  **9585** | **3876**  **3895** | **3994**  **4014** |

| **Day** | **Count** | **Run Date** | **Sample Name** | **Marker** | **Allele 1** | **Allele 2** | **Size 1** | **Size 2** | **Height 1** | **Height 2** | **Peak Area 1** | **Peak Area 2** | **Data Point 1** | **Data Point 2** |
| --- | --- | --- | --- | --- | --- | --- | --- | --- | --- | --- | --- | --- | --- | --- |
|  | **3** | **6/30/05** | **UC005B1M5** | **D6S262** | **172** | **182** | **172.2** | **181.9** | **2106** | **1378** | **17204** | **10837** | **3886** | **4004** |
| **1** | **1**  **2**  **3** | **6/30/05**  **6/30/05**  **6/30/05** | **UC005B1M18**  **UC005B1M3**  **UC005B1M5** | **D7S481**  **D7S481**  **D7S481** | **199**  **199**  **199** | **203**  **203**  **203** | **198.7**  **198.7**  **198.7** | **202.8**  **202.8**  **202.7** | **671**  **589**  **695** | **484**  **452**  **527** | **5495**  **4715**  **5695** | **3849**  **3443**  **4028** | **4200**  **4219**  **4209** | **4247**  **4266**  **4255** |
| **1** | **1**  **2**  **3** | **6/30/05**  **6/30/05**  **6/30/05** | **UC006B1M5**  **UC006B1M9**  **UC006B1M15** | **BAT25**  **BAT25**  **BAT25** | **127**  **127**  **127** |  | **126.8**  **126.8**  **126.8** |  | **635**  **5672**  **1481** |  | **4914**  **42412**  **10842** |  | **3328**  **3341**  **3331** |  |
| **1** | **1**  **2**  **3** | **6/30/05**  **6/30/05**  **6/30/05** | **UC006B1M5**  **UC006B1M9**  **UC006B1M15** | **BAT26**  **BAT26**  **BAT26** | **122**  **122**  **122** |  | **121.7**  **121.6**  **121.7** |  | **228**  **1297**  **557** |  | **1870**  **11027**  **4846** |  | **3263**  **3276**  **3266** |  |
| **1** | **1**  **2** | **6/30/05**  **6/30/05** | **UC006B1M5**  **UC006B1M9** | **D3S3623**  **D3S3623** | **219**  **219** | **223**  **223** | **218.5**  **218.5** | **222.6**  **222.6** | **462**  **4394** | **218**  **3057** | **3736**  **35535** | **1646**  **24251** | **4412**  **4430** | **4457**  **4476** |
|  | **3** | **6/30/05** | **UC006B1M15** | **D3S3623** | **219** | **223** | **218.5** | **222.6** | **1292** | **895** | **10547** | **7040** | **4414** | **4460** |
| **1** | **1**  **2**  **3** | **6/30/05**  **6/30/05**  **6/30/05** | **UC006B1M5**  **UC006B1M9**  **UC006B1M15** | **D5S346**  **D5S346**  **D5S346** | **92**  **92**  **92** | **105**  **105**  **105** | **91.7**  **91.6**  **91.7** | **104.6**  **104.7**  **104.6** | **596**  **3072**  **1930** | **318**  **1072**  **878** | **4950**  **25119**  **15942** | **2562**  **8516**  **7144** | **2874**  **2885**  **2877** | **3041**  **3054**  **3044** |
| **1** | **1**  **2**  **3** | **6/30/05**  **6/30/05**  **6/30/05** | **UC006B1M5**  **UC006B1M9**  **UC006B1M15** | **D6S262**  **D6S262**  **D6S262** | **179**  **179**  **179** | **184**  **184**  **184** | **179.1**  **179.1**  **179.2** | **183.8**  **183.8**  **183.9** | **455**  **3469**  **1675** | **291**  **2074**  **845** | **3682**  **30558**  **15146** | **2184**  **15476**  **6371** | **3951**  **3968**  **3955** | **4009**  **4026**  **4013** |
| **1** | **1**  **2**  **3** | **6/30/05**  **6/30/05**  **6/30/05** | **UC006B1M5**  **UC006B1M9**  **UC006B1M15** | **D7S481**  **D7S481**  **D7S481** | **193**  **193**  **193** | **201**  **201**  **201** | **192.9**  **193.0**  **193.1** | **200.6**  **200.6**  **200.6** | **149**  **814**  **283** | **140**  **656**  **230** | **1257**  **7189**  **2554** | **1349**  **5823**  **2127** | **4120**  **4138**  **4125** | **4213**  **4231**  **4216** |
| **1** | **1**  **2**  **3** | **6/30/05**  **6/30/05**  **6/30/05** | **UC009B1M6**  **UC009B1M8**  **UC009B1M11** | **BAT25**  **BAT25**  **BAT25** | **127**  **127**  **127** |  | **126.9**  **126.8**  **126.9** |  | **4114**  **3909**  **3085** |  | **30655**  **28809**  **23039** |  | **3350**  **3343**  **3318** |  |
| **1** | **1**  **2**  **3** | **6/30/05**  **6/30/05**  **6/30/05** | **UC009B1M6**  **UC009B1M8**  **UC009B1M11** | **BAT26**  **BAT26**  **BAT26** | **123**  **123**  **123** |  | **122.7**  **122.7**  **122.7** |  | **1039**  **1169**  **790** |  | **8744**  **10033**  **6581** |  | **3297**  **3291**  **3266** |  |
| **1** | **1**  **2**  **3** | **6/30/05**  **6/30/05**  **6/30/05** | **UC009B1M6**  **UC009B1M8**  **UC009B1M11** | **D3S3623**  **D3S3623**  **D3S3623** | **219**  **219**  **219** | **221**  **221**  **221** | **218.5**  **218.5**  **218.4** | **220.6**  **220.6**  **220.5** | **4635**  **4152**  **3724** | **3069**  **2697**  **2360** | **36964**  **33449**  **29843** | **23611**  **20694**  **17743** | **4441**  **4429**  **4398** | **4464**  **4452**  **4421** |
| **1** | **1**  **2**  **3** | **6/30/05**  **6/30/05**  **6/30/05** | **UC009B1M6**  **UC009B1M8**  **UC009B1M11** | **D5S346**  **D5S346**  **D5S346** | **94**  **94**  **94** |  | **93.8**  **93.8**  **93.9** |  | **4153**  **4437**  **2993** |  | **33835**  **36908**  **24426** |  | **2921**  **2916**  **2893** |  |
| **1** | **1**  **2**  **3** | **6/30/05**  **6/30/05**  **6/30/05** | **UC009B1M6**  **UC009B1M8**  **UC009B1M11** | **D6S262**  **D6S262**  **D6S262** | **170**  **170**  **170** | **172**  **172**  **172** | **170.3**  **170.3**  **170.2** | **172.3**  **172.2**  **172.2** | **4129**  **4510**  **3516** | **2827**  **3009**  **2355** | **31429**  **34597**  **26242** | **20014**  **21637**  **17024** | **3869**  **3861**  **3833** | **3893**  **3884**  **3857** |
| **1** | **1**  **2**  **3** | **6/30/05**  **6/30/05**  **6/30/05** | **UC009B1M6**  **UC009B1M11**  **UC009B1M8** | **D7S481**  **D7S481**  **D7S481** | **203**  **203**  **203** |  | **202.8**  **202.7**  **202.8** |  | **1362**  **1209**  **1302** |  | **11605**  **10443**  **11337** |  | **4265**  **4224**  **4254** |  |
| **1** | **1**  **1**  **1**  **1**  **1**  **1** | **6/20/05**  **6/20/05**  **6/20/05**  **6/20/05**  **6/20/05**  **6/20/05** | **UC018B1WT6**  **UC018B1WT6**  **UC018B1WT6**  **UC018B1WT6* UC018B1WT6**  **UC018B1WT6** | **BAT25**  **BAT26**  **D3S3623**  **D5S346* D6S262**  **D7S481** | **127**  **122**  **219**  **92**  **172**  **195** | **227**  **94**  **186**  **201** | **126.9**  **121.9**  **218.7**  **91.7**  **172.4**  **195.1** | **227.0**  **94.0**  **185.9**  **200.8** | **1686**  **728**  **2520**  **746**  **2068**  **804** | **1874**  **514**  **1287**  **707** | **12333**  **6503**  **21136**  **5782**  **16601**  **6934** | **15819**  **3489**  **10300**  **6398** | **3457**  **3392**  **4575**  **2990**  **4014**  **4299** | **4670**  **3020**  **4184**  **4370** |
| **1** | **1**  **2**  **3**  **4**  **5**  **6**  **7**  **8**  **9**  **10**  **11** | **7/5/05**  **7/5/05**  **7/5/05**  **7/5/05**  **7/5/05**  **7/5/05**  **7/5/05**  **7/5/05**  **7/5/05**  **7/5/05**  **7/5/05** | **CD3B1_2M9**  **CD3B1_2M19**  **CD3B1_2M35**  **CD3B1_2M2**  **CD3B1_2M10**  **CD3B1_2M20**  **CD3B1_2M28**  **CD3B1_2M36**  **CD3B1_2M3**  **CD3B1_2M11**  **CD3B1_2M21** | **BAT25**  **BAT25**  **BAT25**  **BAT25**  **BAT25**  **BAT25**  **BAT25**  **BAT25**  **BAT25**  **BAT25**  **BAT25** | **127**  **127**  **127**  **127**  **127**  **127**  **127**  **127**  **127**  **127**  **127** | **128** | **126.7**  **126.7**  **126.7**  **126.8**  **126.7**  **126.7**  **126.7**  **126.7**  **126.8**  **126.7**  **126.7** | **127.8** | **6102**  **3455**  **4523**  **2493**  **5877**  **1859**  **4190**  **3020**  **3942**  **3939**  **1095** | **5859** | **53465**  **26623**  **34771**  **19386**  **45731**  **14322**  **31789**  **23764**  **32224**  **31363**  **8963** | **41605** | **3374**  **3366**  **3377**  **3406**  **3342**  **3336**  **3346**  **3348**  **3431**  **3390**  **3384** | **3387** |

| **Day** | **Count** | **Run Date** | **Sample Name** | **Marker** | **Allele 1** | **Allele 2** | **Size 1** | **Size 2** | **Height 1** | **Height 2** | **Peak Area 1** | **Peak Area 2** | **Data Point 1** | **Data Point 2** |
| --- | --- | --- | --- | --- | --- | --- | --- | --- | --- | --- | --- | --- | --- | --- |
|  | **12**  **13**  **14**  **15**  **16**  **17** | **7/5/05**  **7/5/05**  **7/5/05**  **7/5/05**  **7/5/05**  **7/5/05** | **CD3B1_2M37**  **CD3B1_2M12**  **CD3B1_2M22**  **CD3B1_2M30**  **CD3B1_2M38**  **CD3B1_2M5** | **BAT25**  **BAT25**  **BAT25**  **BAT25**  **BAT25**  **BAT25** | **127**  **127**  **127**  **127**  **127**  **126** | **127** | **126.7**  **126.7**  **126.7**  **126.8**  **126.8**  **125.7** | **126.7** | **3306**  **6235**  **5460**  **1410**  **2844**  **5586** | **5835** | **26074**  **50533**  **42388**  **11197**  **22038**  **47446** | **63024** | **3386**  **3382**  **3374**  **3392**  **3390**  **3364** | **3377** |
|  | **18**  **19**  **20**  **21**  **22**  **23**  **24**  **25**  **26**  **27**  **28**  **29**  **30**  **31**  **32**  **33**  **34** | **7/5/05**  **7/5/05**  **7/5/05**  **7/5/05**  **7/5/05**  **7/5/05**  **7/5/05**  **7/5/05**  **7/5/05**  **7/5/05**  **7/5/05**  **7/5/05**  **7/5/05**  **7/5/05**  **7/5/05**  **7/5/05**  **7/5/05** | **CD3B1_2M13**  **CD3B1_2M23**  **CD3B1_2M31**  **CD3B1_2M39**  **CD3B1_2M6**  **CD3B1_2M14**  **CD3B1_2M24**  **CD3B1_2M32**  **CD3B1_2M40**  **CD3B1_2M7**  **CD3B1_2M16**  **CD3B1_2M25**  **CD3B1_2M33**  **CD3B1_2M41**  **CD3B1_2M8**  **CD3B1_2M17**  **CD3B1_2M26** | **BAT25**  **BAT25**  **BAT25**  **BAT25**  **BAT25**  **BAT25**  **BAT25**  **BAT25**  **BAT25**  **BAT25**  **BAT25**  **BAT25**  **BAT25**  **BAT25**  **BAT25**  **BAT25**  **BAT25** | **127**  **127**  **127**  **127**  **127**  **127**  **127**  **127**  **127**  **127**  **127**  **127**  **127**  **127**  **126**  **127**  **127** | **127** | **126.7**  **126.8**  **126.8**  **126.8**  **126.7**  **126.7**  **126.7**  **126.8**  **126.8**  **126.8**  **126.7**  **126.7**  **126.8**  **126.7**  **125.7**  **126.7**  **126.7** | **126.7** | **4071**  **1675**  **2950**  **2813**  **5156**  **3067**  **2074**  **3279**  **665**  **5287**  **2600**  **890**  **1609**  **464**  **5433**  **5694**  **2337** | **5674** | **31972**  **13427**  **22804**  **22147**  **40503**  **23695**  **16211**  **25015**  **5072**  **43581**  **20288**  **7285**  **12218**  **3875**  **43022**  **43893**  **18602** | **57907** | **3378**  **3360**  **3397**  **3366**  **3346**  **3341**  **3330**  **3367**  **3339**  **3390**  **3381**  **3376**  **3410**  **3370**  **3372**  **3378**  **3376** | **3385** |
| **2** | **35**  **36**  **37**  **38** | **7/5/05**  **7/5/05**  **7/15/05**  **7/15/05** | **CD3B1_2M34**  **CD3B1_2M42**  **CD003B1_2M4**  **CD003B1_2M27** | **BAT25**  **BAT25**  **BAT25**  **BAT25** | **127**  **127**  **127**  **127** |  | **126.7**  **126.8**  **126.9**  **127.0** |  | **4576**  **1664**  **1433**  **838** |  | **35609**  **12978**  **11635**  **7386** |  | **3411**  **3376**  **3583**  **3590** |  |
| **1** | **1**  **2**  **3**  **4**  **5**  **6**  **7**  **8**  **9**  **10**  **11**  **12**  **13**  **14**  **15**  **16**  **17**  **18**  **19**  **20**  **21**  **22**  **23**  **24**  **25**  **26**  **27**  **28**  **29**  **30**  **31** | **7/5/05**  **7/5/05**  **7/5/05**  **7/5/05**  **7/5/05**  **7/5/05**  **7/5/05**  **7/5/05**  **7/5/05**  **7/5/05**  **7/5/05**  **7/5/05**  **7/5/05**  **7/5/05**  **7/5/05**  **7/5/05**  **7/5/05**  **7/5/05**  **7/5/05**  **7/5/05**  **7/5/05**  **7/5/05**  **7/5/05**  **7/5/05**  **7/5/05**  **7/5/05**  **7/5/05**  **7/5/05**  **7/5/05**  **7/5/05**  **7/5/05** | **CD3B1_2M9**  **CD3B1_2M19**  **CD3B1_2M35**  **CD3B1_2M2**  **CD3B1_2M10**  **CD3B1_2M20**  **CD3B1_2M28**  **CD3B1_2M36**  **CD3B1_2M3**  **CD3B1_2M11**  **CD3B1_2M21**  **CD3B1_2M37**  **CD3B1_2M12**  **CD3B1_2M22**  **CD3B1_2M30**  **CD3B1_2M38**  **CD3B1_2M5**  **CD3B1_2M13**  **CD3B1_2M23**  **CD3B1_2M31**  **CD3B1_2M39**  **CD3B1_2M6**  **CD3B1_2M14**  **CD3B1_2M24**  **CD3B1_2M32**  **CD3B1_2M40**  **CD3B1_2M7**  **CD3B1_2M16**  **CD3B1_2M25**  **CD3B1_2M33**  **CD3B1_2M41** | **BAT26**  **BAT26**  **BAT26**  **BAT26**  **BAT26**  **BAT26**  **BAT26**  **BAT26**  **BAT26**  **BAT26**  **BAT26**  **BAT26**  **BAT26**  **BAT26**  **BAT26**  **BAT26**  **BAT26**  **BAT26**  **BAT26**  **BAT26**  **BAT26**  **BAT26**  **BAT26**  **BAT26**  **BAT26**  **BAT26**  **BAT26**  **BAT26**  **BAT26**  **BAT26**  **BAT26** | **122**  **122**  **122**  **122**  **122**  **122**  **122**  **122**  **122**  **122**  **122**  **122**  **122**  **122**  **122**  **122**  **122**  **122**  **122**  **122**  **122**  **122**  **122**  **122**  **122**  **121**  **122**  **122**  **122**  **122**  **122** | **122** | **121.7**  **121.7**  **121.7**  **121.7**  **121.7**  **121.7**  **121.7**  **121.7**  **121.7**  **121.6**  **121.7**  **121.7**  **121.6**  **121.7**  **121.7**  **121.7**  **121.7**  **121.7**  **121.6**  **121.7**  **121.7**  **121.7**  **121.7**  **121.6**  **121.7**  **120.7**  **121.8**  **121.7**  **121.7**  **121.7**  **121.7** | **121.7** | **2215**  **1025**  **1415**  **954**  **1938**  **618**  **1204**  **1042**  **1558**  **1458**  **253**  **979**  **2069**  **1667**  **602**  **891**  **3024**  **889**  **447**  **1046**  **968**  **2067**  **1109**  **733**  **1216**  **269**  **1667**  **731**  **309**  **470**  **331** | **277** | **21948**  **9744**  **13673**  **9330**  **19827**  **5762**  **12806**  **10421**  **15423**  **15798**  **2390**  **9511**  **20632**  **16206**  **5461**  **8573**  **33985**  **8764**  **4339**  **9808**  **8810**  **21425**  **11165**  **7128**  **10953**  **2252**  **17431**  **7017**  **2807**  **4336**  **3108** | **2300** | **3309**  **3302**  **3312**  **3339**  **3278**  **3271**  **3282**  **3284**  **3365**  **3324**  **3319**  **3321**  **3317**  **3309**  **3326**  **3325**  **3312**  **3313**  **3294**  **3332**  **3301**  **3282**  **3277**  **3265**  **3302**  **3262**  **3326**  **3316**  **3312**  **3344**  **3306** | **3275** |

| **Day** | **Count** | **Run Date** | **Sample Name** | **Marker** | **Allele 1** | **Allele 2** | **Size 1** | **Size 2** | **Height 1** | **Height 2** | **Peak Area 1** | **Peak Area 2** | **Data Point 1** | **Data Point 2** |
| --- | --- | --- | --- | --- | --- | --- | --- | --- | --- | --- | --- | --- | --- | --- |
| **2** | **32**  **33**  **34**  **35**  **36**  **37**  **38** | **7/5/05**  **7/5/05**  **7/5/05**  **7/5/05**  **7/5/05**  **7/15/05**  **7/15/05** | **CD3B1_2M8**  **CD3B1_2M17**  **CD3B1_2M26**  **CD3B1_2M34**  **CD3B1_2M42**  **CD003B1_2M4**  **CD003B1_2M27** | **BAT26**  **BAT26**  **BAT26**  **BAT26**  **BAT26**  **BAT26**  **BAT26** | **122**  **122**  **122**  **122**  **122**  **122**  **122** |  | **121.8**  **121.7**  **121.7**  **121.7**  **121.7**  **121.9**  **121.9** |  | **3676**  **2128**  **929**  **1515**  **553**  **688**  **282** |  | **37770**  **21016**  **9028**  **14910**  **5115**  **5846**  **2319** |  | **3321**  **3313**  **3311**  **3346**  **3310**  **3516**  **3523** |  |
| **1** | **1**  **2** | **7/5/05**  **7/5/05** | **CD3B1_2M9**  **CD3B1_2M19** | **D3S3623**  **D3S3623** | **219**  **219** |  | **218.6**  **218.5** |  | **7519**  **2888** |  | **68213**  **25548** |  | **4481**  **4471** |  |
|  | **3**  **4**  **5**  **6**  **7**  **8**  **9**  **10**  **11**  **12**  **13**  **14**  **15**  **16**  **17**  **18**  **19**  **20**  **21**  **22**  **23**  **24**  **25**  **26**  **27** | **7/5/05**  **7/5/05**  **7/5/05**  **7/5/05**  **7/5/05**  **7/5/05**  **7/5/05**  **7/5/05**  **7/5/05**  **7/5/05**  **7/5/05**  **7/5/05**  **7/5/05**  **7/5/05**  **7/5/05**  **7/5/05**  **7/5/05**  **7/5/05**  **7/5/05**  **7/5/05**  **7/5/05**  **7/5/05**  **7/5/05**  **7/5/05**  **7/5/05** | **CD3B1_2M35**  **CD3B1_2M2**  **CD3B1_2M10**  **CD3B1_2M20**  **CD3B1_2M28**  **CD3B1_2M36**  **CD3B1_2M3**  **CD3B1_2M11**  **CD3B1_2M21**  **CD3B1_2M37**  **CD3B1_2M12**  **CD3B1_2M22**  **CD3B1_2M30**  **CD3B1_2M38**  **CD3B1_2M5**  **CD3B1_2M13**  **CD3B1_2M23**  **CD3B1_2M31**  **CD3B1_2M39**  **CD3B1_2M6**  **CD3B1_2M14**  **CD3B1_2M24**  **CD3B1_2M32**  **CD3B1_2M40**  **CD3B1_2M7** | **D3S3623**  **D3S3623**  **D3S3623**  **D3S3623**  **D3S3623**  **D3S3623**  **D3S3623**  **D3S3623**  **D3S3623**  **D3S3623**  **D3S3623**  **D3S3623**  **D3S3623**  **D3S3623**  **D3S3623**  **D3S3623**  **D3S3623**  **D3S3623**  **D3S3623**  **D3S3623**  **D3S3623**  **D3S3623**  **D3S3623**  **D3S3623**  **D3S3623** | **219**  **219**  **219**  **219**  **219**  **219**  **219**  **219**  **219**  **219**  **219**  **219**  **219**  **219**  **219**  **219**  **219**  **219**  **219**  **219**  **219**  **219**  **219**  **219**  **219** |  | **218.5**  **218.6**  **218.5**  **218.5**  **218.5**  **218.5**  **218.7**  **218.4**  **218.5**  **218.5**  **218.5**  **218.6**  **218.5**  **218.5**  **218.5**  **218.5**  **218.5**  **218.5**  **218.6**  **218.5**  **218.6**  **218.4**  **218.5**  **218.6**  **218.4** |  | **6977**  **3224**  **7499**  **2696**  **6963**  **4826**  **6194**  **7453**  **1323**  **6153**  **7680**  **7573**  **1455**  **5947**  **8644**  **5757**  **2891**  **4820**  **6061**  **7426**  **5141**  **3028**  **5385**  **1501**  **7354** |  | **61645**  **29763**  **81260**  **23128**  **60947**  **42123**  **59461**  **71550**  **11504**  **53952**  **87566**  **73315**  **12778**  **53357**  **120010**  **51031**  **25555**  **42320**  **53518**  **74145**  **45589**  **26205**  **47173**  **12791**  **85309** |  | **4484**  **4525**  **4441**  **4433**  **4445**  **4447**  **4556**  **4497**  **4490**  **4491**  **4493**  **4482**  **4502**  **4499**  **4486**  **4487**  **4462**  **4508**  **4469**  **4446**  **4442**  **4424**  **4471**  **4435**  **4498** |  |
| **2** | **28**  **29**  **30**  **31**  **32**  **33**  **34**  **35**  **36**  **37**  **38** | **7/5/05**  **7/5/05**  **7/5/05**  **7/5/05**  **7/5/05**  **7/5/05**  **7/5/05**  **7/5/05**  **7/5/05**  **7/15/05**  **7/15/05** | **CD3B1_2M16**  **CD3B1_2M25**  **CD3B1_2M33**  **CD3B1_2M41**  **CD3B1_2M8**  **CD3B1_2M17**  **CD3B1_2M26**  **CD3B1_2M34**  **CD3B1_2M42**  **CD003B1_2M4**  **CD003B1_2M27** | **D3S3623**  **D3S3623**  **D3S3623**  **D3S3623**  **D3S3623**  **D3S3623**  **D3S3623**  **D3S3623**  **D3S3623**  **D3S3623**  **D3S3623** | **219**  **219**  **219**  **219**  **219**  **219**  **219**  **219**  **219**  **219**  **219** |  | **218.5**  **218.5**  **218.5**  **218.4**  **218.6**  **218.5**  **218.5**  **218.6**  **218.6**  **218.8**  **218.8** |  | **3661**  **946**  **2824**  **1154**  **7965**  **7733**  **4952**  **6989**  **2741**  **1341**  **483** |  | **32296**  **8170**  **24664**  **9831**  **104723**  **83173**  **43289**  **62182**  **23428**  **12425**  **4446** |  | **4488**  **4479**  **4520**  **4468**  **4499**  **4488**  **4483**  **4526**  **4480**  **4737**  **4738** |  |
| **1** | **1**  **2**  **3**  **4**  **5**  **6**  **7**  **8**  **9**  **10**  **11**  **12**  **13** | **7/5/05**  **7/5/05**  **7/5/05**  **7/5/05**  **7/5/05**  **7/5/05**  **7/5/05**  **7/5/05**  **7/5/05**  **7/5/05**  **7/5/05**  **7/5/05**  **7/5/05** | **CD3B1_2M9**  **CD3B1_2M19**  **CD3B1_2M35**  **CD3B1_2M2**  **CD3B1_2M10**  **CD3B1_2M20**  **CD3B1_2M28**  **CD3B1_2M36**  **CD3B1_2M3**  **CD3B1_2M11**  **CD3B1_2M21**  **CD3B1_2M37**  **CD3B1_2M12** | **D5S346**  **D5S346**  **D5S346**  **D5S346**  **D5S346**  **D5S346**  **D5S346**  **D5S346**  **D5S346**  **D5S346**  **D5S346**  **D5S346**  **D5S346** | **92**  **92**  **92**  **92**  **92**  **92**  **92**  **92**  **92**  **92**  **92**  **92**  **92** | **105**  **105**  **105**  **105**  **105**  **105**  **105**  **105**  **105**  **105**  **105**  **105**  **105** | **91.7**  **91.6**  **91.6**  **91.6**  **91.6**  **91.6**  **91.6**  **91.6**  **91.7**  **91.6**  **91.5**  **91.6**  **91.6** | **104.7**  **104.6**  **104.6**  **104.7**  **104.7**  **104.6**  **104.6**  **104.6**  **104.7**  **104.6**  **104.6**  **104.6**  **104.7** | **5790**  **2624**  **3846**  **1922**  **4373**  **1284**  **3630**  **2850**  **3517**  **3604**  **192**  **2495**  **5622** | **1816**  **1087**  **1633**  **993**  **2116**  **884**  **1630**  **1058**  **1483**  **1825**  **198**  **1432**  **2498** | **47836**  **22104**  **32227**  **16403**  **36065**  **10576**  **29452**  **23561**  **30143**  **30658**  **1722**  **20807**  **47279** | **14559**  **8443**  **12785**  **7833**  **16623**  **6706**  **12553**  **8253**  **12056**  **14653**  **1516**  **11505**  **19942** | **2912**  **2905**  **2915**  **2940**  **2884**  **2878**  **2887**  **2889**  **2963**  **2927**  **2921**  **2924**  **2919** | **3083**  **3076**  **3086**  **3112**  **3054**  **3047**  **3056**  **3059**  **3136**  **3098**  **3093**  **3095**  **3091** |

| **Day** | **Count** | **Run Date** | **Sample Name** | **Marker** | **Allele 1** | **Allele 2** | **Size 1** | **Size 2** | **Height 1** | **Height 2** | **Peak Area 1** | **Peak Area 2** | **Data Point 1** | **Data Point 2** |
| --- | --- | --- | --- | --- | --- | --- | --- | --- | --- | --- | --- | --- | --- | --- |
|  | **14**  **15**  **16**  **17**  **18**  **19**  **20**  **21**  **22**  **23**  **24**  **25** | **7/5/05**  **7/5/05**  **7/5/05**  **7/5/05**  **7/5/05**  **7/5/05**  **7/5/05**  **7/5/05**  **7/5/05**  **7/5/05**  **7/5/05**  **7/5/05** | **CD3B1_2M22**  **CD3B1_2M30**  **CD3B1_2M38**  **CD3B1_2M5**  **CD3B1_2M13**  **CD3B1_2M23**  **CD3B1_2M31**  **CD3B1_2M39**  **CD3B1_2M6**  **CD3B1_2M14**  **CD3B1_2M24**  **CD3B1_2M32** | **D5S346**  **D5S346**  **D5S346**  **D5S346**  **D5S346**  **D5S346**  **D5S346**  **D5S346**  **D5S346**  **D5S346**  **D5S346**  **D5S346** | **92**  **92**  **92**  **92**  **92**  **92**  **92**  **92**  **92**  **92**  **92**  **92** | **105**  **105**  **105**  **105**  **105**  **105**  **105**  **105**  **105**  **105**  **105**  **105** | **91.7**  **91.6**  **91.7**  **91.7**  **91.6**  **91.6**  **91.6**  **91.6**  **91.6**  **91.5**  **91.6**  **91.6** | **104.6**  **104.7**  **104.7**  **104.7**  **104.7**  **104.6**  **104.7**  **104.7**  **104.6**  **104.6**  **104.6**  **104.6** | **3449**  **2208**  **2053**  **6727**  **2612**  **1328**  **2336**  **2644**  **4282**  **3700**  **1944**  **2675** | **1973**  **1180**  **995**  **3340**  **1317**  **707**  **1159**  **1159**  **2087**  **1744**  **1182**  **1159** | **28585**  **18172**  **17582**  **56840**  **21684**  **11470**  **19652**  **22528**  **35363**  **30847**  **16166**  **21938** | **15702**  **9583**  **7908**  **26513**  **10547**  **5689**  **9251**  **9224**  **16319**  **13723**  **9156**  **8944** | **2912**  **2928**  **2927**  **2915**  **2915**  **2899**  **2933**  **2905**  **2887**  **2882**  **2873**  **2906** | **3082**  **3100**  **3098**  **3086**  **3087**  **3070**  **3105**  **3076**  **3057**  **3052**  **3042**  **3076** |
| **2** | **26**  **27**  **28**  **29**  **30**  **31**  **32**  **33**  **34**  **35**  **36**  **37**  **38** | **7/5/05**  **7/5/05**  **7/5/05**  **7/5/05**  **7/5/05**  **7/5/05**  **7/5/05**  **7/5/05**  **7/5/05**  **7/5/05**  **7/5/05**  **7/15/05**  **7/15/05** | **CD3B1_2M40**  **CD3B1_2M7**  **CD3B1_2M16**  **CD3B1_2M25**  **CD3B1_2M33**  **CD3B1_2M41**  **CD3B1_2M8**  **CD3B1_2M17**  **CD3B1_2M26**  **CD3B1_2M34**  **CD3B1_2M42**  **CD003B1_2M4**  **CD003B1_2M27** | **D5S346**  **D5S346**  **D5S346**  **D5S346**  **D5S346**  **D5S346**  **D5S346**  **D5S346**  **D5S346**  **D5S346**  **D5S346**  **D5S346**  **D5S346** | **92**  **92**  **92**  **92**  **92**  **92**  **92**  **92**  **92**  **92**  **92**  **92**  **92** | **105**  **105**  **105**  **105**  **105**  **105**  **105**  **105**  **105**  **105**  **105**  **105**  **105** | **91.6**  **91.6**  **91.7**  **91.6**  **91.6**  **91.6**  **91.6**  **91.6**  **91.6**  **91.6**  **91.6**  **91.8**  **91.9** | **104.6**  **104.6**  **104.6**  **104.6**  **104.7**  **104.6**  **104.6**  **104.6**  **104.6**  **104.7**  **104.6**  **104.8**  **104.8** | **757**  **3337**  **1835**  **459**  **1109**  **632**  **5403**  **4346**  **1838**  **3520**  **1477**  **797**  **404** | **243**  **1492**  **964**  **433**  **664**  **339**  **2755**  **2081**  **1038**  **1624**  **847**  **403**  **279** | **6039**  **29415**  **15465**  **4683**  **9506**  **5249**  **44971**  **35725**  **14961**  **29593**  **12025**  **6767**  **3488** | **1836**  **11728**  **7745**  **3682**  **5401**  **3147**  **21892**  **16426**  **7989**  **13028**  **6671**  **3136**  **2400** | **2881**  **2927**  **2919**  **2915**  **2946**  **2911**  **2922**  **2915**  **2914**  **2946**  **2914**  **3103**  **3111** | **3050**  **3098**  **3089**  **3086**  **3118**  **3081**  **3093**  **3086**  **3085**  **3118**  **3084**  **3280**  **3287** |
| **1** | **1**  **2**  **3**  **4**  **5**  **6**  **7**  **8**  **9**  **10**  **11**  **12**  **13**  **14**  **15**  **16**  **17**  **18**  **19**  **20** | **7/5/05**  **7/5/05**  **7/5/05**  **7/5/05**  **7/5/05**  **7/5/05**  **7/5/05**  **7/5/05**  **7/5/05**  **7/5/05**  **7/5/05**  **7/5/05**  **7/5/05**  **7/5/05**  **7/5/05**  **7/5/05**  **7/5/05**  **7/5/05**  **7/5/05**  **7/5/05** | **CD3B1_2M9**  **CD3B1_2M19**  **CD3B1_2M35**  **CD3B1_2M2**  **CD3B1_2M10**  **CD3B1_2M20**  **CD3B1_2M28**  **CD3B1_2M36**  **CD3B1_2M3**  **CD3B1_2M11**  **CD3B1_2M21**  **CD3B1_2M37**  **CD3B1_2M12**  **CD3B1_2M22**  **CD3B1_2M30**  **CD3B1_2M38**  **CD3B1_2M5**  **CD3B1_2M13**  **CD3B1_2M23**  **CD3B1_2M31** | **D6S262**  **D6S262**  **D6S262**  **D6S262**  **D6S262**  **D6S262**  **D6S262**  **D6S262**  **D6S262**  **D6S262**  **D6S262**  **D6S262**  **D6S262**  **D6S262**  **D6S262**  **D6S262**  **D6S262**  **D6S262**  **D6S262**  **D6S262** | **182**  **182**  **182**  **182**  **182**  **182**  **182**  **182**  **182**  **182**  **182**  **182**  **182**  **182**  **182**  **182**  **182**  **182**  **182**  **182** | **185**  **185**  **185**  **185**  **185**  **185**  **185**  **185**  **185**  **185**  **185**  **185**  **185**  **185**  **185**  **185**  **185**  **185**  **185**  **185** | **182.0**  **181.9**  **181.8**  **182.0**  **181.9**  **181.8**  **181.9**  **181.9**  **181.9**  **181.9**  **181.8**  **181.8**  **181.9**  **181.9**  **181.9**  **181.9**  **181.9**  **181.9**  **181.9**  **181.9** | **184.9**  **184.8**  **184.8**  **185.0**  **184.9**  **184.7**  **184.9**  **184.8**  **184.9**  **184.8**  **184.8**  **184.8**  **184.9**  **184.8**  **184.8**  **184.9**  **184.9**  **184.9**  **184.8**  **184.8** | **2832**  **1360**  **2319**  **1089**  **3547**  **976**  **2701**  **1657**  **1966**  **2575**  **297**  **1665**  **3699**  **2682**  **862**  **1322**  **5467**  **1590**  **831**  **1460** | **3323**  **2219**  **2766**  **1396**  **3849**  **909**  **3066**  **1702**  **2374**  **2798**  **528**  **1805**  **4111**  **3011**  **1287**  **1637**  **5803**  **1877**  **984**  **1862** | **26313**  **12068**  **20218**  **10351**  **31620**  **8372**  **23048**  **14288**  **18597**  **22856**  **2146**  **14636**  **32698**  **23599**  **6995**  **12235**  **49998**  **13971**  **6798**  **12870** | **26376**  **17242**  **21656**  **11569**  **29751**  **6652**  **23714**  **12872**  **20160**  **21769**  **3966**  **13988**  **32165**  **23300**  **10283**  **13061**  **49938**  **14736**  **7399**  **14449** | **4046**  **4037**  **4049**  **4085**  **4009**  **4002**  **4013**  **4015**  **4113**  **4062**  **4055**  **4057**  **4057**  **4046**  **4066**  **4063**  **4050**  **4051**  **4029**  **4072** | **4082**  **4073**  **4086**  **4122**  **4046**  **4038**  **4050**  **4051**  **4151**  **4099**  **4092**  **4094**  **4094**  **4083**  **4102**  **4100**  **4087**  **4088**  **4065**  **4108** |
|  | **21**  **22**  **23**  **24**  **25**  **26**  **27**  **28**  **29**  **30**  **31**  **32**  **33** | **7/5/05**  **7/5/05**  **7/5/05**  **7/5/05**  **7/5/05**  **7/5/05**  **7/5/05**  **7/5/05**  **7/5/05**  **7/5/05**  **7/5/05**  **7/5/05**  **7/5/05** | **CD3B1_2M39**  **CD3B1_2M6**  **CD3B1_2M14**  **CD3B1_2M24**  **CD3B1_2M32**  **CD3B1_2M40**  **CD3B1_2M7**  **CD3B1_2M16**  **CD3B1_2M25**  **CD3B1_2M33**  **CD3B1_2M41**  **CD3B1_2M8**  **CD3B1_2M17** | **D6S262**  **D6S262**  **D6S262**  **D6S262**  **D6S262**  **D6S262**  **D6S262**  **D6S262**  **D6S262**  **D6S262**  **D6S262**  **D6S262**  **D6S262** | **182**  **182**  **182**  **182**  **182**  **182**  **182**  **182**  **182**  **182**  **182**  **182**  **182** | **185**  **185**  **185**  **185**  **185**  **185**  **185**  **185**  **185**  **185**  **185**  **185**  **185** | **181.9**  **181.9**  **181.8**  **181.9**  **181.9**  **181.9**  **181.9**  **181.9**  **181.9**  **181.9**  **181.9**  **181.9**  **181.9** | **184.9**  **184.8**  **184.8**  **184.8**  **184.8**  **184.8**  **184.8**  **184.8**  **184.9**  **184.9**  **184.9**  **184.9**  **184.9** | **1837**  **3272**  **2152**  **926**  **1985**  **368**  **2647**  **1503**  **423**  **890**  **414**  **3660**  **3394** | **2053**  **3899**  **2439**  **1429**  **2510**  **191**  **3149**  **1487**  **319**  **921**  **477**  **4823**  **3240** | **14694**  **30806**  **17452**  **7277**  **16438**  **2546**  **25586**  **13139**  **3205**  **7142**  **3196**  **35468**  **29975** | **15308**  **31600**  **19025**  **10963**  **19119**  **1343**  **26239**  **11490**  **2274**  **7137**  **3368**  **40907**  **24938** | **4035**  **4014**  **4009**  **3994**  **4037**  **4004**  **4064**  **4053**  **4046**  **4084**  **4037**  **4061**  **4052** | **4072**  **4050**  **4046**  **4030**  **4073**  **4040**  **4100**  **4090**  **4083**  **4121**  **4074**  **4098**  **4089** |

| **Day** | **Count** | **Run Date** | **Sample Name** | **Marker** | **Allele 1** | **Allele 2** | **Size 1** | **Size 2** | **Height 1** | **Height 2** | **Peak Area 1** | **Peak Area 2** | **Data Point 1** | **Data Point 2** |
| --- | --- | --- | --- | --- | --- | --- | --- | --- | --- | --- | --- | --- | --- | --- |
| **2** | **34**  **35**  **36**  **37**  **38** | **7/5/05**  **7/5/05**  **7/5/05**  **7/15/05**  **7/15/05** | **CD3B1_2M26**  **CD3B1_2M34**  **CD3B1_2M42**  **CD003B1_2M4**  **CD003B1_2M27** | **D6S262**  **D6S262**  **D6S262**  **D6S262**  **D6S262** | **182**  **182**  **182**  **182**  **182** | **185**  **185**  **185**  **185**  **185** | **181.8**  **181.9**  **182.0**  **182.0**  **182.1** | **184.8**  **184.9**  **184.9**  **185.0**  **185.1** | **1292**  **2313**  **697**  **465**  **204** | **1266**  **2741**  **908**  **509**  **225** | **11279**  **20747**  **5690**  **4626**  **1666** | **9748**  **21716**  **6667**  **3920**  **1713** | **4048**  **4087**  **4046**  **4283**  **4287** | **4085**  **4125**  **4083**  **4322**  **4326** |
| **1** | **1**  **2**  **3**  **4**  **5**  **6**  **7**  **8**  **9**  **10** | **7/5/05**  **7/5/05**  **7/5/05**  **7/5/05**  **7/5/05**  **7/5/05**  **7/5/05**  **7/5/05**  **7/5/05**  **7/5/05** | **CD3B1_2M9**  **CD3B1_2M19**  **CD3B1_2M35**  **CD3B1_2M2**  **CD3B1_2M10**  **CD3B1_2M20**  **CD3B1_2M28**  **CD3B1_2M36**  **CD3B1_2M3**  **CD3B1_2M11** | **D7S481**  **D7S481**  **D7S481**  **D7S481**  **D7S481**  **D7S481**  **D7S481**  **D7S481**  **D7S481**  **D7S481** | **197**  **197**  **197**  **197**  **197**  **197**  **197**  **197**  **197**  **197** | **201**  **201**  **201**  **201**  **201**  **201**  **201**  **201**  **201**  **201** | **196.8**  **196.8**  **196.7**  **196.8**  **196.9**  **196.8**  **196.8**  **196.8**  **196.8**  **196.8** | **200.6**  **200.7**  **200.6**  **200.8**  **200.7**  **200.6**  **200.6**  **200.6**  **200.7**  **200.7** | **866**  **417**  **806**  **476**  **1150**  **378**  **715**  **599**  **920**  **917** | **738**  **273**  **586**  **368**  **982**  **332**  **617**  **411**  **728**  **673** | **7774**  **3712**  **6992**  **4503**  **10053**  **3314**  **6393**  **5361**  **9179**  **8331** | **6484**  **2351**  **4902**  **3403**  **8539**  **2844**  **5400**  **3518**  **6908**  **5904** | **4231**  **4222**  **4234**  **4272**  **4194**  **4186**  **4198**  **4199**  **4301**  **4248** | **4278**  **4270**  **4282**  **4321**  **4241**  **4233**  **4244**  **4246**  **4350**  **4296** |
| **2** | **11**  **12**  **13**  **14**  **15**  **16**  **17**  **18**  **19**  **20**  **21**  **22**  **23**  **24**  **25**  **26**  **27**  **28**  **29**  **30**  **31**  **32**  **33**  **34**  **35**  **36**  **37**  **38** | **7/5/05**  **7/5/05**  **7/5/05**  **7/5/05**  **7/5/05**  **7/5/05**  **7/5/05**  **7/5/05**  **7/5/05**  **7/5/05**  **7/5/05**  **7/5/05**  **7/5/05**  **7/5/05**  **7/5/05**  **7/5/05**  **7/5/05**  **7/5/05**  **7/5/05**  **7/5/05**  **7/5/05**  **7/5/05**  **7/5/05**  **7/5/05**  **7/5/05**  **7/5/05**  **7/15/05**  **7/15/05** | **CD3B1_2M21**  **CD3B1_2M37**  **CD3B1_2M12**  **CD3B1_2M22**  **CD3B1_2M30**  **CD3B1_2M38**  **CD3B1_2M5**  **CD3B1_2M13**  **CD3B1_2M23**  **CD3B1_2M31**  **CD3B1_2M39**  **CD3B1_2M6**  **CD3B1_2M14**  **CD3B1_2M24**  **CD3B1_2M32**  **CD3B1_2M40**  **CD3B1_2M7**  **CD3B1_2M16**  **CD3B1_2M25**  **CD3B1_2M33**  **CD3B1_2M41**  **CD3B1_2M8**  **CD3B1_2M17**  **CD3B1_2M26**  **CD3B1_2M34**  **CD3B1_2M42**  **CD003B1_2M4**  **CD003B1_2M27** | **D7S481**  **D7S481**  **D7S481**  **D7S481**  **D7S481**  **D7S481**  **D7S481**  **D7S481**  **D7S481**  **D7S481**  **D7S481**  **D7S481**  **D7S481**  **D7S481**  **D7S481**  **D7S481**  **D7S481**  **D7S481**  **D7S481**  **D7S481**  **D7S481**  **D7S481**  **D7S481**  **D7S481**  **D7S481**  **D7S481**  **D7S481**  **D7S481** | **197**  **197**  **197**  **197**  **197**  **197**  **197**  **197**  **197**  **197**  **197**  **197**  **197**  **197**  **197**  **197**  **197**  **197**  **197**  **197**  **197**  **197**  **197**  **197**  **197**  **197**  **197**  **197** | **201**  **201**  **201**  **201**  **201**  **201**  **201**  **201**  **201**  **201**  **201**  **201**  **201**  **201**  **201**  **201**  **201**  **201**  **201**  **201**  **201**  **201**  **201**  **201**  **201**  **201**  **201**  **201** | **196.8**  **196.8**  **196.8**  **196.8**  **196.8**  **196.8**  **196.8**  **196.7**  **196.8**  **196.8**  **196.8**  **196.8**  **196.8**  **196.8**  **196.8**  **196.8**  **196.9**  **196.7**  **196.8**  **196.8**  **196.8**  **196.7**  **196.8**  **196.7**  **196.8**  **196.9**  **196.9**  **197.0** | **200.6**  **200.6**  **200.7**  **200.7**  **200.6**  **200.6**  **200.7**  **200.6**  **200.6**  **200.6**  **200.6**  **200.7**  **200.6**  **200.5**  **200.6**  **200.6**  **200.7**  **200.6**  **200.6**  **200.7**  **200.6**  **200.6**  **200.7**  **200.5**  **200.7**  **200.7**  **200.9**  **200.9** | **398**  **685**  **1279**  **928**  **360**  **682**  **2242**  **637**  **549**  **557**  **632**  **1607**  **657**  **464**  **761**  **209**  **1725**  **484**  **283**  **388**  **306**  **1932**  **1357**  **726**  **946**  **555**  **344**  **155** | **179**  **533**  **1000**  **689**  **259**  **526**  **1780**  **455**  **431**  **442**  **489**  **1082**  **546**  **435**  **555**  **227**  **1075**  **381**  **253**  **308**  **173**  **1333**  **1070**  **393**  **705**  **472**  **368**  **107** | **3567**  **5994**  **11705**  **8393**  **3133**  **6300**  **21633**  **5866**  **4927**  **4901**  **5528**  **15256**  **6021**  **4200**  **6762**  **1656**  **16758**  **4183**  **2525**  **3310**  **2678**  **18836**  **12062**  **6813**  **8484**  **4693**  **3186**  **1385** | **1499**  **4597**  **8936**  **6343**  **2346**  **4713**  **16576**  **4091**  **3921**  **3861**  **4072**  **10057**  **4986**  **3908**  **4800**  **2187**  **9825**  **3303**  **2106**  **2696**  **1519**  **12281**  **9392**  **3464**  **6204**  **4104**  **3596**  **1002** | **4241**  **4242**  **4243**  **4232**  **4252**  **4249**  **4236**  **4236**  **4213**  **4258**  **4220**  **4198**  **4193**  **4178**  **4222**  **4188**  **4250**  **4238**  **4231**  **4270**  **4221**  **4247**  **4238**  **4233**  **4274**  **4231**  **4476**  **4479** | **4288**  **4289**  **4291**  **4280**  **4299**  **4296**  **4284**  **4284**  **4260**  **4305**  **4267**  **4246**  **4240**  **4224**  **4269**  **4234**  **4297**  **4286**  **4278**  **4318**  **4268**  **4295**  **4286**  **4280**  **4322**  **4278**  **4526**  **4528** |
| **1** | **1**  **2**  **3**  **4**  **5** | **11/14/05**  **11/14/05**  **11/14/05**  **11/14/05**  **11/14/05** | **CD005B1M1**  **CD005B1M3**  **CD005B1M4**  **CD005B1M5**  **CD005B1M6** | **BAT25**  **BAT25**  **BAT25**  **BAT25**  **BAT25** | **127**  **127**  **127**  **127**  **127** |  | **126.7**  **126.8**  **126.8**  **126.8**  **126.7** |  | **4464**  **4908**  **5652**  **5135**  **1901** |  | **36399**  **39330**  **45929**  **40184**  **14887** |  | **3613**  **3571**  **3623**  **3641**  **3641** |  |
| **1** | **1**  **2**  **3**  **4**  **5** | **11/14/05**  **11/14/05**  **11/14/05**  **11/14/05**  **11/14/05** | **CD005B1M1**  **CD005B1M3**  **CD005B1M4**  **CD005B1M5**  **CD005B1M6** | **BAT26**  **BAT26**  **BAT26**  **BAT26**  **BAT26** | **122**  **122**  **122**  **122**  **122** | **123** | **121.6**  **121.7**  **121.7**  **121.7**  **121.6** | **122.6** | **1142**  **1267**  **1473**  **1053**  **432** | **1038** | **11109**  **11464**  **18149**  **10234**  **4034** | **9114** | **3546**  **3505**  **3555**  **3572**  **3573** | **3584** |
| **1** | **1**  **2**  **3**  **4** | **11/14/05**  **11/14/05**  **11/14/05**  **11/14/05** | **CD005B1M1**  **CD005B1M3**  **CD005B1M4**  **CD005B1M5** | **D3S3623**  **D3S3623**  **D3S3623**  **D3S3623** | **219**  **219**  **219**  **219** | **221**  **221**  **221**  **221** | **218.5**  **218.5**  **218.5**  **218.6** | **220.6**  **220.6**  **220.7**  **220.7** | **5206**  **5339**  **6127**  **5612** | **3272**  **3502**  **3874**  **3553** | **49122**  **47888**  **59701**  **51246** | **29103**  **29214**  **34803**  **30574** | **4753**  **4699**  **4765**  **4800** | **4777**  **4723**  **4790**  **4825** |

| **Day** | **Count** | **Run Date** | **Sample Name** | **Marker** | **Allele 1** | **Allele 2** | **Size 1** | **Size 2** | **Height 1** | **Height 2** | **Peak Area 1** | **Peak Area 2** | **Data Point 1** | **Data Point 2** |
| --- | --- | --- | --- | --- | --- | --- | --- | --- | --- | --- | --- | --- | --- | --- |
|  | **5** | **11/14/05** | **CD005B1M6** | **D3S3623** | **219** | **221** | **218.7** | **220.7** | **2270** | **1222** | **21939** | **10710** | **4799** | **4823** |
| **1** | **1**  **2**  **3**  **4**  **5** | **11/14/05**  **11/14/05**  **11/14/05**  **11/14/05**  **11/14/05** | **CD005B1M1**  **CD005B1M3**  **CD005B1M4**  **CD005B1M5**  **CD005B1M6** | **D5S346**  **D5S346**  **D5S346**  **D5S346**  **D5S346** | **89**  **89**  **89**  **89**  **89** | **92**  **92**  **92**  **92**  **92** | **89.4**  **89.4**  **89.4**  **89.5**  **89.4** | **91.6**  **91.6**  **91.6**  **91.7**  **91.6** | **4847**  **4714**  **5050**  **4676**  **1491** | **3276**  **3305**  **3483**  **3332**  **1390** | **38404**  **35188**  **39547**  **36415**  **11503** | **23755**  **22887**  **25254**  **23973**  **10504** | **3107**  **3069**  **3114**  **3127**  **3128** | **3136**  **3099**  **3144**  **3157**  **3158** |
| **1** | **1**  **2**  **3**  **4**  **5** | **11/14/05**  **11/14/05**  **11/14/05**  **11/14/05**  **11/14/05** | **CD005B1M1**  **CD005B1M3**  **CD005B1M4**  **CD005B1M5**  **CD005B1M6** | **D6S262**  **D6S262**  **D6S262**  **D6S262**  **D6S262** | **170**  **170**  **170**  **170**  **170** | **172**  **172**  **172**  **172**  **172** | **170.2**  **170.3**  **170.3**  **170.3**  **170.3** | **172.2**  **172.1**  **172.2**  **172.2**  **172.2** | **5580**  **5627**  **5879**  **5238**  **1728** | **3893**  **4169**  **4066**  **3789**  **1289** | **53698**  **53869**  **54810**  **46654**  **15605** | **30679**  **31551**  **33604**  **29988**  **10515** | **4155**  **4109**  **4166**  **4192**  **4191** | **4181**  **4132**  **4191**  **4217**  **4217** |
| **1** | **1**  **2**  **3**  **4**  **5** | **11/14/05**  **11/14/05**  **11/14/05**  **11/14/05**  **11/14/05** | **CD005B1M1**  **CD005B1M3**  **CD005B1M4**  **CD005B1M5**  **CD005B1M6** | **D7S481**  **D7S481**  **D7S481**  **D7S481**  **D7S481** | **187**  **187**  **187**  **187**  **187** | **203**  **203**  **203**  **203**  **203** | **187.1**  **187.1**  **187.2**  **187.2**  **187.2** | **202.8**  **202.6**  **202.7**  **202.8**  **202.7** | **1478**  **1548**  **1945**  **1427**  **884** | **1054**  **1125**  **1289**  **1007**  **497** | **17154**  **17410**  **23342**  **16212**  **10298** | **11680**  **12220**  **14579**  **10915**  **5360** | **4373**  **4323**  **4384**  **4413**  **4412** | **4570**  **4516**  **4581**  **4613**  **4611** |
| **1** | **1**  **2**  **3**  **4**  **5**  **6**  **7**  **8**  **9**  **10**  **11**  **12**  **13**  **14**  **15**  **16**  **17**  **18**  **19**  **20**  **21**  **22**  **23**  **24**  **25**  **26**  **27**  **28**  **29**  **30**  **31**  **32**  **33**  **34**  **35**  **36**  **37**  **38**  **39**  **40**  **41** | **7/6/05**  **7/6/05**  **7/6/05**  **7/6/05**  **7/6/05**  **7/6/05**  **7/6/05**  **7/6/05**  **7/6/05**  **7/6/05**  **7/6/05**  **7/6/05**  **7/6/05**  **7/6/05**  **7/6/05**  **7/6/05**  **7/6/05**  **7/6/05**  **7/6/05**  **7/6/05**  **7/6/05**  **7/6/05**  **7/6/05**  **7/6/05**  **7/6/05**  **7/6/05**  **7/6/05**  **7/6/05**  **7/6/05**  **7/6/05**  **7/6/05**  **7/6/05**  **7/6/05**  **7/6/05**  **7/6/05**  **7/6/05**  **7/6/05**  **7/6/05**  **7/6/05**  **7/6/05**  **7/6/05** | **CD006B1_M2**  **CD006B1_M15**  **CD006B1_M27**  **CD006B1_M37**  **CD006B1_M48**  **CD006B1_M73**  **CD006B1_M3**  **CD006B1_M16**  **CD006B1_M28**  **CD006B1_M39**  **CD006B1_M51**  **CD006B1_M75**  **CD006B1_M4**  **CD006B1_M19**  **CD006B1_M29**  **CD006B1_M40**  **CD006B1_M52**  **CD006B1_M82**  **CD006B1_M5**  **CD006B1_M20**  **CD006B1_M31**  **CD006B1_M41**  **CD006B1_M53**  **CD006B1_M6**  **CD006B1_M23**  **CD006B1_M32**  **CD006B1_M54**  **CD006B1_M8**  **CD006B1_M24**  **CD006B1_M33**  **CD006B1_M45**  **CD006B1_M55**  **CD006B1_M9**  **CD006B1_M25**  **CD006B1_M35**  **CD006B1_M46**  **CD006B1_M65**  **CD006B1_M26**  **CD006B1_M36**  **CD006B1_M47**  **CD006B1_M67** | **BAT25**  **BAT25**  **BAT25**  **BAT25**  **BAT25**  **BAT25**  **BAT25**  **BAT25**  **BAT25**  **BAT25**  **BAT25**  **BAT25**  **BAT25**  **BAT25**  **BAT25**  **BAT25**  **BAT25**  **BAT25**  **BAT25**  **BAT25**  **BAT25**  **BAT25**  **BAT25**  **BAT25**  **BAT25**  **BAT25**  **BAT25**  **BAT25**  **BAT25**  **BAT25**  **BAT25**  **BAT25**  **BAT25**  **BAT25**  **BAT25**  **BAT25**  **BAT25**  **BAT25**  **BAT25**  **BAT25**  **BAT25** | **127**  **127**  **127**  **127**  **126**  **127**  **127**  **127**  **127**  **127**  **126**  **127**  **127**  **127**  **127**  **127**  **126**  **126**  **127**  **127**  **127**  **127**  **127**  **127**  **127**  **127**  **127**  **127**  **127**  **127**  **127**  **127**  **127**  **127**  **127**  **127**  **126**  **127**  **126**  **127**  **127** | **127**  **127**  **127**  **127**  **127**  **127** | **126.8**  **126.7**  **126.7**  **126.7**  **125.7**  **126.7**  **126.9**  **126.7**  **126.7**  **126.7**  **125.7**  **126.7**  **126.8**  **126.7**  **126.7**  **126.7**  **125.7**  **125.7**  **126.8**  **126.8**  **126.7**  **126.8**  **126.8**  **126.7**  **126.7**  **126.8**  **126.8**  **126.7**  **126.7**  **126.7**  **126.8**  **126.7**  **126.7**  **126.7**  **126.7**  **126.7**  **125.7**  **126.7**  **125.7**  **126.7**  **126.8** | **126.7**  **126.7**  **126.7**  **126.7**  **126.7**  **126.7** | **5074**  **4773**  **5760**  **4665**  **3430**  **2198**  **3370**  **4315**  **1632**  **4453**  **2919**  **1455**  **4001**  **4055**  **1865**  **2766**  **1177**  **1410**  **1906**  **5064**  **3521**  **4763**  **4402**  **4019**  **4219**  **3699**  **2331**  **3512**  **3433**  **2995**  **3358**  **3096**  **3810**  **4586**  **3550**  **3582**  **4018**  **5326**  **3375**  **3361**  **4823** | **3451**  **2791**  **1155**  **1473**  **4191**  **3465** | **39843**  **36909**  **43960**  **36540**  **26520**  **16507**  **25994**  **32285**  **12946**  **34223**  **22239**  **11367**  **32409**  **31561**  **14564**  **21896**  **9409**  **10808**  **15664**  **39598**  **27714**  **36838**  **33656**  **30955**  **33601**  **28597**  **17732**  **27101**  **26915**  **22859**  **25521**  **23883**  **30145**  **35535**  **27843**  **27405**  **30549**  **41660**  **25166**  **25704**  **35875** | **26576**  **20267**  **9473**  **11008**  **32410**  **25960** | **3458**  **3370**  **3336**  **3355**  **3369**  **3400**  **3433**  **3345**  **3310**  **3332**  **3345**  **3377**  **3448**  **3364**  **3328**  **3349**  **3361**  **3356**  **3462**  **3373**  **3340**  **3359**  **3383**  **3368**  **3352**  **3369**  **3397**  **3345**  **3327**  **3345**  **3354**  **3374**  **3364**  **3347**  **3363**  **3375**  **3376**  **3355**  **3360**  **3386**  **3399** | **3382**  **3358**  **3374**  **3369**  **3389**  **3373** |

| **Day** | **Count** | **Run Date** | **Sample Name** | **Marker** | **Allele 1** | **Allele 2** | **Size 1** | **Size 2** | **Height 1** | **Height 2** | **Peak Area 1** | **Peak Area 2** | **Data Point 1** | **Data Point 2** |
| --- | --- | --- | --- | --- | --- | --- | --- | --- | --- | --- | --- | --- | --- | --- |
| **2** | **42** | **7/7/05** | **CD006B1_M43-REDO** | **BAT25** | **127** |  | **126.7** |  | **2189** |  | **17457** |  | **3368** |  |
| **1** | **1**  **2**  **3**  **4**  **5**  **6**  **7**  **8**  **9**  **10** | **7/6/05**  **7/6/05**  **7/6/05**  **7/6/05**  **7/6/05**  **7/6/05**  **7/6/05**  **7/6/05**  **7/6/05**  **7/6/05** | **CD006B1_M2**  **CD006B1_M15**  **CD006B1_M27**  **CD006B1_M37**  **CD006B1_M48**  **CD006B1_M73**  **CD006B1_M3**  **CD006B1_M16**  **CD006B1_M28**  **CD006B1_M39** | **BAT26**  **BAT26**  **BAT26**  **BAT26**  **BAT26**  **BAT26**  **BAT26**  **BAT26**  **BAT26**  **BAT26** | **122**  **121**  **122**  **122**  **122**  **122**  **122**  **122**  **122**  **122** | **122** | **121.7**  **120.8**  **121.6**  **121.6**  **121.6**  **121.6**  **121.8**  **121.6**  **121.6**  **121.6** | **121.7** | **1920**  **1250**  **1621**  **1324**  **1117**  **638**  **1430**  **1317**  **682**  **1443** | **1296** | **17595**  **11544**  **15660**  **12369**  **10353**  **5323**  **12655**  **12805**  **6414**  **13327** | **11169** | **3392**  **3294**  **3271**  **3290**  **3316**  **3334**  **3367**  **3280**  **3246**  **3267** | **3305** |
|  | **11**  **12**  **13**  **14**  **15**  **16**  **17**  **18**  **19**  **20**  **21** | **7/6/05**  **7/6/05**  **7/6/05**  **7/6/05**  **7/6/05**  **7/6/05**  **7/6/05**  **7/6/05**  **7/6/05**  **7/6/05**  **7/6/05** | **CD006B1_M51**  **CD006B1_M75**  **CD006B1_M4**  **CD006B1_M19**  **CD006B1_M29**  **CD006B1_M40**  **CD006B1_M52**  **CD006B1_M82**  **CD006B1_M5**  **CD006B1_M20**  **CD006B1_M31** | **BAT26**  **BAT26**  **BAT26**  **BAT26**  **BAT26**  **BAT26**  **BAT26**  **BAT26**  **BAT26**  **BAT26**  **BAT26** | **122**  **122**  **122**  **122**  **122**  **122**  **122**  **122**  **122**  **121**  **122** | **122** | **121.6**  **121.6**  **121.8**  **121.6**  **121.7**  **121.6**  **121.6**  **121.6**  **121.8**  **120.7**  **121.7** | **121.6** | **913**  **597**  **1688**  **1371**  **791**  **1021**  **456**  **495**  **587**  **1391**  **1145** | **1463** | **8127**  **4846**  **15142**  **13260**  **7789**  **9941**  **4177**  **4135**  **5146**  **12582**  **10585** | **12620** | **3293**  **3312**  **3382**  **3299**  **3265**  **3284**  **3309**  **3304**  **3396**  **3295**  **3276** | **3307** |
| **2** | **22**  **23**  **24**  **25**  **26**  **27**  **28**  **29**  **30**  **31**  **32**  **33**  **34**  **35**  **36**  **37**  **38**  **39**  **40**  **41**  **42** | **7/6/05**  **7/6/05**  **7/6/05**  **7/6/05**  **7/6/05**  **7/6/05**  **7/6/05**  **7/6/05**  **7/6/05**  **7/6/05**  **7/6/05**  **7/6/05**  **7/6/05**  **7/6/05**  **7/6/05**  **7/6/05**  **7/6/05**  **7/6/05**  **7/6/05**  **7/6/05**  **7/7/05** | **CD006B1_M41**  **CD006B1_M53**  **CD006B1_M6**  **CD006B1_M23**  **CD006B1_M32**  **CD006B1_M54**  **CD006B1_M8**  **CD006B1_M24**  **CD006B1_M33**  **CD006B1_M45**  **CD006B1_M55**  **CD006B1_M9**  **CD006B1_M25**  **CD006B1_M35**  **CD006B1_M46**  **CD006B1_M65**  **CD006B1_M26**  **CD006B1_M36**  **CD006B1_M47**  **CD006B1_M67**  **CD006B1_M43-REDO** | **BAT26**  **BAT26**  **BAT26**  **BAT26**  **BAT26**  **BAT26**  **BAT26**  **BAT26**  **BAT26**  **BAT26**  **BAT26**  **BAT26**  **BAT26**  **BAT26**  **BAT26**  **BAT26**  **BAT26**  **BAT26**  **BAT26**  **BAT26**  **BAT26** | **122**  **122**  **122**  **122**  **122**  **122**  **122**  **122**  **122**  **122**  **122**  **122**  **122**  **122**  **122**  **122**  **122**  **122**  **122**  **122**  **122** |  | **121.7**  **121.7**  **121.7**  **121.7**  **121.7**  **121.7**  **121.7**  **121.6**  **121.6**  **121.7**  **121.6**  **121.7**  **121.6**  **121.6**  **121.6**  **121.7**  **121.7**  **121.6**  **121.6**  **121.7**  **121.7** |  | **1550**  **1444**  **1282**  **1211**  **1230**  **814**  **1326**  **1226**  **1126**  **1223**  **1062**  **1349**  **1620**  **1386**  **1272**  **1337**  **1717**  **1275**  **1128**  **1185**  **665** |  | **14269**  **13415**  **11865**  **11722**  **11494**  **7497**  **12725**  **11899**  **10654**  **11687**  **9905**  **12953**  **15792**  **13491**  **12004**  **12982**  **15616**  **11339**  **9666**  **11066**  **5628** |  | **3294**  **3318**  **3303**  **3287**  **3304**  **3331**  **3280**  **3262**  **3280**  **3289**  **3309**  **3299**  **3282**  **3298**  **3310**  **3324**  **3291**  **3308**  **3320**  **3333**  **3303** |  |
| **1** | **1**  **2**  **3**  **4**  **5**  **6**  **7**  **8**  **9**  **10**  **11**  **12**  **13**  **14**  **15** | **7/6/05**  **7/6/05**  **7/6/05**  **7/6/05**  **7/6/05**  **7/6/05**  **7/6/05**  **7/6/05**  **7/6/05**  **7/6/05**  **7/6/05**  **7/6/05**  **7/6/05**  **7/6/05**  **7/6/05** | **CD006B1_M2**  **CD006B1_M15**  **CD006B1_M27**  **CD006B1_M37**  **CD006B1_M48**  **CD006B1_M73**  **CD006B1_M3**  **CD006B1_M16**  **CD006B1_M28**  **CD006B1_M39**  **CD006B1_M51**  **CD006B1_M75**  **CD006B1_M4**  **CD006B1_M19**  **CD006B1_M29** | **D3S3623**  **D3S3623**  **D3S3623**  **D3S3623**  **D3S3623**  **D3S3623**  **D3S3623**  **D3S3623**  **D3S3623**  **D3S3623**  **D3S3623**  **D3S3623**  **D3S3623**  **D3S3623**  **D3S3623** | **219**  **219**  **219**  **219**  **219**  **219**  **219**  **219**  **219**  **219**  **219**  **219**  **219**  **219**  **219** |  | **218.8**  **218.6**  **218.4**  **218.5**  **218.6**  **218.6**  **218.7**  **218.5**  **218.5**  **218.4**  **218.5**  **218.6**  **218.7**  **218.6**  **218.4** |  | **6929**  **7553**  **7716**  **5503**  **5322**  **3663**  **4823**  **6216**  **2586**  **7235**  **4279**  **2520**  **6042**  **6853**  **3386** |  | **68467**  **70369**  **77139**  **49573**  **48360**  **32050**  **44230**  **55792**  **22979**  **64896**  **38406**  **22222**  **55116**  **61369**  **30308** |  | **4586**  **4476**  **4435**  **4459**  **4493**  **4513**  **4555**  **4445**  **4401**  **4430**  **4462**  **4486**  **4571**  **4462**  **4420** |  |

| **Day** | **Count** | **Run Date** | **Sample Name** | **Marker** | **Allele 1** | **Allele 2** | **Size 1** | **Size 2** | **Height 1** | **Height 2** | **Peak Area 1** | **Peak Area 2** | **Data Point 1** | **Data Point 2** |
| --- | --- | --- | --- | --- | --- | --- | --- | --- | --- | --- | --- | --- | --- | --- |
| **2** | **16**  **17**  **18**  **19**  **20**  **21**  **22**  **23**  **24**  **25**  **26**  **27**  **28**  **29**  **30**  **31**  **32**  **33**  **34**  **35**  **36**  **37**  **38**  **39**  **40**  **41**  **42** | **7/6/05**  **7/6/05**  **7/6/05**  **7/6/05**  **7/6/05**  **7/6/05**  **7/6/05**  **7/6/05**  **7/6/05**  **7/6/05**  **7/6/05**  **7/6/05**  **7/6/05**  **7/6/05**  **7/6/05**  **7/6/05**  **7/6/05**  **7/6/05**  **7/6/05**  **7/6/05**  **7/6/05**  **7/6/05**  **7/6/05**  **7/6/05**  **7/6/05**  **7/6/05**  **7/7/05** | **CD006B1_M40**  **CD006B1_M52**  **CD006B1_M82**  **CD006B1_M5**  **CD006B1_M20**  **CD006B1_M31**  **CD006B1_M41**  **CD006B1_M53**  **CD006B1_M6**  **CD006B1_M23**  **CD006B1_M32**  **CD006B1_M54**  **CD006B1_M8**  **CD006B1_M24**  **CD006B1_M33**  **CD006B1_M45**  **CD006B1_M55**  **CD006B1_M9**  **CD006B1_M25* CD006B1_M35**  **CD006B1_M46**  **CD006B1_M65**  **CD006B1_M26**  **CD006B1_M36**  **CD006B1_M47**  **CD006B1_M67**  **CD006B1_M43-REDO** | **D3S3623**  **D3S3623**  **D3S3623**  **D3S3623**  **D3S3623**  **D3S3623**  **D3S3623**  **D3S3623**  **D3S3623**  **D3S3623**  **D3S3623**  **D3S3623**  **D3S3623**  **D3S3623**  **D3S3623**  **D3S3623**  **D3S3623**  **D3S3623**  **D3S3623* D3S3623**  **D3S3623**  **D3S3623**  **D3S3623**  **D3S3623**  **D3S3623**  **D3S3623**  **D3S3623** | **219**  **219**  **219**  **219**  **219**  **219**  **219**  **219**  **219**  **219**  **219**  **219**  **219**  **219**  **219**  **219**  **219**  **219**  **219**  **219**  **219**  **219**  **219**  **219**  **219**  **219**  **219** | **223** | **218.5**  **218.6**  **218.5**  **218.8**  **218.6**  **218.5**  **218.6**  **218.6**  **218.6**  **218.5**  **218.6**  **218.5**  **218.5**  **218.6**  **218.5**  **218.6**  **218.6**  **218.6**  **218.5**  **218.5**  **218.5**  **218.6**  **218.6**  **218.5**  **218.6**  **218.6**  **218.5** | **222.7** | **4994**  **2351**  **2281**  **2912**  **7697**  **7242**  **7517**  **7537**  **6935**  **7261**  **6760**  **4879**  **5283**  **6169**  **4877**  **5944**  **5486**  **6231**  **6107**  **5506**  **5934**  **6652**  **7522**  **5356**  **5738**  **6414**  **3867** | **1412** | **45906**  **20135**  **20118**  **27084**  **79401**  **63971**  **69225**  **70897**  **62061**  **64610**  **60977**  **44590**  **46965**  **54733**  **43677**  **53145**  **48995**  **56090**  **54407**  **49575**  **53163**  **60507**  **71306**  **47327**  **51323**  **57625**  **34557** | **11319** | **4446**  **4477**  **4464**  **4593**  **4479**  **4440**  **4462**  **4492**  **4473**  **4451**  **4472**  **4509**  **4446**  **4420**  **4442**  **4456**  **4482**  **4463**  **4440**  **4459**  **4476**  **4494**  **4457**  **4477**  **4494**  **4511**  **4474** | **4487** |
| **1** | **1**  **2**  **3**  **4**  **5**  **6**  **7**  **8**  **9**  **10**  **11**  **12**  **13**  **14**  **15**  **16**  **17**  **18**  **19**  **20**  **21**  **22**  **23**  **24**  **25**  **26**  **27**  **28**  **29**  **30**  **31** | **7/6/05**  **7/6/05**  **7/6/05**  **7/6/05**  **7/6/05**  **7/6/05**  **7/6/05**  **7/6/05**  **7/6/05**  **7/6/05**  **7/6/05**  **7/6/05**  **7/6/05**  **7/6/05**  **7/6/05**  **7/6/05**  **7/6/05**  **7/6/05**  **7/6/05**  **7/6/05**  **7/6/05**  **7/6/05**  **7/6/05**  **7/6/05**  **7/6/05**  **7/6/05**  **7/6/05**  **7/6/05**  **7/6/05**  **7/6/05**  **7/6/05** | **CD006B1_M2**  **CD006B1_M15**  **CD006B1_M27**  **CD006B1_M37**  **CD006B1_M48**  **CD006B1_M73**  **CD006B1_M3**  **CD006B1_M16**  **CD006B1_M28**  **CD006B1_M39**  **CD006B1_M51**  **CD006B1_M75**  **CD006B1_M4**  **CD006B1_M19**  **CD006B1_M29**  **CD006B1_M40**  **CD006B1_M52**  **CD006B1_M82**  **CD006B1_M5**  **CD006B1_M20**  **CD006B1_M31**  **CD006B1_M41**  **CD006B1_M53**  **CD006B1_M6**  **CD006B1_M23**  **CD006B1_M32**  **CD006B1_M54**  **CD006B1_M8**  **CD006B1_M24**  **CD006B1_M33**  **CD006B1_M45** | **D5S346**  **D5S346**  **D5S346**  **D5S346**  **D5S346**  **D5S346**  **D5S346**  **D5S346**  **D5S346**  **D5S346**  **D5S346**  **D5S346**  **D5S346**  **D5S346**  **D5S346**  **D5S346**  **D5S346**  **D5S346**  **D5S346**  **D5S346**  **D5S346**  **D5S346**  **D5S346**  **D5S346**  **D5S346**  **D5S346**  **D5S346**  **D5S346**  **D5S346**  **D5S346**  **D5S346** | **92**  **92**  **92**  **92**  **92**  **92**  **92**  **92**  **92**  **92**  **92**  **92**  **92**  **92**  **92**  **92**  **92**  **92**  **92**  **92**  **92**  **92**  **92**  **92**  **92**  **92**  **92**  **92**  **92**  **92**  **92** | **94**  **94**  **94**  **94**  **94**  **94**  **94**  **94**  **94**  **94**  **94**  **94**  **94**  **94**  **94**  **94**  **94**  **94**  **94**  **94**  **94**  **94**  **94**  **94**  **94**  **94**  **94**  **94**  **94**  **94**  **94** | **91.7**  **91.6**  **91.6**  **91.6**  **91.6**  **91.6**  **91.7**  **91.6**  **91.6**  **91.6**  **91.6**  **91.6**  **91.7**  **91.6**  **91.6**  **91.6**  **91.6**  **91.6**  **91.7**  **91.6**  **91.6**  **91.6**  **91.6**  **91.6**  **91.6**  **91.6**  **91.6**  **91.5**  **91.6**  **91.5**  **91.6** | **93.9**  **93.8**  **93.9**  **93.8**  **93.9**  **93.9**  **94.0**  **93.8**  **93.8**  **93.9**  **93.8**  **93.8**  **93.9**  **93.8**  **93.8**  **93.8**  **93.7**  **93.9**  **93.9**  **93.9**  **93.9**  **93.9**  **93.8**  **93.8**  **93.9**  **93.9**  **93.9**  **93.8**  **93.8**  **93.8**  **93.8** | **3824**  **4043**  **4830**  **3207**  **3058**  **1688**  **2595**  **3286**  **1504**  **3677**  **3138**  **1397**  **3519**  **3208**  **1833**  **3131**  **1234**  **1110**  **1997**  **4766**  **2701**  **3903**  **4150**  **2824**  **3108**  **3249**  **1934**  **3181**  **3039**  **2668**  **3267** | **2545**  **2668**  **3076**  **2014**  **1751**  **1087**  **1565**  **2268**  **952**  **2428**  **1316**  **969**  **2348**  **2167**  **1134**  **2221**  **726**  **736**  **1436**  **3067**  **1918**  **2673**  **2547**  **1892**  **2056**  **2070**  **1429**  **2156**  **2000**  **1654**  **2134** | **30121**  **31279**  **36990**  **25233**  **24099**  **13358**  **19989**  **24858**  **11339**  **27879**  **25141**  **10713**  **27309**  **24546**  **14111**  **24280**  **10199**  **8538**  **15565**  **36352**  **20822**  **30148**  **32440**  **21617**  **23215**  **25129**  **14743**  **24073**  **23033**  **20537**  **25096** | **18558**  **19336**  **21852**  **14644**  **12787**  **8189**  **11310**  **16104**  **6538**  **17443**  **9331**  **6993**  **17318**  **15295**  **8121**  **16222**  **5342**  **5273**  **10762**  **22176**  **13998**  **19108**  **18692**  **13549**  **14546**  **14996**  **10274**  **15062**  **14347**  **11718**  **15214** | **2987**  **2908**  **2877**  **2894**  **2918**  **2935**  **2964**  **2886**  **2854**  **2874**  **2897**  **2915**  **2979**  **2904**  **2872**  **2891**  **2913**  **2910**  **2991**  **2911**  **2881**  **2898**  **2920**  **2907**  **2892**  **2908**  **2932**  **2885**  **2870**  **2886**  **2894** | **3017**  **2937**  **2906**  **2923**  **2948**  **2965**  **2994**  **2914**  **2883**  **2903**  **2926**  **2944**  **3008**  **2933**  **2900**  **2919**  **2941**  **2939**  **3020**  **2940**  **2910**  **2927**  **2949**  **2935**  **2921**  **2937**  **2961**  **2914**  **2898**  **2915**  **2922** |

| **Day** | **Count** | **Run Date** | **Sample Name** | **Marker** | **Allele 1** | **Allele 2** | **Size 1** | **Size 2** | **Height 1** | **Height 2** | **Peak Area 1** | **Peak Area 2** | **Data Point 1** | **Data Point 2** |
| --- | --- | --- | --- | --- | --- | --- | --- | --- | --- | --- | --- | --- | --- | --- |
| **2** | **32**  **33**  **34**  **35**  **36**  **37**  **38**  **39**  **40**  **41**  **42** | **7/6/05**  **7/6/05**  **7/6/05**  **7/6/05**  **7/6/05**  **7/6/05**  **7/6/05**  **7/6/05**  **7/6/05**  **7/6/05**  **7/7/05** | **CD006B1_M55**  **CD006B1_M9**  **CD006B1_M25**  **CD006B1_M35**  **CD006B1_M46**  **CD006B1_M65**  **CD006B1_M26**  **CD006B1_M36**  **CD006B1_M47**  **CD006B1_M67**  **CD006B1_M43-REDO** | **D5S346**  **D5S346**  **D5S346**  **D5S346**  **D5S346**  **D5S346**  **D5S346**  **D5S346**  **D5S346**  **D5S346**  **D5S346** | **92**  **92**  **92**  **92**  **92**  **92**  **92**  **92**  **92**  **92**  **92** | **94**  **94**  **94**  **94**  **94**  **94**  **94**  **94**  **94**  **94**  **94** | **91.6**  **91.6**  **91.5**  **91.6**  **91.6**  **91.6**  **91.6**  **91.6**  **91.6**  **91.6**  **91.6** | **93.8**  **93.9**  **93.7**  **93.8**  **93.8**  **93.8**  **93.8**  **93.9**  **93.8**  **93.8**  **93.8** | **3089**  **3141**  **4435**  **3666**  **3068**  **3830**  **4463**  **2764**  **3394**  **3579**  **1848** | **2027**  **2108**  **2882**  **2379**  **2126**  **2374**  **2997**  **1790**  **2241**  **2244**  **1214** | **24241**  **24128**  **33111**  **27972**  **23723**  **29880**  **34220**  **21165**  **26651**  **28025**  **13961** | **14447**  **15034**  **20702**  **17028**  **15413**  **17451**  **21614**  **12832**  **16473**  **16404**  **8669** | **2912**  **2904**  **2889**  **2904**  **2914**  **2927**  **2896**  **2912**  **2923**  **2934**  **2907** | **2941**  **2933**  **2917**  **2933**  **2943**  **2955**  **2924**  **2941**  **2952**  **2963**  **2936** |
| **1**  **2** | **1**  **2**  **3**  **4**  **5**  **6**  **7**  **8**  **9**  **10**  **11**  **12**  **13**  **14**  **15**  **16**  **17**  **18**  **19**  **20**  **21**  **22**  **23**  **24**  **25**  **26**  **27**  **28**  **29**  **30**  **31**  **32**  **33**  **34**  **35**  **36**  **37**  **38**  **39**  **40**  **41**  **42** | **7/6/05**  **7/6/05**  **7/6/05**  **7/6/05**  **7/6/05**  **7/6/05**  **7/6/05**  **7/6/05**  **7/6/05**  **7/6/05**  **7/6/05**  **7/6/05**  **7/6/05**  **7/6/05**  **7/6/05**  **7/6/05**  **7/6/05**  **7/6/05**  **7/6/05**  **7/6/05**  **7/6/05**  **7/6/05**  **7/6/05**  **7/6/05**  **7/6/05**  **7/6/05**  **7/6/05**  **7/6/05**  **7/6/05**  **7/6/05**  **7/6/05**  **7/6/05**  **7/6/05**  **7/6/05**  **7/6/05**  **7/6/05**  **7/6/05**  **7/6/05**  **7/6/05**  **7/6/05**  **7/6/05**  **7/7/05** | **CD006B1_M2**  **CD006B1_M15**  **CD006B1_M27**  **CD006B1_M37**  **CD006B1_M48**  **CD006B1_M73**  **CD006B1_M3**  **CD006B1_M16**  **CD006B1_M28**  **CD006B1_M39**  **CD006B1_M51**  **CD006B1_M75**  **CD006B1_M4**  **CD006B1_M19**  **CD006B1_M29**  **CD006B1_M40**  **CD006B1_M52**  **CD006B1_M82**  **CD006B1_M5**  **CD006B1_M20**  **CD006B1_M31**  **CD006B1_M41**  **CD006B1_M53**  **CD006B1_M6**  **CD006B1_M23**  **CD006B1_M32**  **CD006B1_M54**  **CD006B1_M8**  **CD006B1_M24**  **CD006B1_M33**  **CD006B1_M45**  **CD006B1_M55**  **CD006B1_M9**  **CD006B1_M25**  **CD006B1_M35**  **CD006B1_M46**  **CD006B1_M65**  **CD006B1_M26**  **CD006B1_M36**  **CD006B1_M47**  **CD006B1_M67**  **CD006B1_M43-REDO** | **D6S262**  **D6S262**  **D6S262**  **D6S262**  **D6S262**  **D6S262**  **D6S262**  **D6S262**  **D6S262**  **D6S262**  **D6S262**  **D6S262**  **D6S262**  **D6S262**  **D6S262**  **D6S262**  **D6S262**  **D6S262**  **D6S262**  **D6S262**  **D6S262**  **D6S262**  **D6S262**  **D6S262**  **D6S262**  **D6S262**  **D6S262**  **D6S262**  **D6S262**  **D6S262**  **D6S262**  **D6S262**  **D6S262**  **D6S262**  **D6S262**  **D6S262**  **D6S262**  **D6S262**  **D6S262**  **D6S262**  **D6S262**  **D6S262** | **182**  **182**  **182**  **182**  **182**  **182**  **182**  **182**  **182**  **182**  **182**  **182**  **182**  **182**  **182**  **182**  **182**  **182**  **182**  **182**  **182**  **182**  **182**  **182**  **182**  **182**  **182**  **182**  **182**  **182**  **182**  **182**  **182**  **182**  **182**  **182**  **182**  **182**  **182**  **182**  **182**  **182** | **184**  **184**  **184**  **184**  **184**  **184**  **184**  **184**  **184**  **184**  **184**  **184**  **184**  **184**  **184**  **184**  **184**  **184**  **184**  **184**  **184**  **184**  **184**  **184**  **184**  **184**  **184**  **184**  **184**  **184**  **184**  **184**  **184**  **184**  **184**  **184**  **184**  **184**  **184**  **184**  **184**  **184** | **182.0**  **181.8**  **181.8**  **181.8**  **181.9**  **181.9**  **181.9**  **181.8**  **181.8**  **181.8**  **181.9**  **181.9**  **182.0**  **181.8**  **181.8**  **181.8**  **181.8**  **182.0**  **181.9**  **181.9**  **181.9**  **181.9**  **181.9**  **181.9**  **181.9**  **182.0**  **181.9**  **181.8**  **181.8**  **181.8**  **181.8**  **182.0**  **181.9**  **181.8**  **181.9**  **181.8**  **181.8**  **181.9**  **181.8**  **181.9**  **181.9**  **181.9** | **183.9**  **183.7**  **183.7**  **183.7**  **183.8**  **183.9**  **183.9**  **183.8**  **183.7**  **183.7**  **183.8**  **183.8**  **183.9**  **183.8**  **183.7**  **183.8**  **183.8**  **183.8**  **183.9**  **183.8**  **183.8**  **183.8**  **183.8**  **183.8**  **183.8**  **183.9**  **183.8**  **183.8**  **183.8**  **183.8**  **183.8**  **183.9**  **183.7**  **183.8**  **183.8**  **183.8**  **183.8**  **183.8**  **183.9**  **183.8**  **183.9**  **183.8** | **3916**  **4095**  **4909**  **3097**  **2733**  **1739**  **3055**  **4053**  **1618**  **4649**  **2342**  **1603**  **3669**  **4182**  **2050**  **3198**  **1373**  **1256**  **1785**  **4836**  **3399**  **4562**  **4355**  **3132**  **3750**  **3449**  **2352**  **3361**  **3632**  **2941**  **3595**  **3257**  **3580**  **4799**  **3665**  **3658**  **4049**  **4497**  **2779**  **3386**  **3502**  **2305** | **2367**  **2578**  **3129**  **1878**  **1697**  **1014**  **1750**  **2420**  **953**  **2807**  **1624**  **989**  **2214**  **2561**  **1256**  **2028**  **876**  **832**  **1125**  **2966**  **2101**  **2896**  **2682**  **1977**  **2384**  **2132**  **1426**  **1966**  **2212**  **1880**  **2171**  **2012**  **2137**  **2948**  **2176**  **2193**  **2485**  **2867**  **1771**  **1947**  **2073**  **1461** | **33904**  **32768**  **39815**  **25551**  **22438**  **13905**  **25591**  **33245**  **12890**  **38454**  **19006**  **12443**  **31268**  **33539**  **16906**  **25694**  **11254**  **10200**  **15206**  **39675**  **27666**  **36981**  **35334**  **25494**  **30253**  **28332**  **19173**  **27693**  **29291**  **23907**  **29597**  **26704**  **29438**  **38620**  **29485**  **30386**  **32576**  **36901**  **22681**  **27851**  **28826**  **18649** | **18224**  **19583**  **22947**  **14363**  **12884**  **7435**  **13461**  **18083**  **6990**  **21274**  **12342**  **7247**  **16748**  **19022**  **9380**  **14803**  **6866**  **5881**  **8594**  **21962**  **15816**  **21690**  **19914**  **15003**  **17708**  **15913**  **10969**  **14629**  **16498**  **14067**  **16552**  **15193**  **16059**  **21528**  **16112**  **16567**  **18717**  **20892**  **13319**  **14642**  **15653**  **10829** | **4143**  **4041**  **4003**  **4025**  **4056**  **4076**  **4114**  **4012**  **3972**  **3998**  **4028**  **4050**  **4130**  **4030**  **3991**  **4015**  **4043**  **4035**  **4148**  **4044**  **4008**  **4028**  **4056**  **4039**  **4019**  **4039**  **4072**  **4013**  **3990**  **4011**  **4022**  **4047**  **4031**  **4011**  **4029**  **4043**  **4060**  **4024**  **4043**  **4058**  **4074**  **4040** | **4167**  **4065**  **4027**  **4049**  **4080**  **4100**  **4139**  **4036**  **3996**  **4022**  **4052**  **4074**  **4154**  **4054**  **4015**  **4039**  **4067**  **4058**  **4173**  **4068**  **4032**  **4052**  **4080**  **4063**  **4043**  **4063**  **4096**  **4037**  **4014**  **4035**  **4046**  **4071**  **4054**  **4035**  **4053**  **4067**  **4084**  **4048**  **4068**  **4082**  **4098**  **4064** |
| **1** | **1**  **2**  **3**  **4**  **5** | **7/6/05**  **7/6/05**  **7/6/05**  **7/6/05**  **7/6/05** | **CD006B1_M2**  **CD006B1_M15**  **CD006B1_M27**  **CD006B1_M37**  **CD006B1_M48** | **D7S481**  **D7S481**  **D7S481**  **D7S481**  **D7S481** | **187**  **187**  **187**  **187**  **187** | **197**  **197**  **197**  **197**  **197** | **187.3**  **187.2**  **187.1**  **187.1**  **187.3** | **197.0**  **196.8**  **196.7**  **196.7**  **196.9** | **1082**  **907**  **1031**  **676**  **729** | **797**  **741**  **791**  **506**  **592** | **11246**  **8810**  **9873**  **6525**  **6997** | **7956**  **6724**  **7268**  **4626**  **5532** | **4210**  **4108**  **4069**  **4091**  **4123** | **4332**  **4227**  **4187**  **4210**  **4243** |

| **Day** | **Count** | **Run Date** | **Sample Name** | **Marker** | **Allele 1** | **Allele 2** | **Size 1** | **Size 2** | **Height 1** | **Height 2** | **Peak Area 1** | **Peak Area 2** | **Data Point 1** | **Data Point 2** |
| --- | --- | --- | --- | --- | --- | --- | --- | --- | --- | --- | --- | --- | --- | --- |
| **2** | **6**  **7**  **8**  **9**  **10**  **11**  **12**  **13**  **14**  **15**  **16**  **17**  **18**  **19**  **20**  **21**  **22**  **23**  **24**  **25**  **26**  **27**  **28**  **29**  **30**  **31**  **32**  **33**  **34**  **35**  **36**  **37**  **38**  **39**  **40**  **41**  **42** | **7/6/05**  **7/6/05**  **7/6/05**  **7/6/05**  **7/6/05**  **7/6/05**  **7/6/05**  **7/6/05**  **7/6/05**  **7/6/05**  **7/6/05**  **7/6/05**  **7/6/05**  **7/6/05**  **7/6/05**  **7/6/05**  **7/6/05**  **7/6/05**  **7/6/05**  **7/6/05**  **7/6/05**  **7/6/05**  **7/6/05**  **7/6/05**  **7/6/05**  **7/6/05**  **7/6/05**  **7/6/05**  **7/6/05**  **7/6/05**  **7/6/05**  **7/6/05**  **7/6/05**  **7/6/05**  **7/6/05**  **7/6/05**  **7/7/05** | **CD006B1_M73**  **CD006B1_M3**  **CD006B1_M16**  **CD006B1_M28**  **CD006B1_M39**  **CD006B1_M51**  **CD006B1_M75**  **CD006B1_M4**  **CD006B1_M19**  **CD006B1_M29**  **CD006B1_M40**  **CD006B1_M52**  **CD006B1_M82**  **CD006B1_M5**  **CD006B1_M20**  **CD006B1_M31**  **CD006B1_M41**  **CD006B1_M53**  **CD006B1_M6**  **CD006B1_M23**  **CD006B1_M32**  **CD006B1_M54**  **CD006B1_M8**  **CD006B1_M24**  **CD006B1_M33**  **CD006B1_M45**  **CD006B1_M55**  **CD006B1_M9**  **CD006B1_M25**  **CD006B1_M35**  **CD006B1_M46**  **CD006B1_M65**  **CD006B1_M26**  **CD006B1_M36**  **CD006B1_M47**  **CD006B1_M67**  **CD006B1_M43-REDO** | **D7S481**  **D7S481**  **D7S481**  **D7S481**  **D7S481**  **D7S481**  **D7S481**  **D7S481**  **D7S481**  **D7S481**  **D7S481**  **D7S481**  **D7S481**  **D7S481**  **D7S481**  **D7S481**  **D7S481**  **D7S481**  **D7S481**  **D7S481**  **D7S481**  **D7S481**  **D7S481**  **D7S481**  **D7S481**  **D7S481**  **D7S481**  **D7S481**  **D7S481**  **D7S481**  **D7S481**  **D7S481**  **D7S481**  **D7S481**  **D7S481**  **D7S481**  **D7S481** | **187**  **187**  **187**  **187**  **187**  **187**  **187**  **187**  **187**  **187**  **187**  **187**  **187**  **187**  **187**  **187**  **187**  **187**  **187**  **187**  **187**  **187**  **187**  **187**  **187**  **187**  **187**  **187**  **187**  **187**  **187**  **187**  **187**  **187**  **187**  **187**  **187** | **197**  **197**  **197**  **197**  **197**  **197**  **197**  **197**  **197**  **197**  **197**  **197**  **197**  **197**  **197**  **197**  **197**  **197**  **197**  **197**  **197**  **197**  **197**  **197**  **197**  **197**  **197**  **197**  **197**  **197**  **197**  **197**  **197**  **197**  **197**  **197**  **197** | **187.2**  **187.3**  **187.2**  **187.2**  **187.1**  **187.2**  **187.2**  **187.3**  **187.2**  **187.1**  **187.2**  **187.2**  **187.2**  **187.3**  **187.2**  **187.1**  **187.2**  **187.1**  **187.2**  **187.2**  **187.2**  **187.2**  **187.2**  **187.1**  **187.2**  **187.2**  **187.3**  **187.1**  **187.2**  **187.2**  **187.2**  **187.2**  **187.2**  **187.2**  **187.1**  **187.2**  **187.1** | **196.8**  **197.0**  **196.7**  **196.7**  **196.7**  **196.8**  **196.8**  **196.9**  **196.8**  **196.7**  **196.7**  **196.7**  **196.8**  **196.9**  **196.8**  **196.8**  **196.8**  **196.8**  **196.8**  **196.8**  **196.8**  **196.8**  **196.8**  **196.7**  **196.8**  **196.8**  **196.8**  **196.8**  **196.7**  **196.8**  **196.8**  **196.8**  **196.8**  **196.8**  **196.8**  **196.8**  **196.8** | **391**  **755**  **864**  **504**  **893**  **614**  **403**  **858**  **949**  **653**  **697**  **433**  **278**  **312**  **1109**  **905**  **1009**  **946**  **869**  **807**  **793**  **571**  **749**  **851**  **670**  **754**  **734**  **1004**  **1131**  **844**  **867**  **920**  **1105**  **798**  **692**  **752**  **481** | **307**  **631**  **627**  **470**  **693**  **706**  **290**  **634**  **749**  **499**  **497**  **390**  **211**  **253**  **897**  **682**  **802**  **830**  **657**  **734**  **673**  **418**  **596**  **606**  **570**  **596**  **543**  **726**  **844**  **641**  **638**  **726**  **836**  **604**  **526**  **537**  **401** | **3444**  **7428**  **8270**  **4651**  **8671**  **5862**  **3644**  **8589**  **9101**  **5912**  **6641**  **3989**  **2657**  **3059**  **10796**  **8607**  **9599**  **9152**  **8158**  **7722**  **7621**  **5327**  **7114**  **7908**  **6316**  **7197**  **6825**  **9704**  **10761**  **8067**  **8619**  **8739**  **10627**  **7635**  **6628**  **7234**  **4510** | **2788**  **5772**  **5780**  **4140**  **6364**  **6318**  **2586**  **6171**  **6773**  **4657**  **4581**  **3556**  **1922**  **2396**  **8494**  **6250**  **7356**  **7492**  **6084**  **6841**  **6028**  **3836**  **5490**  **5601**  **5098**  **5593**  **4982**  **6708**  **7774**  **5841**  **6045**  **6622**  **7687**  **5383**  **4831**  **5071**  **3437** | **4142**  **4182**  **4078**  **4038**  **4064**  **4094**  **4117**  **4197**  **4096**  **4057**  **4081**  **4109**  **4099**  **4216**  **4110**  **4073**  **4094**  **4122**  **4105**  **4085**  **4104**  **4139**  **4079**  **4055**  **4077**  **4089**  **4113**  **4096**  **4077**  **4095**  **4110**  **4126**  **4090**  **4109**  **4124**  **4140**  **4105** | **4262**  **4303**  **4196**  **4155**  **4182**  **4213**  **4236**  **4318**  **4214**  **4174**  **4198**  **4227**  **4217**  **4338**  **4229**  **4192**  **4213**  **4242**  **4224**  **4203**  **4223**  **4259**  **4198**  **4173**  **4195**  **4207**  **4232**  **4215**  **4194**  **4213**  **4228**  **4245**  **4208**  **4229**  **4244**  **4260**  **4225** |
| **1** | **1**  **1**  **1**  **1**  **1**  **1** | **11/14/05**  **11/14/05**  **11/14/05**  **11/14/05**  **11/14/05**  **11/14/05** | **CD007B1M2**  **CD007B1M2**  **CD007B1M2**  **CD007B1M2**  **CD007B1M2**  **CD007B1M2** | **BAT25**  **BAT26**  **D3S3623**  **D5S346**  **D6S262**  **D7S481** | **128**  **122**  **225**  **94**  **172**  **201** | **227**  **203** | **127.8**  **121.7**  **224.8**  **93.8**  **172.1**  **200.6** | **226.8**  **202.7** | **3021**  **1624**  **3794**  **6563**  **6331**  **1193** | **2351**  **820** | **21674**  **16952**  **34573**  **57316**  **72428**  **11630** | **19950**  **7144** | **3614**  **3533**  **4817**  **3152**  **4168**  **4535** | **4841**  **4560** |
| **1** | **1**  **2**  **3**  **4**  **5**  **6**  **7** | **11/14/05**  **11/14/05**  **11/14/05**  **11/14/05**  **11/14/05**  **11/14/05**  **11/14/05** | **CD008B1M1**  **CD008B1M2**  **CD008B1M3**  **CD008B1M5**  **CD008B1M6**  **CD008B1M7**  **CD008B1M8** | **BAT25**  **BAT25**  **BAT25**  **BAT25**  **BAT25**  **BAT25**  **BAT25** | **127**  **127**  **127**  **127**  **127**  **127**  **127** | **128** | **126.8**  **126.8**  **126.7**  **126.8**  **126.8**  **126.8**  **126.8** | **127.8** | **3823**  **5984**  **4739**  **5261**  **4784**  **4656**  **4667** | **5856** | **30949**  **57763**  **39715**  **40897**  **37917**  **36481**  **38632** | **49142** | **3653**  **3619**  **3619**  **3580**  **3630**  **3650**  **3648** | **3633** |
| **1** | **1**  **2**  **3**  **4**  **5**  **6**  **7** | **11/14/05**  **11/14/05**  **11/14/05**  **11/14/05**  **11/14/05**  **11/14/05**  **11/14/05** | **CD008B1M1**  **CD008B1M2**  **CD008B1M3**  **CD008B1M5**  **CD008B1M6**  **CD008B1M7**  **CD008B1M8** | **BAT26**  **BAT26**  **BAT26**  **BAT26**  **BAT26**  **BAT26**  **BAT26** | **122**  **122**  **122**  **122**  **122**  **122**  **122** |  | **121.6**  **121.8**  **121.7**  **121.6**  **121.7**  **121.7**  **121.7** |  | **987**  **2174**  **1592**  **1882**  **1526**  **1416**  **1474** |  | **9685**  **22167**  **17626**  **19945**  **15856**  **13629**  **15709** |  | **3584**  **3552**  **3552**  **3512**  **3562**  **3581**  **3579** |  |

| **Day** | **Count** | **Run Date** | **Sample Name** | **Marker** | **Allele 1** | **Allele 2** | **Size 1** | **Size 2** | **Height 1** | **Height 2** | **Peak Area 1** | **Peak Area 2** | **Data Point 1** | **Data Point 2** |
| --- | --- | --- | --- | --- | --- | --- | --- | --- | --- | --- | --- | --- | --- | --- |
| **1** | **1**  **2**  **3**  **4**  **5**  **6**  **7** | **11/14/05**  **11/14/05**  **11/14/05**  **11/14/05**  **11/14/05**  **11/14/05**  **11/14/05** | **CD008B1M1**  **CD008B1M2**  **CD008B1M3**  **CD008B1M5**  **CD008B1M6**  **CD008B1M7**  **CD008B1M8** | **D3S3623**  **D3S3623**  **D3S3623**  **D3S3623**  **D3S3623**  **D3S3623**  **D3S3623** | **221**  **221**  **221**  **221**  **221**  **221**  **221** | **227**  **227**  **227**  **227**  **227**  **227**  **227** | **220.7**  **220.7**  **220.6**  **220.7**  **220.7**  **220.7**  **220.7** | **227.1**  **227.0**  **226.9**  **227.0**  **227.0**  **227.0**  **227.0** | **2477**  **6891**  **3559**  **3998**  **3410**  **3443**  **3199** | **2903**  **7139**  **3551**  **4028**  **3570**  **3279**  **3311** | **25988**  **66879**  **36998**  **38652**  **36299**  **33385**  **34146** | **30943**  **69722**  **37304**  **38954**  **37878**  **32136**  **35330** | **4837**  **4791**  **4787**  **4737**  **4802**  **4838**  **4831** | **4913**  **4865**  **4861**  **4810**  **4876**  **4913**  **4906** |
| **1** | **1**  **2**  **3**  **4**  **5**  **6**  **7** | **11/14/05**  **11/14/05**  **11/14/05**  **11/14/05**  **11/14/05**  **11/14/05**  **11/14/05** | **CD008B1M1**  **CD008B1M2**  **CD008B1M3**  **CD008B1M5**  **CD008B1M6**  **CD008B1M7**  **CD008B1M8** | **D5S346**  **D5S346**  **D5S346**  **D5S346**  **D5S346**  **D5S346**  **D5S346** | **94**  **94**  **94**  **94**  **94**  **94**  **94** | **101**  **101**  **101**  **101**  **101**  **101**  **101** | **93.9**  **93.8**  **93.9**  **93.8**  **93.8**  **93.8**  **93.9** | **100.6**  **100.5**  **100.5**  **100.5**  **100.5**  **100.5**  **100.6** | **2088**  **4605**  **3661**  **3699**  **2863**  **2793**  **3025** | **1424**  **2915**  **2465**  **2299**  **1906**  **1486**  **1962** | **18260**  **40267**  **32989**  **30990**  **25283**  **24715**  **26997** | **12313**  **24678**  **20546**  **18957**  **15944**  **12460**  **16986** | **3199**  **3169**  **3171**  **3135**  **3180**  **3194**  **3194** | **3289**  **3258**  **3259**  **3223**  **3269**  **3284**  **3284** |
| **1** | **1**  **2**  **3**  **4**  **5**  **6**  **7** | **11/14/05**  **11/14/05**  **11/14/05**  **11/14/05**  **11/14/05**  **11/14/05**  **11/14/05** | **CD008B1M1**  **CD008B1M2**  **CD008B1M3**  **CD008B1M5**  **CD008B1M6**  **CD008B1M7**  **CD008B1M8** | **D6S262**  **D6S262**  **D6S262**  **D6S262**  **D6S262**  **D6S262**  **D6S262** | **182**  **182**  **182**  **182**  **182**  **182**  **182** | **184**  **184**  **184**  **184**  **184**  **184**  **184** | **182.0**  **181.9**  **181.9**  **181.9**  **181.9**  **181.9**  **181.9** | **183.9**  **183.8**  **183.8**  **183.8**  **183.8**  **183.8**  **183.8** | **2585**  **5884**  **4198**  **4624**  **3466**  **3200**  **3346** | **1700**  **5101**  **2554**  **2539**  **1913**  **1765**  **1950** | **23808**  **55453**  **38703**  **40771**  **32714**  **28826**  **31100** | **14298**  **40021**  **20381**  **19431**  **15659**  **13728**  **15845** | **4356**  **4316**  **4313**  **4267**  **4327**  **4355**  **4350** | **4381**  **4340**  **4338**  **4291**  **4351**  **4380**  **4375** |
| **1** | **1**  **2**  **3**  **4**  **5**  **6**  **7** | **11/14/05**  **11/14/05**  **11/14/05**  **11/14/05**  **11/14/05**  **11/14/05**  **11/14/05** | **CD008B1M1**  **CD008B1M2**  **CD008B1M3**  **CD008B1M5**  **CD008B1M6**  **CD008B1M7**  **CD008B1M8** | **D7S481**  **D7S481**  **D7S481**  **D7S481**  **D7S481**  **D7S481**  **D7S481** | **197**  **197**  **197**  **197**  **197**  **197**  **197** | **199**  **199**  **199**  **199**  **199**  **199**  **199** | **196.8**  **196.8**  **196.7**  **196.7**  **196.7**  **196.9**  **196.7** | **198.8**  **198.7**  **198.6**  **198.7**  **198.7**  **198.7**  **198.6** | **997**  **1870**  **1398**  **1783**  **1602**  **1617**  **1536** | **567**  **1406**  **981**  **1194**  **1044**  **1046**  **997** | **10364**  **18469**  **14603**  **17235**  **16417**  **15988**  **15646** | **5434**  **12633**  **9496**  **10557**  **9886**  **9746**  **9629** | **4550**  **4508**  **4504**  **4456**  **4518**  **4551**  **4544** | **4576**  **4532**  **4529**  **4481**  **4543**  **4575**  **4569** |
| **1** | **1**  **2**  **3**  **4**  **5**  **6** | **7/7/05**  **7/7/05**  **7/7/05**  **7/7/05**  **7/7/05**  **7/7/05** | **CD009B1_M27**  **CD009B1_M70**  **CD009B1_M30**  **CD009B1_M82**  **CD009B1_M31**  **CD009B1_M42** | **BAT25**  **BAT25**  **BAT25**  **BAT25**  **BAT25**  **BAT25** | **127**  **127**  **127**  **127**  **127**  **127** |  | **126.8**  **126.7**  **126.8**  **126.7**  **126.8**  **126.8** |  | **844**  **6201**  **2677**  **5791**  **2602**  **5815** |  | **7217**  **48682**  **21716**  **44021**  **21083**  **45919** |  | **3503**  **3333**  **3482**  **3309**  **3473**  **3384** |  |
| **2**  **3** | **7**  **8**  **9**  **10**  **11**  **12**  **13**  **14**  **15**  **16**  **17**  **18**  **19**  **20**  **21**  **22**  **23**  **24**  **25**  **26**  **27**  **28**  **29** | **7/7/05**  **7/7/05**  **7/7/05**  **7/7/05**  **7/7/05**  **7/7/05**  **7/7/05**  **7/7/05**  **7/7/05**  **7/7/05**  **7/7/05**  **7/7/05**  **7/13/05**  **7/13/05**  **7/13/05**  **7/13/05**  **7/15/05**  **7/15/05**  **7/15/05**  **7/15/05**  **7/15/05**  **7/15/05**  **7/15/05** | **CD009B1_M103**  **CD009B1_M33**  **CD009B1_M43**  **CD009B1_M108**  **CD009B1_M34**  **CD009B1_M117**  **CD009B1_M46**  **CD009B1_M11**  **CD009B1_M51**  **CD009B1_M6**  **CD009B1_M8**  **CD009B1_M22**  **CD009B1M5_1uL CD009B1M9_1uL CD009B1M37_1uL CD009B1M12_1uL CD009B1M32**  **CD009B1M35**  **CD009B1M2**  **CD009B1M3**  **CD009B1M4**  **CD009B1M41**  **CD009B1M14** | **BAT25**  **BAT25**  **BAT25**  **BAT25**  **BAT25**  **BAT25**  **BAT25**  **BAT25**  **BAT25**  **BAT25**  **BAT25**  **BAT25**  **BAT25**  **BAT25**  **BAT25**  **BAT25**  **BAT25**  **BAT25**  **BAT25**  **BAT25**  **BAT25**  **BAT25**  **BAT25** | **127**  **127**  **127**  **127**  **127**  **127**  **127**  **127**  **127**  **127**  **127**  **127**  **127**  **127**  **127**  **127**  **127**  **127**  **127**  **127**  **127**  **127**  **127** | **128** | **126.7**  **126.7**  **126.7**  **126.7**  **126.7**  **126.7**  **126.7**  **126.7**  **126.7**  **126.7**  **126.8**  **126.7**  **126.9**  **126.8**  **126.8**  **126.9**  **126.8**  **126.7**  **126.9**  **126.9**  **126.8**  **126.8**  **126.7** | **127.7** | **1962**  **5386**  **5233**  **6264**  **4866**  **4937**  **2682**  **3589**  **6311**  **2493**  **5025**  **4578**  **2165**  **3971**  **3172**  **2769**  **3672**  **846**  **3609**  **4593**  **2008**  **3388**  **899** | **6151** | **15459**  **42717**  **40078**  **52875**  **38259**  **37497**  **20467**  **27687**  **57787**  **19983**  **40154**  **36505**  **16993**  **30120**  **24753**  **21722**  **28263**  **7364**  **29244**  **36240**  **15795**  **26779**  **7754** | **49330** | **3344**  **3385**  **3354**  **3362**  **3362**  **3341**  **3330**  **3358**  **3365**  **3386**  **3381**  **3339**  **3470**  **3446**  **3429**  **3461**  **3363**  **3371**  **3597**  **3436**  **3416**  **3452**  **3427** | **3378** |

| **Day** | **Count** | **Run Date** | **Sample Name** | **Marker** | **Allele 1** | **Allele 2** | **Size 1** | **Size 2** | **Height 1** | **Height 2** | **Peak Area 1** | **Peak Area 2** | **Data Point 1** | **Data Point 2** |
| --- | --- | --- | --- | --- | --- | --- | --- | --- | --- | --- | --- | --- | --- | --- |
|  | **30**  **31** | **7/15/05**  **7/15/05** | **CD009B1M44**  **CD009B1M16** | **BAT25**  **BAT25** | **127**  **127** |  | **126.8**  **126.8** |  | **2372**  **5589** |  | **20053**  **42070** |  | **3462**  **3428** |  |
| **1**  **2**  **3** | **1**  **2**  **3**  **4**  **5**  **6**  **7**  **8**  **9**  **10**  **11**  **12**  **13**  **14**  **15**  **16**  **17**  **18**  **19**  **20**  **21**  **22**  **23**  **24**  **25**  **26**  **27**  **28**  **29**  **30**  **31** | **7/7/05**  **7/7/05**  **7/7/05**  **7/7/05**  **7/7/05**  **7/7/05**  **7/7/05**  **7/7/05**  **7/7/05**  **7/7/05**  **7/7/05**  **7/7/05**  **7/7/05**  **7/7/05**  **7/7/05**  **7/7/05**  **7/7/05**  **7/7/05**  **7/13/05**  **7/13/05**  **7/13/05**  **7/13/05**  **7/15/05**  **7/15/05**  **7/15/05**  **7/15/05**  **7/15/05**  **7/15/05**  **7/15/05**  **7/15/05**  **7/15/05** | **CD009B1_M27**  **CD009B1_M70**  **CD009B1_M30**  **CD009B1_M82**  **CD009B1_M31**  **CD009B1_M42**  **CD009B1_M103**  **CD009B1_M33**  **CD009B1_M43**  **CD009B1_M108**  **CD009B1_M34**  **CD009B1_M117**  **CD009B1_M46**  **CD009B1_M11**  **CD009B1_M51**  **CD009B1_M6**  **CD009B1_M8**  **CD009B1_M22**  **CD009B1M5_1uL CD009B1M9_1uL CD009B1M37_1uL CD009B1M12_1uL CD009B1M32**  **CD009B1M35**  **CD009B1M2**  **CD009B1M3**  **CD009B1M4**  **CD009B1M41**  **CD009B1M14**  **CD009B1M44**  **CD009B1M16** | **BAT26**  **BAT26**  **BAT26**  **BAT26**  **BAT26**  **BAT26**  **BAT26**  **BAT26**  **BAT26**  **BAT26**  **BAT26**  **BAT26**  **BAT26**  **BAT26**  **BAT26**  **BAT26**  **BAT26**  **BAT26**  **BAT26**  **BAT26**  **BAT26**  **BAT26**  **BAT26**  **BAT26**  **BAT26**  **BAT26**  **BAT26**  **BAT26**  **BAT26**  **BAT26**  **BAT26** | **122**  **123**  **122**  **123**  **122**  **122**  **122**  **122**  **122**  **122**  **123**  **123**  **122**  **122**  **123**  **122**  **122**  **123**  **123**  **123**  **123**  **123**  **123**  **123**  **122**  **122**  **123**  **123**  **123**  **122**  **123** | **123**  **123**  **123**  **123**  **123**  **123**  **123**  **123**  **123**  **123**  **123**  **123**  **123** | **121.7**  **122.5**  **121.7**  **122.6**  **121.7**  **121.8**  **121.6**  **121.6**  **121.6**  **121.6**  **122.6**  **122.5**  **121.5**  **121.6**  **122.5**  **121.6**  **121.7**  **122.6**  **122.8**  **122.6**  **122.7**  **122.7**  **122.6**  **122.6**  **121.9**  **121.7**  **122.7**  **122.6**  **122.5**  **121.7**  **122.7** | **122.7**  **122.7**  **122.6**  **122.5**  **122.6**  **122.5**  **122.6**  **122.6**  **122.5**  **122.5**  **122.6**  **122.7**  **122.6** | **178**  **986**  **527**  **1286**  **572**  **1057**  **423**  **795**  **835**  **633**  **933**  **930**  **585**  **569**  **1507**  **490**  **896**  **839**  **630**  **1556**  **978**  **1011**  **1198**  **316**  **984**  **986**  **680**  **1281**  **213**  **723**  **1400** | **186**  **536**  **1006**  **433**  **812**  **857**  **615**  **589**  **588**  **471**  **858**  **1032**  **728** | **1338**  **8729**  **4125**  **11876**  **4434**  **8711**  **3434**  **6747**  **7492**  **5410**  **7770**  **7642**  **5114**  **4860**  **13141**  **4037**  **7273**  **7283**  **5568**  **14461**  **9635**  **9539**  **11101**  **3176**  **7701**  **9355**  **5862**  **11671**  **2083**  **6456**  **12574** | **1202**  **4128**  **8018**  **3287**  **7021**  **7385**  **4913**  **4758**  **4779**  **3903**  **7270**  **9333**  **6181** | **3436**  **3279**  **3415**  **3257**  **3406**  **3319**  **3279**  **3320**  **3289**  **3297**  **3309**  **3288**  **3265**  **3293**  **3311**  **3320**  **3316**  **3287**  **3417**  **3392**  **3375**  **3407**  **3310**  **3319**  **3530**  **3370**  **3363**  **3398**  **3373**  **3396**  **3375** | **3449**  **3428**  **3330**  **3291**  **3332**  **3301**  **3309**  **3278**  **3305**  **3332**  **3327**  **3382**  **3408** |
| **1**  **2**  **3** | **1**  **2**  **3**  **4**  **5**  **6**  **7**  **8**  **9**  **10**  **11**  **12**  **13**  **14**  **15**  **16**  **17**  **18**  **19**  **20**  **21**  **22**  **23**  **24**  **25** | **7/7/05**  **7/7/05**  **7/7/05**  **7/7/05**  **7/7/05**  **7/7/05**  **7/7/05**  **7/7/05**  **7/7/05**  **7/7/05**  **7/7/05**  **7/7/05**  **7/7/05**  **7/7/05**  **7/7/05**  **7/7/05**  **7/7/05**  **7/7/05**  **7/13/05**  **7/13/05**  **7/13/05**  **7/13/05**  **7/15/05**  **7/15/05**  **7/15/05** | **CD009B1_M27**  **CD009B1_M70**  **CD009B1_M30**  **CD009B1_M82**  **CD009B1_M31**  **CD009B1_M42**  **CD009B1_M103**  **CD009B1_M33**  **CD009B1_M43**  **CD009B1_M108**  **CD009B1_M34**  **CD009B1_M117**  **CD009B1_M46**  **CD009B1_M11**  **CD009B1_M51**  **CD009B1_M6**  **CD009B1_M8**  **CD009B1_M22**  **CD009B1M5_1uL CD009B1M9_1uL CD009B1M37_1uL CD009B1M12_1uL CD009B1M32**  **CD009B1M35**  **CD009B1M2** | **D3S3623**  **D3S3623**  **D3S3623**  **D3S3623**  **D3S3623**  **D3S3623**  **D3S3623**  **D3S3623**  **D3S3623**  **D3S3623**  **D3S3623**  **D3S3623**  **D3S3623**  **D3S3623**  **D3S3623**  **D3S3623**  **D3S3623**  **D3S3623**  **D3S3623**  **D3S3623**  **D3S3623**  **D3S3623**  **D3S3623**  **D3S3623**  **D3S3623** | **219**  **219**  **219**  **219**  **219**  **219**  **219**  **219**  **219**  **219**  **219**  **219**  **219**  **219**  **219**  **219**  **219**  **219**  **219**  **219**  **219**  **219**  **219**  **219**  **219** | **221**  **221**  **221**  **221**  **221**  **221**  **221**  **221**  **221**  **221**  **221**  **221**  **221**  **221**  **221**  **221**  **221**  **221**  **221**  **221**  **221**  **221**  **221**  **221** | **218.8**  **218.5**  **218.8**  **218.4**  **218.8**  **218.6**  **218.5**  **218.6**  **218.4**  **218.5**  **218.5**  **218.5**  **218.5**  **218.5**  **218.6**  **218.6**  **218.5**  **218.5**  **218.7**  **218.5**  **218.5**  **218.6**  **218.4**  **218.5**  **218.9** | **220.5**  **220.9**  **220.5**  **220.9**  **220.6**  **220.6**  **220.6**  **220.6**  **220.6**  **220.6**  **220.6**  **220.6**  **220.6**  **220.6**  **220.8**  **220.7**  **220.6**  **220.7**  **220.6**  **220.6**  **220.6**  **220.5**  **220.4**  **220.9** | **359**  **5007**  **1701**  **4974**  **1436**  **5317**  **2321**  **4644**  **5239**  **2143**  **3998**  **3844**  **811**  **2745**  **7004**  **2195**  **4512**  **4182**  **1222**  **423**  **233**  **799**  **1128**  **112**  **2166** | **3311**  **1216**  **3453**  **860**  **3408**  **1164**  **3172**  **3769**  **1067**  **2708**  **2543**  **652**  **1953**  **4619**  **1318**  **2995**  **2807**  **863**  **283**  **173**  **560**  **728**  **77**  **1432** | **3366**  **40660**  **14946**  **40553**  **12839**  **44851**  **19664**  **39107**  **43349**  **17809**  **32988**  **31684**  **6619**  **23083**  **57883**  **18513**  **37385**  **33983**  **10246**  **3456**  **1963**  **6818**  **9154**  **931**  **19144** | **25279**  **9966**  **26334**  **7058**  **26992**  **9253**  **25118**  **29711**  **8390**  **20815**  **19801**  **5141**  **15647**  **36688**  **10570**  **23450**  **21365**  **7185**  **2277**  **1479**  **4461**  **5499**  **553**  **12057** | **4644**  **4430**  **4619**  **4401**  **4602**  **4494**  **4445**  **4494**  **4453**  **4467**  **4466**  **4441**  **4416**  **4459**  **4469**  **4493**  **4475**  **4428**  **4587**  **4558**  **4539**  **4573**  **4459**  **4464**  **4752** | **4453**  **4644**  **4424**  **4626**  **4517**  **4469**  **4517**  **4477**  **4491**  **4490**  **4464**  **4439**  **4483**  **4492**  **4517**  **4499**  **4451**  **4610**  **4582**  **4563**  **4596**  **4483**  **4486**  **4776** |

| **Day** | **Count** | **Run Date** | **Sample Name** | **Marker** | **Allele 1** | **Allele 2** | **Size 1** | **Size 2** | **Height 1** | **Height 2** | **Peak Area 1** | **Peak Area 2** | **Data Point 1** | **Data Point 2** |
| --- | --- | --- | --- | --- | --- | --- | --- | --- | --- | --- | --- | --- | --- | --- |
|  | **26**  **27**  **28**  **29**  **30**  **31** | **7/15/05**  **7/15/05**  **7/15/05**  **7/15/05**  **7/15/05**  **7/15/05** | **CD009B1M3**  **CD009B1M4**  **CD009B1M41**  **CD009B1M14**  **CD009B1M44**  **CD009B1M16** | **D3S3623**  **D3S3623**  **D3S3623**  **D3S3623**  **D3S3623**  **D3S3623** | **219**  **219**  **219**  **219**  **219**  **219** | **221**  **221**  **221**  **221**  **221**  **221** | **218.5**  **218.5**  **218.5**  **218.5**  **218.6**  **218.4** | **220.5**  **220.6**  **220.6**  **220.6**  **220.6**  **220.5** | **1471**  **495**  **529**  **124**  **227**  **1498** | **888**  **321**  **370**  **65**  **149**  **980** | **12142**  **4207**  **4255**  **1176**  **1967**  **12081** | **7182**  **2555**  **2883**  **514**  **1194**  **7494** | **4545**  **4523**  **4570**  **4529**  **4576**  **4533** | **4568**  **4546**  **4594**  **4552**  **4599**  **4556** |
| **1**  **2**  **3** | **1**  **2**  **3**  **4**  **5**  **6**  **7**  **8**  **9**  **10**  **11**  **12**  **13**  **14**  **15**  **16**  **17**  **18**  **19**  **20**  **21**  **22**  **23**  **24**  **25**  **26**  **27**  **28**  **29**  **30**  **31** | **7/7/05**  **7/7/05**  **7/7/05**  **7/7/05**  **7/7/05**  **7/7/05**  **7/7/05**  **7/7/05**  **7/7/05**  **7/7/05**  **7/7/05**  **7/7/05**  **7/7/05**  **7/7/05**  **7/7/05**  **7/7/05**  **7/7/05**  **7/7/05**  **7/13/05**  **7/13/05**  **7/13/05**  **7/13/05**  **7/15/05**  **7/15/05**  **7/15/05**  **7/15/05**  **7/15/05**  **7/15/05**  **7/15/05**  **7/15/05**  **7/15/05** | **CD009B1_M27**  **CD009B1_M70**  **CD009B1_M30**  **CD009B1_M82**  **CD009B1_M31**  **CD009B1_M42**  **CD009B1_M103**  **CD009B1_M33**  **CD009B1_M43**  **CD009B1_M108**  **CD009B1_M34**  **CD009B1_M117**  **CD009B1_M46**  **CD009B1_M11**  **CD009B1_M51**  **CD009B1_M6**  **CD009B1_M8**  **CD009B1_M22**  **CD009B1M5_1uL CD009B1M9_1uL CD009B1M37_1uL CD009B1M12_1uL CD009B1M32**  **CD009B1M35**  **CD009B1M2**  **CD009B1M4**  **CD009B1M41**  **CD009B1M14**  **CD009B1M44**  **CD009B1M16**  **CD009B1M3** | **D5S346**  **D5S346**  **D5S346**  **D5S346**  **D5S346**  **D5S346**  **D5S346**  **D5S346**  **D5S346**  **D5S346**  **D5S346**  **D5S346**  **D5S346**  **D5S346**  **D5S346**  **D5S346**  **D5S346**  **D5S346**  **D5S346**  **D5S346**  **D5S346**  **D5S346**  **D5S346**  **D5S346**  **D5S346**  **D5S346**  **D5S346**  **D5S346**  **D5S346**  **D5S346**  **D5S346** | **94**  **94**  **94**  **94**  **94**  **94**  **94**  **94**  **94**  **94**  **94**  **94**  **94**  **94**  **94**  **94**  **94**  **94**  **94**  **94**  **94**  **94**  **94**  **94**  **94**  **94**  **94**  **94**  **94**  **94**  **94** |  | **94.0**  **93.8**  **93.9**  **93.8**  **94.0**  **93.8**  **93.8**  **93.8**  **93.8**  **93.8**  **93.8**  **93.8**  **93.7**  **93.8**  **94.0**  **93.8**  **93.9**  **93.8**  **94.0**  **93.9**  **93.9**  **94.0**  **93.9**  **93.9**  **94.1**  **93.9**  **93.9**  **93.8**  **93.9**  **93.9**  **93.9** |  | **1302**  **5852**  **3103**  **6693**  **2162**  **6130**  **1345**  **4798**  **5257**  **3194**  **5732**  **5504**  **3286**  **3123**  **6920**  **2724**  **5203**  **5274**  **2750**  **7218**  **7182**  **3266**  **5199**  **1375**  **4694**  **2773**  **5013**  **831**  **4485**  **6029**  **4609** |  | **11211**  **48204**  **26709**  **54251**  **18723**  **51459**  **10903**  **40548**  **43284**  **26713**  **48239**  **45469**  **27055**  **25417**  **70986**  **23744**  **43944**  **44500**  **22987**  **62868**  **64739**  **28608**  **42040**  **12048**  **40415**  **23119**  **42947**  **7070**  **39657**  **49360**  **39052** |  | **3058**  **2902**  **3038**  **2882**  **3032**  **2949**  **2913**  **2951**  **2923**  **2930**  **2929**  **2911**  **2903**  **2927**  **2935**  **2952**  **2950**  **2912**  **3031**  **3009**  **2993**  **3023**  **2933**  **2942**  **3146**  **2982**  **3014**  **2994**  **3025**  **2994**  **3000** |  |
| **1**  **2** | **1**  **2**  **3**  **4**  **5**  **6**  **7**  **8**  **9**  **10**  **11**  **12**  **13**  **15**  **14**  **16**  **17**  **18**  **19**  **20**  **21** | **7/7/05**  **7/7/05**  **7/7/05**  **7/7/05**  **7/7/05**  **7/7/05**  **7/7/05**  **7/7/05**  **7/7/05**  **7/7/05**  **7/7/05**  **7/7/05**  **7/7/05**  **7/7/05**  **7/7/05**  **7/7/05**  **7/7/05**  **7/7/05**  **7/13/05**  **7/13/05**  **7/13/05** | **CD009B1_M27**  **CD009B1_M70**  **CD009B1_M30**  **CD009B1_M82**  **CD009B1_M31**  **CD009B1_M42**  **CD009B1_M103**  **CD009B1_M33**  **CD009B1_M43**  **CD009B1_M108**  **CD009B1_M34**  **CD009B1_M117**  **CD009B1_M46**  **CD009B1_M51**  **CD009B1_M11**  **CD009B1_M6**  **CD009B1_M8**  **CD009B1_M22**  **CD009B1M5_1uL CD009B1M9_1uL CD009B1M12_1uL** | **D6S262**  **D6S262**  **D6S262**  **D6S262**  **D6S262**  **D6S262**  **D6S262**  **D6S262**  **D6S262**  **D6S262**  **D6S262**  **D6S262**  **D6S262**  **D6S262**  **D6S262**  **D6S262**  **D6S262**  **D6S262**  **D6S262**  **D6S262**  **D6S262** | **170**  **170**  **170**  **170**  **170**  **170**  **170**  **170**  **170**  **170**  **170**  **170**  **170**  **170**  **170**  **170**  **170**  **170**  **170**  **170**  **170** | **172**  **172**  **172**  **172**  **172**  **172**  **172**  **172**  **172**  **172**  **172**  **172**  **172**  **172**  **172**  **172**  **172**  **172**  **172**  **172**  **172** | **170.5**  **170.2**  **170.3**  **170.2**  **170.4**  **170.2**  **170.2**  **170.2**  **170.2**  **170.2**  **170.2**  **170.2**  **170.2**  **170.3**  **170.3**  **170.3**  **170.3**  **170.3**  **170.3**  **170.3**  **170.3** | **172.4**  **172.1**  **172.4**  **172.1**  **172.3**  **172.1**  **172.2**  **172.2**  **172.2**  **172.2**  **172.2**  **172.2**  **172.3**  **172.2**  **172.2**  **172.2**  **172.2**  **172.2**  **172.3**  **172.3**  **172.3** | **390**  **4458**  **1873**  **4959**  **1336**  **4258**  **1209**  **3073**  **3746**  **1526**  **3803**  **4039**  **1059**  **6262**  **2229**  **1638**  **3250**  **3518**  **1500**  **1786**  **1461** | **203**  **3054**  **1311**  **3478**  **702**  **3028**  **606**  **2137**  **2474**  **988**  **2613**  **2928**  **857**  **4456**  **1266**  **1115**  **2055**  **2380**  **1048**  **1184**  **1042** | **3305**  **34446**  **15871**  **38627**  **11464**  **34362**  **9537**  **24867**  **29229**  **12270**  **29598**  **31898**  **8344**  **51434**  **17917**  **13170**  **25913**  **26996**  **11978**  **13710**  **11839** | **1385**  **22007**  **9987**  **24669**  **5217**  **22373**  **4219**  **15972**  **17826**  **7038**  **18413**  **21234**  **6231**  **32207**  **9396**  **8300**  **14978**  **16887**  **8107**  **8518**  **8354** | **4047**  **3855**  **4023**  **3830**  **4011**  **3912**  **3868**  **3913**  **3878**  **3889**  **3888**  **3865**  **3847**  **3891**  **3883**  **3913**  **3903**  **3858**  **4002**  **3976**  **3991** | **4071**  **3879**  **4049**  **3853**  **4035**  **3936**  **3892**  **3937**  **3902**  **3913**  **3912**  **3889**  **3872**  **3915**  **3906**  **3937**  **3927**  **3882**  **4027**  **4001**  **4015** |

| **Day** | **Count** | **Run Date** | **Sample Name** | **Marker** | **Allele 1** | **Allele 2** | **Size 1** | **Size 2** | **Height 1** | **Height 2** | **Peak Area 1** | **Peak Area 2** | **Data Point 1** | **Data Point 2** |
| --- | --- | --- | --- | --- | --- | --- | --- | --- | --- | --- | --- | --- | --- | --- |
| **3** | **22**  **23**  **24**  **25**  **26**  **27**  **28**  **29**  **30**  **31** | **7/13/05**  **7/15/05**  **7/15/05**  **7/15/05**  **7/15/05**  **7/15/05**  **7/15/05**  **7/15/05**  **7/15/05**  **7/15/05** | **CD009B1M37_1uL CD009B1M32**  **CD009B1M35**  **CD009B1M2**  **CD009B1M4**  **CD009B1M41**  **CD009B1M14**  **CD009B1M44**  **CD009B1M16**  **CD009B1M3** | **D6S262**  **D6S262**  **D6S262**  **D6S262**  **D6S262**  **D6S262**  **D6S262**  **D6S262**  **D6S262**  **D6S262** | **170**  **170**  **170**  **170**  **170**  **170**  **170**  **170**  **170**  **170** | **172**  **172**  **172**  **172**  **172**  **172**  **172**  **172**  **172**  **172** | **170.3**  **170.3**  **170.2**  **170.5**  **170.3**  **170.3**  **170.2**  **170.3**  **170.3**  **170.3** | **172.2**  **172.2**  **172.2**  **172.5**  **172.2**  **172.3**  **172.1**  **172.3**  **172.3**  **172.2** | **987**  **2164**  **197**  **2633**  **848**  **1297**  **179**  **692**  **2297**  **1713** | **662**  **1469**  **145**  **1850**  **487**  **948**  **135**  **470**  **1451**  **1160** | **7734**  **16479**  **1536**  **22312**  **6812**  **10114**  **1319**  **5386**  **17589**  **12983** | **4775**  **10319**  **1035**  **14828**  **3505**  **6670**  **1002**  **3411**  **10173**  **8439** | **3958**  **3885**  **3892**  **4147**  **3943**  **3985**  **3952**  **3992**  **3955**  **3964** | **3982**  **3909**  **3916**  **4172**  **3967**  **4010**  **3976**  **4017**  **3979**  **3988** |
| **1**  **2**  **3** | **1**  **2**  **3**  **4**  **5**  **6**  **7**  **8**  **9**  **10**  **11**  **12**  **13**  **15**  **14**  **16**  **17**  **18**  **19**  **20**  **21**  **22**  **23**  **24**  **25**  **26**  **27**  **28**  **29**  **30**  **31** | **7/7/05**  **7/7/05**  **7/7/05**  **7/7/05**  **7/7/05**  **7/7/05**  **7/7/05**  **7/7/05**  **7/7/05**  **7/7/05**  **7/7/05**  **7/7/05**  **7/7/05**  **7/7/05**  **7/7/05**  **7/7/05**  **7/7/05**  **7/7/05**  **7/13/05**  **7/13/05**  **7/13/05**  **7/13/05**  **7/15/05**  **7/15/05**  **7/15/05**  **7/15/05**  **7/15/05**  **7/15/05**  **7/15/05**  **7/15/05**  **7/15/05** | **CD009B1_M27**  **CD009B1_M70**  **CD009B1_M30**  **CD009B1_M82**  **CD009B1_M31**  **CD009B1_M42**  **CD009B1_M103**  **CD009B1_M33**  **CD009B1_M43**  **CD009B1_M108**  **CD009B1_M34**  **CD009B1_M117**  **CD009B1_M46**  **CD009B1_M51**  **CD009B1_M11**  **CD009B1_M6**  **CD009B1_M8**  **CD009B1_M22**  **CD009B1M9_1uL CD009B1M12_1uL CD009B1M37_1uL CD009B1M5_1uL CD009B1M32**  **CD009B1M35**  **CD009B1M2**  **CD009B1M4**  **CD009B1M41**  **CD009B1M14**  **CD009B1M44**  **CD009B1M16**  **CD009B1M3** | **D7S481**  **D7S481**  **D7S481**  **D7S481**  **D7S481**  **D7S481**  **D7S481**  **D7S481**  **D7S481**  **D7S481**  **D7S481**  **D7S481**  **D7S481**  **D7S481**  **D7S481**  **D7S481**  **D7S481**  **D7S481**  **D7S481**  **D7S481**  **D7S481**  **D7S481**  **D7S481**  **D7S481**  **D7S481**  **D7S481**  **D7S481**  **D7S481**  **D7S481**  **D7S481**  **D7S481** | **203**  **203**  **203**  **203**  **203**  **203**  **203**  **203**  **203**  **203**  **203**  **203**  **203**  **203**  **203**  **203**  **203**  **203**  **203**  **203**  **203**  **203**  **203**  **203**  **203**  **203**  **203**  **203**  **203**  **203**  **203** |  | **203.1**  **202.7**  **203.0**  **202.6**  **203.1**  **202.7**  **202.8**  **202.7**  **202.8**  **202.7**  **202.8**  **202.8**  **202.7**  **202.8**  **202.8**  **202.8**  **202.7**  **202.8**  **202.8**  **202.7**  **202.6**  **202.9**  **202.8**  **202.7**  **203.1**  **202.7**  **202.8**  **202.7**  **202.8**  **202.7**  **202.7** |  | **186**  **1331**  **536**  **1547**  **684**  **1872**  **856**  **1734**  **1745**  **531**  **1427**  **1265**  **263**  **1946**  **981**  **970**  **1896**  **1561**  **172**  **203**  **99**  **379**  **486**  **61**  **637**  **397**  **379**  **138**  **190**  **590**  **703** |  | **1809**  **11767**  **4788**  **13464**  **6358**  **17032**  **7564**  **16276**  **15679**  **4751**  **12746**  **11441**  **2243**  **17974**  **8773**  **8815**  **17181**  **13782**  **1388**  **1852**  **752**  **3315**  **4316**  **593**  **6281**  **3511**  **3546**  **1223**  **1765**  **5299**  **6401** |  | **4462**  **4253**  **4437**  **4225**  **4421**  **4315**  **4268**  **4315**  **4277**  **4289**  **4289**  **4264**  **4241**  **4291**  **4282**  **4315**  **4299**  **4253**  **4379**  **4393**  **4359**  **4407**  **4283**  **4287**  **4566**  **4344**  **4390**  **4351**  **4396**  **4355**  **4366** |  |
| **1**  **2**  **3** | **1**  **2**  **3**  **4**  **5**  **6**  **7**  **8**  **9**  **10**  **11**  **12**  **13** | **7/7/05**  **7/7/05**  **7/7/05**  **7/7/05**  **7/7/05**  **7/15/05**  **7/15/05**  **7/15/05**  **7/15/05**  **7/15/05**  **7/18/05**  **7/18/05**  **7/18/05** | **CD010B1_2M15**  **CD010B1_2M18**  **CD010B1_2M20**  **CD010B1_2M1**  **CD010B1_2M58**  **CD010B1_2M16**  **CD010B1_2M28**  **CD010B1_2M33**  **CD010B1_2M10**  **CD010B1_2M48**  **CD010B1_2M52**  **CD010B1_2M53**  **CD010B1_2M56** | **BAT25**  **BAT25**  **BAT25**  **BAT25**  **BAT25**  **BAT25**  **BAT25**  **BAT25**  **BAT25**  **BAT25**  **BAT25**  **BAT25**  **BAT25** | **126**  **126**  **126**  **126**  **126**  **127**  **127**  **127**  **127**  **127**  **127**  **127**  **127** | **127**  **127**  **127**  **127**  **127** | **125.7**  **125.7**  **125.7**  **125.6**  **125.7**  **126.9**  **126.8**  **126.8**  **126.9**  **126.9**  **126.9**  **126.9**  **126.9** | **126.7**  **126.8**  **126.8**  **126.6**  **126.7** | **969**  **910**  **1634**  **896**  **873**  **1312**  **1537**  **562**  **2757**  **2362**  **1385**  **1842**  **3766** | **1010**  **907**  **1718**  **941**  **843** | **7987**  **7508**  **12391**  **7436**  **7189**  **10693**  **11978**  **4586**  **22738**  **17967**  **9920**  **13280**  **28556** | **8192**  **7216**  **13537**  **7687**  **6548** | **3364**  **3342**  **3379**  **3325**  **3324**  **3425**  **3405**  **3451**  **3463**  **3446**  **3567**  **3557**  **3528** | **3377**  **3356**  **3393**  **3338**  **3337** |
| **1** | **1**  **2**  **3** | **7/7/05**  **7/7/05**  **7/7/05** | **CD010B1_2M15**  **CD010B1_2M18**  **CD010B1_2M20** | **BAT26**  **BAT26**  **BAT26** | **121**  **121**  **121** |  | **120.7**  **120.7**  **120.7** |  | **272**  **358**  **466** |  | **2514**  **3177**  **4082** |  | **3299**  **3278**  **3314** |  |

| **Day** | **Count** | **Run Date** | **Sample Name** | **Marker** | **Allele 1** | **Allele 2** | **Size 1** | **Size 2** | **Height 1** | **Height 2** | **Peak Area 1** | **Peak Area 2** | **Data Point 1** | **Data Point 2** |
| --- | --- | --- | --- | --- | --- | --- | --- | --- | --- | --- | --- | --- | --- | --- |
| **2**  **3** | **4**  **5**  **6**  **7**  **8**  **9**  **10**  **11**  **12**  **13** | **7/7/05**  **7/7/05**  **7/15/05**  **7/15/05**  **7/15/05**  **7/15/05**  **7/15/05**  **7/18/05**  **7/18/05**  **7/18/05** | **CD010B1_2M1**  **CD010B1_2M58**  **CD010B1_2M16**  **CD010B1_2M28**  **CD010B1_2M33**  **CD010B1_2M10**  **CD010B1_2M48**  **CD010B1_2M52**  **CD010B1_2M53**  **CD010B1_2M56** | **BAT26**  **BAT26**  **BAT26**  **BAT26**  **BAT26**  **BAT26**  **BAT26**  **BAT26**  **BAT26**  **BAT26** | **121**  **121**  **121**  **121**  **121**  **121**  **121**  **121**  **121**  **121** |  | **120.6**  **120.6**  **120.8**  **120.7**  **120.8**  **120.9**  **120.9**  **120.9**  **120.8**  **120.9** |  | **272**  **326**  **293**  **653**  **182**  **852**  **717**  **558**  **777**  **1420** |  | **2565**  **2846**  **2733**  **6105**  **1574**  **7806**  **6152**  **5271**  **7388**  **14685** |  | **3261**  **3260**  **3347**  **3327**  **3372**  **3385**  **3368**  **3488**  **3477**  **3449** |  |
| **1**  **2**  **3** | **1**  **2**  **3**  **4**  **5**  **6**  **7**  **8**  **9**  **10**  **11**  **12**  **13** | **7/7/05**  **7/7/05**  **7/7/05**  **7/7/05**  **7/7/05**  **7/15/05**  **7/15/05**  **7/15/05**  **7/15/05**  **7/15/05**  **7/18/05**  **7/18/05**  **7/18/05** | **CD010B1_2M15**  **CD010B1_2M18**  **CD010B1_2M20**  **CD010B1_2M1**  **CD010B1_2M58**  **CD010B1_2M16**  **CD010B1_2M28**  **CD010B1_2M33**  **CD010B1_2M10**  **CD010B1_2M48**  **CD010B1_2M52**  **CD010B1_2M53**  **CD010B1_2M56** | **D3S3623**  **D3S3623**  **D3S3623**  **D3S3623**  **D3S3623**  **D3S3623**  **D3S3623**  **D3S3623**  **D3S3623**  **D3S3623**  **D3S3623**  **D3S3623**  **D3S3623** | **219**  **219**  **219**  **219**  **219**  **219**  **219**  **219**  **219**  **219**  **219**  **219**  **219** | **225**  **225**  **225**  **225**  **225**  **225**  **225**  **225**  **225**  **225**  **225**  **225**  **225** | **218.5**  **218.4**  **218.6**  **218.5**  **218.5**  **218.5**  **218.4**  **218.5**  **218.6**  **218.4**  **218.5**  **218.5**  **218.6** | **224.8**  **224.7**  **224.7**  **224.7**  **224.7**  **224.7**  **224.7**  **224.7**  **224.8**  **224.7**  **224.7**  **224.7**  **224.8** | **217**  **398**  **656**  **523**  **409**  **428**  **309**  **207**  **1080**  **1739**  **786**  **967**  **1778** | **136**  **467**  **314**  **471**  **314**  **329**  **240**  **162**  **771**  **1284**  **520**  **743**  **1326** | **1912**  **3361**  **5826**  **4481**  **3371**  **3705**  **2632**  **1723**  **9949**  **15275**  **6654**  **8619**  **15880** | **1218**  **4083**  **2662**  **3935**  **2620**  **2752**  **1977**  **1405**  **6685**  **10907**  **4563**  **6505**  **11543** | **4486**  **4450**  **4507**  **4429**  **4424**  **4534**  **4510**  **4572**  **4581**  **4563**  **4704**  **4691**  **4657** | **4557**  **4520**  **4577**  **4498**  **4493**  **4605**  **4581**  **4644**  **4652**  **4635**  **4776**  **4763**  **4729** |
| **1**  **2**  **3** | **1**  **2**  **3**  **4**  **5**  **6**  **7**  **8**  **9**  **10**  **11**  **12**  **13** | **7/7/05**  **7/7/05**  **7/7/05**  **7/7/05**  **7/7/05**  **7/15/05**  **7/15/05**  **7/15/05**  **7/15/05**  **7/15/05**  **7/18/05**  **7/18/05**  **7/18/05** | **CD010B1_2M15**  **CD010B1_2M18* CD010B1_2M20**  **CD010B1_2M1* CD010B1_2M58**  **CD010B1_2M16**  **CD010B1_2M28**  **CD010B1_2M33**  **CD010B1_2M10**  **CD010B1_2M48**  **CD010B1_2M52**  **CD010B1_2M53**  **CD010B1_2M56** | **D5S346**  **D5S346* D5S346**  **D5S346* D5S346**  **D5S346**  **D5S346**  **D5S346**  **D5S346**  **D5S346**  **D5S346**  **D5S346**  **D5S346** | **89**  **92**  **89**  **89**  **89**  **89**  **89**  **89**  **89**  **89**  **89**  **89**  **89** | **92**  **92**  **92**  **92**  **92**  **92**  **92**  **92**  **92**  **92**  **92**  **92** | **89.5**  **91.6**  **89.4**  **89.4**  **89.4**  **89.4**  **89.4**  **89.5**  **89.5**  **89.4**  **89.5**  **89.5**  **89.5** | **91.7**  **91.6**  **91.6**  **91.5**  **91.6**  **91.6**  **91.7**  **91.7**  **91.7**  **91.7**  **91.7**  **91.7** | **315**  **583**  **1388**  **604**  **1287**  **1632**  **1437**  **571**  **2275**  **1901**  **1288**  **1905**  **4104** | **304**  **1011**  **603**  **814**  **1167**  **1089**  **422**  **1631**  **1438**  **804**  **1449**  **3100** | **2353**  **4145**  **10614**  **4459**  **9778**  **12343**  **11292**  **4425**  **17657**  **14798**  **10258**  **15072**  **31729** | **2255**  **7642**  **4357**  **5834**  **8415**  **8066**  **3185**  **12038**  **10718**  **5989**  **10887**  **22915** | **2885**  **2897**  **2899**  **2854**  **2853**  **2931**  **2913**  **2953**  **2966**  **2949**  **3062**  **3054**  **3027** | **2914**  **2928**  **2883**  **2881**  **2960**  **2942**  **2983**  **2995**  **2979**  **3092**  **3083**  **3057** |
| **1**  **2**  **3** | **1**  **2**  **3**  **4**  **5**  **6**  **7**  **8**  **9**  **10**  **11**  **12**  **13** | **7/7/05**  **7/7/05**  **7/7/05**  **7/7/05**  **7/7/05**  **7/15/05**  **7/15/05**  **7/15/05**  **7/15/05**  **7/15/05**  **7/18/05**  **7/18/05**  **7/18/05** | **CD010B1_2M15* CD010B1_2M18**  **CD010B1_2M20**  **CD010B1_2M1**  **CD010B1_2M58**  **CD010B1_2M16**  **CD010B1_2M28**  **CD010B1_2M33**  **CD010B1_2M10**  **CD010B1_2M48**  **CD010B1_2M52**  **CD010B1_2M53**  **CD010B1_2M56** | **D6S262* D6S262**  **D6S262**  **D6S262**  **D6S262**  **D6S262**  **D6S262**  **D6S262**  **D6S262**  **D6S262**  **D6S262**  **D6S262**  **D6S262** | **176**  **172**  **172**  **172**  **172**  **172**  **172**  **172**  **172**  **172**  **172**  **172**  **172** | **176**  **176**  **176**  **176**  **176**  **176**  **176**  **176**  **176**  **176**  **176**  **176** | **176.1**  **172.2**  **172.2**  **172.2**  **172.2**  **172.2**  **172.2**  **172.3**  **172.3**  **172.2**  **172.3**  **172.2**  **172.2** | **176.1**  **176.1**  **176.1**  **176.0**  **176.1**  **176.1**  **176.1**  **176.1**  **176.1**  **176.2**  **176.1**  **176.2** | **605**  **464**  **726**  **515**  **277**  **877**  **652**  **335**  **1187**  **1876**  **880**  **1543**  **2828** | **682**  **414**  **420**  **298**  **715**  **535**  **281**  **878**  **1510**  **710**  **1226**  **2271** | **5298**  **3747**  **6019**  **4962**  **2009**  **6932**  **5330**  **2507**  **9779**  **15116**  **7398**  **12525**  **24805** | **5922**  **3571**  **3699**  **2421**  **5721**  **4339**  **2205**  **7855**  **12219**  **6285**  **11127**  **20462** | **3978**  **3901**  **3947**  **3882**  **3879**  **3977**  **3955**  **4010**  **4020**  **4002**  **4133**  **4122**  **4090** | **3949**  **3995**  **3930**  **3926**  **4026**  **4004**  **4058**  **4068**  **4051**  **4183**  **4171**  **4140** |
| **1**  **2** | **1**  **2**  **3**  **4**  **5**  **6**  **7**  **8**  **9** | **7/7/05**  **7/7/05**  **7/7/05**  **7/7/05**  **7/7/05**  **7/15/05**  **7/15/05**  **7/15/05**  **7/15/05** | **CD010B1_2M15**  **CD010B1_2M18**  **CD010B1_2M20**  **CD010B1_2M1**  **CD010B1_2M58**  **CD010B1_2M16**  **CD010B1_2M28**  **CD010B1_2M33**  **CD010B1_2M10** | **D7S481**  **D7S481**  **D7S481**  **D7S481**  **D7S481**  **D7S481**  **D7S481**  **D7S481**  **D7S481** | **187**  **187**  **187**  **187**  **187**  **187**  **187**  **187**  **187** | **199**  **199**  **199**  **199**  **199**  **199**  **199**  **199**  **199** | **187.1**  **187.2**  **187.2**  **187.2**  **187.0**  **187.2**  **187.2**  **187.2**  **187.2** | **198.7**  **198.6**  **198.6**  **198.7**  **198.5**  **198.8**  **198.7**  **198.7**  **198.7** | **256**  **326**  **166**  **289**  **142**  **112**  **240**  **93**  **545** | **75**  **262**  **154**  **122**  **60**  **74**  **133**  **59**  **336** | **2430**  **3008**  **1461**  **2592**  **1302**  **909**  **2190**  **819**  **5135** | **685**  **2398**  **1187**  **1197**  **539**  **699**  **1168**  **499**  **3038** | **4116**  **4086**  **4135**  **4066**  **4061**  **4164**  **4142**  **4198**  **4208** | **4261**  **4227**  **4279**  **4207**  **4201**  **4309**  **4285**  **4343**  **4352** |

| **Day** | **Count** | **Run Date** | **Sample Name** | **Marker** | **Allele 1** | **Allele 2** | **Size 1** | **Size 2** | **Height 1** | **Height 2** | **Peak Area 1** | **Peak Area 2** | **Data Point 1** | **Data Point 2** |
| --- | --- | --- | --- | --- | --- | --- | --- | --- | --- | --- | --- | --- | --- | --- |
| **3** | **10**  **11**  **12**  **13** | **7/15/05**  **7/18/05**  **7/18/05**  **7/18/05** | **CD010B1_2M48**  **CD010B1_2M52**  **CD010B1_2M53**  **CD010B1_2M56** | **D7S481**  **D7S481**  **D7S481**  **D7S481** | **187**  **187**  **187**  **187** | **199**  **199**  **199**  **199** | **187.2**  **187.3**  **187.2**  **187.3** | **198.7**  **198.8**  **198.7**  **198.7** | **649**  **383**  **332**  **663** | **453**  **208**  **248**  **447** | **5698**  **3620**  **3165**  **6323** | **4010**  **1899**  **2352**  **4248** | **4191**  **4325**  **4313**  **4281** | **4335**  **4472**  **4459**  **4426** |
| **1** | **1**  **1**  **1**  **1**  **1**  **1** | **11/14/05**  **11/14/05**  **11/14/05**  **11/14/05**  **11/14/05**  **11/14/05** | **CD0011B1M1**  **CD0011B1M1**  **CD0011B1M1**  **CD0011B1M1**  **CD0011B1M1**  **CD0011B1M1** | **BAT25**  **BAT26**  **D3S3623**  **D5S346**  **D6S262**  **D7S481** | **127**  **122**  **225**  **94**  **174**  **203** | **128**  **96**  **184** | **126.9**  **121.7**  **224.8**  **93.9**  **174.2**  **202.7** | **127.8**  **96.1**  **183.9** | **3284**  **1233**  **5174**  **3029**  **2967**  **1237** | **3372**  **1917**  **2085** | **27314**  **14245**  **58129**  **24189**  **33699**  **15439** | **27324**  **13721**  **22574** | **3663**  **3593**  **4897**  **3207**  **4265**  **4635** | **3676**  **3237**  **4392** |
| **1**  **2**  **3** | **1**  **2**  **3**  **4**  **5**  **6**  **7**  **8**  **9**  **10**  **11**  **12**  **13**  **14**  **15**  **16**  **17**  **18**  **19**  **20**  **21**  **22**  **23** | **7/8/05**  **7/8/05**  **7/8/05**  **7/8/05**  **7/8/05**  **7/8/05**  **7/8/05**  **7/8/05**  **7/13/05**  **7/18/05**  **7/18/05**  **7/18/05**  **7/18/05**  **7/18/05**  **7/18/05**  **7/18/05**  **7/18/05**  **7/18/05**  **7/18/05**  **7/18/05**  **7/18/05**  **7/18/05**  **7/18/05** | **CD0013B1M5_1UL**  **0013B1M10_.4UL**  **0013B1M10_1UL**  **0013B1M18_1UL CD013B1_M1**  **CD013B1_M4**  **CD013B1_M24**  **CD013B1_M35**  **CD013B1M15_1uL CD013B1M41**  **CD013B1M55**  **CD013B1M17**  **CD013B1M30**  **CD013B1M57**  **CD013B1M46**  **CD013B1M58**  **CD013B1M20**  **CD013B1M33**  **CD013B1M47**  **CD013B1M21**  **CD013B1M34**  **CD013B1M36**  **CD013B1M49** | **BAT25**  **BAT25**  **BAT25**  **BAT25**  **BAT25**  **BAT25**  **BAT25**  **BAT25**  **BAT25**  **BAT25**  **BAT25**  **BAT25**  **BAT25**  **BAT25**  **BAT25**  **BAT25**  **BAT25**  **BAT25**  **BAT25**  **BAT25**  **BAT25**  **BAT25**  **BAT25** | **128**  **128**  **128**  **129**  **129**  **129**  **128**  **128**  **128**  **128**  **128**  **129**  **128**  **129**  **128**  **129**  **128**  **128**  **128**  **128**  **128**  **128**  **128** | **129**  **129**  **129**  **129**  **129**  **129**  **129**  **129**  **129**  **129**  **129**  **129**  **129**  **129** | **127.9**  **127.9**  **127.8**  **128.9**  **129.0**  **129.1**  **127.9**  **128.0**  **127.8**  **127.8**  **127.8**  **128.9**  **127.9**  **128.9**  **127.9**  **128.9**  **127.9**  **127.9**  **127.9**  **128.0**  **127.9**  **127.9**  **127.9** | **128.9**  **128.8**  **129.0**  **128.8**  **128.9**  **128.9**  **128.9**  **128.9**  **128.9**  **128.9**  **128.9**  **129.0**  **129.0**  **129.0** | **1633**  **908**  **2462**  **4375**  **2500**  **2565**  **1993**  **686**  **2006**  **1406**  **1139**  **2001**  **1389**  **1313**  **2570**  **1400**  **1799**  **2987**  **1817**  **5093**  **2804**  **1193**  **2573** | **933**  **2578**  **711**  **2090**  **1383**  **1137**  **1402**  **2578**  **1796**  **3069**  **1865**  **5232**  **2716**  **2612** | **10997**  **6219**  **17053**  **32354**  **19011**  **19664**  **15286**  **4841**  **14673**  **10189**  **8334**  **15400**  **10120**  **8869**  **18124**  **8964**  **14276**  **22152**  **13173**  **38142**  **20453**  **8442**  **17847** | **6120**  **17597**  **4734**  **15721**  **9296**  **8030**  **10016**  **17918**  **13972**  **24025**  **13637**  **38742**  **18878**  **18229** | **3449**  **3450**  **3425**  **3463**  **3477**  **3485**  **3428**  **3454**  **3433**  **3537**  **3519**  **3507**  **3533**  **3566**  **3538**  **3551**  **3464**  **3486**  **3511**  **3480**  **3514**  **3548**  **3558** | **3463**  **3438**  **3467**  **3446**  **3551**  **3533**  **3546**  **3551**  **3477**  **3499**  **3524**  **3493**  **3528**  **3572** |
| **1**  **2**  **3** | **1**  **2**  **3**  **4**  **5**  **6**  **7**  **8**  **9**  **10**  **11**  **12**  **13**  **14**  **15**  **16** | **7/8/05**  **7/8/05**  **7/8/05**  **7/8/05**  **7/8/05**  **7/8/05**  **7/8/05**  **7/8/05**  **7/13/05**  **7/18/05**  **7/18/05**  **7/18/05**  **7/18/05**  **7/18/05**  **7/18/05**  **7/18/05** | **CD0013B1M5_1UL**  **0013B1M10_.4UL**  **0013B1M10_1UL**  **0013B1M18_1UL CD013B1_M1**  **CD013B1_M4**  **CD013B1_M24**  **CD013B1_M35**  **CD013B1M15_1uL CD013B1M41**  **CD013B1M55**  **CD013B1M17**  **CD013B1M30**  **CD013B1M57**  **CD013B1M46**  **CD013B1M58** | **BAT26**  **BAT26**  **BAT26**  **BAT26**  **BAT26**  **BAT26**  **BAT26**  **BAT26**  **BAT26**  **BAT26**  **BAT26**  **BAT26**  **BAT26**  **BAT26**  **BAT26**  **BAT26** | **122**  **122**  **122**  **122**  **122**  **122**  **122**  **122**  **122**  **122**  **122**  **122**  **122**  **122**  **122**  **122** | **123** | **121.7**  **121.8**  **121.7**  **121.8**  **122.0**  **122.0**  **121.9**  **121.9**  **121.7**  **121.7**  **121.7**  **121.8**  **121.9**  **121.8**  **121.8**  **121.8** | **122.7** | **908**  **415**  **1160**  **1463**  **775**  **887**  **717**  **308**  **838**  **767**  **630**  **692**  **582**  **612**  **1547**  **582** | **554** | **8099**  **3880**  **10311**  **14393**  **7084**  **7973**  **6751**  **2583**  **8003**  **6449**  **5723**  **6489**  **5337**  **5150**  **13152**  **4800** | **4068** | **3370**  **3371**  **3346**  **3371**  **3386**  **3393**  **3349**  **3375**  **3355**  **3457**  **3439**  **3415**  **3453**  **3472**  **3458**  **3457** | **3470** |
|  | **17**  **18**  **19**  **20**  **21**  **22**  **23** | **7/18/05**  **7/18/05**  **7/18/05**  **7/18/05**  **7/18/05**  **7/18/05**  **7/18/05** | **CD013B1M20**  **CD013B1M33**  **CD013B1M47**  **CD013B1M21**  **CD013B1M34**  **CD013B1M36**  **CD013B1M49** | **BAT26**  **BAT26**  **BAT26**  **BAT26**  **BAT26**  **BAT26**  **BAT26** | **122**  **122**  **122**  **122**  **122**  **122**  **122** |  | **121.7**  **121.8**  **121.8**  **121.9**  **121.9**  **121.8**  **121.8** |  | **835**  **1424**  **790**  **2303**  **1447**  **598**  **1366** |  | **8525**  **12932**  **7491**  **21818**  **12311**  **5242**  **12306** |  | **3384**  **3407**  **3431**  **3401**  **3435**  **3468**  **3478** |  |
| **1** | **1** | **7/8/05** | **CD0013B1M5_1UL*** | **D3S3623*** | **219** |  | **218.4** |  | **1030** |  | **8668** |  | **4546** |  |

| **Day** | **Count** | **Run Date** | **Sample Name** | **Marker** | **Allele 1** | **Allele 2** | **Size 1** | **Size 2** | **Height 1** | **Height 2** | **Peak Area 1** | **Peak Area 2** | **Data Point 1** | **Data Point 2** |
| --- | --- | --- | --- | --- | --- | --- | --- | --- | --- | --- | --- | --- | --- | --- |
| **2**  **3** | **2**  **3**  **4**  **5**  **6**  **7**  **8**  **9**  **10**  **11**  **12**  **13**  **14**  **15**  **16**  **17**  **18**  **19**  **20**  **21**  **22**  **23** | **7/8/05**  **7/8/05**  **7/8/05**  **7/8/05**  **7/8/05**  **7/8/05**  **7/8/05**  **7/13/05**  **7/18/05**  **7/18/05**  **7/18/05**  **7/18/05**  **7/18/05**  **7/18/05**  **7/18/05**  **7/18/05**  **7/18/05**  **7/18/05**  **7/18/05**  **7/18/05**  **7/18/05**  **7/18/05** | **0013B1M10_.4UL***  **0013B1M10_1UL**  **0013B1M18_1UL CD013B1_M1**  **CD013B1_M4**  **CD013B1_M24**  **CD013B1_M35**  **CD013B1M15_1uL CD013B1M41**  **CD013B1M55**  **CD013B1M17**  **CD013B1M30**  **CD013B1M57**  **CD013B1M46**  **CD013B1M58**  **CD013B1M20**  **CD013B1M33**  **CD013B1M47**  **CD013B1M21**  **CD013B1M34**  **CD013B1M36**  **CD013B1M49** | **D3S3623* D3S3623**  **D3S3623**  **D3S3623**  **D3S3623**  **D3S3623**  **D3S3623**  **D3S3623**  **D3S3623**  **D3S3623**  **D3S3623**  **D3S3623**  **D3S3623**  **D3S3623**  **D3S3623**  **D3S3623**  **D3S3623**  **D3S3623**  **D3S3623**  **D3S3623**  **D3S3623**  **D3S3623** | **223**  **219**  **219**  **219**  **219**  **219**  **219**  **219**  **219**  **219**  **219**  **219**  **219**  **219**  **219**  **219**  **219**  **219**  **219**  **219**  **219**  **219** | **223**  **223**  **223**  **223**  **223**  **223**  **223**  **223**  **223**  **223**  **223**  **223**  **223**  **223**  **223**  **223**  **223**  **223**  **223**  **223**  **223** | **222.7**  **218.5**  **218.5**  **218.7**  **218.8**  **218.7**  **218.7**  **218.4**  **218.4**  **218.4**  **218.6**  **218.4**  **218.4**  **218.4**  **218.3**  **218.6**  **218.4**  **218.4**  **218.5**  **218.5**  **218.4**  **218.4** | **222.6**  **222.7**  **223.0**  **223.1**  **222.8**  **223.0**  **222.7**  **222.6**  **222.6**  **222.7**  **222.6**  **222.6**  **222.6**  **222.5**  **222.7**  **222.6**  **222.5**  **222.7**  **222.6**  **222.6**  **222.5** | **621**  **1416**  **2532**  **1266**  **1740**  **796**  **600**  **1169**  **353**  **408**  **1424**  **738**  **200**  **359**  **706**  **327**  **655**  **880**  **1060**  **490**  **759**  **552** | **993**  **1954**  **1001**  **1378**  **690**  **382**  **837**  **225**  **400**  **1055**  **644**  **191**  **277**  **564**  **251**  **484**  **745**  **749**  **345**  **678**  **379** | **5477**  **11993**  **21432**  **10763**  **14966**  **6675**  **4999**  **9682**  **2987**  **3462**  **12218**  **6186**  **1629**  **2995**  **5962**  **2960**  **5559**  **7402**  **9011**  **4229**  **6423**  **4694** | **8350**  **16374**  **8069**  **11695**  **5866**  **3207**  **6760**  **1839**  **3391**  **8638**  **5329**  **1679**  **2195**  **4493**  **2115**  **3925**  **6251**  **6250**  **2785**  **5632**  **2956** | **4597**  **4518**  **4548**  **4569**  **4584**  **4537**  **4560**  **4522**  **4657**  **4630**  **4602**  **4653**  **4678**  **4656**  **4659**  **4563**  **4593**  **4620**  **4586**  **4633**  **4670**  **4682** | **4565**  **4596**  **4618**  **4633**  **4585**  **4609**  **4570**  **4706**  **4679**  **4650**  **4702**  **4728**  **4705**  **4708**  **4610**  **4642**  **4667**  **4635**  **4681**  **4720**  **4730** |
| **1**  **2**  **3** | **1**  **2**  **3**  **4**  **5**  **6**  **7**  **8**  **9**  **10**  **11**  **12**  **13**  **14**  **15**  **16**  **17**  **18**  **19**  **20**  **21**  **22**  **23** | **7/8/05**  **7/8/05**  **7/8/05**  **7/8/05**  **7/8/05**  **7/8/05**  **7/8/05**  **7/8/05**  **7/13/05**  **7/18/05**  **7/18/05**  **7/18/05**  **7/18/05**  **7/18/05**  **7/18/05**  **7/18/05**  **7/18/05**  **7/18/05**  **7/18/05**  **7/18/05**  **7/18/05**  **7/18/05**  **7/18/05** | **CD0013B1M5_1UL***  **0013B1M10_.4UL**  **0013B1M10_1UL**  **0013B1M18_1UL CD013B1_M1**  **CD013B1_M4**  **CD013B1_M24**  **CD013B1_M35**  **CD013B1M15_1uL CD013B1M41**  **CD013B1M55**  **CD013B1M17**  **CD013B1M30**  **CD013B1M57**  **CD013B1M46**  **CD013B1M58**  **CD013B1M20**  **CD013B1M33**  **CD013B1M47**  **CD013B1M21**  **CD013B1M34**  **CD013B1M36**  **CD013B1M49** | **D5S346* D5S346**  **D5S346**  **D5S346**  **D5S346**  **D5S346**  **D5S346**  **D5S346**  **D5S346**  **D5S346**  **D5S346**  **D5S346**  **D5S346**  **D5S346**  **D5S346**  **D5S346**  **D5S346**  **D5S346**  **D5S346**  **D5S346**  **D5S346**  **D5S346**  **D5S346** | **103**  **103**  **103**  **103**  **103**  **103**  **103**  **103**  **103**  **103**  **103**  **103**  **103**  **103**  **103**  **103**  **103**  **103**  **103**  **103**  **103**  **103**  **103** | **107**  **107**  **107**  **107**  **107**  **107**  **107**  **107**  **107**  **107**  **107**  **107**  **107**  **107**  **107**  **107**  **107**  **107**  **107**  **107**  **107**  **107**  **107** | **102.6**  **102.6**  **102.6**  **102.6**  **102.8**  **102.7**  **102.7**  **102.8**  **102.6**  **102.6**  **102.5**  **102.6**  **102.7**  **102.6**  **102.6**  **102.6**  **102.5**  **102.6**  **102.5**  **102.7**  **102.6**  **102.6**  **102.6** | **106.7**  **106.7**  **106.7**  **106.7**  **106.8**  **106.9**  **106.9**  **106.9**  **106.7**  **106.7**  **106.7**  **106.7**  **106.8**  **106.8**  **106.7**  **106.7**  **106.7**  **106.8**  **106.7**  **106.8**  **106.7**  **106.7**  **106.7** | **646**  **497**  **2007**  **2909**  **1536**  **1359**  **1335**  **401**  **1346**  **1527**  **930**  **1034**  **917**  **1019**  **2704**  **1145**  **1390**  **1633**  **919**  **2903**  **2117**  **743**  **2381** | **760**  **338**  **1419**  **2284**  **1127**  **1007**  **856**  **338**  **1001**  **932**  **581**  **813**  **657**  **867**  **1808**  **905**  **962**  **1205**  **732**  **2014**  **1534**  **547**  **1802** | **5170**  **4060**  **16125**  **23503**  **12290**  **10453**  **10460**  **3104**  **11297**  **12234**  **7941**  **8414**  **7463**  **8552**  **21915**  **9373**  **11942**  **14070**  **7847**  **23623**  **17418**  **6119**  **19946** | **6032**  **2721**  **11572**  **18058**  **8945**  **7736**  **6656**  **2647**  **8141**  **7491**  **4679**  **6514**  **5409**  **7237**  **14344**  **7338**  **7659**  **9851**  **6019**  **16097**  **12533**  **4306**  **14715** | **3114**  **3114**  **3091**  **3115**  **3128**  **3134**  **3092**  **3118**  **3101**  **3196**  **3180**  **3156**  **3192**  **3210**  **3197**  **3196**  **3128**  **3149**  **3172**  **3143**  **3174**  **3206**  **3215** | **3170**  **3170**  **3147**  **3171**  **3184**  **3191**  **3149**  **3174**  **3157**  **3254**  **3238**  **3213**  **3249**  **3268**  **3254**  **3253**  **3185**  **3206**  **3229**  **3200**  **3231**  **3263**  **3273** |
| **1**  **2**  **3** | **1**  **2**  **3**  **4**  **5**  **6**  **7**  **8**  **9**  **10**  **11**  **12**  **13** | **7/8/05**  **7/8/05**  **7/8/05**  **7/8/05**  **7/8/05**  **7/8/05**  **7/8/05**  **7/8/05**  **7/13/05**  **7/18/05**  **7/18/05**  **7/18/05**  **7/18/05** | **CD0013B1M5_1UL**  **0013B1M10_.4UL**  **0013B1M10_1UL**  **0013B1M18_1UL CD013B1_M1**  **CD013B1_M4**  **CD013B1_M24**  **CD013B1_M35**  **CD013B1M15_1uL CD013B1M41**  **CD013B1M55**  **CD013B1M17**  **CD013B1M30** | **D6S262**  **D6S262**  **D6S262**  **D6S262**  **D6S262**  **D6S262**  **D6S262**  **D6S262**  **D6S262**  **D6S262**  **D6S262**  **D6S262**  **D6S262** | **172**  **172**  **172**  **172**  **172**  **172**  **172**  **172**  **172**  **172**  **172**  **172**  **172** | **182**  **182**  **182**  **182**  **182**  **182**  **182**  **182**  **182**  **182**  **182**  **182**  **182** | **172.3**  **172.3**  **172.3**  **172.2**  **172.4**  **172.4**  **172.4**  **172.3**  **172.3**  **172.3**  **172.2**  **172.3**  **172.3** | **181.9**  **181.9**  **182.0**  **182.0**  **182.1**  **182.1**  **182.1**  **182.0**  **182.0**  **182.0**  **181.9**  **181.9**  **181.9** | **293**  **416**  **1705**  **3033**  **3306**  **3803**  **2603**  **1356**  **1241**  **261**  **521**  **1271**  **932** | **786**  **125**  **1159**  **1921**  **2068**  **2558**  **1802**  **705**  **849**  **181**  **260**  **717**  **547** | **2309**  **3343**  **13871**  **26120**  **30345**  **35753**  **23798**  **11771**  **10779**  **2032**  **4328**  **10712**  **7549** | **6203**  **904**  **9303**  **16225**  **18785**  **23418**  **15944**  **5869**  **7199**  **1259**  **2069**  **6071**  **4363** | **3989**  **3991**  **3963**  **3990**  **4008**  **4018**  **3973**  **3998**  **3970**  **4088**  **4066**  **4039**  **4084** | **4110**  **4112**  **4084**  **4112**  **4130**  **4141**  **4096**  **4120**  **4090**  **4212**  **4188**  **4161**  **4207** |

| **Day** | **Count** | **Run Date** | **Sample Name** | **Marker** | **Allele 1** | **Allele 2** | **Size 1** | **Size 2** | **Height 1** | **Height 2** | **Peak Area 1** | **Peak Area 2** | **Data Point 1** | **Data Point 2** |
| --- | --- | --- | --- | --- | --- | --- | --- | --- | --- | --- | --- | --- | --- | --- |
|  | **14**  **15**  **16**  **17**  **18**  **19**  **20**  **21**  **22**  **23** | **7/18/05**  **7/18/05**  **7/18/05**  **7/18/05**  **7/18/05**  **7/18/05**  **7/18/05**  **7/18/05**  **7/18/05**  **7/18/05** | **CD013B1M57**  **CD013B1M46**  **CD013B1M58**  **CD013B1M20**  **CD013B1M33**  **CD013B1M47**  **CD013B1M21**  **CD013B1M34**  **CD013B1M36**  **CD013B1M49** | **D6S262**  **D6S262**  **D6S262**  **D6S262**  **D6S262**  **D6S262**  **D6S262**  **D6S262**  **D6S262**  **D6S262** | **172**  **172**  **172**  **172**  **172**  **172**  **172**  **172**  **172**  **172** | **182**  **182**  **182**  **182**  **182**  **182**  **182**  **182**  **182**  **182** | **172.3**  **172.2**  **172.2**  **172.3**  **172.2**  **172.3**  **172.3**  **172.2**  **172.3**  **172.2** | **181.9**  **181.9**  **181.9**  **182.0**  **181.8**  **181.9**  **181.9**  **182.0**  **182.0**  **181.9** | **301**  **1050**  **1062**  **804**  **1258**  **985**  **1652**  **858**  **613**  **764** | **169**  **560**  **722**  **573**  **707**  **695**  **1033**  **477**  **365**  **391** | **2522**  **8297**  **8409**  **6833**  **10728**  **8122**  **13388**  **7088**  **4908**  **6203** | **1335**  **4205**  **5577**  **4521**  **5691**  **5724**  **8130**  **3678**  **2823**  **2958** | **4107**  **4088**  **4089**  **4004**  **4031**  **4057**  **4024**  **4064**  **4100**  **4111** | **4230**  **4212**  **4213**  **4125**  **4153**  **4179**  **4146**  **4188**  **4225**  **4235** |
| **1**  **2**  **3** | **1**  **2**  **3**  **4**  **5**  **7**  **8**  **9**  **10**  **11**  **12**  **13**  **14**  **15**  **16**  **17**  **18**  **19**  **20**  **21**  **22**  **23**  **24** | **7/8/05**  **7/8/05**  **7/8/05**  **7/8/05**  **7/8/05**  **7/8/05**  **7/8/05**  **7/8/05**  **7/13/05**  **7/18/05**  **7/18/05**  **7/18/05**  **7/18/05**  **7/18/05**  **7/18/05**  **7/18/05**  **7/18/05**  **7/18/05**  **7/18/05**  **7/18/05**  **7/18/05**  **7/18/05**  **7/18/05** | **CD0013B1M5_1UL**  **0013B1M10_.4UL**  **0013B1M10_1UL**  **0013B1M18_1UL CD013B1_M1**  **CD013B1_M4**  **CD013B1_M24**  **CD013B1_M35**  **CD013B1M15_1uL CD013B1M41**  **CD013B1M55**  **CD013B1M17**  **CD013B1M30**  **CD013B1M57**  **CD013B1M46**  **CD013B1M58**  **CD013B1M20**  **CD013B1M33**  **CD013B1M47**  **CD013B1M21**  **CD013B1M34**  **CD013B1M36**  **CD013B1M49** | **D7S481**  **D7S481**  **D7S481**  **D7S481**  **D7S481**  **D7S481**  **D7S481**  **D7S481**  **D7S481**  **D7S481**  **D7S481**  **D7S481**  **D7S481**  **D7S481**  **D7S481**  **D7S481**  **D7S481**  **D7S481**  **D7S481**  **D7S481**  **D7S481**  **D7S481**  **D7S481** | **199**  **199**  **199**  **199**  **199**  **199**  **199**  **199**  **199**  **199**  **199**  **199**  **199**  **199**  **199**  **199**  **199**  **199**  **199**  **199**  **199**  **199**  **199** |  | **198.7**  **198.7**  **198.7**  **198.7**  **198.9**  **199.0**  **198.9**  **198.9**  **198.6**  **198.7**  **198.8**  **198.8**  **198.7**  **198.8**  **198.7**  **198.7**  **198.7**  **198.6**  **198.7**  **198.8**  **198.7**  **198.8**  **198.7** |  | **253**  **179**  **336**  **425**  **338**  **517**  **311**  **139**  **255**  **74**  **203**  **340**  **88**  **150**  **161**  **139**  **166**  **224**  **362**  **312**  **221**  **538**  **151** |  | **2140**  **1601**  **2691**  **3863**  **3200**  **5125**  **2971**  **1286**  **2152**  **619**  **1880**  **2881**  **768**  **1289**  **1381**  **1152**  **1439**  **2081**  **3187**  **2858**  **1884**  **4579**  **1320** |  | **4320**  **4322**  **4292**  **4321**  **4341**  **4354**  **4308**  **4332**  **4297**  **4426**  **4402**  **4373**  **4422**  **4446**  **4424**  **4428**  **4335**  **4364**  **4390**  **4358**  **4401**  **4439**  **4450** |  |
| **1** | **1**  **2**  **3**  **4**  **5**  **6**  **7**  **8**  **9**  **10**  **11**  **12**  **13**  **14**  **15**  **16**  **17**  **18**  **19**  **20**  **21**  **22**  **23**  **24** | **7/18/05**  **7/18/05**  **7/18/05**  **7/18/05**  **7/18/05**  **7/18/05**  **7/18/05**  **7/18/05**  **7/18/05**  **7/18/05**  **7/18/05**  **7/18/05**  **7/18/05**  **7/18/05**  **7/18/05**  **7/18/05**  **7/18/05**  **7/18/05**  **7/18/05**  **7/18/05**  **7/18/05**  **7/18/05**  **7/18/05**  **7/18/05** | **CD014B1M3**  **CD014B1M11**  **CD014B1M19**  **CD014B1M12**  **CD014B1M5**  **CD014B1M13**  **CD014B1M7**  **CD014B1M8**  **CD014B1M2**  **CD014B1M10**  **CD014B1M18**  **CD014B1M20**  **CD014B1M21**  **CD014B1M22**  **CD014B1M23**  **CD014B1M24**  **CD014B1M25**  **CD014B1M30**  **CD014B1M31**  **CD014B1M32**  **CD014B1M37**  **CD014B1M44**  **CD014B1M46**  **CD014B1M48** | **BAT25**  **BAT25**  **BAT25**  **BAT25**  **BAT25**  **BAT25**  **BAT25**  **BAT25**  **BAT25**  **BAT25**  **BAT25**  **BAT25**  **BAT25**  **BAT25**  **BAT25**  **BAT25**  **BAT25**  **BAT25**  **BAT25**  **BAT25**  **BAT25**  **BAT25**  **BAT25**  **BAT25** | **127**  **127**  **127**  **127**  **127**  **127**  **127**  **127**  **127**  **127**  **127**  **127**  **127**  **127**  **127**  **127**  **127**  **127**  **127**  **127**  **127**  **127**  **127**  **127** |  | **126.9**  **126.9**  **126.9**  **126.9**  **126.9**  **126.8**  **126.9**  **126.9**  **126.8**  **126.9**  **127.0**  **126.9**  **126.9**  **126.9**  **126.9**  **126.9**  **126.8**  **126.9**  **126.9**  **126.9**  **126.9**  **126.9**  **126.8**  **126.9** |  | **1495**  **3672**  **1222**  **916**  **568**  **1246**  **1286**  **1226**  **2927**  **3284**  **1107**  **2845**  **2621**  **638**  **1895**  **703**  **1014**  **2064**  **2387**  **582**  **1017**  **1598**  **905**  **801** |  | **10895**  **28001**  **9216**  **6925**  **4660**  **9993**  **9519**  **9115**  **21571**  **24161**  **9401**  **21415**  **20657**  **5294**  **14522**  **5708**  **8286**  **15835**  **18050**  **4521**  **8255**  **12403**  **7061**  **6369** |  | **3568**  **3368**  **3407**  **3363**  **3527**  **3398**  **3541**  **3526**  **3525**  **3528**  **3438**  **3386**  **3391**  **3408**  **3405**  **3384**  **3398**  **3412**  **3401**  **3380**  **3393**  **3406**  **3400**  **3380** |  |

| **Day** | **Count** | **Run Date** | **Sample Name** | **Marker** | **Allele 1** | **Allele 2** | **Size 1** | **Size 2** | **Height 1** | **Height 2** | **Peak Area 1** | **Peak Area 2** | **Data Point 1** | **Data Point 2** |
| --- | --- | --- | --- | --- | --- | --- | --- | --- | --- | --- | --- | --- | --- | --- |
| **1** | **1**  **2**  **3**  **4**  **5**  **6**  **7**  **8**  **9**  **10**  **11**  **12**  **13**  **14**  **15**  **16**  **17**  **18**  **19**  **20**  **21**  **22**  **23**  **24** | **7/18/05**  **7/18/05**  **7/18/05**  **7/18/05**  **7/18/05**  **7/18/05**  **7/18/05**  **7/18/05**  **7/18/05**  **7/18/05**  **7/18/05**  **7/18/05**  **7/18/05**  **7/18/05**  **7/18/05**  **7/18/05**  **7/18/05**  **7/18/05**  **7/18/05**  **7/18/05**  **7/18/05**  **7/18/05**  **7/18/05**  **7/18/05** | **CD014B1M3**  **CD014B1M11**  **CD014B1M19**  **CD014B1M12**  **CD014B1M5**  **CD014B1M13**  **CD014B1M7**  **CD014B1M8**  **CD014B1M2**  **CD014B1M10**  **CD014B1M18**  **CD014B1M20**  **CD014B1M21**  **CD014B1M22**  **CD014B1M23**  **CD014B1M24**  **CD014B1M25**  **CD014B1M30**  **CD014B1M31**  **CD014B1M32**  **CD014B1M37**  **CD014B1M44**  **CD014B1M46**  **CD014B1M48** | **BAT26**  **BAT26**  **BAT26**  **BAT26**  **BAT26**  **BAT26**  **BAT26**  **BAT26**  **BAT26**  **BAT26**  **BAT26**  **BAT26**  **BAT26**  **BAT26**  **BAT26**  **BAT26**  **BAT26**  **BAT26**  **BAT26**  **BAT26**  **BAT26**  **BAT26**  **BAT26**  **BAT26** | **122**  **122**  **122**  **122**  **122**  **122**  **122**  **122**  **122**  **122**  **122**  **122**  **122**  **122**  **122**  **122**  **122**  **122**  **122**  **122**  **122**  **122**  **122**  **122** |  | **121.8**  **121.9**  **121.7**  **121.8**  **121.8**  **121.7**  **121.8**  **121.9**  **121.8**  **121.9**  **121.9**  **121.8**  **121.8**  **121.9**  **121.8**  **121.9**  **121.7**  **121.8**  **121.8**  **121.8**  **121.8**  **121.8**  **121.8**  **121.7** |  | **595**  **1198**  **330**  **281**  **262**  **663**  **410**  **440**  **1335**  **1211**  **374**  **1170**  **973**  **232**  **724**  **304**  **346**  **620**  **703**  **216**  **293**  **498**  **293**  **312** |  | **5113**  **10906**  **2782**  **2394**  **2245**  **6314**  **3399**  **3584**  **10701**  **9297**  **3641**  **11236**  **9170**  **1898**  **6617**  **2594**  **3100**  **5482**  **6398**  **1841**  **2576**  **4432**  **2554**  **2770** |  | **3501**  **3304**  **3341**  **3298**  **3460**  **3333**  **3474**  **3460**  **3459**  **3462**  **3372**  **3321**  **3326**  **3344**  **3340**  **3320**  **3332**  **3347**  **3336**  **3315**  **3328**  **3341**  **3335**  **3315** |  |
| **1** | **1**  **2**  **3**  **4**  **5**  **6**  **7**  **8**  **9**  **10**  **11**  **12**  **13**  **14**  **15**  **16**  **17**  **18**  **19**  **20**  **21**  **22**  **23**  **24** | **7/18/05**  **7/18/05**  **7/18/05**  **7/18/05**  **7/18/05**  **7/18/05**  **7/18/05**  **7/18/05**  **7/18/05**  **7/18/05**  **7/18/05**  **7/18/05**  **7/18/05**  **7/18/05**  **7/18/05**  **7/18/05**  **7/18/05**  **7/18/05**  **7/18/05**  **7/18/05**  **7/18/05**  **7/18/05**  **7/18/05**  **7/18/05** | **CD014B1M3**  **CD014B1M11**  **CD014B1M19**  **CD014B1M12**  **CD014B1M5**  **CD014B1M13**  **CD014B1M7**  **CD014B1M8**  **CD014B1M2**  **CD014B1M10**  **CD014B1M18**  **CD014B1M20**  **CD014B1M21**  **CD014B1M22**  **CD014B1M23**  **CD014B1M24**  **CD014B1M25**  **CD014B1M30**  **CD014B1M31**  **CD014B1M32**  **CD014B1M37**  **CD014B1M44**  **CD014B1M46**  **CD014B1M48** | **D3S3623**  **D3S3623**  **D3S3623**  **D3S3623**  **D3S3623**  **D3S3623**  **D3S3623**  **D3S3623**  **D3S3623**  **D3S3623**  **D3S3623**  **D3S3623**  **D3S3623**  **D3S3623**  **D3S3623**  **D3S3623**  **D3S3623**  **D3S3623**  **D3S3623**  **D3S3623**  **D3S3623**  **D3S3623**  **D3S3623**  **D3S3623** | **219**  **219**  **219**  **219**  **219**  **219**  **219**  **219**  **219**  **219**  **219**  **219**  **219**  **219**  **219**  **219**  **219**  **219**  **219**  **219**  **219**  **219**  **219**  **219** | **221**  **221**  **221**  **221**  **221**  **221**  **221**  **221**  **221**  **221**  **221**  **221**  **221**  **221**  **221**  **221**  **221**  **221**  **221**  **221**  **221**  **221**  **221**  **221** | **218.4**  **218.5**  **218.5**  **218.6**  **218.4**  **218.4**  **218.3**  **218.3**  **218.4**  **218.4**  **218.6**  **218.4**  **218.5**  **218.4**  **218.5**  **218.6**  **218.5**  **218.6**  **218.5**  **218.4**  **218.4**  **218.6**  **218.5**  **218.5** | **220.5**  **220.6**  **220.6**  **220.6**  **220.6**  **220.5**  **220.5**  **220.4**  **220.5**  **220.5**  **220.7**  **220.4**  **220.5**  **220.5**  **220.6**  **220.6**  **220.6**  **220.7**  **220.6**  **220.5**  **220.6**  **220.7**  **220.6**  **220.5** | **972**  **1029**  **756**  **548**  **358**  **387**  **852**  **1196**  **306**  **959**  **508**  **277**  **399**  **221**  **292**  **248**  **672**  **912**  **913**  **415**  **523**  **832**  **418**  **444** | **506**  **667**  **495**  **335**  **319**  **227**  **498**  **849**  **232**  **688**  **435**  **193**  **234**  **147**  **169**  **142**  **440**  **342**  **624**  **264**  **335**  **531**  **258**  **289** | **7879**  **8270**  **5852**  **4119**  **3110**  **3358**  **7049**  **9676**  **2505**  **8045**  **4559**  **2161**  **3211**  **1844**  **2227**  **1910**  **5454**  **7530**  **7271**  **3329**  **4382**  **6813**  **3350**  **3435** | **3872**  **5105**  **3811**  **2484**  **2850**  **1775**  **3910**  **6832**  **1775**  **5179**  **3631**  **1424**  **1803**  **1189**  **1267**  **1122**  **3445**  **2656**  **4864**  **2104**  **2653**  **4284**  **2000**  **2132** | **4710**  **4459**  **4508**  **4455**  **4655**  **4496**  **4679**  **4660**  **4662**  **4664**  **4552**  **4482**  **4486**  **4511**  **4510**  **4484**  **4497**  **4519**  **4501**  **4474**  **4488**  **4509**  **4502**  **4476** | **4734**  **4482**  **4532**  **4477**  **4680**  **4519**  **4704**  **4685**  **4686**  **4689**  **4576**  **4505**  **4509**  **4535**  **4533**  **4507**  **4521**  **4543**  **4524**  **4497**  **4512**  **4533**  **4525**  **4499** |
| **1** | **1**  **2**  **3**  **4**  **5**  **6**  **7**  **8**  **9**  **10** | **7/18/05**  **7/18/05**  **7/18/05**  **7/18/05**  **7/18/05**  **7/18/05**  **7/18/05**  **7/18/05**  **7/18/05**  **7/18/05** | **CD014B1M3**  **CD014B1M11**  **CD014B1M19**  **CD014B1M12**  **CD014B1M5**  **CD014B1M13**  **CD014B1M7**  **CD014B1M8**  **CD014B1M2**  **CD014B1M10** | **D5S346**  **D5S346**  **D5S346**  **D5S346**  **D5S346**  **D5S346**  **D5S346**  **D5S346**  **D5S346**  **D5S346** | **89**  **89**  **89**  **89**  **89**  **89**  **89**  **89**  **89**  **89** | **92**  **92**  **92**  **92**  **92**  **92**  **92**  **92**  **92**  **92** | **89.5**  **89.5**  **89.5**  **89.5**  **89.5**  **89.5**  **89.5**  **89.5**  **89.5**  **89.5** | **91.7**  **91.7**  **91.7**  **91.8**  **91.7**  **91.6**  **91.7**  **91.7**  **91.7**  **91.7** | **1441**  **3470**  **1206**  **916**  **524**  **1299**  **925**  **887**  **3509**  **2702** | **922**  **2529**  **894**  **705**  **240**  **940**  **644**  **683**  **2488**  **2053** | **11904**  **25638**  **8994**  **6799**  **4347**  **10237**  **7455**  **7123**  **27574**  **21433** | **7142**  **18165**  **6580**  **4852**  **1819**  **7479**  **4821**  **5340**  **18746**  **15589** | **3060**  **2882**  **2916**  **2877**  **3024**  **2910**  **3034**  **3022**  **3021**  **3023** | **3090**  **2911**  **2945**  **2906**  **3054**  **2938**  **3064**  **3051**  **3050**  **3053** |

| **Day** | **Count** | **Run Date** | **Sample Name** | **Marker** | **Allele 1** | **Allele 2** | **Size 1** | **Size 2** | **Height 1** | **Height 2** | **Peak Area 1** | **Peak Area 2** | **Data Point 1** | **Data Point 2** |
| --- | --- | --- | --- | --- | --- | --- | --- | --- | --- | --- | --- | --- | --- | --- |
|  | **11**  **12**  **13**  **14**  **15**  **16**  **17**  **18**  **19**  **20**  **21**  **22**  **23**  **24** | **7/18/05**  **7/18/05**  **7/18/05**  **7/18/05**  **7/18/05**  **7/18/05**  **7/18/05**  **7/18/05**  **7/18/05**  **7/18/05**  **7/18/05**  **7/18/05**  **7/18/05**  **7/18/05** | **CD014B1M18**  **CD014B1M20**  **CD014B1M21**  **CD014B1M22**  **CD014B1M23**  **CD014B1M24**  **CD014B1M25**  **CD014B1M30**  **CD014B1M31**  **CD014B1M32**  **CD014B1M37**  **CD014B1M44**  **CD014B1M46**  **CD014B1M48** | **D5S346**  **D5S346**  **D5S346**  **D5S346**  **D5S346**  **D5S346**  **D5S346**  **D5S346**  **D5S346**  **D5S346**  **D5S346**  **D5S346**  **D5S346**  **D5S346** | **89**  **89**  **89**  **89**  **89**  **89**  **89**  **89**  **89**  **89**  **89**  **89**  **89**  **89** | **92**  **92**  **92**  **92**  **92**  **92**  **92**  **92**  **92**  **92**  **92**  **92**  **92**  **92** | **89.5**  **89.5**  **89.5**  **89.5**  **89.6**  **89.5**  **89.5**  **89.6**  **89.5**  **89.5**  **89.5**  **89.5**  **89.4**  **89.5** | **91.8**  **91.7**  **91.7**  **91.7**  **91.8**  **91.7**  **91.6**  **91.8**  **91.7**  **91.7**  **91.7**  **91.7**  **91.7**  **91.7** | **955**  **4138**  **3271**  **930**  **2344**  **1112**  **1038**  **1590**  **1913**  **549**  **1117**  **1701**  **778**  **868** | **644**  **3002**  **2408**  **709**  **1752**  **732**  **766**  **1117**  **1555**  **359**  **840**  **1299**  **585**  **672** | **7551**  **31596**  **25169**  **6863**  **18162**  **8420**  **7812**  **12165**  **14476**  **4189**  **8618**  **12916**  **5938**  **6507** | **4978**  **22101**  **17937**  **5101**  **13278**  **5440**  **5754**  **8350**  **11693**  **2651**  **6245**  **9530**  **4290**  **4785** | **2942**  **2897**  **2903**  **2918**  **2914**  **2895**  **2909**  **2920**  **2911**  **2893**  **2905**  **2916**  **2910**  **2892** | **2972**  **2926**  **2932**  **2947**  **2943**  **2924**  **2937**  **2949**  **2940**  **2921**  **2933**  **2945**  **2939**  **2921** |
| **1** | **1**  **2**  **3**  **4**  **5**  **6**  **7**  **8**  **9**  **10**  **11**  **12**  **13**  **14**  **15**  **16**  **17**  **18**  **19**  **20**  **21**  **22**  **23**  **24** | **7/18/05**  **7/18/05**  **7/18/05**  **7/18/05**  **7/18/05**  **7/18/05**  **7/18/05**  **7/18/05**  **7/18/05**  **7/18/05**  **7/18/05**  **7/18/05**  **7/18/05**  **7/18/05**  **7/18/05**  **7/18/05**  **7/18/05**  **7/18/05**  **7/18/05**  **7/18/05**  **7/18/05**  **7/18/05**  **7/18/05**  **7/18/05** | **CD014B1M3**  **CD014B1M11**  **CD014B1M19**  **CD014B1M12**  **CD014B1M5**  **CD014B1M13**  **CD014B1M7**  **CD014B1M8**  **CD014B1M2**  **CD014B1M10**  **CD014B1M18**  **CD014B1M20**  **CD014B1M21**  **CD014B1M22**  **CD014B1M23**  **CD014B1M24**  **CD014B1M25**  **CD014B1M30**  **CD014B1M31**  **CD014B1M32**  **CD014B1M37**  **CD014B1M44**  **CD014B1M46**  **CD014B1M48** | **D6S262**  **D6S262**  **D6S262**  **D6S262**  **D6S262**  **D6S262**  **D6S262**  **D6S262**  **D6S262**  **D6S262**  **D6S262**  **D6S262**  **D6S262**  **D6S262**  **D6S262**  **D6S262**  **D6S262**  **D6S262**  **D6S262**  **D6S262**  **D6S262**  **D6S262**  **D6S262**  **D6S262** | **174**  **174**  **174**  **174**  **174**  **174**  **174**  **174**  **174**  **174**  **174**  **174**  **174**  **174**  **174**  **174**  **174**  **174**  **174**  **174**  **174**  **174**  **174**  **174** | **182**  **182**  **182**  **182**  **182**  **182**  **182**  **182**  **182**  **182**  **182**  **182**  **182**  **182**  **182**  **182**  **182**  **182**  **182**  **182**  **182**  **182**  **182**  **182** | **174.3**  **174.2**  **174.2**  **174.2**  **174.2**  **174.2**  **174.1**  **174.2**  **174.2**  **174.2**  **174.3**  **174.2**  **174.3**  **174.3**  **174.3**  **174.3**  **174.3**  **174.3**  **174.2**  **174.2**  **174.3**  **174.2**  **174.2**  **174.1** | **182.0**  **181.9**  **182.0**  **182.0**  **182.0**  **182.0**  **181.8**  **181.9**  **181.9**  **181.9**  **182.0**  **181.8**  **182.0**  **182.0**  **182.0**  **182.0**  **181.9**  **182.0**  **181.9**  **182.0**  **182.0**  **182.0**  **181.9**  **181.9** | **466**  **1206**  **798**  **675**  **423**  **408**  **693**  **889**  **597**  **930**  **443**  **751**  **720**  **385**  **404**  **306**  **614**  **578**  **917**  **356**  **626**  **819**  **334**  **447** | **333**  **845**  **589**  **519**  **226**  **301**  **412**  **612**  **312**  **669**  **291**  **462**  **478**  **220**  **272**  **198**  **473**  **407**  **493**  **100**  **451**  **611**  **324**  **322** | **4101**  **9728**  **6667**  **5486**  **3688**  **3592**  **5693**  **7126**  **5298**  **7835**  **4195**  **6256**  **6358**  **3291**  **3353**  **2646**  **5272**  **4721**  **7596**  **2788**  **5392**  **6836**  **2944**  **3694** | **2547**  **6296**  **4568**  **3818**  **1895**  **2261**  **3193**  **4729**  **2494**  **5219**  **2444**  **3693**  **3773**  **1687**  **2176**  **1506**  **3714**  **3194**  **3948**  **748**  **3667**  **4605**  **2379**  **2374** | **4162**  **3936**  **3980**  **3931**  **4114**  **3970**  **4133**  **4116**  **4116**  **4119**  **4018**  **3957**  **3961**  **3983**  **3980**  **3956**  **3971**  **3988**  **3973**  **3950**  **3964**  **3980**  **3974**  **3950** | **4262**  **4031**  **4076**  **4027**  **4213**  **4066**  **4232**  **4215**  **4215**  **4218**  **4115**  **4051**  **4056**  **4079**  **4076**  **4052**  **4066**  **4084**  **4069**  **4046**  **4059**  **4076**  **4069**  **4046** |
| **1** | **1**  **2**  **3**  **4**  **5**  **6**  **7**  **8**  **9**  **10**  **11**  **12**  **13**  **14**  **15**  **16**  **17**  **18**  **19**  **20** | **7/18/05**  **7/18/05**  **7/18/05**  **7/18/05**  **7/18/05**  **7/18/05**  **7/18/05**  **7/18/05**  **7/18/05**  **7/18/05**  **7/18/05**  **7/18/05**  **7/18/05**  **7/18/05**  **7/18/05**  **7/18/05**  **7/18/05**  **7/18/05**  **7/18/05**  **7/18/05** | **CD014B1M3**  **CD014B1M11**  **CD014B1M19**  **CD014B1M12**  **CD014B1M5**  **CD014B1M13**  **CD014B1M7**  **CD014B1M8**  **CD014B1M2**  **CD014B1M10**  **CD014B1M18**  **CD014B1M20**  **CD014B1M21**  **CD014B1M22* CD014B1M23**  **CD014B1M24* CD014B1M25**  **CD014B1M30**  **CD014B1M31**  **CD014B1M32** | **D7S481**  **D7S481**  **D7S481**  **D7S481**  **D7S481**  **D7S481**  **D7S481**  **D7S481**  **D7S481**  **D7S481**  **D7S481**  **D7S481**  **D7S481**  **D7S481* D7S481**  **D7S481* D7S481**  **D7S481**  **D7S481**  **D7S481** | **199**  **199**  **199**  **199**  **199**  **199**  **199**  **199**  **199**  **199**  **199**  **199**  **199**  **199**  **199**  **199**  **199**  **199**  **199**  **199** | **201**  **201**  **201**  **201**  **201**  **201**  **201**  **201**  **201**  **201**  **201**  **201**  **201**  **201**  **201**  **201**  **201**  **201**  **201**  **201** | **198.8**  **198.8**  **198.8**  **198.8**  **198.7**  **198.8**  **198.7**  **198.8**  **198.7**  **198.7**  **198.7**  **198.7**  **198.8**  **198.8**  **198.7**  **198.9**  **198.8**  **198.8**  **198.8**  **198.8** | **200.7**  **200.7**  **200.8**  **200.7**  **200.7**  **200.7**  **200.7**  **200.6**  **200.8**  **200.7**  **200.8**  **200.6**  **200.7**  **200.8**  **200.7**  **200.8**  **200.9**  **200.7**  **200.7**  **200.6** | **435**  **338**  **173**  **127**  **218**  **150**  **305**  **284**  **86**  **293**  **141**  **117**  **130**  **59**  **118**  **154**  **141**  **293**  **339**  **160** | **260**  **202**  **125**  **81**  **68**  **131**  **189**  **197**  **67**  **194**  **140**  **72**  **97**  **45**  **110**  **164**  **95**  **99**  **245**  **94** | **3628**  **2619**  **1361**  **963**  **1761**  **1277**  **2510**  **2390**  **721**  **2351**  **1311**  **974**  **1109**  **481**  **968**  **1288**  **1138**  **2341**  **2722**  **1330** | **2084**  **1680**  **984**  **773**  **522**  **1097**  **1582**  **1627**  **699**  **1495**  **1416**  **674**  **806**  **404**  **902**  **1459**  **796**  **724**  **1992**  **825** | **4477**  **4237**  **4284**  **4232**  **4425**  **4273**  **4448**  **4430**  **4429**  **4433**  **4324**  **4259**  **4263**  **4287**  **4284**  **4260**  **4274**  **4293**  **4277**  **4252** | **4501**  **4260**  **4308**  **4255**  **4449**  **4296**  **4472**  **4453**  **4455**  **4457**  **4349**  **4282**  **4286**  **4311**  **4308**  **4283**  **4299**  **4316**  **4300**  **4274** |

| **Day** | **Count** | **Run Date** | **Sample Name** | **Marker** | **Allele 1** | **Allele 2** | **Size 1** | **Size 2** | **Height 1** | **Height 2** | **Peak Area 1** | **Peak Area 2** | **Data Point 1** | **Data Point 2** |
| --- | --- | --- | --- | --- | --- | --- | --- | --- | --- | --- | --- | --- | --- | --- |
|  | **21**  **22**  **23**  **24** | **7/18/05**  **7/18/05**  **7/18/05**  **7/18/05** | **CD014B1M37**  **CD014B1M44**  **CD014B1M46**  **CD014B1M48** | **D7S481**  **D7S481**  **D7S481**  **D7S481** | **199**  **199**  **199**  **199** | **201**  **201**  **201**  **201** | **198.8**  **198.9**  **198.8**  **198.8** | **200.6**  **200.8**  **200.8**  **200.8** | **104**  **167**  **102**  **115** | **73**  **113**  **74**  **86** | **905**  **1397**  **780**  **801** | **632**  **1029**  **683**  **682** | **4266**  **4285**  **4278**  **4253** | **4288**  **4308**  **4302**  **4277** |
| **1**  **2**  **3** | **1**  **2**  **3**  **4**  **5**  **6**  **7**  **8**  **9**  **10**  **11**  **12**  **13**  **14**  **15**  **16**  **17**  **18**  **19**  **20**  **21**  **22**  **23**  **24**  **25**  **26**  **27**  **28**  **29**  **30**  **31**  **32**  **33**  **34**  **35**  **36**  **37**  **38**  **39**  **40**  **41**  **42**  **43** | **7/11/05**  **7/11/05**  **7/11/05**  **7/11/05**  **7/11/05**  **7/11/05**  **7/11/05**  **7/11/05**  **7/11/05**  **7/13/05**  **7/13/05**  **7/13/05**  **7/13/05**  **7/13/05**  **7/13/05**  **7/13/05**  **7/13/05**  **7/13/05**  **7/13/05**  **7/13/05**  **7/13/05**  **7/13/05**  **7/13/05**  **7/13/05**  **7/13/05**  **7/13/05**  **7/13/05**  **7/13/05**  **7/13/05**  **7/13/05**  **7/13/05**  **7/13/05**  **7/13/05**  **7/13/05**  **7/13/05**  **7/13/05**  **7/13/05**  **7/13/05**  **7/13/05**  **7/13/05**  **7/13/05**  **7/18/05**  **7/18/05** | **CD015B1_M12**  **CD015B1_M1**  **CD015B1_M13**  **CD015B1_M2**  **CD015B1_M14**  **CD015B1_M3**  **CD015B1_M5**  **CD015B1_M9**  **CD015B1_M10**  **CD015B1_M17**  **CD015B1_M30**  **CD015B1_M42**  **CD015B1_M54**  **CD015B1_M66**  **CD015B1_M19**  **CD015B1_M31**  **CD015B1_M44**  **CD015B1_M67**  **CD015B1_M32**  **CD015B1_M46**  **CD015B1_M56**  **CD015B1_M70**  **CD015B1_M21**  **CD015B1_M34**  **CD015B1_M48**  **CD015B1_M57**  **CD015B1_M72**  **CD015B1_M23**  **CD015B1_M36**  **CD015B1_M58**  **CD015B1_M73**  **CD015B1_M26**  **CD015B1_M50**  **CD015B1_M38**  **CD015B1_M51**  **CD015B1_M64**  **CD015B1_M15**  **CD015B1_M29**  **CD015B1_M39**  **CD015B1_M53**  **CD015B1_M65**  **CD015B1M20**  **CD015B1M28** | **BAT25**  **BAT25**  **BAT25**  **BAT25**  **BAT25**  **BAT25**  **BAT25**  **BAT25**  **BAT25**  **BAT25**  **BAT25**  **BAT25**  **BAT25**  **BAT25**  **BAT25**  **BAT25**  **BAT25**  **BAT25**  **BAT25**  **BAT25**  **BAT25**  **BAT25**  **BAT25**  **BAT25**  **BAT25**  **BAT25**  **BAT25**  **BAT25**  **BAT25**  **BAT25**  **BAT25**  **BAT25**  **BAT25**  **BAT25**  **BAT25**  **BAT25**  **BAT25**  **BAT25**  **BAT25**  **BAT25**  **BAT25**  **BAT25**  **BAT25** | **126**  **126**  **126**  **126**  **126**  **126**  **126**  **126**  **126**  **126**  **126**  **126**  **126**  **126**  **126**  **126**  **126**  **126**  **126**  **126**  **127**  **126**  **126**  **126**  **126**  **126**  **126**  **126**  **126**  **126**  **126**  **126**  **126**  **126**  **126**  **126**  **126**  **126**  **126**  **127**  **126**  **126**  **126** | **127**  **127**  **127**  **127**  **127**  **127** | **125.8**  **125.8**  **125.8**  **125.8**  **125.9**  **125.8**  **125.9**  **125.8**  **125.9**  **125.8**  **125.9**  **125.9**  **125.8**  **125.9**  **125.8**  **125.9**  **125.8**  **125.9**  **125.8**  **125.8**  **126.9**  **125.9**  **125.8**  **125.9**  **125.9**  **125.9**  **125.9**  **125.8**  **125.9**  **125.9**  **125.9**  **125.8**  **125.8**  **125.8**  **125.8**  **125.9**  **125.9**  **125.8**  **126.0**  **126.9**  **125.9**  **125.9**  **125.8** | **126.8**  **126.9**  **126.9**  **126.9**  **126.9**  **126.9** | **1343**  **2969**  **417**  **1772**  **1455**  **1205**  **879**  **1023**  **3224**  **948**  **2898**  **2591**  **1107**  **2987**  **578**  **1375**  **2274**  **683**  **2793**  **2153**  **416**  **2023**  **1558**  **794**  **731**  **2630**  **3653**  **1080**  **3297**  **3540**  **2159**  **1374**  **4105**  **2301**  **2252**  **1739**  **2306**  **3358**  **3867**  **278**  **1246**  **1295**  **1031** | **942**  **2789**  **600**  **745**  **1315**  **4139** | **10334**  **22856**  **3369**  **14948**  **11516**  **9832**  **6684**  **8264**  **24941**  **6931**  **22847**  **20219**  **8615**  **24704**  **4391**  **10659**  **17565**  **5603**  **22803**  **17033**  **3630**  **15852**  **11934**  **6309**  **5860**  **20065**  **29262**  **8418**  **25567**  **27114**  **17310**  **10392**  **31616**  **18850**  **18170**  **13614**  **17888**  **26355**  **29459**  **2787**  **9726**  **10003**  **7862** | **6717**  **21826**  **4532**  **5994**  **10352**  **32520** | **3374**  **3355**  **3345**  **3394**  **3386**  **3404**  **3352**  **3394**  **3402**  **3390**  **3477**  **3496**  **3513**  **3582**  **3367**  **3455**  **3474**  **3557**  **3470**  **3489**  **3517**  **3570**  **3402**  **3484**  **3503**  **3519**  **3582**  **3464**  **3498**  **3536**  **3608**  **3442**  **3470**  **3490**  **3486**  **3525**  **3415**  **3469**  **3506**  **3517**  **3538**  **3378**  **3392** | **3403**  **3490**  **3381**  **3517**  **3455**  **3484** |
| **1**  **2** | **1**  **2**  **3**  **4**  **5**  **6**  **7**  **8**  **9**  **10** | **7/11/05**  **7/11/05**  **7/11/05**  **7/11/05**  **7/11/05**  **7/11/05**  **7/11/05**  **7/11/05**  **7/11/05**  **7/13/05** | **CD015B1_M12**  **CD015B1_M1**  **CD015B1_M13**  **CD015B1_M2**  **CD015B1_M14**  **CD015B1_M3**  **CD015B1_M5**  **CD015B1_M9**  **CD015B1_M10**  **CD015B1_M17** | **BAT26**  **BAT26**  **BAT26**  **BAT26**  **BAT26**  **BAT26**  **BAT26**  **BAT26**  **BAT26**  **BAT26** | **122**  **122**  **122**  **122**  **122**  **122**  **122**  **122**  **122**  **122** |  | **121.7**  **121.8**  **121.8**  **121.8**  **121.7**  **121.8**  **121.9**  **122.0**  **121.8**  **121.7** |  | **487**  **931**  **205**  **516**  **553**  **688**  **442**  **234**  **1324**  **400** |  | **4444**  **9321**  **1731**  **5463**  **4947**  **6796**  **4091**  **2211**  **12482**  **3541** |  | **3322**  **3304**  **3293**  **3343**  **3333**  **3352**  **3301**  **3344**  **3349**  **3337** |  |

| **Day** | **Count** | **Run Date** | **Sample Name** | **Marker** | **Allele 1** | **Allele 2** | **Size 1** | **Size 2** | **Height 1** | **Height 2** | **Peak Area 1** | **Peak Area 2** | **Data Point 1** | **Data Point 2** |
| --- | --- | --- | --- | --- | --- | --- | --- | --- | --- | --- | --- | --- | --- | --- |
|  | **11**  **12**  **13**  **14**  **15**  **16** | **7/13/05**  **7/13/05**  **7/13/05**  **7/13/05**  **7/13/05**  **7/13/05** | **CD015B1_M30**  **CD015B1_M42**  **CD015B1_M54**  **CD015B1_M66**  **CD015B1_M19**  **CD015B1_M31** | **BAT26**  **BAT26**  **BAT26**  **BAT26**  **BAT26**  **BAT26** | **122**  **122**  **122**  **122**  **122**  **122** |  | **121.9**  **121.8**  **121.8**  **121.9**  **121.8**  **121.8** |  | **1094**  **799**  **468**  **1008**  **306**  **487** |  | **10769**  **8175**  **4652**  **9666**  **2529**  **4652** |  | **3424**  **3442**  **3460**  **3528**  **3316**  **3402** |  |
| **3** | **17**  **18**  **19**  **20**  **21**  **22**  **23**  **24**  **25**  **26**  **27**  **28**  **29**  **30**  **31**  **32**  **33**  **34**  **35**  **36**  **37**  **38**  **39**  **40**  **41**  **42**  **43** | **7/13/05**  **7/13/05**  **7/13/05**  **7/13/05**  **7/13/05**  **7/13/05**  **7/13/05**  **7/13/05**  **7/13/05**  **7/13/05**  **7/13/05**  **7/13/05**  **7/13/05**  **7/13/05**  **7/13/05**  **7/13/05**  **7/13/05**  **7/13/05**  **7/13/05**  **7/13/05**  **7/13/05**  **7/13/05**  **7/13/05**  **7/13/05**  **7/13/05**  **7/18/05**  **7/18/05** | **CD015B1_M44**  **CD015B1_M67**  **CD015B1_M32**  **CD015B1_M46**  **CD015B1_M56**  **CD015B1_M70**  **CD015B1_M21**  **CD015B1_M34**  **CD015B1_M48**  **CD015B1_M57**  **CD015B1_M72**  **CD015B1_M23**  **CD015B1_M36**  **CD015B1_M58**  **CD015B1_M73**  **CD015B1_M26**  **CD015B1_M50**  **CD015B1_M38**  **CD015B1_M51**  **CD015B1_M64**  **CD015B1_M15**  **CD015B1_M29**  **CD015B1_M39**  **CD015B1_M53**  **CD015B1_M65**  **CD015B1M20**  **CD015B1M28** | **BAT26**  **BAT26**  **BAT26**  **BAT26**  **BAT26**  **BAT26**  **BAT26**  **BAT26**  **BAT26**  **BAT26**  **BAT26**  **BAT26**  **BAT26**  **BAT26**  **BAT26**  **BAT26**  **BAT26**  **BAT26**  **BAT26**  **BAT26**  **BAT26**  **BAT26**  **BAT26**  **BAT26**  **BAT26**  **BAT26**  **BAT26** | **122**  **122**  **122**  **122**  **122**  **122**  **122**  **122**  **122**  **122**  **122**  **122**  **122**  **122**  **122**  **122**  **122**  **122**  **122**  **122**  **122**  **122**  **122**  **122**  **122**  **122**  **122** | **123** | **121.8**  **121.8**  **121.7**  **121.8**  **121.8**  **121.8**  **121.8**  **121.8**  **121.8**  **121.8**  **121.8**  **121.9**  **121.8**  **121.8**  **121.9**  **121.8**  **121.9**  **121.8**  **121.8**  **121.8**  **121.8**  **121.8**  **121.9**  **121.8**  **121.8**  **121.8**  **121.8** | **122.7** | **953**  **386**  **1196**  **925**  **134**  **827**  **659**  **735**  **520**  **977**  **1401**  **354**  **1218**  **1184**  **722**  **648**  **1431**  **950**  **862**  **562**  **1148**  **1314**  **1436**  **202**  **702**  **363**  **398** | **1368** | **9612**  **3715**  **11830**  **8788**  **1131**  **7683**  **5678**  **6672**  **4634**  **8865**  **12528**  **3485**  **11569**  **11751**  **6639**  **5933**  **13317**  **9013**  **8275**  **5399**  **11129**  **12100**  **13852**  **1634**  **6872**  **3289**  **3378** | **11351** | **3421**  **3503**  **3417**  **3436**  **3451**  **3516**  **3350**  **3431**  **3450**  **3465**  **3528**  **3412**  **3445**  **3482**  **3554**  **3390**  **3418**  **3437**  **3433**  **3472**  **3362**  **3417**  **3453**  **3450**  **3484**  **3326**  **3340** | **3429** |
| **1**  **2** | **1**  **2**  **3**  **4**  **5**  **6**  **7**  **8**  **9**  **10**  **11**  **12**  **13**  **14**  **15**  **16**  **17**  **18**  **19**  **20**  **21**  **22**  **23**  **24**  **25** | **7/11/05**  **7/11/05**  **7/11/05**  **7/11/05**  **7/11/05**  **7/11/05**  **7/11/05**  **7/11/05**  **7/11/05**  **7/13/05**  **7/13/05**  **7/13/05**  **7/13/05**  **7/13/05**  **7/13/05**  **7/13/05**  **7/13/05**  **7/13/05**  **7/13/05**  **7/13/05**  **7/13/05**  **7/13/05**  **7/13/05**  **7/13/05**  **7/13/05** | **CD015B1_M12**  **CD015B1_M1**  **CD015B1_M13**  **CD015B1_M2**  **CD015B1_M14**  **CD015B1_M3**  **CD015B1_M5**  **CD015B1_M9**  **CD015B1_M10**  **CD015B1_M17**  **CD015B1_M30**  **CD015B1_M42**  **CD015B1_M54**  **CD015B1_M66**  **CD015B1_M19**  **CD015B1_M31**  **CD015B1_M44**  **CD015B1_M67**  **CD015B1_M32**  **CD015B1_M46**  **CD015B1_M56**  **CD015B1_M70**  **CD015B1_M21**  **CD015B1_M34**  **CD015B1_M48** | **D3S3623**  **D3S3623**  **D3S3623**  **D3S3623**  **D3S3623**  **D3S3623**  **D3S3623**  **D3S3623**  **D3S3623**  **D3S3623**  **D3S3623**  **D3S3623**  **D3S3623**  **D3S3623**  **D3S3623**  **D3S3623**  **D3S3623**  **D3S3623**  **D3S3623**  **D3S3623**  **D3S3623**  **D3S3623**  **D3S3623**  **D3S3623**  **D3S3623** | **221**  **221**  **221**  **221**  **221**  **221**  **221**  **221**  **221**  **221**  **221**  **221**  **221**  **221**  **221**  **221**  **221**  **221**  **221**  **221**  **221**  **221**  **221**  **221**  **221** |  | **220.6**  **220.6**  **220.6**  **220.6**  **220.7**  **220.7**  **220.5**  **220.5**  **220.6**  **220.5**  **220.7**  **220.7**  **220.6**  **220.8**  **220.5**  **220.7**  **220.6**  **220.8**  **220.6**  **220.7**  **220.6**  **220.8**  **220.6**  **220.7**  **220.7** |  | **2025**  **2453**  **1123**  **2725**  **2851**  **1547**  **2427**  **2292**  **6394**  **2761**  **5316**  **4148**  **1842**  **6163**  **1042**  **2569**  **4791**  **1050**  **6549**  **3703**  **485**  **4199**  **3939**  **2088**  **1352** |  | **17330**  **20976**  **9230**  **24736**  **25065**  **13767**  **20702**  **20457**  **55962**  **23888**  **47505**  **37568**  **16738**  **57103**  **8759**  **22542**  **42242**  **9484**  **58654**  **33313**  **4471**  **38407**  **34184**  **18640**  **12256** |  | **4517**  **4490**  **4478**  **4539**  **4527**  **4556**  **4486**  **4538**  **4551**  **4532**  **4641**  **4665**  **4687**  **4773**  **4502**  **4612**  **4635**  **4742**  **4626**  **4650**  **4669**  **4752**  **4547**  **4649**  **4672** |  |

| **Day** | **Count** | **Run Date** | **Sample Name** | **Marker** | **Allele 1** | **Allele 2** | **Size 1** | **Size 2** | **Height 1** | **Height 2** | **Peak Area 1** | **Peak Area 2** | **Data Point 1** | **Data Point 2** |
| --- | --- | --- | --- | --- | --- | --- | --- | --- | --- | --- | --- | --- | --- | --- |
| **3** | **26**  **27**  **28**  **29**  **30**  **31**  **32**  **33**  **34**  **35**  **36**  **37**  **38**  **39**  **40**  **41**  **42**  **43** | **7/13/05**  **7/13/05**  **7/13/05**  **7/13/05**  **7/13/05**  **7/13/05**  **7/13/05**  **7/13/05**  **7/13/05**  **7/13/05**  **7/13/05**  **7/13/05**  **7/13/05**  **7/13/05**  **7/13/05**  **7/13/05**  **7/18/05**  **7/18/05** | **CD015B1_M57**  **CD015B1_M72**  **CD015B1_M23**  **CD015B1_M36**  **CD015B1_M58**  **CD015B1_M73**  **CD015B1_M26**  **CD015B1_M50**  **CD015B1_M38**  **CD015B1_M51**  **CD015B1_M64**  **CD015B1_M15**  **CD015B1_M29**  **CD015B1_M39**  **CD015B1_M53**  **CD015B1_M65**  **CD015B1M20**  **CD015B1M28** | **D3S3623**  **D3S3623**  **D3S3623**  **D3S3623**  **D3S3623**  **D3S3623**  **D3S3623**  **D3S3623**  **D3S3623**  **D3S3623**  **D3S3623**  **D3S3623**  **D3S3623**  **D3S3623**  **D3S3623**  **D3S3623**  **D3S3623**  **D3S3623** | **221**  **221**  **221**  **221**  **221**  **221**  **221**  **221**  **221**  **221**  **221**  **221**  **221**  **221**  **221**  **221**  **221**  **221** |  | **220.7**  **220.9**  **220.7**  **220.6**  **220.7**  **220.9**  **220.6**  **220.6**  **220.6**  **220.6**  **220.6**  **220.6**  **220.7**  **220.7**  **220.7**  **220.8**  **220.6**  **220.7** |  | **4183**  **6506**  **2173**  **6228**  **5447**  **4097**  **1668**  **2070**  **3839**  **3880**  **2410**  **6338**  **5617**  **6702**  **876**  **2141**  **772**  **324** |  | **37597**  **60928**  **19555**  **56489**  **50737**  **38894**  **14502**  **18258**  **34543**  **34957**  **21764**  **55100**  **48888**  **59958**  **7704**  **19504**  **6752**  **2820** |  | **4694**  **4774**  **4624**  **4666**  **4715**  **4808**  **4596**  **4629**  **4651**  **4645**  **4695**  **4558**  **4631**  **4676**  **4671**  **4717**  **4511**  **4533** |  |
| **1**  **2** | **1**  **2**  **3**  **4**  **5**  **6**  **7**  **8**  **9**  **10**  **11**  **12**  **13**  **14**  **15**  **16**  **17**  **18**  **19**  **20**  **21**  **22**  **23**  **24**  **25**  **26**  **27**  **28**  **29**  **30**  **31**  **32**  **33**  **34**  **35**  **36**  **37**  **38**  **39**  **40** | **7/11/05**  **7/11/05**  **7/11/05**  **7/11/05**  **7/11/05**  **7/11/05**  **7/11/05**  **7/11/05**  **7/11/05**  **7/13/05**  **7/13/05**  **7/13/05**  **7/13/05**  **7/13/05**  **7/13/05**  **7/13/05**  **7/13/05**  **7/13/05**  **7/13/05**  **7/13/05**  **7/13/05**  **7/13/05**  **7/13/05**  **7/13/05**  **7/13/05**  **7/13/05**  **7/13/05**  **7/13/05**  **7/13/05**  **7/13/05**  **7/13/05**  **7/13/05**  **7/13/05**  **7/13/05**  **7/13/05**  **7/13/05**  **7/13/05**  **7/13/05**  **7/13/05**  **7/13/05** | **CD015B1_M12**  **CD015B1_M1**  **CD015B1_M13**  **CD015B1_M2**  **CD015B1_M14**  **CD015B1_M3**  **CD015B1_M5**  **CD015B1_M9**  **CD015B1_M10**  **CD015B1_M17**  **CD015B1_M30**  **CD015B1_M42**  **CD015B1_M54**  **CD015B1_M66**  **CD015B1_M19**  **CD015B1_M31**  **CD015B1_M44**  **CD015B1_M67**  **CD015B1_M32**  **CD015B1_M46**  **CD015B1_M56**  **CD015B1_M70**  **CD015B1_M21**  **CD015B1_M34**  **CD015B1_M48* CD015B1_M57**  **CD015B1_M72**  **CD015B1_M23**  **CD015B1_M36**  **CD015B1_M58**  **CD015B1_M73**  **CD015B1_M26**  **CD015B1_M50**  **CD015B1_M38**  **CD015B1_M51**  **CD015B1_M64**  **CD015B1_M15**  **CD015B1_M29**  **CD015B1_M39**  **CD015B1_M53** | **D5S346**  **D5S346**  **D5S346**  **D5S346**  **D5S346**  **D5S346**  **D5S346**  **D5S346**  **D5S346**  **D5S346**  **D5S346**  **D5S346**  **D5S346**  **D5S346**  **D5S346**  **D5S346**  **D5S346**  **D5S346**  **D5S346**  **D5S346**  **D5S346**  **D5S346**  **D5S346**  **D5S346**  **D5S346* D5S346**  **D5S346**  **D5S346**  **D5S346**  **D5S346**  **D5S346**  **D5S346**  **D5S346**  **D5S346**  **D5S346**  **D5S346**  **D5S346**  **D5S346**  **D5S346**  **D5S346** | **92**  **92**  **92**  **92**  **92**  **92**  **92**  **92**  **92**  **92**  **92**  **92**  **92**  **92**  **92**  **92**  **92**  **92**  **92**  **92**  **92**  **92**  **92**  **92**  **92**  **92**  **92**  **92**  **92**  **92**  **92**  **92**  **92**  **92**  **92**  **92**  **92**  **92**  **92**  **92** | **101**  **101**  **101**  **101**  **101**  **101**  **101**  **101**  **101**  **101**  **101**  **101**  **101**  **101**  **101**  **101**  **101**  **101**  **101**  **101**  **101**  **101**  **101**  **101**  **101**  **101**  **101**  **101**  **101**  **101**  **101**  **101**  **101**  **101**  **101**  **101**  **101**  **101**  **101**  **101** | **91.7**  **91.7**  **91.7**  **91.7**  **91.7**  **91.7**  **91.6**  **91.7**  **91.7**  **91.7**  **91.7**  **91.8**  **91.7**  **91.8**  **91.6**  **91.6**  **91.7**  **91.8**  **91.7**  **91.7**  **91.7**  **91.8**  **91.6**  **91.7**  **91.8**  **91.7**  **91.8**  **91.7**  **91.7**  **91.8**  **91.8**  **91.7**  **91.7**  **91.7**  **91.7**  **91.7**  **91.7**  **91.7**  **91.8**  **91.7** | **100.5**  **100.5**  **100.5**  **100.6**  **100.6**  **100.5**  **100.5**  **100.6**  **100.6**  **100.5**  **100.6**  **100.6**  **100.6**  **100.6**  **100.4**  **100.5**  **100.6**  **100.6**  **100.6**  **100.6**  **100.6**  **100.6**  **100.5**  **100.6**  **100.6**  **100.6**  **100.7**  **100.6**  **100.6**  **100.6**  **100.7**  **100.5**  **100.6**  **100.5**  **100.5**  **100.6**  **100.5**  **100.6**  **100.6**  **100.6** | **541**  **1996**  **387**  **1534**  **1375**  **856**  **759**  **663**  **2800**  **997**  **3076**  **1828**  **588**  **2375**  **570**  **965**  **2198**  **371**  **2734**  **2010**  **192**  **1487**  **1613**  **835**  **521**  **2991**  **3106**  **550**  **2798**  **2251**  **1559**  **1565**  **4224**  **1885**  **1961**  **1202**  **1804**  **3494**  **3546**  **214** | **491**  **1326**  **559**  **872**  **855**  **206**  **578**  **348**  **1762**  **670**  **1805**  **1037**  **656**  **1339**  **442**  **481**  **1307**  **233**  **1516**  **1116**  **132**  **1011**  **740**  **779**  **328**  **1740**  **1742**  **344**  **1702**  **1276**  **776**  **678**  **1336**  **1067**  **1044**  **520**  **1324**  **2084**  **2042**  **212** | **4424**  **16097**  **3214**  **13888**  **12258**  **7220**  **6327**  **5856**  **23665**  **8704**  **26789**  **15517**  **5289**  **21171**  **4725**  **8175**  **18713**  **3088**  **24124**  **17898**  **1764**  **13131**  **13416**  **7757**  **4455**  **25631**  **27322**  **5012**  **23843**  **19989**  **13616**  **12342**  **35165**  **16917**  **17457**  **10558**  **15257**  **30019**  **30616**  **1842** | **3902**  **10702**  **4439**  **7338**  **7139**  **1601**  **4769**  **2949**  **14059**  **5523**  **14992**  **8624**  **5492**  **11274**  **3619**  **4072**  **10694**  **2032**  **13163**  **9656**  **1102**  **8580**  **5968**  **6619**  **2609**  **14816**  **14912**  **2905**  **14075**  **10739**  **6684**  **5418**  **11052**  **9323**  **8915**  **4256**  **10780**  **17016**  **17044**  **1559** | **2925**  **2909**  **2900**  **2944**  **2936**  **2953**  **2905**  **2944**  **2950**  **2940**  **3019**  **3036**  **3051**  **3114**  **2921**  **2999**  **3016**  **3092**  **3015**  **3032**  **3045**  **3105**  **2951**  **3027**  **3044**  **3057**  **3115**  **3008**  **3038**  **3072**  **3137**  **2988**  **3014**  **3033**  **3030**  **3064**  **2964**  **3013**  **3046**  **3043** | **3039**  **3022**  **3013**  **3059**  **3051**  **3067**  **3019**  **3059**  **3065**  **3054**  **3135**  **3153**  **3169**  **3233**  **3034**  **3115**  **3133**  **3210**  **3131**  **3148**  **3163**  **3223**  **3066**  **3143**  **3161**  **3174**  **3234**  **3124**  **3155**  **3190**  **3257**  **3103**  **3130**  **3149**  **3145**  **3182**  **3078**  **3129**  **3163**  **3161** |

| **Day** | **Count** | **Run Date** | **Sample Name** | **Marker** | **Allele 1** | **Allele 2** | **Size 1** | **Size 2** | **Height 1** | **Height 2** | **Peak Area 1** | **Peak Area 2** | **Data Point 1** | **Data Point 2** |
| --- | --- | --- | --- | --- | --- | --- | --- | --- | --- | --- | --- | --- | --- | --- |
| **3** | **41**  **42**  **43** | **7/13/05**  **7/18/05**  **7/18/05** | **CD015B1_M65**  **CD015B1M20**  **CD015B1M28** | **D5S346**  **D5S346**  **D5S346** | **92**  **92**  **92** | **101**  **101**  **101** | **91.8**  **91.8**  **91.8** | **100.6**  **100.6**  **100.6** | **1138**  **933**  **1011** | **936**  **546**  **513** | **9983**  **8367**  **8481** | **7998**  **4648**  **4125** | **3075**  **2932**  **2944** | **3193**  **3045**  **3058** |
| **1**  **2**  **3** | **1**  **2**  **3**  **4**  **5**  **6**  **7**  **8**  **9**  **10**  **11**  **12**  **13**  **14**  **15**  **16**  **17**  **18**  **19**  **20**  **21**  **22**  **23**  **24**  **25**  **26**  **27**  **28**  **29**  **30**  **31**  **32**  **33**  **34**  **35**  **36**  **37**  **38**  **39**  **40**  **41**  **42**  **43** | **7/11/05**  **7/11/05**  **7/11/05**  **7/11/05**  **7/11/05**  **7/11/05**  **7/11/05**  **7/11/05**  **7/11/05**  **7/13/05**  **7/13/05**  **7/13/05**  **7/13/05**  **7/13/05**  **7/13/05**  **7/13/05**  **7/13/05**  **7/13/05**  **7/13/05**  **7/13/05**  **7/13/05**  **7/13/05**  **7/13/05**  **7/13/05**  **7/13/05**  **7/13/05**  **7/13/05**  **7/13/05**  **7/13/05**  **7/13/05**  **7/13/05**  **7/13/05**  **7/13/05**  **7/13/05**  **7/13/05**  **7/13/05**  **7/13/05**  **7/13/05**  **7/13/05**  **7/13/05**  **7/13/05**  **7/18/05**  **7/18/05** | **CD015B1_M12**  **CD015B1_M1**  **CD015B1_M13* CD015B1_M2**  **CD015B1_M14**  **CD015B1_M3**  **CD015B1_M5**  **CD015B1_M9**  **CD015B1_M10**  **CD015B1_M17**  **CD015B1_M30**  **CD015B1_M42**  **CD015B1_M54**  **CD015B1_M66**  **CD015B1_M19**  **CD015B1_M31**  **CD015B1_M44**  **CD015B1_M67**  **CD015B1_M32**  **CD015B1_M46**  **CD015B1_M56**  **CD015B1_M70**  **CD015B1_M21**  **CD015B1_M34**  **CD015B1_M48**  **CD015B1_M57**  **CD015B1_M72**  **CD015B1_M23**  **CD015B1_M36**  **CD015B1_M58**  **CD015B1_M73**  **CD015B1_M26**  **CD015B1_M50**  **CD015B1_M38**  **CD015B1_M51**  **CD015B1_M64**  **CD015B1_M15**  **CD015B1_M29**  **CD015B1_M39**  **CD015B1_M53**  **CD015B1_M65**  **CD015B1M20**  **CD015B1M28** | **D6S262**  **D6S262**  **D6S262* D6S262**  **D6S262**  **D6S262**  **D6S262**  **D6S262**  **D6S262**  **D6S262**  **D6S262**  **D6S262**  **D6S262**  **D6S262**  **D6S262**  **D6S262**  **D6S262**  **D6S262**  **D6S262**  **D6S262**  **D6S262**  **D6S262**  **D6S262**  **D6S262**  **D6S262**  **D6S262**  **D6S262**  **D6S262**  **D6S262**  **D6S262**  **D6S262**  **D6S262**  **D6S262**  **D6S262**  **D6S262**  **D6S262**  **D6S262**  **D6S262**  **D6S262**  **D6S262**  **D6S262**  **D6S262**  **D6S262** | **184**  **184**  **184**  **184**  **184**  **184**  **184**  **184**  **184**  **184**  **184**  **184**  **184**  **184**  **184**  **184**  **184**  **184**  **184**  **184**  **184**  **184**  **184**  **184**  **184**  **184**  **184**  **184**  **184**  **184**  **184**  **184**  **184**  **184**  **184**  **184**  **184**  **184**  **184**  **184**  **184**  **184**  **184** | **186**  **186**  **186**  **186**  **186**  **186**  **186**  **186**  **186**  **186**  **186**  **186**  **186**  **186**  **186**  **186**  **186**  **186**  **186**  **186**  **186**  **186**  **186**  **186**  **186**  **186**  **186**  **186**  **186**  **186**  **186**  **186**  **186**  **186**  **186**  **186**  **186**  **186**  **186**  **186**  **186**  **186** | **184.0**  **183.8**  **183.9**  **183.8**  **183.9**  **183.9**  **183.9**  **183.9**  **183.9**  **183.8**  **184.0**  **183.9**  **183.9**  **184.0**  **183.8**  **183.8**  **183.9**  **183.9**  **183.8**  **183.9**  **183.9**  **184.0**  **183.8**  **183.9**  **183.9**  **183.9**  **184.0**  **183.9**  **184.0**  **183.9**  **184.0**  **183.9**  **184.0**  **183.8**  **183.9**  **183.9**  **183.9**  **183.9**  **183.9**  **183.9**  **184.0**  **183.9**  **183.9** | **185.8**  **185.8**  **185.8**  **185.9**  **185.8**  **185.8**  **185.8**  **185.8**  **185.8**  **185.9**  **185.8**  **185.9**  **185.9**  **185.8**  **185.8**  **185.8**  **185.9**  **185.8**  **185.9**  **185.8**  **186.0**  **185.8**  **185.8**  **185.8**  **185.9**  **185.9**  **185.9**  **185.9**  **185.9**  **185.9**  **185.8**  **185.8**  **185.8**  **185.8**  **185.8**  **185.8**  **185.8**  **185.9**  **185.8**  **185.9**  **185.7**  **185.8** | **1244**  **1900**  **436**  **1713**  **1705**  **628**  **1321**  **802**  **3277**  **759**  **3161**  **2035**  **1204**  **2823**  **733**  **1419**  **2754**  **311**  **3585**  **2442**  **744**  **1902**  **1555**  **906**  **563**  **2338**  **3639**  **1079**  **2887**  **2673**  **2010**  **954**  **2011**  **2602**  **2502**  **1653**  **2758**  **3531**  **4160**  **178**  **1251**  **756**  **369** | **601**  **994**  **723**  **1032**  **354**  **758**  **392**  **2050**  **385**  **1959**  **1227**  **581**  **1708**  **422**  **783**  **1550**  **146**  **2240**  **1456**  **696**  **1087**  **758**  **477**  **318**  **1642**  **2301**  **432**  **1619**  **1670**  **1188**  **348**  **1117**  **1532**  **1498**  **1082**  **1847**  **2176**  **2552**  **98**  **623**  **460**  **279** | **10296**  **15345**  **3319**  **14467**  **13428**  **5009**  **10456**  **6765**  **26435**  **6044**  **25578**  **17288**  **10372**  **23968**  **5912**  **11816**  **22548**  **2690**  **28956**  **20253**  **5884**  **15736**  **12756**  **7690**  **4536**  **19564**  **31031**  **9312**  **24197**  **22839**  **17867**  **7865**  **16460**  **21447**  **20837**  **13795**  **22217**  **29014**  **34550**  **1415**  **10684**  **5993**  **2896** | **4559**  **7811**  **5828**  **7786**  **2786**  **5491**  **3100**  **15239**  **2836**  **15079**  **9713**  **4576**  **13737**  **3129**  **5853**  **12257**  **1134**  **17084**  **11094**  **5784**  **8784**  **5694**  **3721**  **2187**  **12939**  **18339**  **3353**  **12292**  **13110**  **9736**  **2651**  **8768**  **11855**  **11485**  **8727**  **13889**  **16431**  **19562**  **657**  **4742**  **3468**  **2002** | **4084**  **4059**  **4048**  **4104**  **4093**  **4118**  **4056**  **4104**  **4115**  **4098**  **4199**  **4221**  **4241**  **4320**  **4072**  **4172**  **4194**  **4291**  **4187**  **4209**  **4226**  **4303**  **4112**  **4206**  **4228**  **4247**  **4320**  **4183**  **4223**  **4267**  **4352**  **4158**  **4190**  **4210**  **4205**  **4251**  **4124**  **4189**  **4231**  **4227**  **4268**  **4081**  **4099** | **4107**  **4083**  **4129**  **4118**  **4142**  **4080**  **4128**  **4139**  **4123**  **4223**  **4245**  **4266**  **4345**  **4096**  **4197**  **4218**  **4317**  **4212**  **4234**  **4251**  **4328**  **4136**  **4230**  **4253**  **4272**  **4345**  **4208**  **4247**  **4292**  **4377**  **4182**  **4213**  **4235**  **4229**  **4275**  **4148**  **4214**  **4256**  **4251**  **4293**  **4104**  **4123** |
| **1**  **2** | **1**  **2**  **3**  **4**  **5**  **6**  **7**  **8**  **9**  **10**  **11**  **12** | **7/11/05**  **7/11/05**  **7/11/05**  **7/11/05**  **7/11/05**  **7/11/05**  **7/11/05**  **7/11/05**  **7/11/05**  **7/13/05**  **7/13/05**  **7/13/05** | **CD015B1_M12**  **CD015B1_M1**  **CD015B1_M13**  **CD015B1_M2**  **CD015B1_M14**  **CD015B1_M3**  **CD015B1_M5**  **CD015B1_M9**  **CD015B1_M10**  **CD015B1_M17**  **CD015B1_M30**  **CD015B1_M42** | **D7S481**  **D7S481**  **D7S481**  **D7S481**  **D7S481**  **D7S481**  **D7S481**  **D7S481**  **D7S481**  **D7S481**  **D7S481**  **D7S481** | **187**  **187**  **187**  **187**  **187**  **187**  **187**  **187**  **187**  **187**  **187**  **187** | **197**  **197**  **197**  **197**  **197**  **197**  **197**  **197**  **197**  **197**  **197**  **197** | **187.3**  **187.1**  **187.2**  **187.3**  **187.2**  **187.2**  **187.3**  **187.2**  **187.3**  **187.2**  **187.3**  **187.3** | **196.9**  **196.8**  **196.8**  **196.8**  **196.9**  **196.9**  **196.8**  **196.8**  **196.9**  **196.8**  **196.8**  **196.9** | **408**  **513**  **234**  **395**  **564**  **383**  **527**  **689**  **1065**  **476**  **711**  **630** | **329**  **378**  **127**  **649**  **438**  **574**  **406**  **305**  **756**  **281**  **576**  **610** | **3595**  **4673**  **2072**  **3556**  **4963**  **3553**  **4751**  **6308**  **9869**  **4317**  **6748**  **5907** | **2810**  **3273**  **1229**  **6224**  **4051**  **5224**  **3578**  **2789**  **6713**  **2434**  **5465**  **5637** | **4125**  **4100**  **4089**  **4147**  **4135**  **4160**  **4098**  **4146**  **4157**  **4140**  **4241**  **4264** | **4245**  **4219**  **4207**  **4265**  **4255**  **4281**  **4216**  **4265**  **4277**  **4259**  **4362**  **4386** |

| **Day** | **Count** | **Run Date** | **Sample Name** | **Marker** | **Allele 1** | **Allele 2** | **Size 1** | **Size 2** | **Height 1** | **Height 2** | **Peak Area 1** | **Peak Area 2** | **Data Point 1** | **Data Point 2** |
| --- | --- | --- | --- | --- | --- | --- | --- | --- | --- | --- | --- | --- | --- | --- |
| **3** | **13**  **14**  **15**  **16**  **17**  **18**  **19**  **20**  **21**  **22**  **23**  **24**  **25**  **26**  **27**  **28**  **29**  **30**  **31**  **32**  **33**  **34**  **35**  **36**  **37**  **38**  **39**  **40**  **41**  **42**  **43** | **7/13/05**  **7/13/05**  **7/13/05**  **7/13/05**  **7/13/05**  **7/13/05**  **7/13/05**  **7/13/05**  **7/13/05**  **7/13/05**  **7/13/05**  **7/13/05**  **7/13/05**  **7/13/05**  **7/13/05**  **7/13/05**  **7/13/05**  **7/13/05**  **7/13/05**  **7/13/05**  **7/13/05**  **7/13/05**  **7/13/05**  **7/13/05**  **7/13/05**  **7/13/05**  **7/13/05**  **7/13/05**  **7/13/05**  **7/18/05**  **7/18/05** | **CD015B1_M54**  **CD015B1_M66**  **CD015B1_M19**  **CD015B1_M31**  **CD015B1_M44**  **CD015B1_M67**  **CD015B1_M32**  **CD015B1_M46**  **CD015B1_M56**  **CD015B1_M70**  **CD015B1_M21**  **CD015B1_M34**  **CD015B1_M48* CD015B1_M57**  **CD015B1_M72**  **CD015B1_M23**  **CD015B1_M36**  **CD015B1_M58**  **CD015B1_M73**  **CD015B1_M26**  **CD015B1_M50**  **CD015B1_M38**  **CD015B1_M51**  **CD015B1_M64**  **CD015B1_M15**  **CD015B1_M29**  **CD015B1_M39**  **CD015B1_M53**  **CD015B1_M65**  **CD015B1M20**  **CD015B1M28** | **D7S481**  **D7S481**  **D7S481**  **D7S481**  **D7S481**  **D7S481**  **D7S481**  **D7S481**  **D7S481**  **D7S481**  **D7S481**  **D7S481**  **D7S481* D7S481**  **D7S481**  **D7S481**  **D7S481**  **D7S481**  **D7S481**  **D7S481**  **D7S481**  **D7S481**  **D7S481**  **D7S481**  **D7S481**  **D7S481**  **D7S481**  **D7S481**  **D7S481**  **D7S481**  **D7S481** | **187**  **187**  **187**  **187**  **187**  **187**  **187**  **187**  **187**  **187**  **187**  **187**  **187**  **187**  **187**  **187**  **187**  **187**  **187**  **187**  **187**  **187**  **187**  **187**  **187**  **187**  **187**  **187**  **187**  **187**  **187** | **197**  **197**  **197**  **197**  **197**  **197**  **197**  **197**  **197**  **197**  **197**  **197**  **197**  **197**  **197**  **197**  **197**  **197**  **197**  **197**  **197**  **197**  **197**  **197**  **197**  **197**  **197**  **197**  **197**  **197**  **197** | **187.3**  **187.4**  **187.2**  **187.3**  **187.3**  **187.3**  **187.3**  **187.3**  **187.2**  **187.4**  **187.2**  **187.3**  **187.3**  **187.3**  **187.5**  **187.3**  **187.4**  **187.3**  **187.4**  **187.3**  **187.3**  **187.2**  **187.3**  **187.2**  **187.2**  **187.3**  **187.3**  **187.3**  **187.4**  **187.2**  **187.2** | **196.9**  **196.9**  **196.7**  **196.9**  **196.8**  **197.0**  **196.8**  **196.9**  **196.9**  **197.0**  **196.8**  **196.9**  **196.9**  **196.9**  **197.0**  **196.9**  **196.9**  **197.0**  **196.9**  **196.8**  **197.0**  **196.8**  **196.8**  **196.9**  **196.8**  **196.8**  **196.9**  **196.9**  **197.0**  **196.8**  **196.9** | **414**  **676**  **311**  **394**  **707**  **575**  **1062**  **608**  **272**  **582**  **690**  **383**  **734**  **611**  **899**  **536**  **894**  **804**  **845**  **355**  **427**  **744**  **513**  **534**  **841**  **856**  **995**  **145**  **500**  **205**  **127** | **421**  **541**  **208**  **384**  **457**  **587**  **825**  **440**  **182**  **451**  **389**  **153**  **130**  **451**  **783**  **319**  **676**  **632**  **659**  **273**  **272**  **568**  **460**  **340**  **920**  **619**  **771**  **201**  **492**  **145**  **96** | **3799**  **6502**  **2782**  **3501**  **6420**  **5457**  **9900**  **5854**  **2649**  **5565**  **6260**  **3304**  **6910**  **5830**  **8691**  **4952**  **8466**  **7915**  **8297**  **3177**  **3851**  **6827**  **4890**  **4955**  **7602**  **7965**  **9322**  **1254**  **4531**  **1845**  **1057** | **3857**  **5225**  **1772**  **3398**  **4195**  **5396**  **7508**  **4026**  **1785**  **4253**  **3408**  **1461**  **1227**  **3913**  **7375**  **2893**  **6392**  **5900**  **6366**  **2471**  **2466**  **5129**  **4259**  **3095**  **8267**  **5673**  **7148**  **1773**  **4434**  **1263**  **827** | **4285**  **4364**  **4113**  **4215**  **4237**  **4335**  **4230**  **4252**  **4269**  **4347**  **4154**  **4249**  **4271**  **4291**  **4365**  **4226**  **4266**  **4310**  **4396**  **4200**  **4232**  **4253**  **4248**  **4293**  **4166**  **4233**  **4274**  **4270**  **4312**  **4122**  **4141** | **4407**  **4488**  **4231**  **4336**  **4358**  **4459**  **4350**  **4373**  **4392**  **4470**  **4273**  **4371**  **4393**  **4413**  **4489**  **4347**  **4388**  **4434**  **4521**  **4320**  **4354**  **4374**  **4368**  **4416**  **4285**  **4353**  **4396**  **4392**  **4435**  **4240**  **4260** |
| **1** | **1**  **1**  **1**  **1**  **1**  **1** | **11/14/05**  **11/14/05**  **11/14/05**  **11/14/05**  **11/14/05**  **11/14/05** | **CD0016B1M1**  **CD0016B1M1**  **CD0016B1M1**  **CD0016B1M1**  **CD0016B1M1**  **CD0016B1M1** | **BAT25**  **BAT26**  **D3S3623**  **D5S346**  **D6S262**  **D7S481** | **128**  **122**  **219**  **103**  **172**  **187** | **221**  **105**  **182**  **203** | **127.8**  **121.7**  **218.6**  **102.6**  **172.2**  **187.2** | **220.7**  **104.7**  **181.9**  **202.7** | **4171**  **1593**  **4873**  **2572**  **4175**  **1463** | **3141**  **1664**  **2786**  **973** | **31042**  **16572**  **43810**  **20188**  **40554**  **15695** | **27096**  **11602**  **26544**  **10232** | **3624**  **3542**  **4757**  **3279**  **4181**  **4375** | **4782**  **3308**  **4306**  **4572** |
| **1**  **2**  **3**  **4**  **5**  **6**  **7**  **8**  **9**  **10**  **11** | **1**  **2**  **3**  **4**  **5**  **6**  **7**  **8**  **9**  **10**  **11**  **12**  **13**  **14**  **15**  **16**  **17**  **18**  **19**  **20** | **5/31/05**  **6/6/05**  **6/6/05**  **6/8/05**  **6/8/05**  **6/15/05**  **6/15/05**  **6/16/05**  **6/16/05**  **6/16/05**  **6/20/05**  **6/30/05**  **6/30/05**  **7/5/05**  **7/6/05**  **7/7/05**  **7/7/05**  **7/8/05**  **7/8/05**  **7/8/05** | **G1WT7**  **G1WT.5**  **G1WT.25**  **G1WT.5_1-2**  **G1WT.25_1-2**  **.2N1.5M.025T_1-2**  **.2N1.5M.05T_1-3**  **.2N1.5M.025T_1-2**  **.8N6M.1T_1-4**  **.2N1.5M.05T_1-2**  **0.25uM CTRL6-19-05**  **CTRL CTRL_61905**  **CTRL CRTL CRTL**  **CTRL_.4UL CTRL_1UL CTRL** | **BAT25**  **BAT25**  **BAT25**  **BAT25**  **BAT25**  **BAT25**  **BAT25**  **BAT25**  **BAT25**  **BAT25**  **BAT25**  **BAT25**  **BAT25**  **BAT25**  **BAT25**  **BAT25**  **BAT25**  **BAT25**  **BAT25**  **BAT25** | **127**  **127**  **127**  **127**  **127**  **127**  **127**  **127**  **127**  **127**  **127**  **127**  **127**  **127**  **127**  **127**  **127**  **127**  **127**  **127** | **128**  **128**  **128**  **128**  **128** | **127.1**  **126.7**  **126.6**  **126.8**  **126.9**  **127.0**  **126.9**  **127.0**  **126.9**  **127.1**  **126.9**  **126.7**  **126.7**  **126.7**  **126.8**  **126.7**  **126.7**  **126.8**  **126.8**  **127.0** | **128.0**  **128.0**  **128.0**  **127.9**  **128.1** | **1469**  **663**  **263**  **2249**  **1314**  **1919**  **1918**  **1987**  **1432**  **2304**  **727**  **985**  **2381**  **747**  **1129**  **2118**  **2010**  **2275**  **1545**  **1872** | **1989**  **1957**  **2018**  **1470**  **2310** | **10830**  **5152**  **2799**  **21059**  **10249**  **14389**  **14664**  **14730**  **10112**  **17886**  **5247**  **6884**  **17490**  **5655**  **8755**  **16416**  **15591**  **17311**  **11774**  **14395** | **15304**  **14354**  **15253**  **10151**  **17580** | **3649**  **3369**  **3379**  **3548**  **3557**  **3567**  **3506**  **3670**  **3560**  **3638**  **3445**  **3268**  **3311**  **3337**  **3381**  **3371**  **3397**  **3436**  **3413**  **3419** | **3581**  **3520**  **3684**  **3574**  **3652** |

| **Day** | **Count** | **Run Date** | **Sample Name** | **Marker** | **Allele 1** | **Allele 2** | **Size 1** | **Size 2** | **Height 1** | **Height 2** | **Peak Area 1** | **Peak Area 2** | **Data Point 1** | **Data Point 2** |
| --- | --- | --- | --- | --- | --- | --- | --- | --- | --- | --- | --- | --- | --- | --- |
| **12**  **13**  **14**  **15** | **21**  **22**  **23**  **24** | **7/11/05**  **7/13/05**  **7/15/05**  **11/14/05** | **CTRL CTRL CTRL**  **10K_CTRL** | **BAT25**  **BAT25**  **BAT25**  **BAT25** | **127**  **127**  **127**  **127** |  | **126.8**  **126.9**  **126.9**  **126.8** |  | **4069**  **5731**  **3589**  **4616** |  | **33614**  **46618**  **27723**  **37024** |  | **3407**  **3685**  **3454**  **3610** |  |
| **1**  **2**  **3**  **4**  **5**  **6**  **7**  **8**  **9**  **10**  **11**  **12**  **13**  **14**  **15** | **1**  **2**  **3**  **4**  **5**  **6**  **7**  **8**  **9**  **10**  **11**  **12**  **13**  **14**  **15**  **16**  **17**  **18**  **19**  **20**  **21**  **22**  **23**  **24** | **5/31/05**  **6/6/05**  **6/6/05**  **6/8/05**  **6/8/05**  **6/15/05**  **6/15/05**  **6/16/05**  **6/16/05**  **6/16/05**  **6/20/05**  **6/30/05**  **6/30/05**  **7/5/05**  **7/6/05**  **7/7/05**  **7/7/05**  **7/8/05**  **7/8/05**  **7/8/05**  **7/11/05**  **7/13/05**  **7/15/05**  **11/14/05** | **G1WT7**  **G1WT.5**  **G1WT.25**  **G1WT.5_1-2**  **G1WT.25_1-2**  **.2N1.5M.025T_1-2**  **.2N1.5M.05T_1-3**  **.2N1.5M.025T_1-2**  **.8N6M.1T_1-4**  **.2N1.5M.05T_1-2**  **0.25uM CTRL6-19-05**  **CTRL CTRL_61905**  **CTRL CRTL CRTL**  **CTRL_.4UL CTRL_1UL CTRL CTRL CTRL CTRL**  **10K_CTRL** | **BAT26**  **BAT26**  **BAT26**  **BAT26**  **BAT26**  **BAT26**  **BAT26**  **BAT26**  **BAT26**  **BAT26**  **BAT26**  **BAT26**  **BAT26**  **BAT26**  **BAT26**  **BAT26**  **BAT26**  **BAT26**  **BAT26**  **BAT26**  **BAT26**  **BAT26**  **BAT26**  **BAT26** | **122**  **122**  **122**  **122**  **122**  **122**  **122**  **122**  **122**  **122**  **122**  **122**  **122**  **122**  **122**  **122**  **122**  **122**  **122**  **122**  **122**  **122**  **122**  **122** |  | **122.0**  **121.8**  **121.6**  **121.7**  **121.8**  **122.0**  **121.9**  **121.9**  **121.8**  **122.0**  **121.8**  **121.6**  **121.6**  **121.6**  **121.6**  **121.7**  **121.6**  **121.7**  **121.8**  **121.8**  **121.8**  **121.9**  **121.8**  **121.6** |  | **245**  **161**  **143**  **539**  **730**  **411**  **585**  **456**  **380**  **761**  **257**  **330**  **623**  **289**  **403**  **676**  **591**  **764**  **623**  **478**  **1138**  **1887**  **781**  **1458** |  | **2028**  **1348**  **1546**  **5668**  **5854**  **3426**  **4755**  **3855**  **3153**  **6455**  **2204**  **2700**  **5510**  **2438**  **3090**  **5949**  **5268**  **6679**  **5338**  **4714**  **11605**  **16461**  **7002**  **15395** |  | **3580**  **3306**  **3315**  **3480**  **3489**  **3500**  **3440**  **3600**  **3491**  **3569**  **3379**  **3204**  **3247**  **3273**  **3315**  **3306**  **3331**  **3370**  **3348**  **3353**  **3342**  **3616**  **3388**  **3542** |  |
| **1**  **2**  **3**  **4**  **5**  **6**  **7**  **8**  **9**  **10**  **11**  **12**  **13**  **14**  **15** | **1**  **2**  **3**  **4**  **5**  **6**  **7**  **8**  **9**  **10**  **11**  **12**  **13**  **14**  **15**  **16**  **17**  **18**  **19**  **20**  **21**  **22**  **23**  **24** | **5/31/05**  **6/6/05**  **6/6/05**  **6/8/05**  **6/8/05**  **6/15/05**  **6/15/05**  **6/16/05**  **6/16/05**  **6/16/05**  **6/20/05**  **6/30/05**  **6/30/05**  **7/5/05**  **7/6/05**  **7/7/05**  **7/7/05**  **7/8/05**  **7/8/05**  **7/8/05**  **7/11/05**  **7/13/05**  **7/15/05**  **11/14/05** | **G1WT7**  **G1WT.5**  **G1WT.25**  **G1WT.5_1-2**  **G1WT.25_1-2**  **.2N1.5M.025T_1-2**  **.2N1.5M.05T_1-3**  **.2N1.5M.025T_1-2**  **.8N6M.1T_1-4**  **.2N1.5M.05T_1-2**  **0.25uM CTRL6-19-05**  **CTRL CTRL_61905**  **CTRL CRTL CRTL**  **CTRL_.4UL CTRL_1UL CTRL CTRL CTRL CTRL**  **10K_CTRL** | **D3S3623**  **D3S3623**  **D3S3623**  **D3S3623**  **D3S3623**  **D3S3623**  **D3S3623**  **D3S3623**  **D3S3623**  **D3S3623**  **D3S3623**  **D3S3623**  **D3S3623**  **D3S3623**  **D3S3623**  **D3S3623**  **D3S3623**  **D3S3623**  **D3S3623**  **D3S3623**  **D3S3623**  **D3S3623**  **D3S3623**  **D3S3623** | **219**  **219**  **219**  **219**  **219**  **219**  **219**  **219**  **219**  **219**  **219**  **219**  **219**  **219**  **219**  **219**  **219**  **219**  **219**  **219**  **219**  **219**  **219**  **219** | **225**  **225**  **225**  **225**  **225**  **225**  **225**  **225**  **225**  **225**  **225**  **225**  **225**  **225**  **225**  **225**  **225**  **225**  **225**  **225**  **225**  **225**  **225**  **225** | **219.1**  **218.3**  **218.2**  **218.8**  **218.9**  **219.1**  **219.0**  **219.1**  **218.7**  **218.9**  **218.5**  **218.4**  **218.4**  **218.5**  **218.6**  **218.5**  **218.6**  **218.4**  **218.4**  **218.7**  **218.6**  **218.9**  **218.5**  **218.5** | **225.4**  **224.6**  **224.7**  **225.0**  **225.1**  **225.4**  **225.3**  **225.4**  **224.9**  **225.2**  **224.8**  **224.6**  **224.6**  **224.7**  **224.8**  **224.8**  **224.8**  **224.6**  **224.6**  **224.9**  **224.8**  **225.1**  **224.7**  **224.8** | **1752**  **703**  **256**  **2025**  **1614**  **408**  **591**  **447**  **900**  **679**  **680**  **838**  **1818**  **626**  **859**  **1569**  **1609**  **1648**  **1219**  **1230**  **2670**  **3657**  **2300**  **4249** | **1406**  **507**  **218**  **1515**  **1302**  **278**  **507**  **258**  **592**  **619**  **558**  **674**  **1380**  **489**  **575**  **1058**  **1068**  **1128**  **1015**  **920**  **1733**  **2300**  **1516**  **3346** | **16055**  **5941**  **3454**  **25725**  **14966**  **3611**  **4970**  **3959**  **7937**  **6468**  **5708**  **7007**  **15146**  **5055**  **7291**  **13786**  **13738**  **14113**  **10539**  **10660**  **23518**  **34439**  **19393**  **40681** | **12464**  **4435**  **3067**  **19273**  **11782**  **2318**  **4316**  **2445**  **5417**  **5533**  **4572**  **5714**  **11489**  **4100**  **4927**  **9131**  **9194**  **9679**  **8477**  **7973**  **15295**  **21880**  **13017**  **31627** | **4813**  **4460**  **4479**  **4692**  **4704**  **4717**  **4638**  **4856**  **4739**  **4813**  **4560**  **4340**  **4387**  **4429**  **4481**  **4476**  **4509**  **4548**  **4519**  **4532**  **4519**  **4863**  **4570**  **4751** | **4887**  **4530**  **4552**  **4765**  **4776**  **4791**  **4709**  **4932**  **4814**  **4889**  **4632**  **4409**  **4455**  **4498**  **4551**  **4547**  **4580**  **4619**  **4589**  **4603**  **4590**  **4938**  **4641**  **4824** |
| **1**  **2**  **3**  **4** | **1**  **2**  **3**  **4**  **5**  **6** | **5/31/05**  **6/6/05**  **6/6/05**  **6/8/05**  **6/8/05**  **6/15/05** | **G1WT7**  **G1WT.5**  **G1WT.25**  **G1WT.5_1-2**  **G1WT.25_1-2**  **.2N1.5M.025T_1-2** | **D5S346**  **D5S346**  **D5S346**  **D5S346**  **D5S346**  **D5S346** | **92**  **92**  **92**  **92**  **92**  **92** | **94**  **94**  **94**  **94**  **94**  **94** | **91.9**  **91.6**  **91.6**  **91.8**  **91.8**  **92.0** | **94.2**  **93.8**  **93.8**  **94.0**  **94.0**  **94.1** | **1824**  **1186**  **403**  **4136**  **1745**  **1808** | **1360**  **804**  **270**  **2713**  **1199**  **1096** | **14359**  **9197**  **3788**  **35028**  **13486**  **14291** | **9900**  **5930**  **2431**  **21329**  **8868**  **7996** | **3163**  **2912**  **2919**  **3071**  **3079**  **3089** | **3193**  **2940**  **2947**  **3101**  **3109**  **3118** |

| **Day** | **Count** | **Run Date** | **Sample Name** | **Marker** | **Allele 1** | **Allele 2** | **Size 1** | **Size 2** | **Height 1** | **Height 2** | **Peak Area 1** | **Peak Area 2** | **Data Point 1** | **Data Point 2** |
| --- | --- | --- | --- | --- | --- | --- | --- | --- | --- | --- | --- | --- | --- | --- |
| **5**  **6**  **7**  **8**  **9**  **10**  **11**  **12**  **13**  **14**  **15** | **7**  **8**  **9**  **10**  **11**  **12**  **13**  **14**  **15**  **16**  **17**  **18**  **19**  **20**  **21**  **22**  **23**  **24** | **6/15/05**  **6/16/05**  **6/16/05**  **6/16/05**  **6/20/05**  **6/30/05**  **6/30/05**  **7/5/05**  **7/6/05**  **7/7/05**  **7/7/05**  **7/8/05**  **7/8/05**  **7/8/05**  **7/11/05**  **7/13/05**  **7/15/05**  **11/14/05** | **.2N1.5M.05T_1-3**  **.2N1.5M.025T_1-2**  **.8N6M.1T_1-4***  **.2N1.5M.05T_1-2**  **0.25uM CTRL6-19-05* CTRL CTRL_61905* CTRL**  **CRTL CRTL CTRL_.4UL CTRL_1UL CTRL**  **CTRL CTRL CTRL**  **10K_CTRL** | **D5S346**  **D5S346**  **D5S346* D5S346**  **D5S346**  **D5S346* D5S346**  **D5S346* D5S346**  **D5S346**  **D5S346**  **D5S346**  **D5S346**  **D5S346**  **D5S346**  **D5S346**  **D5S346**  **D5S346** | **92**  **92**  **92**  **92**  **92**  **92**  **92**  **92**  **92**  **92**  **92**  **92**  **92**  **92**  **92**  **92**  **92**  **92** | **94**  **94**  **94**  **94**  **94**  **94**  **94**  **94**  **94**  **94**  **94**  **94**  **94**  **94**  **94**  **94**  **94**  **94** | **91.9**  **91.9**  **91.7**  **91.8**  **91.7**  **91.6**  **91.7**  **91.6**  **91.6**  **91.6**  **91.6**  **91.7**  **91.6**  **91.8**  **91.7**  **91.8**  **91.7**  **91.6** | **94.1**  **94.1**  **94.0**  **94.0**  **93.9**  **93.8**  **93.9**  **93.8**  **93.9**  **93.8**  **93.8**  **93.9**  **93.9**  **94.0**  **93.9**  **94.1**  **93.9**  **93.8** | **1623**  **1984**  **1125**  **2085**  **576**  **716**  **1757**  **588**  **866**  **1459**  **1347**  **2165**  **1958**  **1719**  **2960**  **4008**  **2275**  **3463** | **1033**  **1177**  **622**  **1281**  **355**  **430**  **1120**  **344**  **526**  **870**  **826**  **1520**  **1301**  **1214**  **1973**  **2669**  **1456**  **2450** | **12860**  **16548**  **10169**  **17194**  **5128**  **6340**  **13701**  **5008**  **6555**  **11505**  **10549**  **16970**  **15299**  **13007**  **23957**  **33131**  **17591**  **27117** | **7754**  **9036**  **4778**  **9708**  **2431**  **2954**  **8418**  **2452**  **3711**  **6426**  **5921**  **11338**  **9417**  **8721**  **15129**  **21154**  **10976**  **17460** | **3035**  **3178**  **3073**  **3149**  **2978**  **2819**  **2860**  **2880**  **2920**  **2910**  **2933**  **2970**  **2950**  **2954**  **2944**  **3194**  **2987**  **3133** | **3064**  **3208**  **3104**  **3179**  **3007**  **2847**  **2888**  **2909**  **2949**  **2938**  **2962**  **2999**  **2979**  **2983**  **2973**  **3225**  **3016**  **3163** |
| **1**  **2**  **3**  **4**  **5**  **6**  **7**  **8**  **9**  **10**  **11**  **12**  **13**  **14**  **15** | **1**  **2**  **3**  **4**  **5**  **6**  **7**  **8**  **9**  **10**  **11**  **12**  **13**  **14**  **15**  **16**  **17**  **18**  **19**  **20**  **21**  **22**  **23**  **24** | **5/31/05**  **6/6/05**  **6/6/05**  **6/8/05**  **6/8/05**  **6/15/05**  **6/15/05**  **6/16/05**  **6/16/05**  **6/16/05**  **6/20/05**  **6/30/05**  **6/30/05**  **7/5/05**  **7/6/05**  **7/7/05**  **7/7/05**  **7/8/05**  **7/8/05**  **7/8/05**  **7/11/05**  **7/13/05**  **7/15/05**  **11/14/05** | **G1WT7**  **G1WT.5**  **G1WT.25**  **G1WT.5_1-2**  **G1WT.25_1-2**  **.2N1.5M.025T_1-2**  **.2N1.5M.05T_1-3**  **.2N1.5M.025T_1-2**  **.8N6M.1T_1-4**  **.2N1.5M.05T_1-2**  **0.25uM CTRL6-19-05**  **CTRL CTRL_61905**  **CTRL CRTL CRTL**  **CTRL_.4UL CTRL_1UL CTRL CTRL CTRL CTRL**  **10K_CTRL** | **D6S262**  **D6S262**  **D6S262**  **D6S262**  **D6S262**  **D6S262**  **D6S262**  **D6S262**  **D6S262**  **D6S262**  **D6S262**  **D6S262**  **D6S262**  **D6S262**  **D6S262**  **D6S262**  **D6S262**  **D6S262**  **D6S262**  **D6S262**  **D6S262**  **D6S262**  **D6S262**  **D6S262** | **176**  **176**  **176**  **176**  **176**  **176**  **176**  **176**  **176**  **176**  **176**  **176**  **176**  **176**  **176**  **176**  **176**  **176**  **176**  **176**  **176**  **176**  **176**  **176** | **182**  **182**  **182**  **182**  **182**  **182**  **182**  **182**  **182**  **182**  **182**  **182**  **182**  **182**  **182**  **182**  **182**  **182**  **182**  **182**  **182**  **182**  **182**  **182** | **176.5**  **176.0**  **176.0**  **176.3**  **176.3**  **176.4**  **176.4**  **176.5**  **176.2**  **176.4**  **176.2**  **176.0**  **176.1**  **176.1**  **176.1**  **176.1**  **176.0**  **176.1**  **176.1**  **176.3**  **176.1**  **176.3**  **176.0**  **176.1** | **182.3**  **181.8**  **181.8**  **182.0**  **182.1**  **182.3**  **182.2**  **182.3**  **182.1**  **182.2**  **182.0**  **181.8**  **181.9**  **181.8**  **181.9**  **181.9**  **181.9**  **181.8**  **181.9**  **182.1**  **182.0**  **182.1**  **181.8**  **181.9** | **346**  **438**  **296**  **1145**  **1591**  **837**  **1061**  **859**  **587**  **1276**  **538**  **645**  **1498**  **528**  **815**  **1361**  **1248**  **1512**  **1323**  **2745**  **2788**  **4046**  **1794**  **3600** | **263**  **300**  **223**  **795**  **1219**  **581**  **802**  **577**  **412**  **908**  **363**  **428**  **1294**  **358**  **600**  **1013**  **955**  **1223**  **1039**  **2232**  **1493**  **2217**  **1531**  **2678** | **2871**  **3393**  **3819**  **13509**  **15317**  **6881**  **8759**  **7836**  **5217**  **11245**  **4486**  **5407**  **12349**  **4544**  **6843**  **11316**  **10699**  **12362**  **10662**  **23608**  **26082**  **38496**  **15557**  **35279** | **2075**  **2380**  **2831**  **8896**  **11115**  **4633**  **6277**  **5144**  **3584**  **8021**  **2712**  **3169**  **10220**  **2667**  **4836**  **7981**  **7606**  **9814**  **8310**  **18880**  **14002**  **20814**  **12905**  **24676** | **4279**  **3961**  **3977**  **4168**  **4178**  **4189**  **4119**  **4312**  **4197**  **4274**  **4049**  **3849**  **3895**  **3929**  **3977**  **3970**  **3999**  **4039**  **4013**  **4022**  **4009**  **4322**  **4058**  **4228** | **4355**  **4032**  **4048**  **4242**  **4253**  **4265**  **4193**  **4389**  **4275**  **4351**  **4122**  **3919**  **3965**  **4000**  **4049**  **4042**  **4072**  **4111**  **4085**  **4095**  **4082**  **4399**  **4131**  **4302** |
| **1**  **2**  **3**  **4**  **5**  **6**  **7**  **8**  **9**  **10** | **1**  **2**  **3**  **4**  **5**  **6**  **7**  **8**  **9**  **10**  **11**  **12**  **13**  **14**  **15**  **16** | **5/31/05**  **6/6/05**  **6/6/05**  **6/8/05**  **6/8/05**  **6/15/05**  **6/15/05**  **6/16/05**  **6/16/05**  **6/16/05**  **6/20/05**  **6/30/05**  **6/30/05**  **7/5/05**  **7/6/05**  **7/7/05** | **G1WT7**  **G1WT.5**  **G1WT.25**  **G1WT.5_1-2**  **G1WT.25_1-2**  **.2N1.5M.025T_1-2**  **.2N1.5M.05T_1-3**  **.2N1.5M.025T_1-2**  **.8N6M.1T_1-4**  **.2N1.5M.05T_1-2**  **0.25uM CTRL6-19-05**  **CTRL CTRL_61905**  **CTRL CRTL** | **D7S481**  **D7S481**  **D7S481**  **D7S481**  **D7S481**  **D7S481**  **D7S481**  **D7S481**  **D7S481**  **D7S481**  **D7S481**  **D7S481**  **D7S481**  **D7S481**  **D7S481**  **D7S481** | **187**  **187**  **187**  **187**  **187**  **187**  **187**  **187**  **187**  **187**  **187**  **187**  **187**  **187**  **187**  **187** | **193**  **193**  **193**  **193**  **193**  **193**  **193**  **193**  **193**  **193**  **193**  **193**  **193**  **193**  **193**  **193** | **187.6**  **187.1**  **187.1**  **187.3**  **187.4**  **187.6**  **187.4**  **187.6**  **187.4**  **187.6**  **187.3**  **187.0**  **187.2**  **187.1**  **187.2**  **187.2** | **193.4**  **192.9**  **192.7**  **193.2**  **193.2**  **193.4**  **193.3**  **193.3**  **193.2**  **193.3**  **193.0**  **192.8**  **192.9**  **192.9**  **192.9**  **192.9** | **421**  **230**  **156**  **699**  **920**  **212**  **438**  **226**  **254**  **522**  **338**  **376**  **738**  **325**  **348**  **620** | **417**  **191**  **139**  **503**  **780**  **177**  **322**  **190**  **230**  **379**  **237**  **276**  **657**  **231**  **289**  **483** | **3767**  **2054**  **2053**  **8559**  **9241**  **1911**  **3821**  **2192**  **2357**  **5081**  **2819**  **3339**  **6308**  **2757**  **3220**  **5649** | **3794**  **1535**  **1811**  **6200**  **7703**  **1707**  **2824**  **1818**  **2269**  **3487**  **1962**  **2382**  **5623**  **1937**  **2490**  **4425** | **4425**  **4097**  **4114**  **4310**  **4322**  **4334**  **4260**  **4460**  **4345**  **4422**  **4188**  **3982**  **4029**  **4065**  **4114**  **4108** | **4500**  **4168**  **4184**  **4386**  **4396**  **4409**  **4334**  **4537**  **4422**  **4498**  **4260**  **4051**  **4098**  **4136**  **4185**  **4179** |

| **Day** | **Count** | **Run Date** | **Sample Name** | **Marker** | **Allele 1** | **Allele 2** | **Size 1** | **Size 2** | **Height 1** | **Height 2** | **Peak Area 1** | **Peak Area 2** | **Data Point 1** | **Data Point 2** |
| --- | --- | --- | --- | --- | --- | --- | --- | --- | --- | --- | --- | --- | --- | --- |
| **11**  **12**  **13**  **14**  **15** | **17**  **18**  **19**  **20**  **21**  **22**  **23**  **24** | **7/7/05**  **7/8/05**  **7/8/05**  **7/8/05**  **7/11/05**  **7/13/05**  **7/15/05**  **11/14/05** | **CRTL CTRL_.4UL CTRL_1UL CTRL**  **CTRL CTRL CTRL**  **10K_CTRL** | **D7S481**  **D7S481**  **D7S481**  **D7S481**  **D7S481**  **D7S481**  **D7S481**  **D7S481** | **187**  **187**  **187**  **187**  **187**  **187**  **187**  **187** | **193**  **193**  **193**  **193**  **193**  **193**  **193**  **193** | **187.2**  **187.2**  **187.2**  **187.4**  **187.2**  **187.5**  **187.2**  **187.2** | **193.0**  **192.9**  **192.9**  **193.1**  **193.0**  **193.2**  **192.9**  **192.9** | **615**  **627**  **478**  **829**  **660**  **998**  **845**  **1877** | **480**  **564**  **374**  **706**  **622**  **927**  **740**  **1505** | **5584**  **5437**  **4130**  **8324**  **6739**  **9684**  **7381**  **20115** | **4370**  **4934**  **3259**  **6878**  **6677**  **9236**  **6701**  **16043** | **4139**  **4178**  **4151**  **4161**  **4148**  **4470**  **4198**  **4370** | **4211**  **4250**  **4222**  **4233**  **4220**  **4546**  **4270**  **4444** |
| **1**  **2**  **3**  **4** | **1**  **2**  **3**  **4**  **5**  **6**  **7**  **8**  **9**  **10**  **11** | **5/31/05**  **5/31/05**  **5/31/05**  **5/31/05**  **5/31/05**  **5/31/05**  **6/6/05**  **6/6/05**  **6/8/05**  **6/8/05**  **7/14/05** | **G1MF7_10**  **G1MF7_15**  **G1MF7_20**  **G1MF7_25**  **G1MF10**  **G1MF15**  **G1MF10**  **G1MF.5**  **G1MF.5_1-9**  **G1MF.25_1-6**  **MF** | **BAT25**  **BAT25**  **BAT25**  **BAT25**  **BAT25**  **BAT25**  **BAT25**  **BAT25**  **BAT25**  **BAT25**  **BAT25** | **126**  **126**  **126**  **126**  **126**  **126**  **126**  **126**  **126**  **126**  **126** |  | **126.1**  **126.0**  **126.1**  **125.9**  **126.0**  **126.0**  **125.8**  **125.7**  **125.9**  **125.9**  **125.9** |  | **2508**  **2072**  **3760**  **1246**  **820**  **1013**  **752**  **1758**  **2334**  **1584**  **3137** |  | **18819**  **15553**  **28514**  **9276**  **6444**  **7578**  **7783**  **12869**  **17630**  **12078**  **24882** |  | **3623**  **3633**  **3648**  **3526**  **3516**  **3532**  **3369**  **3366**  **3516**  **3554**  **3498** |  |
| **1**  **2**  **3**  **4** | **1**  **2**  **3**  **4**  **5**  **6**  **7**  **8**  **9**  **10**  **11** | **5/31/05**  **5/31/05**  **5/31/05**  **5/31/05**  **5/31/05**  **5/31/05**  **6/6/05**  **6/6/05**  **6/8/05**  **6/8/05**  **7/14/05** | **G1MF7_10**  **G1MF7_15**  **G1MF7_20**  **G1MF7_25**  **G1MF10**  **G1MF15**  **G1MF10**  **G1MF.5**  **G1MF.5_1-9**  **G1MF.25_1-6**  **MF** | **BAT26**  **BAT26**  **BAT26**  **BAT26**  **BAT26**  **BAT26**  **BAT26**  **BAT26**  **BAT26**  **BAT26**  **BAT26** | **121**  **121**  **121**  **121**  **121**  **121**  **121**  **121**  **121**  **121**  **121** |  | **121.1**  **121.0**  **121.1**  **120.9**  **120.9**  **120.9**  **120.8**  **120.7**  **120.9**  **120.9**  **120.9** |  | **409**  **383**  **605**  **196**  **165**  **175**  **115**  **281**  **439**  **663**  **871** |  | **2912**  **2731**  **4426**  **1287**  **1121**  **1247**  **981**  **2128**  **3206**  **4526**  **7016** |  | **3556**  **3565**  **3579**  **3460**  **3449**  **3465**  **3305**  **3301**  **3450**  **3487**  **3432** |  |
| **1**  **2**  **3**  **4** | **1**  **2**  **3**  **4**  **5**  **6**  **7**  **8**  **9**  **10**  **11** | **5/31/05**  **5/31/05**  **5/31/05**  **5/31/05**  **5/31/05**  **5/31/05**  **6/6/05**  **6/6/05**  **6/8/05**  **6/8/05**  **7/14/05** | **G1MF7_10**  **G1MF7_15**  **G1MF7_20**  **G1MF7_25**  **G1MF10**  **G1MF15**  **G1MF10**  **G1MF.5**  **G1MF.5_1-9**  **G1MF.25_1-6**  **MF** | **D3S3623**  **D3S3623**  **D3S3623**  **D3S3623**  **D3S3623**  **D3S3623**  **D3S3623**  **D3S3623**  **D3S3623**  **D3S3623**  **D3S3623** | **219**  **219**  **219**  **219**  **219**  **219**  **219**  **219**  **219**  **219**  **219** | **225**  **225**  **225**  **225**  **225**  **225**  **225**  **225**  **225**  **225**  **225** | **219.0**  **219.0**  **219.1**  **218.8**  **218.8**  **218.9**  **218.2**  **218.4**  **218.8**  **218.8**  **218.6** | **225.4**  **225.4**  **225.4**  **225.1**  **225.2**  **225.1**  **224.6**  **224.7**  **225.2**  **225.2**  **224.9** | **1657**  **1801**  **3130**  **1617**  **1227**  **1248**  **591**  **2088**  **2598**  **1868**  **5926** | **1315**  **1297**  **2188**  **1224**  **978**  **945**  **399**  **1435**  **1799**  **1385**  **4360** | **14507**  **16236**  **28361**  **14366**  **10863**  **11003**  **7475**  **17354**  **23822**  **16530**  **54764** | **11512**  **11306**  **19382**  **10229**  **8501**  **8162**  **5118**  **11841**  **16278**  **12128**  **40174** | **4798**  **4810**  **4833**  **4677**  **4664**  **4684**  **4480**  **4478**  **4672**  **4722**  **4644** | **4874**  **4885**  **4908**  **4750**  **4738**  **4756**  **4552**  **4548**  **4746**  **4797**  **4717** |
| **1**  **2**  **3**  **4** | **1**  **2**  **3**  **4**  **5**  **6**  **7**  **8**  **9**  **10**  **11** | **5/31/05**  **5/31/05**  **5/31/05**  **5/31/05**  **5/31/05**  **5/31/05**  **6/6/05**  **6/6/05**  **6/8/05**  **6/8/05**  **7/14/05** | **G1MF7_10**  **G1MF7_15**  **G1MF7_20**  **G1MF7_25**  **G1MF10**  **G1MF15**  **G1MF10**  **G1MF.5**  **G1MF.5_1-9**  **G1MF.25_1-6**  **MF** | **D5S346**  **D5S346**  **D5S346**  **D5S346**  **D5S346**  **D5S346**  **D5S346**  **D5S346**  **D5S346**  **D5S346**  **D5S346** | **92**  **92**  **92**  **92**  **92**  **92**  **92**  **92**  **92**  **92**  **92** | **109**  **109**  **109**  **109**  **109**  **109**  **109**  **109**  **109**  **109**  **109** | **91.9**  **91.9**  **92.0**  **91.9**  **91.8**  **91.9**  **91.7**  **91.6**  **91.7**  **91.8**  **91.7** | **109.1**  **109.1**  **109.1**  **108.9**  **109.0**  **109.0**  **108.8**  **108.8**  **108.9**  **109.0**  **108.9** | **2489**  **2369**  **3779**  **1355**  **1197**  **1352**  **1106**  **2191**  **3388**  **1552**  **2156** | **945**  **986**  **1531**  **723**  **685**  **698**  **529**  **1021**  **1531**  **695**  **1205** | **21574**  **20694**  **33213**  **11464**  **10001**  **11787**  **10713**  **18005**  **29452**  **12847**  **18432** | **7696**  **8462**  **12955**  **5590**  **5557**  **5797**  **5264**  **8096**  **12437**  **5669**  **9819** | **3152**  **3161**  **3174**  **3065**  **3055**  **3071**  **2921**  **2919**  **3053**  **3087**  **3038** | **3389**  **3399**  **3412**  **3297**  **3287**  **3303**  **3147**  **3145**  **3287**  **3323**  **3270** |
| **1** | **1**  **2**  **3**  **4**  **5** | **5/31/05**  **5/31/05**  **5/31/05**  **5/31/05**  **5/31/05** | **G1MF7_10**  **G1MF7_15**  **G1MF7_20**  **G1MF7_25**  **G1MF10** | **D6S262**  **D6S262**  **D6S262**  **D6S262**  **D6S262** | **172**  **172**  **172**  **172**  **172** | **186**  **186**  **186**  **186**  **186** | **172.7**  **172.6**  **172.6**  **172.5**  **172.4** | **186.2**  **186.1**  **186.1**  **185.9**  **186.0** | **1023**  **885**  **1428**  **344**  **322** | **596**  **533**  **864**  **249**  **212** | **8276**  **7453**  **11986**  **2594**  **2562** | **4781**  **4327**  **7265**  **1879**  **1675** | **4215**  **4226**  **4244**  **4106**  **4094** | **4392**  **4403**  **4422**  **4279**  **4267** |

| **Day** | **Count** | **Run Date** | **Sample Name** | **Marker** | **Allele 1** | **Allele 2** | **Size 1** | **Size 2** | **Height 1** | **Height 2** | **Peak Area 1** | **Peak Area 2** | **Data Point 1** | **Data Point 2** |
| --- | --- | --- | --- | --- | --- | --- | --- | --- | --- | --- | --- | --- | --- | --- |
| **2**  **3**  **4** | **6**  **7**  **8**  **9**  **10**  **11** | **5/31/05**  **6/6/05**  **6/6/05**  **6/8/05**  **6/8/05**  **7/14/05** | **G1MF15**  **G1MF10**  **G1MF.5**  **G1MF.5_1-9**  **G1MF.25_1-6**  **MF** | **D6S262**  **D6S262**  **D6S262**  **D6S262**  **D6S262**  **D6S262** | **172**  **172**  **172**  **172**  **172**  **172** | **186**  **186**  **186**  **186**  **186**  **186** | **172.5**  **172.2**  **172.2**  **172.4**  **172.4**  **172.4** | **186.0**  **185.6**  **185.7**  **185.9**  **185.9**  **185.8** | **427**  **350**  **1047**  **1488**  **1909**  **2873** | **262**  **203**  **691**  **923**  **1087**  **2044** | **3441**  **4023**  **8362**  **12773**  **16904**  **27778** | **2136**  **2430**  **5234**  **8060**  **9259**  **18709** | **4112**  **3931**  **3927**  **4098**  **4142**  **4076** | **4286**  **4097**  **4094**  **4272**  **4318**  **4248** |
| **1**  **2**  **3**  **4** | **1**  **2**  **3**  **4**  **5**  **6**  **7**  **8**  **9**  **10**  **11** | **5/31/05**  **5/31/05**  **5/31/05**  **5/31/05**  **5/31/05**  **5/31/05**  **6/6/05**  **6/6/05**  **6/8/05**  **6/8/05**  **7/14/05** | **G1MF7_10**  **G1MF7_15**  **G1MF7_20**  **G1MF7_25**  **G1MF10**  **G1MF15**  **G1MF10**  **G1MF.5**  **G1MF.5_1-9**  **G1MF.25_1-6**  **MF** | **D7S481**  **D7S481**  **D7S481**  **D7S481**  **D7S481**  **D7S481**  **D7S481**  **D7S481**  **D7S481**  **D7S481**  **D7S481** | **187**  **187**  **187**  **187**  **187**  **187**  **187**  **187**  **187**  **187**  **187** | **197**  **197**  **197**  **197**  **197**  **197**  **197**  **197**  **197**  **197**  **197** | **187.6**  **187.5**  **187.6**  **187.4**  **187.4**  **187.4**  **187.2**  **187.1**  **187.5**  **187.4**  **187.2** | **197.3**  **197.2**  **197.3**  **197.0**  **197.0**  **197.0**  **196.7**  **196.8**  **197.0**  **197.1**  **196.9** | **410**  **470**  **704**  **402**  **347**  **353**  **163**  **499**  **721**  **964**  **3234** | **278**  **319**  **507**  **320**  **252**  **257**  **126**  **351**  **506**  **658**  **2262** | **3735**  **4133**  **6447**  **3554**  **2999**  **3058**  **1902**  **4403**  **6733**  **9009**  **31635** | **2539**  **2774**  **4631**  **2680**  **2129**  **2133**  **1467**  **2964**  **4656**  **6166**  **22110** | **4411**  **4422**  **4442**  **4298**  **4285**  **4304**  **4116**  **4112**  **4292**  **4337**  **4266** | **4537**  **4548**  **4569**  **4421**  **4408**  **4426**  **4234**  **4231**  **4415**  **4463**  **4389** |
| **1** | **1**  **1**  **1** | **06-06-05-GS**  **06-06-05-GS**  **06-06-05-GS** | **G1BF53WT3**  **G1BF53M3**  **G1BF53M4** | **BAT25**  **BAT25**  **BAT25** | **126**  **127**  **127** | **127** | **125.9**  **126.9**  **126.8** | **127.0** | **2193**  **2576**  **2946** | **2137** | **17698**  **19203**  **27563** | **16849** | **3569**  **3582**  **3456** | **3583** |
|  | **1**  **1**  **1** | **06-06-05-GS**  **06-06-05-GS**  **06-06-05-GS** | **G1BF53WT3**  **G1BF53M3**  **G1BF53M4** | **BAT26**  **BAT26**  **BAT26** | **121**  **122**  **122** | **123** | **120.9**  **121.9**  **121.7** | **122.6** | **494**  **438**  **344** | **354** | **4304**  **3386**  **3344** | **2950** | **3502**  **3515**  **3390** | **3402** |
|  | **1**  **1**  **1** | **06-06-05-GS**  **06-06-05-GS**  **06-06-05-GS** | **G1BF53WT3**  **G1BF53M3**  **G1BF53M4** | **D3S3623**  **D3S3623**  **D3S3623** | **219**  **217**  **217** | **221**  **225**  **225** | **218.8**  **216.8**  **216.4** | **220.9**  **225.1**  **224.8** | **3789**  **2683**  **1787** | **2203**  **1824**  **1441** | **41714**  **25228**  **21623** | **22853**  **17296**  **18060** | **4730**  **4707**  **4548** | **4755**  **4804**  **4643** |
|  | **1**  **1**  **1** | **06-06-05-GS**  **06-06-05-GS**  **06-06-05-GS** | **G1BF53WT3**  **G1BF53M3**  **G1BF53M4** | **D5S346**  **D5S346**  **D5S346** | **103**  **92**  **92** | **107**  **96**  **96** | **102.7**  **91.8**  **91.6** | **106.9**  **96.3**  **96.1** | **1864**  **2680**  **3291** | **1455**  **1848**  **2115** | **16847**  **22754**  **30336** | **12468**  **15188**  **19237** | **3251**  **3103**  **2988** | **3309**  **3163**  **3047** |
|  | **1**  **1**  **1** | **06-06-05-GS**  **06-06-05-GS**  **06-06-05-GS** | **G1BF53WT3**  **G1BF53M3**  **G1BF53M4** | **D6S262**  **D6S262**  **D6S262** | **180**  **179**  **179** | **182**  **184**  **184** | **180.2**  **179.2**  **179.0** | **182.1**  **184.0**  **183.8** | **1209**  **1239**  **1124** | **744**  **863**  **686** | **12097**  **11463**  **13157** | **6402**  **7680**  **7257** | **4255**  **4243**  **4098** | **4280**  **4305**  **4158** |
|  | **1**  **1**  **1** | **06-06-05-GS**  **06-06-05-GS**  **06-06-05-GS** | **G1BF53WT3**  **G1BF53M3**  **G1BF53M4** | **D7S481**  **D7S481**  **D7S481** | **193**  **187**  **187** | **203**  **193**  **193** | **193.1**  **187.3**  **187.1** | **203.0**  **193.2**  **193.0** | **511**  **681**  **447** | **419**  **509**  **481** | **5834**  **6780**  **5293** | **4665**  **4948**  **5620** | **4422**  **4347**  **4199** | **4546**  **4423**  **4273** |
|  | **1**  **1**  **1** | **06-06-05-GS**  **06-06-05-GS**  **06-06-05-GS** | **G1CS72M1**  **G1CS72M2**  **G1CS72M6** | **BAT25**  **BAT25**  **BAT25** | **126**  **126**  **126** |  | **125.7**  **125.7**  **125.7** |  | **3013**  **1394**  **4354** |  | **23265**  **12614**  **34967** |  | **3401**  **3415**  **3405** |  |
|  | **1**  **1**  **1** | **06-06-05-GS**  **06-06-05-GS**  **06-06-05-GS** | **G1CS72M1**  **G1CS72M2**  **G1CS72M6** | **BAT26**  **BAT26**  **BAT26** | **111**  **122**  **111** |  | **110.6**  **121.7**  **110.7** |  | **310**  **371**  **712** |  | **3428**  **4595**  **8122** |  | **3204**  **3363**  **3208** |  |
|  | **1**  **1**  **1** | **06-06-05-GS**  **06-06-05-GS**  **06-06-05-GS** | **G1CS72M1**  **G1CS72M2**  **G1CS72M6** | **D3S3623**  **D3S3623**  **D3S3623** | **217**  **219**  **217** | **225**  **225**  **225** | **216.3**  **218.3**  **216.3** | **224.8**  **224.6**  **224.8** | **1624**  **1413**  **3423** | **1392**  **775**  **2539** | **15161**  **20128**  **31410** | **12467**  **10873**  **23503** | **4494**  **4539**  **4498** | **4589**  **4611**  **4594** |
|  | **1**  **1**  **1** | **06-06-05-GS**  **06-06-05-GS**  **06-06-05-GS** | **G1CS72M1**  **G1CS72M2**  **G1CS72M6** | **D5S346**  **D5S346**  **D5S346** | **92**  **94**  **92** | **107**  **111**  **107** | **91.6**  **93.9**  **91.7** | **106.7**  **110.8**  **106.7** | **2679**  **2611**  **4104** | **1246**  **1265**  **2256** | **22220**  **25210**  **33277** | **9958**  **12663**  **17984** | **2952**  **2992**  **2956** | **3151**  **3219**  **3155** |
|  | **1**  **1**  **1** | **06-06-05-GS**  **06-06-05-GS**  **06-06-05-GS** | **G1CS72M1**  **G1CS72M2**  **G1CS72M6** | **D6S262**  **D6S262**  **D6S262** | **179**  **172**  **179** | **186**  **178**  **186** | **179.0**  **172.2**  **179.0** | **185.7**  **178.0**  **185.7** | **883**  **793**  **1967** | **578**  **472**  **1192** | **7497**  **9728**  **18097** | **4639**  **5592**  **9535** | **4050**  **3983**  **4054** | **4133**  **4056**  **4137** |
|  | **1**  **1**  **1** | **06-06-05-GS**  **06-06-05-GS**  **06-06-05-GS** | **G1CS72M1**  **G1CS72M2**  **G1CS72M6** | **D7S481**  **D7S481**  **D7S481** | **187**  **195**  **187** | **201**  **203**  **201** | **187.2**  **194.9**  **187.2** | **200.5**  **202.6**  **200.6** | **400**  **369**  **987** | **291**  **243**  **624** | **3831**  **5017**  **9591** | **2723**  **3343**  **5880** | **4151**  **4268**  **4155** | **4316**  **4361**  **4321** |
|  | **1**  **1**  **1**  **1** | **06-06-05-GS**  **06-06-05-GS**  **06-06-05-GS**  **06-06-05-GS** | **G1BF113WT1**  **G1BF113WT2**  **G1BF113M1**  **G1BF113M4** | **BAT25**  **BAT25**  **BAT25**  **BAT25** | **126**  **126**  **126**  **127** | **127**  **127** | **125.8**  **125.7**  **125.7**  **126.7** | **126.7**  **126.8** | **776**  **1910**  **4378**  **4521** | **1894**  **4175** | **6347**  **17994**  **34175**  **48283** | **18286**  **30643** | **3437**  **3416**  **3420**  **3438** | **3430**  **3433** |

| **Day** | **Count** | **Run Date** | **Sample Name** | **Marker** | **Allele 1** | **Allele 2** | **Size 1** | **Size 2** | **Height 1** | **Height 2** | **Peak Area 1** | **Peak Area 2** | **Data Point 1** | **Data Point 2** |
| --- | --- | --- | --- | --- | --- | --- | --- | --- | --- | --- | --- | --- | --- | --- |
|  | **1**  **1**  **1**  **1** | **06-06-05-GS**  **06-06-05-GS**  **06-06-05-GS**  **06-06-05-GS** | **G1BF113WT1**  **G1BF113WT2**  **G1BF113M1**  **G1BF113M4** | **BAT26**  **BAT26**  **BAT26**  **BAT26** | **121**  **122**  **122**  **122** |  | **120.7**  **121.7**  **121.6**  **121.7** |  | **121**  **460**  **911**  **729** |  | **965**  **4919**  **10154**  **8622** |  | **3371**  **3364**  **3367**  **3372** |  |
|  | **1**  **1**  **1**  **1** | **06-06-05-GS**  **06-06-05-GS**  **06-06-05-GS**  **06-06-05-GS** | **G1BF113WT1**  **G1BF113WT2**  **G1BF113M1**  **G1BF113M4** | **D3S3623**  **D3S3623**  **D3S3623**  **D3S3623** | **219**  **219**  **219**  **219** | **225**  **225**  **225**  **227** | **218.6**  **218.4**  **218.4**  **218.3** | **224.7**  **224.7**  **224.7**  **226.8** | **415**  **1574**  **3996**  **3065** | **284**  **1172**  **2967**  **1920** | **3836**  **20050**  **36639**  **42012** | **2577**  **14984**  **27394**  **26985** | **4563**  **4541**  **4541**  **4551** | **4633**  **4613**  **4612**  **4647** |
|  | **1**  **1**  **1**  **1** | **06-06-05-GS**  **06-06-05-GS**  **06-06-05-GS**  **06-06-05-GS** | **G1BF113WT1**  **G1BF113WT2**  **G1BF113M1**  **G1BF113M4** | **D5S346**  **D5S346**  **D5S346**  **D5S346** | **92**  **92**  **92**  **94** | **107**  **107**  **107**  **103** | **91.7**  **91.6**  **91.6**  **93.8** | **106.8**  **106.7**  **106.6**  **102.5** | **1465**  **3147**  **6445**  **5405** | **680**  **1455**  **2889**  **3327** | **12392**  **30562**  **56137**  **53673** | **5517**  **13953**  **23459**  **33533** | **2985**  **2963**  **2969**  **3000** | **3185**  **3164**  **3168**  **3115** |
|  | **1**  **1**  **1**  **1** | **06-06-05-GS**  **06-06-05-GS**  **06-06-05-GS**  **06-06-05-GS** | **G1BF113WT1**  **G1BF113WT2**  **G1BF113M1**  **G1BF113M4** | **D6S262**  **D6S262**  **D6S262**  **D6S262** | **182**  **182**  **182**  **178** | **182** | **181.9**  **181.9**  **181.9**  **178.0** | **181.9** | **405**  **1347**  **3989**  **1565** | **1125** | **3646**  **15225**  **33761**  **18248** | **12587** | **4125**  **4106**  **4106**  **4067** | **4115** |
|  | **1**  **1**  **1**  **1** | **06-06-05-GS**  **06-06-05-GS**  **06-06-05-GS**  **06-06-05-GS** | **G1BF113WT1**  **G1BF113WT2**  **G1BF113M1**  **G1BF113M4** | **D7S481**  **D7S481**  **D7S481**  **D7S481** | **187**  **187**  **187**  **187** | **193**  **193**  **193**  **201** | **187.2**  **187.2**  **187.2**  **187.1** | **193.0**  **193.0**  **192.9**  **200.5** | **148**  **434**  **1042**  **835** | **136**  **349**  **887**  **567** | **1431**  **5360**  **9823**  **11044** | **1282**  **4266**  **8361**  **7489** | **4192**  **4172**  **4172**  **4181** | **4264**  **4245**  **4244**  **4348** |
|  | **1**  **1**  **1**  **1** | **06-06-05-GS**  **06-06-05-GS**  **06-06-05-GS**  **06-06-05-GS** | **G1CS143WT6**  **G1CS143MC G1CS143M6**  **G1CS143M3** | **BAT25**  **BAT25**  **BAT25**  **BAT25** | **127**  **126**  **126**  **127** |  | **126.7**  **125.7**  **125.7**  **126.8** |  | **3774**  **1809**  **4293**  **3871** |  | **31397**  **16989**  **32345**  **40379** |  | **3391**  **3391**  **3381**  **3405** |  |
|  | **1**  **1**  **1**  **1** | **06-06-05-GS**  **06-06-05-GS**  **06-06-05-GS**  **06-06-05-GS** | **G1CS143WT6**  **G1CS143MC G1CS143M3**  **G1CS143M6** | **BAT26**  **BAT26**  **BAT26**  **BAT26** | **122**  **122**  **122**  **122** | **123**  **123** | **121.6**  **121.6**  **121.7**  **121.6** | **122.6**  **122.6** | **448**  **209**  **737**  **429** | **216**  **422** | **4786**  **1946**  **8747**  **4129** | **2053**  **3588** | **3325**  **3338**  **3340**  **3329** | **3350**  **3341** |
|  | **1**  **1**  **1**  **1** | **06-06-05-GS**  **06-06-05-GS**  **06-06-05-GS**  **06-06-05-GS** | **G1CS143WT6**  **G1CS143MC G1CS143M3**  **G1CS143M6** | **D3S3623**  **D3S3623**  **D3S3623**  **D3S3623** | **219**  **225**  **219**  **225** | **227**  **227** | **218.4**  **224.7**  **218.4**  **224.7** | **226.6**  **226.7** | **7260**  **1265**  **6213**  **4463** | **708**  **2649** | **66542**  **15791**  **88672**  **38782** | **7550**  **22036** | **4488**  **4581**  **4512**  **4562** | **4603**  **4585** |
|  | **1**  **1**  **1**  **1** | **06-06-05-GS**  **06-06-05-GS**  **06-06-05-GS**  **06-06-05-GS** | **G1CS143WT6**  **G1CS143MC G1CS143M3**  **G1CS143M6** | **D5S346**  **D5S346**  **D5S346**  **D5S346** | **89**  **92**  **89**  **92** | **96**  **107**  **96**  **107** | **89.4**  **91.6**  **89.4**  **91.6** | **96.1**  **106.7**  **96.0**  **106.7** | **3487**  **3928**  **6532**  **4420** | **2696**  **1341**  **4325**  **2045** | **29139**  **37447**  **65985**  **36965** | **21611**  **12831**  **44584**  **16676** | **2902**  **2941**  **2912**  **2934** | **2988**  **3140**  **2999**  **3132** |
|  | **1**  **1**  **1**  **1** | **06-06-05-GS**  **06-06-05-GS**  **06-06-05-GS**  **06-06-05-GS** | **G1CS143WT6**  **G1CS143MC G1CS143M3**  **G1CS143M6** | **D6S262**  **D6S262**  **D6S262**  **D6S262** | **170**  **176**  **170**  **176** | **184**  **182**  **184**  **182** | **170.3**  **176.0**  **170.3**  **176.1** | **183.9**  **181.8**  **183.8**  **181.9** | **1580**  **699**  **1797**  **1763** | **1027**  **506**  **1024**  **1192** | **13175**  **9052**  **22824**  **15130** | **8506**  **6157**  **13040**  **9434** | **3914**  **4004**  **3933**  **3990** | **4082**  **4076**  **4102**  **4062** |
|  | **1**  **1**  **1**  **1** | **06-06-05-GS**  **06-06-05-GS**  **06-06-05-GS**  **06-06-05-GS** | **G1CS143WT6**  **G1CS143MC G1CS143M3**  **G1CS143M6** | **D7S481**  **D7S481**  **D7S481**  **D7S481** | **199**  **193**  **199**  **193** | **203**  **203** | **198.7**  **192.9**  **198.6**  **192.9** | **202.6**  **202.8** | **1269**  **213**  **1064**  **839** | **142**  **660** | **11460**  **2754**  **14361**  **7875** | **1847**  **5893** | **4265**  **4214**  **4286**  **4197** | **4331**  **4316** |
| **1** | **1**  **2**  **3**  **4** | **11/14/05**  **11/14/05**  **11/14/05**  **11/14/05** | **S3_1_10**  **S2_1_10**  **S4_1_10**  **S1_1_10** | **BAT25**  **BAT25**  **BAT25**  **BAT25** | **127**  **126**  **126**  **126** | **127** | **126.9**  **125.9**  **125.9**  **125.9** | **126.9** | **126.9** | **5153**  **3801**  **3892**  **3823** | **3704** | **40079**  **29159**  **31671**  **27815** | **25857** | **3777**  **3802**  **3801**  **3805** |
| **1** | **1**  **2**  **3**  **4** | **11/14/05**  **11/14/05**  **11/14/05**  **11/14/05** | **S3_1_10**  **S2_1_10**  **S4_1_10**  **S1_1_10** | **BAT26**  **BAT26**  **BAT26**  **BAT26** | **122**  **122**  **122**  **122** |  | **121.8**  **121.8**  **121.8**  **121.8** |  |  | **505**  **546**  **501**  **403** |  | **4011**  **4679**  **4326**  **3472** |  | **3706**  **3745**  **3744**  **3748** |
| **1** | **1**  **2**  **3**  **4** | **11/14/05**  **11/14/05**  **11/14/05**  **11/14/05** | **S3_1_10**  **S2_1_10**  **S4_1_10**  **S1_1_10** | **D3S3623**  **D3S3623**  **D3S3623**  **D3S3623** | **219**  **225**  **219**  **219** | **227**  **227**  **227** | **218.6**  **225.2**  **219.0**  **218.9** | **227.3**  **227.4**  **227.3** | **227.3**  **227.4**  **227.3** | **7145**  **4141**  **3532**  **3825** | **2413**  **2248**  **2747** | **95578**  **41293**  **40741**  **38828** | **21651**  **25463**  **27496** | **4955**  **5085**  **5010**  **5015** |
| **1** | **1** | **11/14/05** | **S3_1_10** | **D5S346** | **89** | **92** | **89.6** | **91.8** | **91.8** | **2494** | **1922** | **19783** | **14171** | **3253** |

| **Day** | **Count** | **Run Date** | **Sample Name** | **Marker** | **Allele 1** | **Allele 2** | **Size 1** | **Size 2** | **Height 1** | **Height 2** | **Peak Area 1** | **Peak Area 2** | **Data Point 1** | **Data Point 2** |
| --- | --- | --- | --- | --- | --- | --- | --- | --- | --- | --- | --- | --- | --- | --- |
|  | **2**  **3**  **4** | **11/14/05**  **11/14/05**  **11/14/05** | **S2_1_10**  **S4_1_10**  **S1_1_10** | **D5S346**  **D5S346**  **D5S346** | **94**  **89**  **89** | **103**  **94**  **94** | **93.9**  **89.5**  **89.6** | **102.7**  **94.0**  **94.0** | **102.7**  **94.0**  **94.0** | **2038**  **1569**  **2084** | **993**  **1611**  **1805** | **17895**  **13213**  **17930** | **8418**  **14324**  **15816** | **3349**  **3286**  **3290** |
| **1** | **1**  **2**  **3**  **4** | **11/14/05**  **11/14/05**  **11/14/05**  **11/14/05** | **S3_1_10**  **S2_1_10**  **S4_1_10**  **S1_1_10** | **D6S262**  **D6S262**  **D6S262**  **D6S262** | **180**  **174**  **174**  **174** | **184**  **180**  **184**  **184** | **180.1**  **174.3**  **174.3**  **174.4** | **184.0**  **180.2**  **184.0**  **184.0** | **184.0**  **180.2**  **184.0**  **184.0** | **4164**  **3116**  **2434**  **2880** | **2576**  **2177**  **1916**  **2315** | **36930**  **30132**  **27645**  **26103** | **22370**  **22549**  **20702**  **22775** | **4467**  **4435**  **4435**  **4440** |
| **1** | **1**  **2**  **3**  **4** | **11/14/05**  **11/14/05**  **11/14/05**  **11/14/05** | **S3_1_10**  **S2_1_10**  **S4_1_10**  **S1_1_10** | **D7S481**  **D7S481**  **D7S481**  **D7S481** | **193**  **201**  **193**  **193** | **203**  **203**  **203**  **203** | **193.1**  **200.9**  **193.2**  **193.2** | **203.0**  **203.0**  **203.0**  **203.0** | **203.0**  **203.0**  **203.0**  **203.0** | **475**  **446**  **258**  **262** | **395**  **293**  **175**  **224** | **5543**  **4878**  **3290**  **2955** | **4458**  **2796**  **2179**  **2462** | **4640**  **4791**  **4689**  **4694** |

| **Sample Type** | **# of Patients** | **# of Marker** | **# of Alleles** |
| --- | --- | --- | --- |
| **UC** | **7** | **102** | **204** |
| **CD** | **12** | **1374** | **2748** |
| **Control Sample** | **1** | **144** | **288** |
| **Cell Line** | **1** | **66** | **132** |
| **Other Samples** | **4** | **84** | **168** |
| **Lineage Study** | **4** | **24** | **48** |
| **TOTAL** | **29** | **1794** | **3588** |

**Group II Microsatellite Markers D2S123, D3S1262, D9S171, D17S250, D18S61**

| **Day** | **Count** | **Run Date** | **Sample Name** | **Marker** | **Allele 1** | **Allele 2** | **Size 1** | **Size 2** | **Height 1** | **Height 2** | **Peak Area 1** | **Peak Area 2** | **Data Point 1** | **Data Point 2** |
| --- | --- | --- | --- | --- | --- | --- | --- | --- | --- | --- | --- | --- | --- | --- |
| **1** | **1**  **1**  **1**  **1**  **1** | **6/20/05**  **6/20/05**  **6/20/05**  **6/20/05**  **6/20/05** | **UC001B1WT1**  **UC001B1WT1**  **UC001B1WT1**  **UC001B1WT1**  **UC001B1WT1** | **D17S250**  **D18S61**  **D2S123**  **D3S1262**  **D9S171** | **189**  **154**  **219**  **136**  **113** | **191**  **170**  **236**  **140** | **189.5**  **154.1**  **218.6**  **135.9**  **113.2** | **191.5**  **170.0**  **235.9**  **140.0** | **1689**  **376**  **244**  **1851**  **2359** | **1019**  **280**  **307**  **1291** | **13318**  **3194**  **1909**  **19620**  **19779** | **7456**  **2068**  **2666**  **13422** | **4241**  **3797**  **4587**  **3581**  **3286** | **4266**  **3994**  **4786**  **3632** |
| **1** | **1**  **2**  **3**  **4**  **5** | **6/30/05**  **6/30/05**  **6/30/05**  **6/30/05**  **6/30/05** | **UC002B1M10**  **UC002B1M13**  **UC002B1M15**  **UC002B1M18**  **UC002B1M19** | **D17S250**  **D17S250**  **D17S250**  **D17S250**  **D17S250** | **193**  **193**  **193**  **193**  **193** | **200**  **200**  **200**  **200**  **200** | **193.6**  **193.6**  **193.5**  **193.4**  **193.6** | **199.7**  **199.7**  **199.7**  **199.6**  **199.7** | **1204**  **1806**  **3115**  **2027**  **3063** | **916**  **1342**  **2303**  **1533**  **2280** | **10108**  **14460**  **25171**  **16046**  **25301** | **7561**  **10949**  **18102**  **11864**  **18243** | **4236**  **4154**  **4194**  **4130**  **4169** | **4312**  **4229**  **4270**  **4205**  **4244** |
| **1** | **1**  **2**  **3**  **4**  **5** | **6/30/05**  **6/30/05**  **6/30/05**  **6/30/05**  **6/30/05** | **UC002B1M10**  **UC002B1M13**  **UC002B1M15**  **UC002B1M18**  **UC002B1M19** | **D18S61**  **D18S61**  **D18S61**  **D18S61**  **D18S61** | **152**  **152**  **152**  **152**  **152** | **168**  **168**  **168**  **168**  **168** | **152.0**  **151.8**  **151.9**  **151.8**  **151.9** | **168.0**  **168.0**  **168.0**  **167.9**  **168.0** | **866**  **786**  **1080**  **770**  **1081** | **444**  **468**  **590**  **441**  **610** | **7236**  **6288**  **8856**  **6188**  **8919** | **3596**  **3765**  **4988**  **3647**  **5001** | **3720**  **3646**  **3682**  **3625**  **3660** | **3916**  **3840**  **3877**  **3818**  **3853** |
| **1** | **1**  **2**  **3**  **4**  **5** | **6/30/05**  **6/30/05**  **6/30/05**  **6/30/05**  **6/30/05** | **UC002B1M10**  **UC002B1M13**  **UC002B1M15**  **UC002B1M18**  **UC002B1M19** | **D2S123**  **D2S123**  **D2S123**  **D2S123**  **D2S123** | **216**  **216**  **216**  **216**  **216** | **219**  **219**  **219**  **219**  **219** | **216.4**  **216.2**  **216.3**  **216.2**  **216.3** | **218.6**  **218.5**  **218.6**  **218.3**  **218.4** | **1229**  **1784**  **2100**  **1682**  **2328** | **708**  **1127**  **1324**  **1046**  **1457** | **10016**  **14189**  **16859**  **13259**  **18930** | **5320**  **7931**  **9591**  **7480**  **10656** | **4502**  **4414**  **4458**  **4390**  **4431** | **4527**  **4439**  **4483**  **4414**  **4455** |
| **1** | **1**  **2**  **3**  **4**  **5** | **6/30/05**  **6/30/05**  **6/30/05**  **6/30/05**  **6/30/05** | **UC002B1M10**  **UC002B1M13**  **UC002B1M15**  **UC002B1M18**  **UC002B1M19** | **D3S1262**  **D3S1262**  **D3S1262**  **D3S1262**  **D3S1262** | **136**  **136**  **136**  **136**  **136** | **138**  **138**  **138**  **138**  **138** | **135.9**  **135.8**  **135.8**  **135.8**  **135.7** | **137.8**  **137.9**  **137.8**  **137.8**  **137.8** | **1682**  **2630**  **3237**  **2281**  **3336** | **1261**  **2106**  **2519**  **1761**  **2588** | **16891**  **26474**  **34117**  **23082**  **34083** | **12360**  **20647**  **26249**  **17704**  **25385** | **3532**  **3461**  **3496**  **3441**  **3473** | **3556**  **3486**  **3520**  **3465**  **3498** |
| **1** | **1**  **2**  **3**  **4**  **5** | **6/30/05**  **6/30/05**  **6/30/05**  **6/30/05**  **6/30/05** | **UC002B1M10**  **UC002B1M13**  **UC002B1M15**  **UC002B1M18**  **UC002B1M19** | **D9S171**  **D9S171**  **D9S171**  **D9S171**  **D9S171** | **113**  **113**  **113**  **113**  **113** |  | **113.1**  **113.1**  **113.1**  **113.1**  **113.0** |  | **2447**  **3497**  **4357**  **2707**  **4302** |  | **20492**  **27711**  **35396**  **21879**  **35061** |  | **3239**  **3173**  **3205**  **3154**  **3184** |  |
| **1** | **1**  **1**  **1**  **1**  **1** | **6/20/05**  **6/20/05**  **6/20/05**  **6/20/05**  **6/20/05** | **UC003B1WT1**  **UC003B1WT1**  **UC003B1WT1**  **UC003B1WT1**  **UC003B1WT1** | **D17S250**  **D18S61**  **D2S123**  **D3S1262**  **D9S171** | **191**  **152**  **219**  **132**  **113** | **202**  **172**  **236**  **142** | **191.5**  **152.0**  **218.7**  **132.0**  **113.2** | **202.0**  **171.9**  **236.1**  **142.2** | **1407**  **838**  **658**  **2097**  **2744** | **954**  **437**  **590**  **1870** | **11723**  **6712**  **5386**  **21692**  **23895** | **7741**  **3454**  **5154**  **19773** | **4283**  **3786**  **4605**  **3544**  **3298** | **4413**  **4034**  **4806**  **3670** |
| **1** | **1**  **2**  **3** | **6/30/05**  **6/30/05**  **6/30/05** | **UC005B1M18**  **UC005B1M3**  **UC005B1M5** | **D17S250**  **D17S250**  **D17S250** | **191**  **191**  **191** | **195**  **195**  **195** | **191.4**  **191.4**  **191.4** | **195.5**  **195.5**  **195.5** | **1458**  **1299**  **1712** | **740**  **960**  **1230** | **11828**  **10024**  **13922** | **5669**  **7220**  **9368** | **4077**  **4095**  **4132** | **4126**  **4145**  **4182** |
| **1** | **1**  **2**  **3** | **6/30/05**  **6/30/05**  **6/30/05** | **UC005B1M18**  **UC005B1M3**  **UC005B1M5** | **D18S61**  **D18S61**  **D18S61** | **168**  **168**  **168** | **172**  **172**  **172** | **167.9**  **167.9**  **168.0** | **171.7**  **171.8**  **171.9** | **331**  **311**  **630** | **190**  **219**  **447** | **2604**  **2424**  **5028** | **1410**  **1572**  **3410** | **3791**  **3809**  **3843** | **3838**  **3856**  **3891** |
| **1** | **1**  **2**  **3** | **6/30/05**  **6/30/05**  **6/30/05** | **UC005B1M18**  **UC005B1M3**  **UC005B1M5** | **D2S123**  **D2S123**  **D2S123** | **219**  **219**  **219** | **236**  **236**  **236** | **218.4**  **218.4**  **218.5** | **235.8**  **235.7**  **235.9** | **990**  **650**  **873** | **689**  **643**  **885** | **8531**  **5211**  **7346** | **5836**  **5324**  **7476** | **4385**  **4404**  **4443** | **4577**  **4596**  **4637** |
| **1** | **1**  **2**  **3** | **6/30/05**  **6/30/05**  **6/30/05** | **UC005B1M18**  **UC005B1M3**  **UC005B1M5** | **D3S1262**  **D3S1262**  **D3S1262** | **136**  **136**  **136** |  | **135.7**  **135.8**  **135.8** |  | **2281**  **2714**  **3457** |  | **23772**  **26552**  **35388** |  | **3415**  **3433**  **3464** |  |
| **1** | **1**  **2**  **3** | **6/30/05**  **6/30/05**  **6/30/05** | **UC005B1M18**  **UC005B1M3**  **UC005B1M5** | **D9S171**  **D9S171**  **D9S171** | **105**  **105**  **105** | **113**  **113**  **113** | **105.4**  **105.5**  **105.5** | **113.0**  **113.1**  **113.1** | **984**  **1277**  **1797** | **920**  **907**  **1211** | **7637**  **10294**  **15010** | **7044**  **7415**  **10420** | **3030**  **3046**  **3075** | **3130**  **3146**  **3176** |
| **1** | **1**  **2** | **6/30/05**  **6/30/05** | **UC006B1M5**  **UC006B1M9** | **D17S250**  **D17S250** | **191**  **191** | **204**  **204** | **191.4**  **191.4** | **204.0**  **204.1** | **713**  **1552** | **432**  **1016** | **5762**  **12412** | **3369**  **7747** | **4111**  **4086** | **4261**  **4235** |

| **Day** | **Count** | **Run Date** | **Sample Name** | **Marker** | **Allele 1** | **Allele 2** | **Size 1** | **Size 2** | **Height 1** | **Height 2** | **Peak Area 1** | **Peak Area 2** | **Data Point 1** | **Data Point 2** |
| --- | --- | --- | --- | --- | --- | --- | --- | --- | --- | --- | --- | --- | --- | --- |
|  | **3** | **6/30/05** | **UC006B1M15** | **D17S250** | **191** | **204** | **191.4** | **204.0** | **878** | **551** | **7056** | **4284** | **4120** | **4270** |
| **1** | **1**  **2**  **3** | **6/30/05**  **6/30/05**  **6/30/05** | **UC006B1M5**  **UC006B1M9**  **UC006B1M15** | **D18S61**  **D18S61**  **D18S61** | **166**  **166**  **166** | **172**  **172**  **172** | **165.9**  **166.0**  **165.9** | **171.8**  **171.8**  **171.8** | **327**  **520**  **428** | **282**  **425**  **336** | **2655**  **4351**  **3416** | **2272**  **3228**  **2576** | **3798**  **3776**  **3807** | **3870**  **3847**  **3879** |
| **1** | **1**  **2**  **3** | **6/30/05**  **6/30/05**  **6/30/05** | **UC006B1M5**  **UC006B1M9**  **UC006B1M15** | **D2S123**  **D2S123**  **D2S123** | **219**  **219**  **219** |  | **218.4**  **218.4**  **218.5** |  | **3105**  **2407**  **2840** |  | **26905**  **20047**  **23885** |  | **4421**  **4394**  **4431** |  |
| **1** | **1**  **2**  **3** | **6/30/05**  **6/30/05**  **6/30/05** | **UC006B1M5**  **UC006B1M9**  **UC006B1M15** | **D3S1262**  **D3S1262**  **D3S1262** | **132**  **132**  **132** | **142**  **142**  **142** | **131.8**  **131.8**  **131.7** | **142.0**  **142.0**  **142.0** | **805**  **1858**  **1074** | **779**  **1565**  **993** | **8684**  **18311**  **11318** | **8074**  **15386**  **10185** | **3396**  **3375**  **3404** | **3518**  **3497**  **3527** |
| **1** | **1**  **2**  **3** | **6/30/05**  **6/30/05**  **6/30/05** | **UC006B1M5**  **UC006B1M9**  **UC006B1M15** | **D9S171**  **D9S171**  **D9S171** | **113**  **113**  **113** | **115**  **115**  **115** | **113.0**  **113.0**  **113.1** | **114.9**  **115.0**  **115.0** | **2364**  **1777**  **1604** | **1359**  **1171**  **1042** | **18830**  **13963**  **12395** | **9245**  **7814**  **7325** | **3157**  **3138**  **3166** | **3182**  **3163**  **3191** |
| **1** | **1** | **6/30/05** | **UC009B1M6** | **D17S250** | **191** | **195** | **191.3** | **195.4** | **1417** | **1048** | **11007** | **8105** | **4093** | **4143** |
|  | **2**  **3** | **6/30/05**  **6/30/05** | **UC009B1M8**  **UC009B1M11** | **D17S250**  **D17S250** | **191**  **191** | **195**  **195** | **191.5**  **191.3** | **195.5**  **195.4** | **2668**  **1601** | **1971**  **1116** | **21575**  **12753** | **15539**  **8435** | **4131**  **4056** | **4181**  **4106** |
| **1** | **1**  **2**  **3** | **6/30/05**  **6/30/05**  **6/30/05** | **UC009B1M6**  **UC009B1M8**  **UC009B1M11** | **D18S61**  **D18S61**  **D18S61** | **154**  **154**  **154** | **170**  **170**  **170** | **154.0**  **154.0**  **153.9** | **169.8**  **169.9**  **169.8** | **468**  **980**  **620** | **283**  **622**  **379** | **3746**  **7994**  **4855** | **2122**  **4975**  **2751** | **3640**  **3673**  **3606** | **3831**  **3866**  **3796** |
| **1** | **1**  **2**  **3** | **6/30/05**  **6/30/05**  **6/30/05** | **UC009B1M6**  **UC009B1M8**  **UC009B1M11** | **D2S123**  **D2S123**  **D2S123** | **219**  **219**  **219** |  | **218.3**  **218.4**  **218.3** |  | **1208**  **2415**  **1534** |  | **10008**  **20186**  **12778** |  | **4402**  **4442**  **4362** |  |
| **1** | **1**  **2**  **3** | **6/30/05**  **6/30/05**  **6/30/05** | **UC009B1M6**  **UC009B1M8**  **UC009B1M11** | **D3S1262**  **D3S1262**  **D3S1262** | **132**  **132**  **132** | **136**  **136**  **136** | **131.9**  **131.9**  **131.8** | **135.8**  **135.8**  **135.8** | **1440**  **2714**  **1568** | **1343**  **2716**  **1480** | **13189**  **26119**  **14870** | **12323**  **25643**  **13780** | **3383**  **3414**  **3351** | **3431**  **3463**  **3399** |
| **1** | **1**  **2**  **3** | **6/30/05**  **6/30/05**  **6/30/05** | **UC009B1M6**  **UC009B1M8**  **UC009B1M11** | **D9S171**  **D9S171**  **D9S171** | **115**  **115**  **115** | **121**  **121**  **121** | **115.0**  **115.1**  **115.0** | **120.9**  **120.9**  **120.8** | **747**  **1592**  **803** | **657**  **1342**  **754** | **5666**  **12065**  **6022** | **5105**  **10469**  **5586** | **3170**  **3200**  **3140** | **3245**  **3275**  **3214** |
| **1** | **1**  **1**  **1**  **1**  **1** | **6/20/05**  **6/20/05**  **6/20/05**  **6/20/05**  **6/20/05** | **UC018B1WT6**  **UC018B1WT6**  **UC018B1WT6**  **UC018B1WT6**  **UC018B1WT6** | **D17S250**  **D18S61**  **D2S123**  **D3S1262**  **D9S171** | **187**  **154**  **236**  **136**  **113** | **189**  **176**  **138** | **187.3**  **154.0**  **235.9**  **135.9**  **113.2** | **189.4**  **175.7**  **137.9** | **680**  **198**  **433**  **1041**  **942** | **477**  **153**  **544** | **5054**  **1581**  **3691**  **10256**  **7847** | **3451**  **1149**  **5047** | **4178**  **3764**  **4745**  **3550**  **3257** | **4204**  **4033**  **3575** |
| **1** | **1**  **2**  **3**  **4**  **5**  **6**  **7**  **8**  **9**  **10**  **11**  **12**  **13**  **14**  **15**  **16**  **17**  **18**  **19**  **20** | **7/5/05**  **7/5/05**  **7/5/05**  **7/5/05**  **7/5/05**  **7/5/05**  **7/5/05**  **7/5/05**  **7/5/05**  **7/5/05**  **7/5/05**  **7/5/05**  **7/5/05**  **7/5/05**  **7/5/05**  **7/5/05**  **7/5/05**  **7/5/05**  **7/5/05**  **7/5/05** | **CD3B1_2M9**  **CD3B1_2M27**  **CD3B1_2M35**  **CD3B1_2M2**  **CD3B1_2M10**  **CD3B1_2M20**  **CD3B1_2M28**  **CD3B1_2M36**  **CD3B1_2M3**  **CD3B1_2M11**  **CD3B1_2M21**  **CD3B1_2M37**  **CD3B1_2M12**  **CD3B1_2M22**  **CD3B1_2M30**  **CD3B1_2M38**  **CD3B1_2M5**  **CD3B1_2M13**  **CD3B1_2M23**  **CD3B1_2M31** | **D17S250**  **D17S250**  **D17S250**  **D17S250**  **D17S250**  **D17S250**  **D17S250**  **D17S250**  **D17S250**  **D17S250**  **D17S250**  **D17S250**  **D17S250**  **D17S250**  **D17S250**  **D17S250**  **D17S250**  **D17S250**  **D17S250**  **D17S250** | **181**  **181**  **181**  **181**  **181**  **181**  **181**  **181**  **181**  **181**  **181**  **181**  **181**  **181**  **181**  **181**  **181**  **181**  **181**  **181** | **202**  **202**  **202**  **202**  **202**  **202**  **202**  **202**  **202**  **202**  **202**  **202**  **202**  **202**  **202**  **202**  **202**  **202**  **202**  **202** | **181.0**  **181.0**  **181.1**  **181.0**  **181.0**  **181.0**  **181.0**  **181.1**  **181.0**  **180.9**  **181.1**  **181.0**  **181.0**  **181.0**  **181.1**  **181.1**  **181.0**  **181.0**  **181.1**  **181.1** | **201.6**  **201.7**  **201.8**  **201.7**  **201.7**  **201.7**  **201.7**  **201.7**  **201.7**  **201.7**  **201.7**  **201.7**  **201.8**  **201.7**  **201.9**  **201.8**  **201.8**  **201.7**  **201.8**  **201.7** | **1694**  **734**  **1502**  **1736**  **1251**  **790**  **1198**  **856**  **1920**  **1006**  **625**  **863**  **1641**  **1493**  **858**  **958**  **1815**  **969**  **1016**  **1022** | **726**  **334**  **703**  **763**  **594**  **380**  **631**  **421**  **908**  **463**  **315**  **436**  **830**  **771**  **395**  **380**  **896**  **434**  **536**  **554** | **14356**  **6403**  **12335**  **14353**  **10152**  **6452**  **9790**  **6978**  **16079**  **8178**  **5102**  **7177**  **13329**  **12519**  **6998**  **7893**  **15303**  **7893**  **8701**  **8576** | **5888**  **2861**  **5992**  **6296**  **4874**  **3316**  **4908**  **3355**  **7443**  **3966**  **2534**  **3487**  **6734**  **6381**  **3302**  **3141**  **7527**  **3591**  **4378**  **4505** | **3994**  **4017**  **4001**  **3989**  **3961**  **3982**  **3986**  **3975**  **4021**  **3988**  **4003**  **3993**  **4003**  **4015**  **4018**  **4006**  **4015**  **3996**  **4009**  **4002** | **4246**  **4271**  **4255**  **4242**  **4212**  **4235**  **4239**  **4226**  **4274**  **4241**  **4255**  **4244**  **4257**  **4269**  **4273**  **4260**  **4270**  **4249**  **4263**  **4255** |

| **Day** | **Count** | **Run Date** | **Sample Name** | **Marker** | **Allele 1** | **Allele 2** | **Size 1** | **Size 2** | **Height 1** | **Height 2** | **Peak Area 1** | **Peak Area 2** | **Data Point 1** | **Data Point 2** |
| --- | --- | --- | --- | --- | --- | --- | --- | --- | --- | --- | --- | --- | --- | --- |
|  | **21**  **22**  **23** | **7/5/05**  **7/5/05**  **7/5/05** | **CD3B1_2M39**  **CD3B1_2M6**  **CD3B1_2M14** | **D17S250**  **D17S250**  **D17S250** | **181**  **181**  **181** | **202**  **202**  **202** | **181.0**  **181.1**  **181.0** | **201.7**  **201.7**  **201.7** | **1187**  **1326**  **1301** | **518**  **653**  **646** | **9832**  **10746**  **10594** | **4348**  **5359**  **4953** | **3994**  **3985**  **3966** | **4247**  **4237**  **4218** |
|  | **24**  **25**  **26**  **27**  **28**  **29**  **30**  **31**  **32** | **7/5/05**  **7/5/05**  **7/5/05**  **7/5/05**  **7/5/05**  **7/5/05**  **7/5/05**  **7/5/05**  **7/5/05** | **CD3B1_2M32**  **CD3B1_2M7**  **CD3B1_2M16**  **CD3B1_2M33**  **CD3B1_2M8**  **CD3B1_2M17**  **CD3B1_2M26**  **CD3B1_2M34**  **CD3B1_2M42** | **D17S250**  **D17S250**  **D17S250**  **D17S250**  **D17S250**  **D17S250**  **D17S250**  **D17S250**  **D17S250** | **181**  **181**  **181**  **181**  **181**  **181**  **181**  **181**  **181** | **202**  **202**  **202**  **202**  **202**  **202**  **202**  **202**  **202** | **181.0**  **181.0**  **181.1**  **181.0**  **181.1**  **181.1**  **181.1**  **181.1**  **181.0** | **201.7**  **201.7**  **201.7**  **201.6**  **201.8**  **201.7**  **201.8**  **201.8**  **201.7** | **1114**  **1087**  **833**  **745**  **1599**  **1599**  **1281**  **1883**  **1469** | **509**  **455**  **455**  **367**  **807**  **801**  **472**  **839**  **499** | **9017**  **8967**  **6960**  **6118**  **12957**  **13177**  **10453**  **15605**  **11988** | **4052**  **3830**  **3716**  **3016**  **6598**  **6393**  **3744**  **6824**  **4111** | **3971**  **4012**  **3988**  **3989**  **4023**  **4000**  **4012**  **4003**  **3995** | **4223**  **4265**  **4239**  **4241**  **4277**  **4253**  **4266**  **4257**  **4248** |
| **1** | **1**  **2**  **3**  **4**  **5**  **6**  **7**  **8**  **9**  **10**  **11**  **12**  **13**  **14**  **15**  **16** | **7/5/05**  **7/5/05**  **7/5/05**  **7/5/05**  **7/5/05**  **7/5/05**  **7/5/05**  **7/5/05**  **7/5/05**  **7/5/05**  **7/5/05**  **7/5/05**  **7/5/05**  **7/5/05**  **7/5/05**  **7/5/05** | **CD3B1_2M9**  **CD3B1_2M27**  **CD3B1_2M35**  **CD3B1_2M2**  **CD3B1_2M10**  **CD3B1_2M20**  **CD3B1_2M28**  **CD3B1_2M36**  **CD3B1_2M3**  **CD3B1_2M11**  **CD3B1_2M21**  **CD3B1_2M37**  **CD3B1_2M12**  **CD3B1_2M22**  **CD3B1_2M30**  **CD3B1_2M38** | **D18S61**  **D18S61**  **D18S61**  **D18S61**  **D18S61**  **D18S61**  **D18S61**  **D18S61**  **D18S61**  **D18S61**  **D18S61**  **D18S61**  **D18S61**  **D18S61**  **D18S61**  **D18S61** | **164**  **164**  **164**  **164**  **164**  **164**  **164**  **164**  **164**  **164**  **164**  **164**  **164**  **164**  **164**  **164** | **172**  **172**  **172**  **172**  **172**  **172**  **172**  **172**  **172**  **172**  **172**  **172**  **172**  **172**  **172**  **172** | **163.8**  **163.9**  **163.9**  **163.9**  **163.8**  **163.9**  **163.8**  **163.8**  **163.9**  **163.8**  **163.9**  **163.8**  **163.9**  **163.9**  **163.9**  **163.9** | **171.6**  **171.7**  **171.8**  **171.6**  **171.7**  **171.7**  **171.6**  **171.7**  **171.7**  **171.6**  **171.8**  **171.6**  **171.6**  **171.7**  **171.8**  **171.7** | **484**  **231**  **378**  **536**  **427**  **286**  **416**  **422**  **546**  **347**  **170**  **406**  **521**  **534**  **414**  **455** | **418**  **136**  **286**  **433**  **343**  **204**  **296**  **450**  **398**  **274**  **205**  **308**  **346**  **458**  **233**  **392** | **3981**  **1828**  **3149**  **4693**  **3496**  **2326**  **3421**  **3400**  **4627**  **2759**  **1442**  **3181**  **4179**  **4237**  **3341**  **3608** | **3341**  **1063**  **2311**  **3364**  **2638**  **1520**  **2351**  **3489**  **3070**  **2162**  **1764**  **2408**  **2729**  **3489**  **2006**  **3146** | **3781**  **3804**  **3788**  **3778**  **3750**  **3770**  **3774**  **3762**  **3809**  **3777**  **3791**  **3781**  **3791**  **3802**  **3804**  **3793** | **3877**  **3901**  **3886**  **3874**  **3846**  **3866**  **3871**  **3859**  **3906**  **3873**  **3888**  **3877**  **3887**  **3899**  **3902**  **3890** |
|  | **17**  **18**  **19**  **20**  **21**  **22**  **23**  **24**  **25**  **26**  **27**  **28**  **29**  **30**  **31**  **32** | **7/5/05**  **7/5/05**  **7/5/05**  **7/5/05**  **7/5/05**  **7/5/05**  **7/5/05**  **7/5/05**  **7/5/05**  **7/5/05**  **7/5/05**  **7/5/05**  **7/5/05**  **7/5/05**  **7/5/05**  **7/5/05** | **CD3B1_2M5**  **CD3B1_2M13**  **CD3B1_2M23**  **CD3B1_2M31**  **CD3B1_2M39**  **CD3B1_2M6**  **CD3B1_2M14**  **CD3B1_2M32**  **CD3B1_2M7**  **CD3B1_2M16**  **CD3B1_2M33**  **CD3B1_2M8**  **CD3B1_2M17**  **CD3B1_2M26**  **CD3B1_2M34**  **CD3B1_2M42** | **D18S61**  **D18S61**  **D18S61**  **D18S61**  **D18S61**  **D18S61**  **D18S61**  **D18S61**  **D18S61**  **D18S61**  **D18S61**  **D18S61**  **D18S61**  **D18S61**  **D18S61**  **D18S61** | **164**  **164**  **164**  **164**  **164**  **164**  **164**  **164**  **164**  **164**  **164**  **164**  **164**  **164**  **164**  **164** | **172**  **172**  **172**  **172**  **172**  **172**  **172**  **172**  **172**  **172**  **172**  **172**  **172**  **172**  **172**  **172** | **163.9**  **163.9**  **163.9**  **163.8**  **163.9**  **163.9**  **163.8**  **163.9**  **163.9**  **163.8**  **163.9**  **163.9**  **164.0**  **163.9**  **163.9**  **163.8** | **171.7**  **171.6**  **171.7**  **171.7**  **171.6**  **171.7**  **171.6**  **171.7**  **171.6**  **171.6**  **171.6**  **171.8**  **171.7**  **171.7**  **171.7**  **171.7** | **503**  **552**  **361**  **266**  **439**  **468**  **344**  **312**  **223**  **207**  **384**  **514**  **549**  **314**  **558**  **309** | **372**  **409**  **254**  **206**  **341**  **297**  **262**  **202**  **176**  **283**  **258**  **371**  **479**  **178**  **418**  **230** | **4065**  **4482**  **2884**  **2282**  **3425**  **3827**  **2725**  **2464**  **2007**  **1689**  **3178**  **3969**  **4469**  **2604**  **4524**  **2636** | **2881**  **3336**  **1937**  **1587**  **2627**  **2334**  **2052**  **1551**  **1392**  **2121**  **1982**  **2907**  **3765**  **1424**  **3362**  **1835** | **3802**  **3784**  **3796**  **3788**  **3782**  **3773**  **3755**  **3760**  **3801**  **3776**  **3779**  **3810**  **3788**  **3799**  **3790**  **3782** | **3899**  **3880**  **3893**  **3886**  **3878**  **3869**  **3851**  **3856**  **3896**  **3872**  **3874**  **3907**  **3884**  **3896**  **3887**  **3880** |
| **1** | **1**  **2**  **3**  **4**  **5**  **6**  **7**  **8**  **9**  **10** | **7/5/05**  **7/5/05**  **7/5/05**  **7/5/05**  **7/5/05**  **7/5/05**  **7/5/05**  **7/5/05**  **7/5/05**  **7/5/05** | **CD3B1_2M9**  **CD3B1_2M27**  **CD3B1_2M35**  **CD3B1_2M2**  **CD3B1_2M10**  **CD3B1_2M20**  **CD3B1_2M28**  **CD3B1_2M36**  **CD3B1_2M3**  **CD3B1_2M11** | **D2S123**  **D2S123**  **D2S123**  **D2S123**  **D2S123**  **D2S123**  **D2S123**  **D2S123**  **D2S123**  **D2S123** | **223**  **223**  **223**  **223**  **223**  **223**  **223**  **223**  **223**  **223** | **236**  **236**  **236**  **236**  **236**  **236**  **236**  **236**  **236**  **236** | **222.8**  **222.8**  **222.7**  **222.7**  **222.7**  **222.8**  **222.7**  **222.8**  **222.7**  **222.7** | **235.8**  **236.0**  **235.9**  **235.9**  **235.8**  **235.8**  **235.9**  **235.9**  **235.8**  **235.8** | **723**  **302**  **555**  **882**  **670**  **386**  **727**  **705**  **658**  **462** | **715**  **265**  **518**  **846**  **589**  **324**  **634**  **572**  **635**  **484** | **6367**  **2696**  **4759**  **7948**  **5678**  **3173**  **6452**  **5779**  **5654**  **4219** | **6299**  **2161**  **4468**  **7288**  **4974**  **2610**  **5624**  **5013**  **5342**  **4221** | **4483**  **4508**  **4490**  **4477**  **4446**  **4471**  **4474**  **4462**  **4510**  **4475** | **4629**  **4656**  **4638**  **4624**  **4592**  **4617**  **4621**  **4608**  **4657**  **4621** |

| **Day** | **Count** | **Run Date** | **Sample Name** | **Marker** | **Allele 1** | **Allele 2** | **Size 1** | **Size 2** | **Height 1** | **Height 2** | **Peak Area 1** | **Peak Area 2** | **Data Point 1** | **Data Point 2** |
| --- | --- | --- | --- | --- | --- | --- | --- | --- | --- | --- | --- | --- | --- | --- |
|  | **11**  **12**  **13**  **14** | **7/5/05**  **7/5/05**  **7/5/05**  **7/5/05** | **CD3B1_2M21**  **CD3B1_2M37**  **CD3B1_2M12**  **CD3B1_2M22** | **D2S123**  **D2S123**  **D2S123**  **D2S123** | **223**  **223**  **223**  **223** | **236**  **236**  **236**  **236** | **222.7**  **222.8**  **222.8**  **222.7** | **235.8**  **235.8**  **235.8**  **235.8** | **278**  **708**  **608**  **782** | **410**  **724**  **557**  **695** | **2534**  **5916**  **5188**  **6698** | **3465**  **6026**  **4752**  **6006** | **4490**  **4479**  **4493**  **4506** | **4636**  **4624**  **4639**  **4653** |
|  | **15**  **16**  **17**  **18**  **19**  **20**  **21**  **22**  **23**  **24**  **25**  **26**  **27**  **28**  **29**  **30**  **31**  **32** | **7/5/05**  **7/5/05**  **7/5/05**  **7/5/05**  **7/5/05**  **7/5/05**  **7/5/05**  **7/5/05**  **7/5/05**  **7/5/05**  **7/5/05**  **7/5/05**  **7/5/05**  **7/5/05**  **7/5/05**  **7/5/05**  **7/5/05**  **7/5/05** | **CD3B1_2M30**  **CD3B1_2M38**  **CD3B1_2M5**  **CD3B1_2M13**  **CD3B1_2M23**  **CD3B1_2M31**  **CD3B1_2M39**  **CD3B1_2M6**  **CD3B1_2M14**  **CD3B1_2M32**  **CD3B1_2M7**  **CD3B1_2M16**  **CD3B1_2M33**  **CD3B1_2M8**  **CD3B1_2M17**  **CD3B1_2M26**  **CD3B1_2M34**  **CD3B1_2M42** | **D2S123**  **D2S123**  **D2S123**  **D2S123**  **D2S123**  **D2S123**  **D2S123**  **D2S123**  **D2S123**  **D2S123**  **D2S123**  **D2S123**  **D2S123**  **D2S123**  **D2S123**  **D2S123**  **D2S123**  **D2S123** | **223**  **223**  **223**  **223**  **223**  **223**  **223**  **223**  **223**  **223**  **223**  **223**  **223**  **223**  **223**  **223**  **223**  **223** | **236**  **236**  **236**  **236**  **236**  **236**  **236**  **236**  **236**  **236**  **236**  **236**  **236**  **236**  **236**  **236**  **236**  **236** | **222.9**  **222.8**  **222.8**  **222.7**  **222.8**  **222.8**  **222.8**  **222.8**  **222.7**  **222.7**  **222.7**  **222.7**  **222.8**  **222.8**  **222.8**  **222.8**  **222.8**  **222.8** | **236.0**  **235.9**  **235.8**  **235.8**  **236.0**  **235.9**  **235.9**  **235.9**  **235.7**  **235.8**  **235.8**  **235.7**  **235.8**  **235.9**  **235.9**  **236.0**  **235.9**  **235.8** | **586**  **891**  **622**  **962**  **595**  **395**  **518**  **585**  **471**  **458**  **478**  **667**  **618**  **571**  **752**  **333**  **655**  **463** | **520**  **909**  **566**  **901**  **403**  **387**  **473**  **551**  **476**  **408**  **459**  **486**  **530**  **545**  **699**  **413**  **572**  **385** | **5238**  **7860**  **5351**  **8785**  **5171**  **3559**  **4384**  **5185**  **4018**  **3710**  **3903**  **5673**  **5312**  **5043**  **6523**  **2661**  **5546**  **4098** | **4696**  **7784**  **5024**  **8302**  **3500**  **3344**  **4288**  **4954**  **3952**  **3496**  **4005**  **4125**  **4552**  **4727**  **5983**  **3536**  **4958**  **3443** | **4509**  **4496**  **4506**  **4484**  **4499**  **4492**  **4484**  **4472**  **4452**  **4457**  **4500**  **4473**  **4477**  **4513**  **4489**  **4502**  **4493**  **4484** | **4656**  **4643**  **4652**  **4631**  **4647**  **4638**  **4630**  **4618**  **4597**  **4603**  **4645**  **4618**  **4622**  **4660**  **4636**  **4650**  **4640**  **4630** |
| **1** | **1**  **2**  **3**  **4**  **5**  **6**  **7**  **8**  **9**  **10**  **11**  **12**  **13**  **14**  **15**  **16**  **17**  **18**  **19** | **7/5/05**  **7/5/05**  **7/5/05**  **7/5/05**  **7/5/05**  **7/5/05**  **7/5/05**  **7/5/05**  **7/5/05**  **7/5/05**  **7/5/05**  **7/5/05**  **7/5/05**  **7/5/05**  **7/5/05**  **7/5/05**  **7/5/05**  **7/5/05**  **7/5/05** | **CD3B1_2M9**  **CD3B1_2M27**  **CD3B1_2M35**  **CD3B1_2M2**  **CD3B1_2M10**  **CD3B1_2M20**  **CD3B1_2M28**  **CD3B1_2M36**  **CD3B1_2M3**  **CD3B1_2M11**  **CD3B1_2M21**  **CD3B1_2M37**  **CD3B1_2M12**  **CD3B1_2M22**  **CD3B1_2M30**  **CD3B1_2M38**  **CD3B1_2M5**  **CD3B1_2M13**  **CD3B1_2M23** | **D3S1262**  **D3S1262**  **D3S1262**  **D3S1262**  **D3S1262**  **D3S1262**  **D3S1262**  **D3S1262**  **D3S1262**  **D3S1262**  **D3S1262**  **D3S1262**  **D3S1262**  **D3S1262**  **D3S1262**  **D3S1262**  **D3S1262**  **D3S1262**  **D3S1262** | **136**  **136**  **136**  **136**  **136**  **136**  **136**  **136**  **136**  **136**  **136**  **136**  **136**  **136**  **136**  **136**  **136**  **136**  **136** |  | **135.7**  **135.7**  **135.7**  **135.6**  **135.7**  **135.7**  **135.7**  **135.7**  **135.7**  **135.6**  **135.7**  **135.7**  **135.7**  **135.7**  **135.7**  **135.7**  **135.7**  **135.7**  **135.7** |  | **2750**  **529**  **5026**  **2910**  **3435**  **718**  **2899**  **1260**  **4777**  **2260**  **948**  **1482**  **4916**  **3546**  **951**  **1402**  **4621**  **2363**  **734** |  | **26459**  **4720**  **46811**  **30258**  **32565**  **6654**  **27304**  **11663**  **48842**  **20847**  **9056**  **13849**  **47561**  **34431**  **9076**  **13789**  **46309**  **22732**  **6972** |  | **3450**  **3471**  **3456**  **3446**  **3421**  **3439**  **3443**  **3432**  **3477**  **3447**  **3460**  **3451**  **3459**  **3469**  **3472**  **3461**  **3470**  **3452**  **3464** |  |
|  | **20**  **21**  **22**  **23**  **24**  **25**  **26**  **27**  **28**  **29**  **30**  **31**  **32** | **7/5/05**  **7/5/05**  **7/5/05**  **7/5/05**  **7/5/05**  **7/5/05**  **7/5/05**  **7/5/05**  **7/5/05**  **7/5/05**  **7/5/05**  **7/5/05**  **7/5/05** | **CD3B1_2M31**  **CD3B1_2M39**  **CD3B1_2M6**  **CD3B1_2M14**  **CD3B1_2M32**  **CD3B1_2M7**  **CD3B1_2M16**  **CD3B1_2M33**  **CD3B1_2M8**  **CD3B1_2M17**  **CD3B1_2M26**  **CD3B1_2M34**  **CD3B1_2M42** | **D3S1262**  **D3S1262**  **D3S1262**  **D3S1262**  **D3S1262**  **D3S1262**  **D3S1262**  **D3S1262**  **D3S1262**  **D3S1262**  **D3S1262**  **D3S1262**  **D3S1262** | **136**  **136**  **136**  **136**  **136**  **136**  **136**  **136**  **136**  **136**  **136**  **136**  **136** |  | **135.7**  **135.7**  **135.7**  **135.7**  **135.7**  **135.7**  **135.7**  **135.7**  **135.7**  **135.7**  **135.7**  **135.7**  **135.7** |  | **2507**  **2577**  **2992**  **3645**  **2794**  **1044**  **671**  **1686**  **2467**  **2694**  **924**  **4076**  **565** |  | **23597**  **24553**  **28976**  **34080**  **25848**  **10239**  **5944**  **15801**  **23790**  **25583**  **8542**  **38758**  **5336** |  | **3457**  **3450**  **3442**  **3426**  **3429**  **3470**  **3446**  **3448**  **3477**  **3456**  **3466**  **3458**  **3451** |  |

| **Day** | **Count** | **Run Date** | **Sample Name** | **Marker** | **Allele 1** | **Allele 2** | **Size 1** | **Size 2** | **Height 1** | **Height 2** | **Peak Area 1** | **Peak Area 2** | **Data Point 1** | **Data Point 2** |
| --- | --- | --- | --- | --- | --- | --- | --- | --- | --- | --- | --- | --- | --- | --- |
| **1** | **1**  **2**  **3**  **4**  **5** | **7/5/05**  **7/5/05**  **7/5/05**  **7/5/05**  **7/5/05** | **CD3B1_2M9**  **CD3B1_2M27**  **CD3B1_2M35**  **CD3B1_2M2**  **CD3B1_2M10** | **D9S171**  **D9S171**  **D9S171**  **D9S171**  **D9S171** | **113**  **113**  **113**  **113**  **113** |  | **112.9**  **113.0**  **113.0**  **112.9**  **113.0** |  | **2115**  **701**  **2445**  **2020**  **2100** |  | **17422**  **5689**  **19793**  **16127**  **16834** |  | **3160**  **3180**  **3166**  **3157**  **3133** |  |
|  | **6**  **7**  **8**  **9**  **10**  **11**  **12**  **13**  **14**  **15**  **16**  **17**  **18**  **19**  **20**  **21**  **22**  **23**  **24**  **25**  **26**  **27**  **28**  **29**  **30**  **31**  **32** | **7/5/05**  **7/5/05**  **7/5/05**  **7/5/05**  **7/5/05**  **7/5/05**  **7/5/05**  **7/5/05**  **7/5/05**  **7/5/05**  **7/5/05**  **7/5/05**  **7/5/05**  **7/5/05**  **7/5/05**  **7/5/05**  **7/5/05**  **7/5/05**  **7/5/05**  **7/5/05**  **7/5/05**  **7/5/05**  **7/5/05**  **7/5/05**  **7/5/05**  **7/5/05**  **7/5/05** | **CD3B1_2M20**  **CD3B1_2M28**  **CD3B1_2M36**  **CD3B1_2M3**  **CD3B1_2M11**  **CD3B1_2M21**  **CD3B1_2M37**  **CD3B1_2M12**  **CD3B1_2M22**  **CD3B1_2M30**  **CD3B1_2M38**  **CD3B1_2M5**  **CD3B1_2M13**  **CD3B1_2M23**  **CD3B1_2M31**  **CD3B1_2M39**  **CD3B1_2M6**  **CD3B1_2M14**  **CD3B1_2M32**  **CD3B1_2M7**  **CD3B1_2M16**  **CD3B1_2M33**  **CD3B1_2M8**  **CD3B1_2M17**  **CD3B1_2M26**  **CD3B1_2M34**  **CD3B1_2M42** | **D9S171**  **D9S171**  **D9S171**  **D9S171**  **D9S171**  **D9S171**  **D9S171**  **D9S171**  **D9S171**  **D9S171**  **D9S171**  **D9S171**  **D9S171**  **D9S171**  **D9S171**  **D9S171**  **D9S171**  **D9S171**  **D9S171**  **D9S171**  **D9S171**  **D9S171**  **D9S171**  **D9S171**  **D9S171**  **D9S171**  **D9S171** | **113**  **113**  **113**  **113**  **113**  **113**  **113**  **113**  **113**  **113**  **113**  **113**  **113**  **113**  **113**  **113**  **113**  **113**  **113**  **113**  **113**  **113**  **113**  **113**  **113**  **113**  **113** |  | **112.9**  **112.9**  **113.0**  **112.9**  **112.9**  **112.9**  **112.9**  **112.9**  **113.0**  **112.9**  **112.9**  **112.9**  **112.9**  **112.9**  **112.9**  **113.0**  **112.9**  **113.0**  **112.9**  **112.9**  **112.9**  **112.9**  **112.9**  **113.0**  **113.0**  **112.9**  **112.9** |  | **751**  **1899**  **1166**  **2222**  **1507**  **653**  **1237**  **2498**  **2242**  **1116**  **1310**  **2320**  **3118**  **853**  **1424**  **1619**  **1907**  **1719**  **1454**  **1000**  **839**  **1453**  **1475**  **2059**  **699**  **2150**  **722** |  | **6226**  **15453**  **9264**  **18037**  **12451**  **5248**  **9828**  **20224**  **18199**  **8672**  **10817**  **19022**  **25528**  **7024**  **11925**  **13313**  **15415**  **13675**  **11420**  **8270**  **7082**  **11971**  **11922**  **17248**  **5733**  **17420**  **5730** |  | **3150**  **3154**  **3144**  **3186**  **3158**  **3170**  **3162**  **3168**  **3178**  **3181**  **3170**  **3179**  **3162**  **3173**  **3166**  **3161**  **3153**  **3138**  **3141**  **3180**  **3157**  **3159**  **3186**  **3166**  **3176**  **3168**  **3161** |  |
| **1** | **1**  **2**  **3**  **4**  **5**  **6**  **7**  **8**  **9**  **10**  **11**  **12**  **13**  **14**  **15**  **16**  **17**  **18**  **19**  **20**  **21** | **7/6/05**  **7/6/05**  **7/6/05**  **7/6/05**  **7/6/05**  **7/6/05**  **7/6/05**  **7/6/05**  **7/6/05**  **7/6/05**  **7/6/05**  **7/6/05**  **7/6/05**  **7/6/05**  **7/6/05**  **7/6/05**  **7/6/05**  **7/6/05**  **7/6/05**  **7/6/05**  **7/6/05** | **CD006B1_M2**  **CD006B1_M15**  **CD006B1_M27**  **CD006B1_M37**  **CD006B1_M48**  **CD006B1_M3**  **CD006B1_M28**  **CD006B1_M39**  **CD006B1_M51**  **CD006B1_M4**  **CD006B1_M29**  **CD006B1_M40**  **CD006B1_M52**  **CD006B1_M5**  **CD006B1_M20**  **CD006B1_M31**  **CD006B1_M41**  **CD006B1_M53**  **CD006B1_M73**  **CD006B1_M6**  **CD006B1_M23** | **D17S250**  **D17S250**  **D17S250**  **D17S250**  **D17S250**  **D17S250**  **D17S250**  **D17S250**  **D17S250**  **D17S250**  **D17S250**  **D17S250**  **D17S250**  **D17S250**  **D17S250**  **D17S250**  **D17S250**  **D17S250**  **D17S250**  **D17S250**  **D17S250** | **191**  **191**  **191**  **191**  **191**  **191**  **191**  **191**  **191**  **191**  **191**  **191**  **191**  **191**  **191**  **191**  **191**  **191**  **191**  **191**  **191** |  | **191.3**  **191.3**  **191.4**  **191.4**  **191.3**  **191.4**  **191.4**  **191.3**  **191.4**  **191.4**  **191.4**  **191.3**  **191.3**  **191.4**  **191.4**  **191.5**  **191.4**  **191.4**  **191.4**  **191.4**  **191.4** |  | **3693**  **3922**  **2317**  **1731**  **1391**  **3606**  **1309**  **1777**  **1122**  **3585**  **1314**  **1390**  **1021**  **3985**  **3510**  **1674**  **2108**  **1722**  **2239**  **3000**  **2460** |  | **32218**  **32949**  **19965**  **14769**  **11433**  **31358**  **11037**  **14874**  **8897**  **31324**  **11218**  **12048**  **8714**  **34972**  **30220**  **14383**  **17706**  **14686**  **18975**  **25759**  **20942** |  | **4208**  **4146**  **4230**  **4174**  **4174**  **4179**  **4208**  **4149**  **4149**  **4189**  **4216**  **4158**  **4129**  **4203**  **4152**  **4231**  **4175**  **4187**  **4197**  **4169**  **4157** |  |

| **Day** | **Count** | **Run Date** | **Sample Name** | **Marker** | **Allele 1** | **Allele 2** | **Size 1** | **Size 2** | **Height 1** | **Height 2** | **Peak Area 1** | **Peak Area 2** | **Data Point 1** | **Data Point 2** |
| --- | --- | --- | --- | --- | --- | --- | --- | --- | --- | --- | --- | --- | --- | --- |
|  | **22** | **7/6/05** | **CD006B1_M32** | **D17S250** | **191** |  | **191.3** |  | **1622** |  | **13736** |  | **4183** |  |
|  | **23**  **24**  **25**  **26**  **27**  **28** | **7/6/05**  **7/6/05**  **7/6/05**  **7/6/05**  **7/6/05**  **7/6/05** | **CD006B1_M43**  **CD006B1_M54**  **CD006B1_M75**  **CD006B1_M8**  **CD006B1_M24**  **CD006B1_M33** | **D17S250**  **D17S250**  **D17S250**  **D17S250**  **D17S250**  **D17S250** | **191**  **191**  **191**  **191**  **191**  **191** |  | **191.3**  **191.4**  **191.3**  **191.3**  **191.3**  **191.4** |  | **1648**  **1233**  **1529**  **3101**  **2207**  **1055** |  | **13768**  **10287**  **13034**  **26287**  **18521**  **9031** |  | **4161**  **4180**  **4208**  **4145**  **4132**  **4162** |  |
|  | **29**  **30**  **31**  **32**  **33**  **34**  **35**  **36**  **37**  **38**  **39**  **40**  **41** | **7/6/05**  **7/6/05**  **7/6/05**  **7/6/05**  **7/6/05**  **7/6/05**  **7/6/05**  **7/6/05**  **7/6/05**  **7/6/05**  **7/6/05**  **7/6/05**  **7/6/05** | **CD006B1_M45**  **CD006B1_M55**  **CD006B1_M82**  **CD006B1_M9**  **CD006B1_M25**  **CD006B1_M35**  **CD006B1_M46**  **CD006B1_M65**  **CD006B1_M14**  **CD006B1_M26**  **CD006B1_M36**  **CD006B1_M47**  **CD006B1_M67** | **D17S250**  **D17S250**  **D17S250**  **D17S250**  **D17S250**  **D17S250**  **D17S250**  **D17S250**  **D17S250**  **D17S250**  **D17S250**  **D17S250**  **D17S250** | **191**  **191**  **191**  **191**  **191**  **191**  **191**  **191**  **191**  **191**  **191**  **191**  **191** |  | **191.4**  **191.3**  **191.3**  **191.3**  **191.4**  **191.4**  **191.3**  **191.4**  **191.3**  **191.4**  **191.4**  **191.4**  **191.4** |  | **1671**  **1464**  **1316**  **2700**  **1672**  **2249**  **2159**  **1311**  **3517**  **2983**  **1724**  **2013**  **1591** |  | **14135**  **12191**  **11191**  **22975**  **13943**  **18744**  **18093**  **11061**  **30219**  **25238**  **14839**  **16828**  **13784** |  | **4138**  **4156**  **4184**  **4155**  **4141**  **4167**  **4118**  **4139**  **4167**  **4159**  **4186**  **4164**  **4181** |  |
| **1** | **1**  **2**  **3**  **4**  **5**  **6**  **7**  **8**  **9**  **10**  **11**  **12**  **13**  **14**  **15**  **16**  **17**  **18**  **19**  **20**  **21**  **22**  **23**  **24**  **25**  **26**  **27**  **28**  **29**  **30**  **31**  **32**  **33**  **34** | **7/6/05**  **7/6/05**  **7/6/05**  **7/6/05**  **7/6/05**  **7/6/05**  **7/6/05**  **7/6/05**  **7/6/05**  **7/6/05**  **7/6/05**  **7/6/05**  **7/6/05**  **7/6/05**  **7/6/05**  **7/6/05**  **7/6/05**  **7/6/05**  **7/6/05**  **7/6/05**  **7/6/05**  **7/6/05**  **7/6/05**  **7/6/05**  **7/6/05**  **7/6/05**  **7/6/05**  **7/6/05**  **7/6/05**  **7/6/05**  **7/6/05**  **7/6/05**  **7/6/05**  **7/6/05** | **CD006B1_M2**  **CD006B1_M15**  **CD006B1_M27**  **CD006B1_M37**  **CD006B1_M48**  **CD006B1_M3**  **CD006B1_M28**  **CD006B1_M39**  **CD006B1_M51**  **CD006B1_M4**  **CD006B1_M29**  **CD006B1_M40**  **CD006B1_M52**  **CD006B1_M5**  **CD006B1_M20**  **CD006B1_M31**  **CD006B1_M41**  **CD006B1_M53**  **CD006B1_M73**  **CD006B1_M6**  **CD006B1_M23**  **CD006B1_M32**  **CD006B1_M43**  **CD006B1_M54**  **CD006B1_M75**  **CD006B1_M8**  **CD006B1_M24**  **CD006B1_M33**  **CD006B1_M45**  **CD006B1_M55**  **CD006B1_M82**  **CD006B1_M9**  **CD006B1_M25**  **CD006B1_M35** | **D18S61**  **D18S61**  **D18S61**  **D18S61**  **D18S61**  **D18S61**  **D18S61**  **D18S61**  **D18S61**  **D18S61**  **D18S61**  **D18S61**  **D18S61**  **D18S61**  **D18S61**  **D18S61**  **D18S61**  **D18S61**  **D18S61**  **D18S61**  **D18S61**  **D18S61**  **D18S61**  **D18S61**  **D18S61**  **D18S61**  **D18S61**  **D18S61**  **D18S61**  **D18S61**  **D18S61**  **D18S61**  **D18S61**  **D18S61** | **168**  **168**  **168**  **168**  **168**  **168**  **168**  **168**  **168**  **168**  **168**  **168**  **168**  **168**  **168**  **168**  **168**  **168**  **168**  **168**  **168**  **168**  **168**  **168**  **168**  **168**  **168**  **168**  **168**  **168**  **168**  **168**  **168**  **168** | **170**  **170**  **170**  **170**  **170**  **170**  **170**  **170**  **170**  **170**  **170**  **170**  **170**  **170**  **170**  **170**  **170**  **170**  **170**  **170**  **170**  **170**  **170**  **170**  **170**  **170**  **170**  **170**  **170**  **170**  **170**  **170**  **170**  **170** | **167.9**  **167.8**  **167.9**  **167.9**  **167.8**  **167.8**  **167.8**  **167.8**  **167.8**  **167.8**  **167.8**  **167.7**  **167.8**  **167.8**  **167.8**  **167.8**  **167.9**  **167.9**  **167.8**  **167.8**  **167.8**  **167.7**  **167.7**  **167.8**  **167.8**  **167.7**  **167.8**  **167.8**  **167.7**  **167.8**  **167.7**  **167.7**  **167.8**  **167.8** | **169.8**  **169.8**  **169.8**  **169.8**  **169.7**  **169.8**  **169.7**  **169.6**  **169.7**  **169.8**  **169.7**  **169.7**  **169.7**  **169.8**  **169.8**  **169.8**  **169.8**  **169.8**  **169.8**  **169.8**  **169.8**  **169.7**  **169.7**  **169.7**  **169.8**  **169.6**  **169.7**  **169.7**  **169.7**  **169.8**  **169.7**  **169.8**  **169.7**  **169.7** | **1823**  **1788**  **1187**  **1162**  **807**  **1544**  **703**  **812**  **747**  **1625**  **569**  **750**  **527**  **1812**  **1729**  **1100**  **1176**  **952**  **1355**  **1661**  **1242**  **1050**  **854**  **853**  **704**  **1486**  **1033**  **513**  **759**  **875**  **897**  **982**  **682**  **840** | **1176**  **1157**  **691**  **779**  **486**  **954**  **415**  **578**  **343**  **942**  **381**  **501**  **290**  **1157**  **1105**  **681**  **788**  **569**  **793**  **1117**  **831**  **658**  **561**  **529**  **435**  **886**  **704**  **358**  **462**  **550**  **621**  **657**  **458**  **545** | **14688**  **14283**  **9253**  **9087**  **6345**  **12038**  **5619**  **6480**  **5780**  **13027**  **4619**  **5921**  **4176**  **14567**  **13657**  **8623**  **9325**  **7501**  **10670**  **13268**  **9781**  **8132**  **6604**  **6775**  **5498**  **11873**  **8078**  **3954**  **5906**  **6785**  **7062**  **7728**  **5307**  **6372** | **8587**  **8764**  **5010**  **5796**  **3545**  **7190**  **3036**  **4123**  **2491**  **6921**  **2881**  **3735**  **2113**  **8540**  **7927**  **5179**  **5803**  **4212**  **6013**  **8302**  **6023**  **4999**  **4100**  **3794**  **3168**  **6431**  **5005**  **2491**  **3467**  **4081**  **4668**  **4653**  **3222**  **3835** | **3912**  **3853**  **3933**  **3880**  **3880**  **3884**  **3911**  **3857**  **3856**  **3895**  **3921**  **3866**  **3840**  **3907**  **3858**  **3933**  **3881**  **3892**  **3901**  **3874**  **3863**  **3888**  **3867**  **3885**  **3912**  **3852**  **3840**  **3868**  **3845**  **3862**  **3888**  **3863**  **3849**  **3875** | **3936**  **3878**  **3957**  **3904**  **3904**  **3909**  **3935**  **3880**  **3880**  **3920**  **3945**  **3890**  **3863**  **3932**  **3883**  **3958**  **3905**  **3916**  **3926**  **3899**  **3888**  **3913**  **3892**  **3909**  **3937**  **3876**  **3864**  **3892**  **3869**  **3887**  **3913**  **3888**  **3873**  **3899** |

| **Day** | **Count** | **Run Date** | **Sample Name** | **Marker** | **Allele 1** | **Allele 2** | **Size 1** | **Size 2** | **Height 1** | **Height 2** | **Peak Area 1** | **Peak Area 2** | **Data Point 1** | **Data Point 2** |
| --- | --- | --- | --- | --- | --- | --- | --- | --- | --- | --- | --- | --- | --- | --- |
|  | **35**  **36**  **37**  **38**  **39**  **40**  **41** | **7/6/05**  **7/6/05**  **7/6/05**  **7/6/05**  **7/6/05**  **7/6/05**  **7/6/05** | **CD006B1_M46**  **CD006B1_M65**  **CD006B1_M14**  **CD006B1_M26**  **CD006B1_M36**  **CD006B1_M47**  **CD006B1_M67** | **D18S61**  **D18S61**  **D18S61**  **D18S61**  **D18S61**  **D18S61**  **D18S61** | **168**  **168**  **168**  **168**  **168**  **168**  **168** | **170**  **170**  **170**  **170**  **170**  **170**  **170** | **167.7**  **167.9**  **167.9**  **167.8**  **167.8**  **167.8**  **167.8** | **169.6**  **169.7**  **169.8**  **169.8**  **169.7**  **169.8**  **169.7** | **862**  **654**  **1774**  **1567**  **914**  **928**  **1278** | **582**  **409**  **1137**  **1037**  **531**  **587**  **753** | **6495**  **5217**  **14190**  **12414**  **7198**  **7106**  **10428** | **4135**  **2888**  **8486**  **7495**  **3982**  **4286**  **5636** | **3829**  **3849**  **3874**  **3864**  **3891**  **3870**  **3886** | **3852**  **3872**  **3898**  **3889**  **3915**  **3895**  **3910** |
| **1** | **1** | **7/6/05** | **CD006B1_M2** | **D2S123** | **219** |  | **218.5** |  | **2307** |  | **22294** |  | **4527** |  |
|  | **2**  **3**  **4**  **5**  **6**  **7** | **7/6/05**  **7/6/05**  **7/6/05**  **7/6/05**  **7/6/05**  **7/6/05** | **CD006B1_M15**  **CD006B1_M27**  **CD006B1_M37**  **CD006B1_M48**  **CD006B1_M3**  **CD006B1_M28** | **D2S123**  **D2S123**  **D2S123**  **D2S123**  **D2S123**  **D2S123** | **219**  **219**  **219**  **219**  **219**  **219** |  | **218.4**  **218.6**  **218.5**  **218.4**  **218.5**  **218.5** |  | **2857**  **1804**  **1083**  **1218**  **2579**  **1115** |  | **27006**  **16067**  **9826**  **10682**  **24887**  **10419** |  | **4461**  **4550**  **4490**  **4490**  **4497**  **4527** |  |
|  | **8**  **9**  **10**  **11**  **12**  **13**  **14**  **15**  **16**  **17**  **18**  **19**  **20**  **21**  **22**  **23**  **24**  **25**  **26**  **27**  **28**  **29**  **30**  **31**  **32**  **33**  **34**  **35**  **36**  **37**  **38**  **39**  **40**  **41** | **7/6/05**  **7/6/05**  **7/6/05**  **7/6/05**  **7/6/05**  **7/6/05**  **7/6/05**  **7/6/05**  **7/6/05**  **7/6/05**  **7/6/05**  **7/6/05**  **7/6/05**  **7/6/05**  **7/6/05**  **7/6/05**  **7/6/05**  **7/6/05**  **7/6/05**  **7/6/05**  **7/6/05**  **7/6/05**  **7/6/05**  **7/6/05**  **7/6/05**  **7/6/05**  **7/6/05**  **7/6/05**  **7/6/05**  **7/6/05**  **7/6/05**  **7/6/05**  **7/6/05**  **7/6/05** | **CD006B1_M39**  **CD006B1_M51**  **CD006B1_M4**  **CD006B1_M29**  **CD006B1_M40**  **CD006B1_M52**  **CD006B1_M5**  **CD006B1_M20**  **CD006B1_M31**  **CD006B1_M41**  **CD006B1_M53**  **CD006B1_M73**  **CD006B1_M6**  **CD006B1_M23**  **CD006B1_M32**  **CD006B1_M43**  **CD006B1_M54**  **CD006B1_M75**  **CD006B1_M8**  **CD006B1_M24**  **CD006B1_M33**  **CD006B1_M45**  **CD006B1_M55**  **CD006B1_M82**  **CD006B1_M9**  **CD006B1_M25**  **CD006B1_M35**  **CD006B1_M46**  **CD006B1_M65**  **CD006B1_M14**  **CD006B1_M26**  **CD006B1_M36**  **CD006B1_M47**  **CD006B1_M67** | **D2S123**  **D2S123**  **D2S123**  **D2S123**  **D2S123**  **D2S123**  **D2S123**  **D2S123**  **D2S123**  **D2S123**  **D2S123**  **D2S123**  **D2S123**  **D2S123**  **D2S123**  **D2S123**  **D2S123**  **D2S123**  **D2S123**  **D2S123**  **D2S123**  **D2S123**  **D2S123**  **D2S123**  **D2S123**  **D2S123**  **D2S123**  **D2S123**  **D2S123**  **D2S123**  **D2S123**  **D2S123**  **D2S123**  **D2S123** | **219**  **219**  **219**  **219**  **219**  **219**  **219**  **219**  **219**  **219**  **219**  **219**  **219**  **219**  **219**  **219**  **219**  **219**  **219**  **219**  **219**  **219**  **219**  **219**  **219**  **219**  **219**  **219**  **219**  **219**  **219**  **219**  **219**  **219** |  | **218.5**  **218.4**  **218.5**  **218.6**  **218.5**  **218.4**  **218.6**  **218.5**  **218.7**  **218.5**  **218.5**  **218.5**  **218.4**  **218.5**  **218.5**  **218.4**  **218.5**  **218.5**  **218.4**  **218.4**  **218.4**  **218.4**  **218.5**  **218.5**  **218.4**  **218.5**  **218.5**  **218.5**  **218.5**  **218.4**  **218.5**  **218.6**  **218.5**  **218.5** |  | **1504**  **1181**  **2668**  **852**  **1088**  **718**  **3419**  **2573**  **1475**  **1948**  **1684**  **2261**  **2427**  **2083**  **1403**  **1467**  **1291**  **1018**  **2368**  **1775**  **991**  **1538**  **1451**  **1544**  **1976**  **1238**  **1555**  **1696**  **1235**  **1947**  **2361**  **1210**  **1423**  **2049** |  | **13712**  **10218**  **25699**  **7688**  **9900**  **6115**  **33921**  **24488**  **14053**  **18614**  **15214**  **20358**  **23133**  **19796**  **13218**  **13098**  **11456**  **9438**  **22900**  **16301**  **8689**  **13529**  **12417**  **14375**  **19025**  **11460**  **13814**  **14661**  **10919**  **18189**  **21908**  **11099**  **12836**  **18504** |  | **4465**  **4464**  **4505**  **4533**  **4472**  **4439**  **4522**  **4467**  **4551**  **4491**  **4505**  **4515**  **4484**  **4473**  **4500**  **4477**  **4497**  **4527**  **4460**  **4447**  **4477**  **4453**  **4473**  **4503**  **4469**  **4455**  **4482**  **4429**  **4451**  **4483**  **4475**  **4503**  **4480**  **4498** |  |
| **1** | **1**  **2**  **3**  **4**  **5**  **6** | **7/6/05**  **7/6/05**  **7/6/05**  **7/6/05**  **7/6/05**  **7/6/05** | **CD006B1_M2**  **CD006B1_M15**  **CD006B1_M27**  **CD006B1_M37**  **CD006B1_M48**  **CD006B1_M3** | **D3S1262**  **D3S1262**  **D3S1262**  **D3S1262**  **D3S1262**  **D3S1262** | **132**  **132**  **132**  **132**  **132**  **132** | **146**  **146**  **146**  **146**  **146**  **146** | **131.7**  **131.7**  **131.7**  **131.7**  **131.6**  **131.7** | **146.1**  **146.1**  **146.3**  **146.2**  **146.2**  **146.2** | **1920**  **1918**  **1526**  **942**  **975**  **1754** | **1451**  **1521**  **1174**  **771**  **606**  **1429** | **25366**  **24002**  **17318**  **10224**  **9704**  **23418** | **16812**  **16970**  **11742**  **7390**  **5880**  **17192** | **3474**  **3421**  **3494**  **3446**  **3446**  **3448** | **3649**  **3594**  **3670**  **3620**  **3620**  **3623** |

| **Day** | **Count** | **Run Date** | **Sample Name** | **Marker** | **Allele 1** | **Allele 2** | **Size 1** | **Size 2** | **Height 1** | **Height 2** | **Peak Area 1** | **Peak Area 2** | **Data Point 1** | **Data Point 2** |
| --- | --- | --- | --- | --- | --- | --- | --- | --- | --- | --- | --- | --- | --- | --- |
|  | **7**  **8**  **9**  **10**  **11**  **12**  **13**  **14**  **15** | **7/6/05**  **7/6/05**  **7/6/05**  **7/6/05**  **7/6/05**  **7/6/05**  **7/6/05**  **7/6/05**  **7/6/05** | **CD006B1_M28**  **CD006B1_M39**  **CD006B1_M51**  **CD006B1_M4**  **CD006B1_M29**  **CD006B1_M40**  **CD006B1_M52**  **CD006B1_M5**  **CD006B1_M20** | **D3S1262**  **D3S1262**  **D3S1262**  **D3S1262**  **D3S1262**  **D3S1262**  **D3S1262**  **D3S1262**  **D3S1262** | **132**  **132**  **132**  **132**  **132**  **132**  **132**  **132**  **132** | **146**  **146**  **136**  **146**  **146**  **146**  **146**  **146**  **146** | **131.8**  **131.6**  **131.6**  **131.6**  **131.7**  **131.8**  **131.7**  **131.8**  **131.7** | **146.2**  **146.1**  **135.6**  **146.2**  **146.2**  **146.2**  **146.1**  **146.2**  **146.1** | **863**  **1100**  **1163**  **2019**  **672**  **908**  **556**  **2497**  **2007** | **606**  **974**  **1068**  **1651**  **585**  **710**  **364**  **2020**  **1631** | **9528**  **11720**  **11441**  **26247**  **7598**  **9864**  **5680**  **32681**  **24573** | **6039**  **9363**  **10226**  **19218**  **5896**  **6866**  **3570**  **24354**  **17939** | **3474**  **3424**  **3423**  **3461**  **3485**  **3436**  **3412**  **3471**  **3426** | **3648**  **3597**  **3473**  **3635**  **3660**  **3608**  **3583**  **3645**  **3599** |
|  | **16**  **17**  **18**  **19**  **20**  **21**  **22**  **23**  **24**  **25**  **26**  **27**  **28**  **29**  **30**  **31**  **32**  **33** | **7/6/05**  **7/6/05**  **7/6/05**  **7/6/05**  **7/6/05**  **7/6/05**  **7/6/05**  **7/6/05**  **7/6/05**  **7/6/05**  **7/6/05**  **7/6/05**  **7/6/05**  **7/6/05**  **7/6/05**  **7/6/05**  **7/6/05**  **7/6/05** | **CD006B1_M31**  **CD006B1_M41**  **CD006B1_M53**  **CD006B1_M73**  **CD006B1_M6**  **CD006B1_M23**  **CD006B1_M32**  **CD006B1_M43**  **CD006B1_M54**  **CD006B1_M75**  **CD006B1_M8**  **CD006B1_M24**  **CD006B1_M33**  **CD006B1_M45**  **CD006B1_M55**  **CD006B1_M82**  **CD006B1_M9**  **CD006B1_M25** | **D3S1262**  **D3S1262**  **D3S1262**  **D3S1262**  **D3S1262**  **D3S1262**  **D3S1262**  **D3S1262**  **D3S1262**  **D3S1262**  **D3S1262**  **D3S1262**  **D3S1262**  **D3S1262**  **D3S1262**  **D3S1262**  **D3S1262**  **D3S1262** | **132**  **132**  **132**  **132**  **132**  **132**  **132**  **132**  **132**  **132**  **132**  **132**  **132**  **132**  **132**  **132**  **132**  **132** | **146**  **146**  **146**  **146**  **146**  **146**  **146**  **146**  **146**  **146**  **146**  **146**  **146**  **146**  **146**  **146**  **146**  **146** | **131.7**  **131.7**  **131.8**  **131.8**  **131.6**  **131.7**  **131.7**  **131.7**  **131.6**  **131.7**  **131.6**  **131.7**  **131.7**  **131.7**  **131.6**  **131.7**  **131.7**  **131.7** | **146.2**  **146.2**  **146.2**  **146.2**  **146.1**  **146.2**  **146.2**  **146.2**  **146.2**  **146.2**  **146.2**  **146.2**  **146.2**  **146.2**  **146.2**  **146.2**  **146.1**  **146.1** | **1168**  **1666**  **1324**  **1234**  **1448**  **1598**  **1141**  **1219**  **610**  **787**  **1787**  **1248**  **843**  **1265**  **905**  **681**  **1470**  **1021** | **873**  **1302**  **896**  **976**  **1092**  **1227**  **840**  **943**  **492**  **609**  **1568**  **1004**  **647**  **908**  **737**  **472**  **1254**  **791** | **12329**  **17942**  **13903**  **13602**  **18559**  **18520**  **12440**  **13142**  **6443**  **8138**  **22694**  **14076**  **8781**  **12921**  **9601**  **7204**  **18884**  **11488** | **8592**  **13087**  **8744**  **9635**  **12375**  **12895**  **8416**  **9145**  **4890**  **5688**  **17851**  **10527**  **6375**  **8764**  **6995**  **4720**  **14810**  **7743** | **3495**  **3447**  **3456**  **3466**  **3441**  **3430**  **3454**  **3435**  **3450**  **3475**  **3420**  **3408**  **3435**  **3414**  **3428**  **3452**  **3432**  **3420** | **3670**  **3621**  **3630**  **3640**  **3615**  **3603**  **3628**  **3608**  **3624**  **3650**  **3594**  **3582**  **3608**  **3587**  **3603**  **3627**  **3604**  **3592** |
|  | **34**  **35**  **36**  **37**  **38**  **39**  **40**  **41** | **7/6/05**  **7/6/05**  **7/6/05**  **7/6/05**  **7/6/05**  **7/6/05**  **7/6/05**  **7/6/05** | **CD006B1_M35**  **CD006B1_M46**  **CD006B1_M65**  **CD006B1_M14**  **CD006B1_M26**  **CD006B1_M36**  **CD006B1_M47**  **CD006B1_M67** | **D3S1262**  **D3S1262**  **D3S1262**  **D3S1262**  **D3S1262**  **D3S1262**  **D3S1262**  **D3S1262** | **132**  **132**  **132**  **132**  **132**  **132**  **132**  **132** | **146**  **146**  **146**  **146**  **146**  **146**  **146**  **146** | **131.6**  **131.7**  **131.6**  **131.6**  **131.6**  **131.6**  **131.6**  **131.7** | **146.1**  **146.2**  **146.1**  **146.1**  **146.1**  **146.2**  **146.1**  **146.2** | **1498**  **1580**  **1065**  **1446**  **1791**  **990**  **1603**  **516** | **1245**  **1256**  **701**  **1150**  **1355**  **653**  **1164**  **563** | **16649**  **17302**  **10921**  **18347**  **20214**  **10387**  **16300**  **5428** | **12579**  **12206**  **6806**  **13149**  **14014**  **6398**  **11090**  **5473** | **3443**  **3403**  **3419**  **3440**  **3431**  **3456**  **3437**  **3450** | **3616**  **3574**  **3591**  **3614**  **3605**  **3631**  **3611**  **3625** |
| **1** | **1**  **2**  **3**  **4**  **5**  **6**  **7**  **8**  **9**  **10**  **11**  **12**  **13**  **14**  **15**  **16**  **17**  **18**  **19** | **7/6/05**  **7/6/05**  **7/6/05**  **7/6/05**  **7/6/05**  **7/6/05**  **7/6/05**  **7/6/05**  **7/6/05**  **7/6/05**  **7/6/05**  **7/6/05**  **7/6/05**  **7/6/05**  **7/6/05**  **7/6/05**  **7/6/05**  **7/6/05**  **7/6/05** | **CD006B1_M2**  **CD006B1_M15**  **CD006B1_M27**  **CD006B1_M37**  **CD006B1_M48**  **CD006B1_M3**  **CD006B1_M28**  **CD006B1_M39**  **CD006B1_M51* CD006B1_M4**  **CD006B1_M29**  **CD006B1_M40**  **CD006B1_M52**  **CD006B1_M5**  **CD006B1_M20**  **CD006B1_M31**  **CD006B1_M41**  **CD006B1_M53**  **CD006B1_M73** | **D9S171**  **D9S171**  **D9S171**  **D9S171**  **D9S171**  **D9S171**  **D9S171**  **D9S171**  **D9S171* D9S171**  **D9S171**  **D9S171**  **D9S171**  **D9S171**  **D9S171**  **D9S171**  **D9S171**  **D9S171**  **D9S171** | **111**  **111**  **111**  **111**  **111**  **111**  **111**  **111**  **113**  **111**  **111**  **111**  **111**  **111**  **111**  **111**  **111**  **111**  **111** | **113**  **113**  **113**  **113**  **113**  **113**  **113**  **113**  **113**  **113**  **113**  **113**  **113**  **113**  **113**  **113**  **113**  **113** | **111.1**  **111.0**  **111.1**  **111.1**  **111.0**  **111.0**  **111.0**  **111.1**  **112.9**  **111.0**  **111.1**  **111.1**  **111.0**  **111.1**  **111.0**  **111.1**  **111.1**  **111.1**  **111.1** | **113.0**  **112.9**  **113.0**  **113.0**  **112.9**  **113.0**  **113.0**  **112.9**  **112.9**  **112.9**  **113.0**  **112.9**  **113.0**  **112.9**  **112.9**  **113.0**  **112.9**  **113.0** | **1541**  **1543**  **1083**  **637**  **631**  **1342**  **653**  **871**  **746**  **1582**  **492**  **636**  **426**  **2090**  **1459**  **843**  **1293**  **1059**  **998** | **1180**  **1135**  **762**  **458**  **606**  **944**  **495**  **606**  **1186**  **359**  **481**  **383**  **1566**  **1057**  **596**  **966**  **852**  **726** | **12348**  **12277**  **8634**  **5099**  **5013**  **10708**  **5263**  **6766**  **6088**  **12693**  **4049**  **4867**  **3401**  **16440**  **11382**  **6804**  **10090**  **8657**  **8033** | **8944**  **9577**  **5943**  **3953**  **4986**  **7729**  **3919**  **4578**  **8783**  **2851**  **3592**  **3022**  **11943**  **8072**  **4659**  **7934**  **6695**  **5902** | **3204**  **3154**  **3223**  **3178**  **3178**  **3179**  **3203**  **3158**  **3181**  **3193**  **3216**  **3169**  **3147**  **3201**  **3159**  **3224**  **3179**  **3186**  **3196** | **3230**  **3179**  **3249**  **3203**  **3203**  **3205**  **3229**  **3182**  **3218**  **3241**  **3194**  **3172**  **3227**  **3184**  **3249**  **3205**  **3211**  **3222** |

| **Day** | **Count** | **Run Date** | **Sample Name** | **Marker** | **Allele 1** | **Allele 2** | **Size 1** | **Size 2** | **Height 1** | **Height 2** | **Peak Area 1** | **Peak Area 2** | **Data Point 1** | **Data Point 2** |
| --- | --- | --- | --- | --- | --- | --- | --- | --- | --- | --- | --- | --- | --- | --- |
|  | **20**  **21**  **22**  **23**  **24**  **25**  **26**  **27**  **28**  **29** | **7/6/05**  **7/6/05**  **7/6/05**  **7/6/05**  **7/6/05**  **7/6/05**  **7/6/05**  **7/6/05**  **7/6/05**  **7/6/05** | **CD006B1_M6**  **CD006B1_M23**  **CD006B1_M32**  **CD006B1_M43**  **CD006B1_M54**  **CD006B1_M75**  **CD006B1_M8**  **CD006B1_M24**  **CD006B1_M33**  **CD006B1_M45** | **D9S171**  **D9S171**  **D9S171**  **D9S171**  **D9S171**  **D9S171**  **D9S171**  **D9S171**  **D9S171**  **D9S171** | **111**  **111**  **111**  **111**  **111**  **111**  **111**  **111**  **111**  **111** | **113**  **113**  **113**  **113**  **113**  **113**  **113**  **113**  **113**  **113** | **111.0**  **111.0**  **111.1**  **111.0**  **111.1**  **111.0**  **111.0**  **111.1**  **111.0**  **111.0** | **112.9**  **112.9**  **112.9**  **112.9**  **112.9**  **113.0**  **112.9**  **113.0**  **113.0**  **113.0** | **1354**  **1293**  **934**  **960**  **629**  **494**  **1467**  **964**  **603**  **901** | **966**  **917**  **665**  **646**  **418**  **362**  **1021**  **715**  **500**  **698** | **10968**  **10037**  **7413**  **7483**  **5190**  **3910**  **11403**  **7703**  **4828**  **7003** | **8289**  **6967**  **5277**  **4867**  **3288**  **2923**  **7725**  **5489**  **3814**  **5297** | **3173**  **3162**  **3186**  **3167**  **3182**  **3205**  **3154**  **3142**  **3167**  **3147** | **3198**  **3187**  **3211**  **3192**  **3207**  **3231**  **3179**  **3167**  **3194**  **3173** |
|  | **30**  **31**  **32**  **33**  **34**  **35**  **36**  **37**  **38**  **39**  **40**  **41** | **7/6/05**  **7/6/05**  **7/6/05**  **7/6/05**  **7/6/05**  **7/6/05**  **7/6/05**  **7/6/05**  **7/6/05**  **7/6/05**  **7/6/05**  **7/6/05** | **CD006B1_M55**  **CD006B1_M82**  **CD006B1_M9**  **CD006B1_M25**  **CD006B1_M35**  **CD006B1_M46**  **CD006B1_M65**  **CD006B1_M14**  **CD006B1_M26**  **CD006B1_M36**  **CD006B1_M47**  **CD006B1_M67** | **D9S171**  **D9S171**  **D9S171**  **D9S171**  **D9S171**  **D9S171**  **D9S171**  **D9S171**  **D9S171**  **D9S171**  **D9S171**  **D9S171** | **111**  **111**  **111**  **111**  **111**  **111**  **111**  **111**  **111**  **111**  **111**  **111** | **113**  **113**  **113**  **113**  **113**  **113**  **113**  **113**  **113**  **113**  **113**  **113** | **111.0**  **111.0**  **111.1**  **111.1**  **111.0**  **111.0**  **111.0**  **111.1**  **111.1**  **111.0**  **111.0**  **111.1** | **112.9**  **113.0**  **113.0**  **113.0**  **112.9**  **112.9**  **112.9**  **112.9**  **112.9**  **113.0**  **112.9**  **113.0** | **792**  **564**  **1563**  **815**  **1068**  **1248**  **841**  **1714**  **1556**  **853**  **1132**  **897** | **599**  **430**  **1096**  **567**  **756**  **887**  **669**  **1081**  **1141**  **666**  **768**  **701** | **6107**  **4595**  **12207**  **6470**  **8824**  **9681**  **6446**  **13934**  **12302**  **6837**  **9169**  **7373** | **4581**  **3563**  **7819**  **4172**  **5682**  **6489**  **5193**  **7806**  **8759**  **5283**  **5849**  **5601** | **3161**  **3183**  **3166**  **3154**  **3176**  **3138**  **3154**  **3173**  **3164**  **3188**  **3169**  **3182** | **3186**  **3209**  **3191**  **3179**  **3202**  **3163**  **3179**  **3198**  **3189**  **3214**  **3194**  **3208** |
| **1** | **1**  **2**  **3**  **4**  **5**  **6**  **7**  **8**  **9**  **10**  **11**  **12**  **13**  **14**  **15**  **16**  **17**  **18** | **7/7/05**  **7/7/05**  **7/7/05**  **7/7/05**  **7/7/05**  **7/7/05**  **7/7/05**  **7/7/05**  **7/7/05**  **7/7/05**  **7/7/05**  **7/7/05**  **7/7/05**  **7/7/05**  **7/7/05**  **7/7/05**  **7/7/05**  **7/7/05** | **CD009B1_M1**  **CD009B1_M11**  **CD009B1_M27**  **CD009B1_M70**  **CD009B1_M30**  **CD009B1_M82**  **CD009B1_M15**  **CD009B1_M42**  **CD009B1_M103* CD009B1_M16**  **CD009B1_M33**  **CD009B1_M43**  **CD009B1_M108**  **CD009B1_M6**  **CD009B1_M34**  **CD009B1_M117**  **CD009B1_M8**  **CD009B1_M22** | **D17S250**  **D17S250**  **D17S250**  **D17S250**  **D17S250**  **D17S250**  **D17S250**  **D17S250**  **D17S250* D17S250**  **D17S250**  **D17S250**  **D17S250**  **D17S250**  **D17S250**  **D17S250**  **D17S250**  **D17S250** | **191**  **191**  **191**  **191**  **191**  **191**  **191**  **191**  **195**  **191**  **191**  **191**  **191**  **191**  **191**  **191**  **191**  **191** | **195**  **195**  **195**  **195**  **195**  **195**  **195**  **195**  **195**  **195**  **195**  **195**  **195**  **195**  **195**  **195**  **195** | **191.3**  **191.3**  **191.4**  **191.4**  **191.3**  **191.4**  **191.4**  **191.4**  **195.5**  **191.3**  **191.4**  **191.4**  **191.3**  **191.4**  **191.4**  **191.3**  **191.3**  **191.3** | **195.5**  **195.4**  **195.5**  **195.5**  **195.4**  **195.5**  **195.5**  **195.5**  **195.5**  **195.5**  **195.5**  **195.5**  **195.4**  **195.4**  **195.4**  **195.4**  **195.4** | **221**  **1078**  **362**  **1087**  **542**  **978**  **1452**  **1499**  **582**  **222**  **1189**  **1129**  **1305**  **811**  **974**  **526**  **1110**  **1060** | **193**  **709**  **267**  **863**  **402**  **621**  **1228**  **1200**  **238**  **954**  **880**  **1021**  **589**  **746**  **415**  **923**  **845** | **1918**  **9130**  **3010**  **9317**  **4401**  **8132**  **12143**  **12476**  **4969**  **1766**  **10201**  **9341**  **10877**  **6589**  **8064**  **4288**  **9298**  **8833** | **1661**  **5703**  **2065**  **6891**  **3292**  **5111**  **9916**  **10034**  **1871**  **7943**  **7248**  **8459**  **4735**  **6078**  **3272**  **7310**  **6602** | **4149**  **4159**  **4176**  **4172**  **4146**  **4141**  **4173**  **4207**  **4235**  **4160**  **4199**  **4170**  **4174**  **4121**  **4167**  **4140**  **4111**  **4116** | **4200**  **4210**  **4227**  **4223**  **4197**  **4192**  **4224**  **4259**  **4212**  **4250**  **4221**  **4226**  **4171**  **4217**  **4191**  **4161**  **4166** |
| **2** | **19**  **20**  **21**  **22**  **23**  **24**  **25** | **7/7/05**  **7/7/05**  **7/13/05**  **7/13/05**  **7/13/05**  **7/13/05**  **7/13/05** | **CD009B1_M46**  **CD009B1_M51**  **CD009B1M5_1uL CD009B1M12_1uL CD009B1M9_1uL CD009B1M37_1uL* CD009B1M84_1uL** | **D17S250**  **D17S250**  **D17S250**  **D17S250**  **D17S250**  **D17S250* D17S250** | **191**  **191**  **191**  **191**  **191**  **191**  **191** | **195**  **195**  **195**  **195**  **195**  **195** | **191.3**  **191.3**  **191.6**  **191.6**  **191.6**  **191.6**  **191.7** | **195.4**  **195.5**  **195.7**  **195.7**  **195.7**  **195.8** | **536**  **1339**  **412**  **712**  **284**  **322**  **507** | **230**  **1046**  **292**  **509**  **170**  **282** | **4571**  **11505**  **3793**  **6574**  **2589**  **2919**  **4744** | **1852**  **8795**  **2761**  **4523**  **1491**  **2456** | **4127**  **4179**  **4533**  **4568**  **4546**  **4554**  **4569** | **4178**  **4231**  **4588**  **4623**  **4600**  **4624** |
| **1** | **1**  **2**  **3**  **4**  **5**  **6** | **7/7/05**  **7/7/05**  **7/7/05**  **7/7/05**  **7/7/05**  **7/7/05** | **CD009B1_M1**  **CD009B1_M11**  **CD009B1_M27**  **CD009B1_M70**  **CD009B1_M30**  **CD009B1_M82** | **D18S61**  **D18S61**  **D18S61**  **D18S61**  **D18S61**  **D18S61** | **154**  **154**  **154**  **154**  **154**  **154** | **170**  **170**  **170**  **170**  **170**  **170** | **153.7**  **153.8**  **153.8**  **153.8**  **153.8**  **153.8** | **169.7**  **169.8**  **169.8**  **169.8**  **169.8**  **169.8** | **1629**  **2231**  **1337**  **2393**  **1217**  **2919** | **715**  **1446**  **685**  **1482**  **676**  **1522** | **14620**  **19498**  **11507**  **20375**  **10704**  **25551** | **6294**  **13190**  **5917**  **12968**  **5692**  **13078** | **3685**  **3695**  **3710**  **3707**  **3682**  **3678** | **3881**  **3891**  **3906**  **3903**  **3878**  **3874** |

| **Day** | **Count** | **Run Date** | **Sample Name** | **Marker** | **Allele 1** | **Allele 2** | **Size 1** | **Size 2** | **Height 1** | **Height 2** | **Peak Area 1** | **Peak Area 2** | **Data Point 1** | **Data Point 2** |
| --- | --- | --- | --- | --- | --- | --- | --- | --- | --- | --- | --- | --- | --- | --- |
|  | **7**  **8**  **9**  **10**  **11**  **12**  **13**  **14**  **15**  **16**  **17**  **18** | **7/7/05**  **7/7/05**  **7/7/05**  **7/7/05**  **7/7/05**  **7/7/05**  **7/7/05**  **7/7/05**  **7/7/05**  **7/7/05**  **7/7/05**  **7/7/05** | **CD009B1_M15**  **CD009B1_M42**  **CD009B1_M103**  **CD009B1_M16**  **CD009B1_M33**  **CD009B1_M43**  **CD009B1_M108**  **CD009B1_M6**  **CD009B1_M34**  **CD009B1_M117**  **CD009B1_M8**  **CD009B1_M22** | **D18S61**  **D18S61**  **D18S61**  **D18S61**  **D18S61**  **D18S61**  **D18S61**  **D18S61**  **D18S61**  **D18S61**  **D18S61**  **D18S61** | **154**  **154**  **154**  **154**  **154**  **154**  **154**  **154**  **154**  **154**  **154**  **154** | **170**  **170**  **170**  **170**  **170**  **170**  **170**  **170**  **170**  **170**  **170**  **170** | **153.8**  **153.8**  **153.8**  **153.7**  **153.8**  **153.8**  **153.8**  **153.7**  **153.8**  **153.8**  **153.8**  **153.8** | **169.9**  **169.8**  **169.8**  **169.7**  **169.8**  **169.8**  **169.8**  **169.7**  **169.8**  **169.8**  **169.7**  **169.8** | **3031**  **3569**  **1021**  **250**  **2580**  **2555**  **2899**  **1290**  **1981**  **1316**  **2419**  **2466** | **1891**  **2151**  **591**  **727**  **1700**  **1543**  **1902**  **930**  **1330**  **1043**  **1725**  **1610** | **25220**  **31725**  **9190**  **1957**  **21947**  **22456**  **24523**  **11235**  **17754**  **11266**  **21486**  **22033** | **16601**  **18739**  **4921**  **6303**  **15009**  **13297**  **16534**  **8038**  **11383**  **8713**  **14442**  **13772** | **3707**  **3738**  **3717**  **3696**  **3730**  **3705**  **3709**  **3659**  **3701**  **3678**  **3655**  **3659** | **3904**  **3936**  **3914**  **3892**  **3928**  **3901**  **3905**  **3854**  **3897**  **3874**  **3847**  **3852** |
| **2** | **19**  **20**  **21**  **22**  **23**  **24**  **25** | **7/7/05**  **7/7/05**  **7/13/05**  **7/13/05**  **7/13/05**  **7/13/05**  **7/13/05** | **CD009B1_M46**  **CD009B1_M51**  **CD009B1M5_1uL CD009B1M12_1uL CD009B1M9_1uL CD009B1M37_1uL* CD009B1M84_1uL** | **D18S61**  **D18S61**  **D18S61**  **D18S61**  **D18S61**  **D18S61* D18S61** | **154**  **154**  **154**  **154**  **154**  **154**  **154** | **170**  **170**  **170**  **170**  **170**  **170** | **153.7**  **153.8**  **154.2**  **154.1**  **154.1**  **154.1**  **154.1** | **169.8**  **169.8**  **170.1**  **170.1**  **170.1**  **170.1** | **286**  **3809**  **1223**  **1719**  **313**  **936**  **709** | **231**  **2152**  **695**  **834**  **217**  **458** | **2468**  **34033**  **11270**  **16457**  **2658**  **8584**  **6535** | **1978**  **18646**  **6207**  **7673**  **1862**  **3936** | **3669**  **3713**  **4039**  **4070**  **4052**  **4058**  **4072** | **3863**  **3910**  **4248**  **4280**  **4261**  **4282** |
| **1**  **2** | **1**  **2**  **3**  **4**  **5**  **6**  **7**  **8**  **9**  **10**  **11**  **12**  **13**  **14**  **15**  **16**  **17**  **18**  **19**  **20**  **21**  **22**  **23**  **24**  **25** | **7/7/05**  **7/7/05**  **7/7/05**  **7/7/05**  **7/7/05**  **7/7/05**  **7/7/05**  **7/7/05**  **7/7/05**  **7/7/05**  **7/7/05**  **7/7/05**  **7/7/05**  **7/7/05**  **7/7/05**  **7/7/05**  **7/7/05**  **7/7/05**  **7/7/05**  **7/7/05**  **7/13/05**  **7/13/05**  **7/13/05**  **7/13/05**  **7/13/05** | **CD009B1_M1**  **CD009B1_M11**  **CD009B1_M27**  **CD009B1_M70**  **CD009B1_M30**  **CD009B1_M82**  **CD009B1_M15**  **CD009B1_M42**  **CD009B1_M103**  **CD009B1_M16**  **CD009B1_M33**  **CD009B1_M43**  **CD009B1_M108**  **CD009B1_M6**  **CD009B1_M34**  **CD009B1_M117**  **CD009B1_M8**  **CD009B1_M22**  **CD009B1_M46**  **CD009B1_M51**  **CD009B1M5_1uL CD009B1M12_1uL CD009B1M9_1uL CD009B1M37_1uL CD009B1M84_1uL** | **D2S123**  **D2S123**  **D2S123**  **D2S123**  **D2S123**  **D2S123**  **D2S123**  **D2S123**  **D2S123**  **D2S123**  **D2S123**  **D2S123**  **D2S123**  **D2S123**  **D2S123**  **D2S123**  **D2S123**  **D2S123**  **D2S123**  **D2S123**  **D2S123**  **D2S123**  **D2S123**  **D2S123**  **D2S123** | **219**  **219**  **219**  **219**  **219**  **219**  **219**  **219**  **219**  **219**  **219**  **219**  **219**  **219**  **219**  **219**  **219**  **219**  **219**  **219**  **219**  **219**  **219**  **219**  **219** |  | **218.4**  **218.4**  **218.5**  **218.5**  **218.5**  **218.4**  **218.6**  **218.6**  **218.6**  **218.5**  **218.6**  **218.4**  **218.5**  **218.5**  **218.5**  **218.4**  **218.4**  **218.4**  **218.4**  **218.5**  **218.8**  **218.9**  **218.7**  **218.8**  **218.8** |  | **625**  **1559**  **521**  **2330**  **835**  **1563**  **2508**  **1787**  **391**  **246**  **1924**  **1496**  **2419**  **1025**  **1525**  **1030**  **2131**  **1570**  **372**  **2576**  **652**  **1000**  **203**  **215**  **522** |  | **5550**  **14165**  **4730**  **20798**  **7483**  **14324**  **22586**  **16946**  **3706**  **2120**  **18584**  **14037**  **21448**  **9342**  **14348**  **9149**  **19588**  **14867**  **3251**  **24630**  **6254**  **9781**  **1945**  **2015**  **4998** |  | **4463**  **4474**  **4492**  **4488**  **4462**  **4455**  **4490**  **4526**  **4501**  **4476**  **4517**  **4485**  **4491**  **4435**  **4483**  **4455**  **4421**  **4426**  **4439**  **4496**  **4870**  **4908**  **4881**  **4892**  **4907** |  |
| **1** | **1**  **2**  **3**  **4**  **5**  **6**  **7**  **8**  **9**  **10** | **7/7/05**  **7/7/05**  **7/7/05**  **7/7/05**  **7/7/05**  **7/7/05**  **7/7/05**  **7/7/05**  **7/7/05**  **7/7/05** | **CD009B1_M1**  **CD009B1_M11**  **CD009B1_M27**  **CD009B1_M70**  **CD009B1_M30**  **CD009B1_M82**  **CD009B1_M15**  **CD009B1_M42**  **CD009B1_M103**  **CD009B1_M16** | **D3S1262**  **D3S1262**  **D3S1262**  **D3S1262**  **D3S1262**  **D3S1262**  **D3S1262**  **D3S1262**  **D3S1262**  **D3S1262** | **132**  **132**  **132**  **132**  **132**  **132**  **132**  **132**  **132**  **132** | **136**  **136**  **136**  **136**  **136**  **136**  **136**  **136**  **136**  **136** | **131.6**  **131.6**  **131.6**  **131.6**  **131.7**  **131.6**  **131.7**  **131.7**  **131.6**  **131.7** | **135.6**  **135.6**  **135.6**  **135.6**  **135.6**  **135.5**  **135.7**  **135.6**  **135.5**  **135.7** | **351**  **1433**  **1701**  **2723**  **998**  **1242**  **2710**  **2007**  **138**  **425** | **442**  **1631**  **1970**  **2744**  **1177**  **1445**  **2933**  **2185**  **170**  **339** | **3892**  **15521**  **17444**  **28771**  **11065**  **13472**  **29887**  **23235**  **1459**  **4452** | **4915**  **17669**  **21103**  **29513**  **12977**  **15678**  **32532**  **24570**  **1891**  **3553** | **3424**  **3433**  **3447**  **3444**  **3421**  **3417**  **3445**  **3474**  **3454**  **3435** | **3474**  **3483**  **3497**  **3494**  **3470**  **3466**  **3495**  **3524**  **3503**  **3485** |

| **Day** | **Count** | **Run Date** | **Sample Name** | **Marker** | **Allele 1** | **Allele 2** | **Size 1** | **Size 2** | **Height 1** | **Height 2** | **Peak Area 1** | **Peak Area 2** | **Data Point 1** | **Data Point 2** |
| --- | --- | --- | --- | --- | --- | --- | --- | --- | --- | --- | --- | --- | --- | --- |
| **2** | **11**  **12**  **13**  **14**  **15**  **16**  **17**  **18**  **19**  **20**  **21**  **22**  **23** | **7/7/05**  **7/7/05**  **7/7/05**  **7/7/05**  **7/7/05**  **7/7/05**  **7/7/05**  **7/7/05**  **7/7/05**  **7/7/05**  **7/13/05**  **7/13/05**  **7/13/05** | **CD009B1_M33**  **CD009B1_M43**  **CD009B1_M108**  **CD009B1_M6**  **CD009B1_M34**  **CD009B1_M117**  **CD009B1_M8**  **CD009B1_M22**  **CD009B1_M46**  **CD009B1_M51**  **CD009B1M5_1uL CD009B1M12_1uL CD009B1M9_1uL** | **D3S1262**  **D3S1262**  **D3S1262**  **D3S1262**  **D3S1262**  **D3S1262**  **D3S1262**  **D3S1262**  **D3S1262**  **D3S1262**  **D3S1262**  **D3S1262**  **D3S1262** | **132**  **132**  **132**  **132**  **132**  **132**  **132**  **132**  **132**  **132**  **132**  **132**  **132** | **136**  **136**  **136**  **136**  **136**  **136**  **136**  **136**  **136**  **136**  **136**  **136**  **136** | **131.6**  **131.6**  **131.6**  **131.6**  **131.6**  **131.6**  **131.7**  **131.6**  **131.6**  **131.6**  **132.0**  **131.9**  **131.8** | **135.6**  **135.6**  **135.5**  **135.7**  **135.7**  **135.6**  **135.6**  **135.6**  **135.7**  **135.6**  **135.9**  **135.8**  **135.8** | **2077**  **1669**  **2863**  **1285**  **1723**  **585**  **2263**  **1923**  **244**  **2308**  **1663**  **1538**  **445** | **2296**  **1823**  **2977**  **1471**  **1795**  **579**  **2316**  **2094**  **287**  **2432**  **1655**  **1916**  **420** | **22767**  **17524**  **30913**  **13264**  **18584**  **6192**  **24961**  **20368**  **2511**  **26646**  **20630**  **19296**  **5478** | **25300**  **19204**  **31454**  **15723**  **19729**  **6243**  **24618**  **22318**  **3012**  **27487**  **20785**  **24544**  **5103** | **3466**  **3442**  **3447**  **3400**  **3438**  **3417**  **3397**  **3400**  **3410**  **3450**  **3760**  **3789**  **3773** | **3516**  **3492**  **3496**  **3450**  **3489**  **3467**  **3446**  **3449**  **3460**  **3500**  **3812**  **3842**  **3826** |
|  | **24**  **25** | **7/13/05**  **7/13/05** | **CD009B1M37_1uL CD009B1M84_1uL** | **D3S1262**  **D3S1262** | **132**  **132** | **136**  **136** | **132.0**  **131.9** | **135.8**  **135.8** | **562**  **1112** | **335**  **903** | **6890**  **14260** | **4276**  **11577** | **3777**  **3792** | **3829**  **3845** |
| **1**  **2** | **1**  **2**  **3**  **4**  **5**  **6**  **7**  **8**  **9**  **10**  **11**  **12**  **13**  **14**  **15**  **16**  **17**  **18**  **19**  **20**  **21**  **22**  **23**  **24**  **25** | **7/7/05**  **7/7/05**  **7/7/05**  **7/7/05**  **7/7/05**  **7/7/05**  **7/7/05**  **7/7/05**  **7/7/05**  **7/7/05**  **7/7/05**  **7/7/05**  **7/7/05**  **7/7/05**  **7/7/05**  **7/7/05**  **7/7/05**  **7/7/05**  **7/7/05**  **7/7/05**  **7/13/05**  **7/13/05**  **7/13/05**  **7/13/05**  **7/13/05** | **CD009B1_M1**  **CD009B1_M11**  **CD009B1_M27**  **CD009B1_M70**  **CD009B1_M30**  **CD009B1_M82**  **CD009B1_M15**  **CD009B1_M42**  **CD009B1_M103**  **CD009B1_M16**  **CD009B1_M33**  **CD009B1_M43**  **CD009B1_M108**  **CD009B1_M6**  **CD009B1_M34**  **CD009B1_M117**  **CD009B1_M8* CD009B1_M22**  **CD009B1_M46**  **CD009B1_M51**  **CD009B1M5_1uL CD009B1M12_1uL CD009B1M9_1uL CD009B1M37_1uL CD009B1M84_1uL** | **D9S171**  **D9S171**  **D9S171**  **D9S171**  **D9S171**  **D9S171**  **D9S171**  **D9S171**  **D9S171**  **D9S171**  **D9S171**  **D9S171**  **D9S171**  **D9S171**  **D9S171**  **D9S171**  **D9S171* D9S171**  **D9S171**  **D9S171**  **D9S171**  **D9S171**  **D9S171**  **D9S171**  **D9S171** | **115**  **115**  **115**  **115**  **115**  **115**  **115**  **115**  **121**  **115**  **115**  **115**  **115**  **115**  **115**  **115**  **115**  **115**  **115**  **115**  **115**  **115**  **115**  **115**  **115** | **121**  **121**  **121**  **121**  **121**  **121**  **121**  **121**  **125**  **121**  **121**  **121**  **121**  **121**  **121**  **121**  **121**  **121**  **121**  **121**  **121**  **121**  **121**  **121**  **121** | **114.8**  **114.8**  **114.8**  **114.8**  **114.8**  **114.7**  **114.8**  **114.9**  **120.7**  **114.9**  **114.8**  **114.9**  **114.9**  **114.8**  **114.8**  **114.8**  **114.8**  **114.9**  **114.8**  **114.8**  **115.2**  **115.1**  **115.1**  **115.2**  **115.1** | **120.6**  **120.6**  **120.6**  **120.7**  **120.6**  **120.6**  **120.7**  **120.7**  **125.5**  **120.7**  **120.7**  **120.7**  **120.6**  **120.6**  **120.6**  **120.6**  **120.6**  **120.7**  **120.6**  **120.6**  **121.0**  **120.9**  **120.9**  **121.0**  **120.9** | **552**  **1094**  **1858**  **1818**  **561**  **859**  **1978**  **1477**  **348**  **342**  **1532**  **1140**  **2034**  **784**  **1154**  **486**  **1538**  **1262**  **171**  **1856**  **946**  **1425**  **538**  **200**  **614** | **454**  **815**  **1369**  **1489**  **565**  **714**  **1624**  **1181**  **405**  **186**  **1261**  **889**  **1702**  **733**  **977**  **437**  **1280**  **989**  **163**  **1485**  **766**  **1018**  **369**  **570**  **388** | **4434**  **8789**  **15578**  **14994**  **4371**  **7071**  **16319**  **11872**  **2996**  **2711**  **12541**  **9417**  **16630**  **6254**  **9652**  **3970**  **12503**  **10408**  **1321**  **15184**  **8111**  **12239**  **4870**  **1747**  **5531** | **3747**  **6453**  **11113**  **12215**  **4422**  **5492**  **12977**  **9753**  **5163**  **1577**  **10300**  **7230**  **13677**  **6019**  **7973**  **3502**  **10086**  **7852**  **1224**  **11962**  **6368**  **8418**  **2888**  **5045**  **3270** | **3208**  **3216**  **3229**  **3227**  **3204**  **3201**  **3227**  **3256**  **3313**  **3218**  **3248**  **3226**  **3230**  **3185**  **3221**  **3202**  **3183**  **3187**  **3196**  **3232**  **3530**  **3558**  **3543**  **3547**  **3561** | **3284**  **3292**  **3306**  **3304**  **3280**  **3277**  **3304**  **3333**  **3375**  **3294**  **3325**  **3302**  **3306**  **3260**  **3297**  **3277**  **3258**  **3262**  **3271**  **3309**  **3611**  **3639**  **3624**  **3627**  **3642** |
| **1** | **1**  **2**  **3**  **4**  **5**  **6**  **7**  **8** | **7/7/05**  **7/7/05**  **7/7/05**  **7/7/05**  **7/7/05**  **7/7/05**  **7/7/05**  **7/7/05** | **CD010B1_2M34**  **CD010B1_2M16**  **CD010B1_2M38**  **CD010B1_2M18**  **CD010B1_2M20**  **CD010B1_2M53**  **CD010B1_2M29**  **CD010B1_2M58** | **D17S250**  **D17S250**  **D17S250**  **D17S250**  **D17S250**  **D17S250**  **D17S250**  **D17S250** | **195**  **195**  **195**  **195**  **195**  **195**  **195**  **195** | **197**  **197**  **197**  **197**  **197**  **197**  **197**  **197** | **195.5**  **195.4**  **195.5**  **195.5**  **195.5**  **195.4**  **195.5**  **195.4** | **197.5**  **197.5**  **197.5**  **197.5**  **197.5**  **197.5**  **197.5**  **197.5** | **599**  **545**  **562**  **255**  **566**  **249**  **506**  **555** | **313**  **334**  **440**  **173**  **356**  **113**  **431**  **188** | **4928**  **4331**  **4728**  **2010**  **4891**  **2048**  **4073**  **4766** | **2450**  **2553**  **3244**  **1235**  **2783**  **854**  **3388**  **1389** | **4231**  **4194**  **4196**  **4190**  **4241**  **4237**  **4194**  **4196** | **4256**  **4220**  **4221**  **4214**  **4266**  **4263**  **4219**  **4221** |
| **1** | **1**  **2**  **3**  **4**  **5** | **7/7/05**  **7/7/05**  **7/7/05**  **7/7/05**  **7/7/05** | **CD010B1_2M34**  **CD010B1_2M16**  **CD010B1_2M38**  **CD010B1_2M18**  **CD010B1_2M20** | **D18S61**  **D18S61**  **D18S61**  **D18S61**  **D18S61** | **168**  **168**  **168**  **168**  **168** | **170**  **170**  **170**  **170**  **170** | **167.8**  **167.7**  **167.7**  **167.8**  **167.8** | **169.8**  **169.7**  **169.7**  **169.7**  **169.7** | **1399**  **1305**  **1507**  **726**  **1335** | **600**  **812**  **864**  **485**  **612** | **11484**  **10419**  **12435**  **5620**  **10994** | **4403**  **6052**  **6702**  **3416**  **4483** | **3885**  **3851**  **3852**  **3849**  **3894** | **3910**  **3875**  **3876**  **3873**  **3918** |

| **Day** | **Count** | **Run Date** | **Sample Name** | **Marker** | **Allele 1** | **Allele 2** | **Size 1** | **Size 2** | **Height 1** | **Height 2** | **Peak Area 1** | **Peak Area 2** | **Data Point 1** | **Data Point 2** |
| --- | --- | --- | --- | --- | --- | --- | --- | --- | --- | --- | --- | --- | --- | --- |
|  | **6**  **7**  **8** | **7/7/05**  **7/7/05**  **7/7/05** | **CD010B1_2M53**  **CD010B1_2M29**  **CD010B1_2M58** | **D18S61**  **D18S61**  **D18S61** | **168**  **168**  **168** | **170**  **170**  **170** | **167.7**  **167.6**  **167.7** | **169.7**  **169.7**  **169.6** | **855**  **551**  **606** | **588**  **351**  **476** | **6760**  **4391**  **4987** | **4526**  **2545**  **3691** | **3891**  **3850**  **3854** | **3916**  **3875**  **3878** |
| **1** | **1**  **2**  **3**  **4**  **5**  **6**  **7**  **8** | **7/7/05**  **7/7/05**  **7/7/05**  **7/7/05**  **7/7/05**  **7/7/05**  **7/7/05**  **7/7/05** | **CD010B1_2M34**  **CD010B1_2M16**  **CD010B1_2M38**  **CD010B1_2M18**  **CD010B1_2M20**  **CD010B1_2M53**  **CD010B1_2M29**  **CD010B1_2M58** | **D2S123**  **D2S123**  **D2S123**  **D2S123**  **D2S123**  **D2S123**  **D2S123**  **D2S123** | **221**  **221**  **221**  **221**  **221**  **221**  **221**  **221** | **236**  **236**  **236**  **236**  **236**  **236**  **236**  **236** | **220.7**  **220.6**  **220.6**  **220.6**  **220.7**  **220.7**  **220.7**  **220.7** | **236.0**  **235.9**  **235.9**  **235.8**  **236.0**  **236.0**  **236.0**  **235.9** | **344**  **611**  **665**  **352**  **393**  **112**  **341**  **325** | **280**  **591**  **687**  **293**  **407**  **118**  **273**  **316** | **2960**  **5635**  **5919**  **3128**  **3582**  **1062**  **3129**  **2994** | **2427**  **5348**  **6212**  **2656**  **3623**  **1021**  **2515**  **2898** | **4521**  **4482**  **4484**  **4475**  **4532**  **4528**  **4483**  **4483** | **4693**  **4654**  **4655**  **4644**  **4705**  **4700**  **4654**  **4652** |
| **1** | **1**  **2**  **3** | **7/7/05**  **7/7/05**  **7/7/05** | **CD010B1_2M34**  **CD010B1_2M16**  **CD010B1_2M38** | **D3S1262**  **D3S1262**  **D3S1262** | **136**  **136**  **136** |  | **135.6**  **135.6**  **135.6** |  | **1087**  **2524**  **1564** |  | **12334**  **29348**  **17713** |  | **3501**  **3469**  **3470** |  |
|  | **4** | **7/7/05** | **CD010B1_2M18** | **D3S1262** | **136** |  | **135.7** |  | **2177** |  | **25095** |  | **3470** |  |
|  | **5**  **6**  **7**  **8** | **7/7/05**  **7/7/05**  **7/7/05**  **7/7/05** | **CD010B1_2M20**  **CD010B1_2M53**  **CD010B1_2M29**  **CD010B1_2M58** | **D3S1262**  **D3S1262**  **D3S1262**  **D3S1262** | **136**  **136**  **136**  **136** |  | **135.6**  **135.6**  **135.6**  **135.5** |  | **1109**  **253**  **823**  **1242** |  | **13347**  **2794**  **9416**  **15352** |  | **3508**  **3507**  **3469**  **3474** |  |
| **1** | **1**  **2**  **3**  **4**  **5**  **6**  **7**  **8** | **7/7/05**  **7/7/05**  **7/7/05**  **7/7/05**  **7/7/05**  **7/7/05**  **7/7/05**  **7/7/05** | **CD010B1_2M34**  **CD010B1_2M16**  **CD010B1_2M38**  **CD010B1_2M18**  **CD010B1_2M20**  **CD010B1_2M53**  **CD010B1_2M29**  **CD010B1_2M58** | **D9S171**  **D9S171**  **D9S171**  **D9S171**  **D9S171**  **D9S171**  **D9S171**  **D9S171** | **105**  **105**  **105**  **105**  **105**  **105**  **105**  **105** | **119**  **119**  **119**  **119**  **119**  **119**  **119**  **119** | **105.3**  **105.2**  **105.2**  **105.3**  **105.3**  **105.2**  **105.3**  **105.3** | **118.7**  **118.8**  **118.7**  **118.7**  **118.7**  **118.7**  **118.7**  **118.7** | **647**  **1465**  **1402**  **1394**  **847**  **417**  **514**  **1051** | **467**  **810**  **863**  **786**  **533**  **331**  **354**  **664** | **5539**  **12158**  **11648**  **11495**  **6925**  **3582**  **4248**  **8777** | **3761**  **6575**  **6956**  **6232**  **4285**  **2684**  **2778**  **5534** | **3105**  **3076**  **3077**  **3079**  **3112**  **3110**  **3076**  **3084** | **3284**  **3255**  **3255**  **3256**  **3291**  **3290**  **3254**  **3261** |
| **1** | **1**  **2**  **3** | **7/13/05**  **7/13/05**  **7/13/05** | **CD013B1M15_1uL CD013B1M18_1uL CD013B1M5_1uL** | **D17S250**  **D17S250**  **D17S250** | **193**  **193**  **193** |  | **193.7**  **193.8**  **193.7** |  | **949**  **796**  **787** |  | **8992**  **7672**  **7361** |  | **4607**  **4618**  **4618** |  |
| **1** | **1**  **2**  **3** | **7/13/05**  **7/13/05**  **7/13/05** | **CD013B1M15_1uL CD013B1M18_1uL CD013B1M5_1uL*** | **D18S61**  **D18S61**  **D18S61*** | **168**  **168**  **168** | **176**  **176**  **174** | **168.0**  **168.2**  **168.1** | **175.9**  **175.9**  **174.0** | **833**  **600**  **661** | **607**  **541**  **281** | **7548**  **5445**  **5743** | **5586**  **5144**  **2524** | **4264**  **4276**  **4274** | **4369**  **4379**  **4353** |
| **1** | **1**  **2**  **3** | **7/13/05**  **7/13/05**  **7/13/05** | **CD013B1M15_1uL CD013B1M18_1uL CD013B1M5_1uL** | **D2S123**  **D2S123**  **D2S123** | **219**  **219**  **219** | **221**  **221**  **221** | **218.9**  **218.9**  **218.9** | **221.0**  **221.1**  **221.1** | **605**  **453**  **457** | **377**  **303**  **347** | **5535**  **4168**  **4491** | **3199**  **2579**  **3169** | **4919**  **4929**  **4931** | **4945**  **4955**  **4958** |
| **1** | **1**  **2**  **3** | **7/13/05**  **7/13/05**  **7/13/05** | **CD013B1M15_1uL CD013B1M18_1uL CD013B1M5_1uL*** | **D3S1262**  **D3S1262**  **D3S1262*** | **136**  **136**  **136** | **138**  **138** | **135.9**  **135.8**  **135.9** | **137.9**  **137.9** | **2539**  **1743**  **1397** | **1190**  **1009** | **32457**  **22722**  **18412** | **14175**  **12139** | **3853**  **3864**  **3861** | **3880**  **3891** |
| **1** | **1**  **2**  **3** | **7/13/05**  **7/13/05**  **7/13/05** | **CD013B1M15_1uL CD013B1M18_1uL CD013B1M5_1uL** | **D9S171**  **D9S171**  **D9S171** | **105**  **105**  **105** | **113**  **113**  **113** | **105.7**  **105.6**  **105.7** | **113.2**  **113.3**  **113.3** | **1886**  **1264**  **900** | **1379**  **848**  **749** | **16787**  **11673**  **7867** | **12262**  **8121**  **7084** | **3431**  **3444**  **3438** | **3540**  **3554**  **3548** |
| **1**  **2** | **1**  **2**  **3**  **4**  **5**  **6**  **7**  **8**  **9**  **10**  **11** | **7/13/05**  **7/13/05**  **7/13/05**  **7/13/05**  **7/13/05**  **7/13/05**  **7/14/05**  **7/14/05**  **7/14/05**  **7/14/05**  **7/14/05** | **CD015B1_M5**  **CD015B1_M10**  **CD015B1_M12**  **CD015B1_M1**  **CD015B1_M13**  **CD015B1_M14**  **CD015B1_M26**  **CD015B1_M50**  **CD015B1_M28**  **CD015B1_M38**  **CD015B1_M51** | **D17S250**  **D17S250**  **D17S250**  **D17S250**  **D17S250**  **D17S250**  **D17S250**  **D17S250**  **D17S250**  **D17S250**  **D17S250** | **191**  **191**  **191**  **191**  **191**  **191**  **191**  **191**  **191**  **191**  **191** |  | **191.5**  **191.6**  **191.6**  **191.5**  **191.6**  **191.5**  **191.4**  **191.4**  **191.3**  **191.3**  **191.4** |  | **1269**  **3307**  **933**  **2169**  **587**  **2389**  **3430**  **840**  **2983**  **4086**  **1967** |  | **11224**  **31036**  **8737**  **19627**  **5436**  **22372**  **29589**  **7165**  **25207**  **34748**  **16509** |  | **4468**  **4504**  **4524**  **4419**  **4491**  **4503**  **4263**  **4275**  **4238**  **4248**  **4251** |  |

| **Day** | **Count** | **Run Date** | **Sample Name** | **Marker** | **Allele 1** | **Allele 2** | **Size 1** | **Size 2** | **Height 1** | **Height 2** | **Peak Area 1** | **Peak Area 2** | **Data Point 1** | **Data Point 2** |
| --- | --- | --- | --- | --- | --- | --- | --- | --- | --- | --- | --- | --- | --- | --- |
|  | **12**  **13**  **14**  **15**  **16**  **17**  **18**  **19**  **20**  **21**  **22**  **23**  **24**  **25**  **26**  **27**  **28** | **7/14/05**  **7/14/05**  **7/14/05**  **7/14/05**  **7/14/05**  **7/14/05**  **7/14/05**  **7/14/05**  **7/14/05**  **7/14/05**  **7/14/05**  **7/14/05**  **7/14/05**  **7/14/05**  **7/14/05**  **7/14/05**  **7/14/05** | **CD015B1_M64**  **CD015B1_M15**  **CD015B1_M29**  **CD015B1_M39**  **CD015B1_M53**  **CD015B1_M17**  **CD015B1_M30**  **CD015B1_M42**  **CD015B1_M66**  **CD015B1_M31* CD015B1_M44**  **CD015B1_M32**  **CD015B1_M46**  **CD015B1_M56**  **CD015B1_M70**  **CD015B1_M21**  **CD015B1_M57** | **D17S250**  **D17S250**  **D17S250**  **D17S250**  **D17S250**  **D17S250**  **D17S250**  **D17S250**  **D17S250**  **D17S250* D17S250**  **D17S250**  **D17S250**  **D17S250**  **D17S250**  **D17S250**  **D17S250** | **191**  **191**  **191**  **191**  **191**  **191**  **191**  **191**  **191**  **191**  **191**  **191**  **191**  **191**  **191**  **191**  **191** |  | **191.4**  **191.5**  **191.3**  **191.3**  **191.4**  **191.6**  **191.3**  **191.4**  **191.5**  **191.5**  **191.4**  **191.4**  **191.4**  **191.4**  **191.4**  **191.4**  **191.4** |  | **890**  **4997**  **581**  **3018**  **1366**  **3585**  **4314**  **839**  **2805**  **1107**  **3059**  **4407**  **2723**  **536**  **1061**  **1158**  **1546** |  | **7376**  **44813**  **4980**  **25666**  **11995**  **31984**  **37015**  **7288**  **24551**  **9600**  **26354**  **37453**  **23624**  **4714**  **9099**  **9905**  **13330** |  | **4280**  **4298**  **4242**  **4254**  **4255**  **4310**  **4257**  **4261**  **4294**  **4292**  **4307**  **4267**  **4283**  **4298**  **4291**  **4270**  **4302** |  |
|  | **29**  **30**  **31**  **32**  **33** | **7/14/05**  **7/14/05**  **7/14/05**  **7/14/05**  **7/14/05** | **CD015B1_M72**  **CD015B1_M23**  **CD015B1_M36**  **CD015B1_M58**  **CD015B1_M73** | **D17S250**  **D17S250**  **D17S250**  **D17S250**  **D17S250** | **191**  **191**  **191**  **191**  **191** |  | **191.5**  **191.5**  **191.5**  **191.4**  **191.4** |  | **1669**  **2117**  **5018**  **2638**  **1583** |  | **14532**  **18651**  **43318**  **23095**  **13870** |  | **4295**  **4281**  **4282**  **4313**  **4308** |  |
| **1** | **1**  **2**  **3**  **4**  **5**  **6** | **7/13/05**  **7/13/05**  **7/13/05**  **7/13/05**  **7/13/05**  **7/13/05** | **CD015B1_M5**  **CD015B1_M10**  **CD015B1_M12**  **CD015B1_M1**  **CD015B1_M13**  **CD015B1_M14** | **D18S61**  **D18S61**  **D18S61**  **D18S61**  **D18S61**  **D18S61** | **170**  **170**  **170**  **170**  **170**  **170** | **176**  **176**  **176**  **176**  **176**  **176** | **170.0**  **170.0**  **170.1**  **170.0**  **170.0**  **169.9** | **175.8**  **175.8**  **175.9**  **175.8**  **175.8**  **175.8** | **781**  **2917**  **1549**  **1002**  **489**  **2076** | **619**  **2281**  **553**  **760**  **420**  **1373** | **6997**  **26696**  **13862**  **8827**  **4266**  **19270** | **5260**  **20917**  **4878**  **7104**  **3903**  **12688** | **4185**  **4219**  **4238**  **4139**  **4207**  **4219** | **4262**  **4296**  **4315**  **4215**  **4283**  **4296** |
| **2** | **7**  **8**  **9**  **10**  **11**  **12**  **13**  **14**  **15**  **16**  **17**  **18**  **19**  **20**  **21**  **22**  **23**  **24**  **25**  **26**  **27**  **28**  **29**  **30**  **31**  **32** | **7/14/05**  **7/14/05**  **7/14/05**  **7/14/05**  **7/14/05**  **7/14/05**  **7/14/05**  **7/14/05**  **7/14/05**  **7/14/05**  **7/14/05**  **7/14/05**  **7/14/05**  **7/14/05**  **7/14/05**  **7/14/05**  **7/14/05**  **7/14/05**  **7/14/05**  **7/14/05**  **7/14/05**  **7/14/05**  **7/14/05**  **7/14/05**  **7/14/05**  **7/14/05** | **CD015B1_M26**  **CD015B1_M50**  **CD015B1_M28**  **CD015B1_M38**  **CD015B1_M51**  **CD015B1_M64**  **CD015B1_M15**  **CD015B1_M29* CD015B1_M39**  **CD015B1_M53**  **CD015B1_M17**  **CD015B1_M30**  **CD015B1_M42**  **CD015B1_M66**  **CD015B1_M31**  **CD015B1_M44**  **CD015B1_M32**  **CD015B1_M46**  **CD015B1_M56**  **CD015B1_M70**  **CD015B1_M21**  **CD015B1_M57**  **CD015B1_M72**  **CD015B1_M23**  **CD015B1_M36**  **CD015B1_M58** | **D18S61**  **D18S61**  **D18S61**  **D18S61**  **D18S61**  **D18S61**  **D18S61**  **D18S61* D18S61**  **D18S61**  **D18S61**  **D18S61**  **D18S61**  **D18S61**  **D18S61**  **D18S61**  **D18S61**  **D18S61**  **D18S61**  **D18S61**  **D18S61**  **D18S61**  **D18S61**  **D18S61**  **D18S61**  **D18S61** | **170**  **170**  **170**  **170**  **170**  **170**  **170**  **168**  **170**  **170**  **170**  **170**  **170**  **170**  **170**  **170**  **170**  **170**  **170**  **170**  **170**  **170**  **170**  **170**  **170**  **170** | **176**  **176**  **176**  **176**  **176**  **176**  **176**  **176**  **176**  **176**  **176**  **176**  **176**  **176**  **176**  **176**  **176**  **176**  **176**  **176**  **176**  **176**  **176**  **176**  **176**  **176** | **169.8**  **169.8**  **169.7**  **169.8**  **169.8**  **169.8**  **169.9**  **167.8**  **169.8**  **169.8**  **169.9**  **169.8**  **169.8**  **169.8**  **169.9**  **169.8**  **169.8**  **169.8**  **169.9**  **169.9**  **169.8**  **169.9**  **169.9**  **169.9**  **169.8**  **169.8** | **175.7**  **175.6**  **175.5**  **175.7**  **175.6**  **175.6**  **175.7**  **175.6**  **175.6**  **175.7**  **175.8**  **175.6**  **175.6**  **175.7**  **175.7**  **175.7**  **175.7**  **175.7**  **175.7**  **175.6**  **175.6**  **175.7**  **175.7**  **175.8**  **175.7**  **175.7** | **2529**  **935**  **2453**  **3477**  **1662**  **558**  **3376**  **240**  **2964**  **896**  **1108**  **3655**  **1148**  **3146**  **2390**  **3335**  **3557**  **2139**  **228**  **1171**  **1268**  **1140**  **1699**  **2126**  **4426**  **2257** | **1730**  **741**  **1878**  **2401**  **1295**  **709**  **2620**  **166**  **1738**  **530**  **2533**  **2760**  **784**  **2611**  **1799**  **2388**  **2759**  **1667**  **345**  **983**  **590**  **1093**  **1163**  **3202**  **3268**  **1656** | **22716**  **8029**  **21815**  **31047**  **14236**  **4601**  **31493**  **2046**  **26288**  **7648**  **9188**  **31965**  **9842**  **27589**  **21500**  **29395**  **32543**  **18667**  **1872**  **10152**  **11568**  **9603**  **14546**  **18391**  **40132**  **20204** | **15723**  **6277**  **16819**  **21612**  **11014**  **6239**  **24908**  **1813**  **14969**  **4633**  **23277**  **24601**  **7047**  **22879**  **16227**  **21373**  **25169**  **14600**  **3129**  **8585**  **5591**  **9773**  **10076**  **29303**  **29516**  **15028** | **3990**  **4002**  **3967**  **3977**  **3979**  **4007**  **4025**  **3948**  **3984**  **3984**  **4035**  **3986**  **3990**  **4020**  **4017**  **4032**  **3994**  **4010**  **4024**  **4018**  **3999**  **4029**  **4022**  **4008**  **4008**  **4038** | **4064**  **4076**  **4040**  **4051**  **4052**  **4080**  **4099**  **4045**  **4057**  **4058**  **4110**  **4059**  **4063**  **4094**  **4091**  **4107**  **4068**  **4084**  **4098**  **4091**  **4072**  **4103**  **4096**  **4082**  **4082**  **4113** |

| **Day** | **Count** | **Run Date** | **Sample Name** | **Marker** | **Allele 1** | **Allele 2** | **Size 1** | **Size 2** | **Height 1** | **Height 2** | **Peak Area 1** | **Peak Area 2** | **Data Point 1** | **Data Point 2** |
| --- | --- | --- | --- | --- | --- | --- | --- | --- | --- | --- | --- | --- | --- | --- |
|  | **33** | **7/14/05** | **CD015B1_M73** | **D18S61** | **170** | **176** | **169.9** | **175.7** | **2324** | **1640** | **20594** | **14370** | **4035** | **4109** |
| **1**  **2** | **1**  **2**  **3**  **4**  **5**  **6**  **7**  **8**  **9**  **10**  **11**  **12**  **13**  **14**  **15**  **16**  **17** | **7/13/05**  **7/13/05**  **7/13/05**  **7/13/05**  **7/13/05**  **7/13/05**  **7/14/05**  **7/14/05**  **7/14/05**  **7/14/05**  **7/14/05**  **7/14/05**  **7/14/05**  **7/14/05**  **7/14/05**  **7/14/05**  **7/14/05** | **CD015B1_M5**  **CD015B1_M10**  **CD015B1_M12**  **CD015B1_M1**  **CD015B1_M13**  **CD015B1_M14**  **CD015B1_M26**  **CD015B1_M50**  **CD015B1_M28**  **CD015B1_M38**  **CD015B1_M51**  **CD015B1_M64**  **CD015B1_M15**  **CD015B1_M29**  **CD015B1_M39**  **CD015B1_M53**  **CD015B1_M17** | **D2S123**  **D2S123**  **D2S123**  **D2S123**  **D2S123**  **D2S123**  **D2S123**  **D2S123**  **D2S123**  **D2S123**  **D2S123**  **D2S123**  **D2S123**  **D2S123**  **D2S123**  **D2S123**  **D2S123** | **219**  **219**  **219**  **219**  **219**  **219**  **219**  **219**  **219**  **219**  **219**  **219**  **219**  **219**  **219**  **219**  **219** | **221**  **221**  **221**  **221**  **221**  **221**  **221**  **221**  **221**  **221**  **221**  **221**  **221**  **221**  **221**  **221**  **221** | **218.6**  **218.8**  **218.7**  **218.6**  **218.8**  **218.8**  **218.4**  **218.4**  **218.4**  **218.4**  **218.4**  **218.4**  **218.5**  **218.3**  **218.4**  **218.4**  **218.6** | **220.8**  **221.0**  **221.0**  **220.8**  **221.0**  **220.9**  **220.6**  **220.6**  **220.6**  **220.5**  **220.6**  **220.6**  **220.7**  **220.5**  **220.5**  **220.6**  **220.8** | **613**  **1701**  **1124**  **970**  **598**  **1298**  **1549**  **593**  **2254**  **2422**  **1434**  **1189**  **2994**  **526**  **1831**  **400**  **1139** | **325**  **1207**  **541**  **877**  **400**  **818**  **857**  **400**  **1482**  **1591**  **876**  **757**  **1990**  **197**  **1161**  **244**  **713** | **5514**  **15912**  **10559**  **8843**  **5672**  **12312**  **13955**  **5019**  **19566**  **21401**  **12492**  **10430**  **27992**  **4875**  **15659**  **3573**  **10271** | **2734**  **10658**  **4759**  **7853**  **3690**  **7011**  **7435**  **3203**  **11815**  **13541**  **7135**  **6399**  **18041**  **1665**  **9603**  **2092**  **6244** | **4800**  **4839**  **4860**  **4749**  **4826**  **4837**  **4582**  **4596**  **4556**  **4567**  **4570**  **4601**  **4619**  **4559**  **4571**  **4573**  **4632** | **4826**  **4866**  **4887**  **4775**  **4852**  **4863**  **4607**  **4621**  **4581**  **4591**  **4595**  **4626**  **4644**  **4583**  **4596**  **4598**  **4657** |
|  | **18**  **19**  **20**  **21**  **22**  **23**  **24**  **25**  **26**  **27**  **28**  **29**  **30**  **31**  **32**  **33** | **7/14/05**  **7/14/05**  **7/14/05**  **7/14/05**  **7/14/05**  **7/14/05**  **7/14/05**  **7/14/05**  **7/14/05**  **7/14/05**  **7/14/05**  **7/14/05**  **7/14/05**  **7/14/05**  **7/14/05**  **7/14/05** | **CD015B1_M30**  **CD015B1_M42**  **CD015B1_M66**  **CD015B1_M31**  **CD015B1_M44**  **CD015B1_M32**  **CD015B1_M46**  **CD015B1_M56**  **CD015B1_M70**  **CD015B1_M21**  **CD015B1_M57**  **CD015B1_M72**  **CD015B1_M23**  **CD015B1_M36**  **CD015B1_M58**  **CD015B1_M73** | **D2S123**  **D2S123**  **D2S123**  **D2S123**  **D2S123**  **D2S123**  **D2S123**  **D2S123**  **D2S123**  **D2S123**  **D2S123**  **D2S123**  **D2S123**  **D2S123**  **D2S123**  **D2S123** | **219**  **219**  **219**  **219**  **219**  **219**  **219**  **219**  **219**  **219**  **219**  **219**  **219**  **219**  **219**  **219** | **221**  **221**  **221**  **221**  **221**  **221**  **221**  **221**  **221**  **221**  **221**  **221**  **221**  **221**  **221**  **221** | **218.4**  **218.5**  **218.5**  **218.5**  **218.5**  **218.4**  **218.4**  **218.4**  **218.4**  **218.4**  **218.4**  **218.4**  **218.5**  **218.5**  **218.4**  **218.5** | **220.5**  **220.6**  **220.6**  **220.6**  **220.6**  **220.6**  **220.7**  **220.6**  **220.6**  **220.6**  **220.6**  **220.6**  **220.6**  **220.7**  **220.7**  **220.6** | **2215**  **1147**  **2525**  **2640**  **2379**  **2612**  **1741**  **360**  **1103**  **834**  **922**  **1265**  **1463**  **2845**  **1859**  **1788** | **1493**  **769**  **1731**  **1626**  **1672**  **1760**  **1117**  **306**  **846**  **459**  **739**  **771**  **1203**  **2011**  **1222**  **1361** | **19649**  **10251**  **22694**  **23763**  **20382**  **23150**  **15172**  **3137**  **9840**  **7215**  **8324**  **11236**  **13662**  **25569**  **16718**  **16135** | **12215**  **6447**  **14621**  **13897**  **13945**  **14667**  **9268**  **2620**  **7326**  **3640**  **6350**  **6638**  **10978**  **16945**  **10217**  **11866** | **4576**  **4580**  **4615**  **4614**  **4630**  **4588**  **4604**  **4620**  **4613**  **4588**  **4622**  **4614**  **4601**  **4603**  **4635**  **4630** | **4600**  **4604**  **4639**  **4639**  **4655**  **4613**  **4630**  **4645**  **4638**  **4613**  **4647**  **4640**  **4626**  **4628**  **4661**  **4655** |
| **1**  **2** | **1**  **2**  **3**  **4**  **5**  **6**  **7** | **7/13/05**  **7/13/05**  **7/13/05**  **7/13/05**  **7/13/05**  **7/13/05**  **7/14/05** | **CD015B1_M5**  **CD015B1_M10**  **CD015B1_M12* CD015B1_M1**  **CD015B1_M13**  **CD015B1_M14**  **CD015B1_M26** | **D3S1262**  **D3S1262**  **D3S1262* D3S1262**  **D3S1262**  **D3S1262**  **D3S1262** | **142**  **142**  **136**  **142**  **142**  **142**  **142** | **142** | **142.1**  **142.1**  **135.9**  **142.0**  **142.1**  **142.1**  **142.0** | **142.0** | **1072**  **3380**  **281**  **2140**  **831**  **2540**  **2266** | **451** | **11661**  **43919**  **3534**  **27129**  **10314**  **33598**  **27331** | **6056** | **3834**  **3865**  **3803**  **3790**  **3854**  **3866**  **3653** | **3881** |
|  | **8**  **9**  **10**  **11**  **12**  **13**  **14**  **15**  **16**  **17**  **18**  **19**  **20** | **7/14/05**  **7/14/05**  **7/14/05**  **7/14/05**  **7/14/05**  **7/14/05**  **7/14/05**  **7/14/05**  **7/14/05**  **7/14/05**  **7/14/05**  **7/14/05**  **7/14/05** | **CD015B1_M50**  **CD015B1_M28**  **CD015B1_M38**  **CD015B1_M51**  **CD015B1_M64**  **CD015B1_M15**  **CD015B1_M29* CD015B1_M39**  **CD015B1_M53**  **CD015B1_M17**  **CD015B1_M30**  **CD015B1_M42**  **CD015B1_M66** | **D3S1262**  **D3S1262**  **D3S1262**  **D3S1262**  **D3S1262**  **D3S1262**  **D3S1262* D3S1262**  **D3S1262**  **D3S1262**  **D3S1262**  **D3S1262**  **D3S1262** | **142**  **142**  **142**  **142**  **142**  **142**  **136**  **142**  **142**  **142**  **142**  **142**  **142** | **142** | **142.0**  **141.9**  **141.9**  **142.0**  **142.0**  **141.8**  **135.6**  **141.9**  **141.9**  **142.1**  **142.0**  **142.0**  **141.9** | **141.9** | **2435**  **6011**  **4367**  **2327**  **681**  **2025**  **178**  **4062**  **473**  **812**  **6576**  **678**  **2830** | **425** | **28238**  **70910**  **55049**  **25840**  **8071**  **27779**  **2061**  **48488**  **5462**  **10040**  **79295**  **8217**  **35744** | **5167** | **3663**  **3631**  **3640**  **3642**  **3667**  **3685**  **3562**  **3648**  **3648**  **3695**  **3649**  **3653**  **3680** | **3637** |

| **Day** | **Count** | **Run Date** | **Sample Name** | **Marker** | **Allele 1** | **Allele 2** | **Size 1** | **Size 2** | **Height 1** | **Height 2** | **Peak Area 1** | **Peak Area 2** | **Data Point 1** | **Data Point 2** |
| --- | --- | --- | --- | --- | --- | --- | --- | --- | --- | --- | --- | --- | --- | --- |
|  | **21**  **22**  **23**  **24**  **25**  **26**  **27**  **28**  **29**  **30**  **31**  **32**  **33** | **7/14/05**  **7/14/05**  **7/14/05**  **7/14/05**  **7/14/05**  **7/14/05**  **7/14/05**  **7/14/05**  **7/14/05**  **7/14/05**  **7/14/05**  **7/14/05**  **7/14/05** | **CD015B1_M31**  **CD015B1_M44**  **CD015B1_M32**  **CD015B1_M46**  **CD015B1_M56**  **CD015B1_M70**  **CD015B1_M21**  **CD015B1_M57**  **CD015B1_M72**  **CD015B1_M23**  **CD015B1_M36**  **CD015B1_M58* CD015B1_M73** | **D3S1262**  **D3S1262**  **D3S1262**  **D3S1262**  **D3S1262**  **D3S1262**  **D3S1262**  **D3S1262**  **D3S1262**  **D3S1262**  **D3S1262**  **D3S1262* D3S1262** | **142**  **142**  **142**  **142**  **142**  **142**  **142**  **142**  **142**  **142**  **142**  **142**  **142** |  | **142.0**  **142.0**  **141.9**  **142.0**  **142.0**  **142.0**  **142.0**  **142.0**  **141.9**  **142.0**  **142.0**  **141.9**  **142.0** |  | **1563**  **3039**  **5654**  **3641**  **89**  **629**  **572**  **795**  **1221**  **998**  **4932**  **2479**  **1045** |  | **19658**  **37140**  **72107**  **43727**  **1015**  **7140**  **7278**  **9425**  **14152**  **12280**  **62571**  **32413**  **12667** |  | **3676**  **3691**  **3654**  **3670**  **3683**  **3677**  **3662**  **3690**  **3683**  **3670**  **3669**  **3697**  **3694** |  |
| **1** | **1**  **2**  **3**  **4**  **5**  **6** | **7/13/05**  **7/13/05**  **7/13/05**  **7/13/05**  **7/13/05**  **7/13/05** | **CD015B1_M5**  **CD015B1_M10**  **CD015B1_M12**  **CD015B1_M1**  **CD015B1_M13**  **CD015B1_M14** | **D9S171**  **D9S171**  **D9S171**  **D9S171**  **D9S171**  **D9S171** | **107**  **107**  **107**  **107**  **107**  **107** | **111**  **111**  **111**  **111**  **111**  **111** | **107.5**  **107.4**  **107.5**  **107.4**  **107.4**  **107.5** | **111.2**  **111.3**  **111.4**  **111.3**  **111.2**  **111.3** | **545**  **2893**  **672**  **3048**  **489**  **1944** | **642**  **2082**  **353**  **1776**  **886**  **1623** | **4486**  **25223**  **5669**  **25711**  **3977**  **16411** | **5237**  **17288**  **3013**  **14297**  **8263**  **13362** | **3369**  **3397**  **3411**  **3329**  **3386**  **3400** | **3422**  **3452**  **3467**  **3383**  **3440**  **3454** |
| **2** | **7**  **8**  **9**  **10**  **11**  **12**  **13**  **14**  **15**  **16**  **17**  **18**  **19**  **20**  **21**  **22**  **23**  **24**  **25**  **26**  **27**  **28**  **29**  **30**  **31**  **32**  **33** | **7/14/05**  **7/14/05**  **7/14/05**  **7/14/05**  **7/14/05**  **7/14/05**  **7/14/05**  **7/14/05**  **7/14/05**  **7/14/05**  **7/14/05**  **7/14/05**  **7/14/05**  **7/14/05**  **7/14/05**  **7/14/05**  **7/14/05**  **7/14/05**  **7/14/05**  **7/14/05**  **7/14/05**  **7/14/05**  **7/14/05**  **7/14/05**  **7/14/05**  **7/14/05**  **7/14/05** | **CD015B1_M26**  **CD015B1_M50**  **CD015B1_M28**  **CD015B1_M38**  **CD015B1_M51**  **CD015B1_M64**  **CD015B1_M15**  **CD015B1_M29**  **CD015B1_M39**  **CD015B1_M53**  **CD015B1_M17**  **CD015B1_M30**  **CD015B1_M42**  **CD015B1_M66**  **CD015B1_M31**  **CD015B1_M44**  **CD015B1_M32**  **CD015B1_M46**  **CD015B1_M56**  **CD015B1_M70**  **CD015B1_M21**  **CD015B1_M57**  **CD015B1_M72**  **CD015B1_M23**  **CD015B1_M36**  **CD015B1_M58**  **CD015B1_M73** | **D9S171**  **D9S171**  **D9S171**  **D9S171**  **D9S171**  **D9S171**  **D9S171**  **D9S171**  **D9S171**  **D9S171**  **D9S171**  **D9S171**  **D9S171**  **D9S171**  **D9S171**  **D9S171**  **D9S171**  **D9S171**  **D9S171**  **D9S171**  **D9S171**  **D9S171**  **D9S171**  **D9S171**  **D9S171**  **D9S171**  **D9S171** | **107**  **107**  **107**  **107**  **107**  **107**  **107**  **107**  **107**  **107**  **107**  **107**  **107**  **107**  **107**  **107**  **107**  **107**  **107**  **107**  **107**  **107**  **107**  **107**  **107**  **107**  **107** | **111**  **111**  **111**  **111**  **111**  **111**  **111**  **111**  **111**  **111**  **111**  **111**  **111**  **111**  **111**  **111**  **111**  **111**  **111**  **111**  **111**  **111**  **111**  **111**  **111**  **111**  **111** | **107.3**  **107.3**  **107.2**  **107.3**  **107.3**  **107.3**  **107.3**  **107.4**  **107.3**  **107.3**  **107.4**  **107.3**  **107.4**  **107.3**  **107.3**  **107.3**  **107.3**  **107.3**  **107.4**  **107.3**  **107.3**  **107.3**  **107.3**  **107.3**  **107.3**  **107.3**  **107.3** | **111.1**  **111.2**  **111.1**  **111.0**  **111.1**  **111.2**  **111.1**  **111.1**  **111.1**  **111.1**  **111.2**  **111.2**  **111.1**  **111.2**  **111.1**  **111.1**  **111.1**  **111.1**  **111.1**  **111.1**  **111.1**  **111.1**  **111.1**  **111.1**  **111.1**  **111.2**  **111.1** | **4275**  **3257**  **4531**  **4273**  **2020**  **967**  **2272**  **250**  **2898**  **505**  **816**  **5190**  **834**  **3819**  **3006**  **3541**  **4399**  **3035**  **201**  **883**  **691**  **922**  **1681**  **1670**  **4468**  **2782**  **1767** | **2401**  **1960**  **3475**  **2856**  **1574**  **566**  **2215**  **115**  **2238**  **326**  **1224**  **3820**  **864**  **2758**  **2247**  **2625**  **3623**  **2616**  **204**  **884**  **597**  **759**  **1322**  **2182**  **3612**  **2248**  **1126** | **33633**  **24971**  **35936**  **33194**  **15972**  **7609**  **19270**  **2195**  **23148**  **4113**  **6532**  **41172**  **6636**  **30891**  **24538**  **28898**  **34502**  **24130**  **1618**  **6825**  **5131**  **7335**  **13872**  **12885**  **35854**  **22714**  **14348** | **18774**  **14773**  **26412**  **21249**  **12310**  **5167**  **18458**  **961**  **17668**  **2556**  **10443**  **28759**  **7148**  **21389**  **17940**  **20907**  **27783**  **20319**  **1663**  **6778**  **4491**  **5963**  **10636**  **17999**  **27927**  **17723**  **9129** | **3204**  **3213**  **3184**  **3193**  **3193**  **3217**  **3236**  **3193**  **3202**  **3201**  **3243**  **3201**  **3206**  **3230**  **3224**  **3238**  **3204**  **3219**  **3232**  **3226**  **3213**  **3239**  **3234**  **3220**  **3218**  **3245**  **3242** | **3256**  **3265**  **3236**  **3244**  **3245**  **3269**  **3288**  **3244**  **3253**  **3253**  **3295**  **3253**  **3257**  **3282**  **3276**  **3290**  **3256**  **3271**  **3283**  **3278**  **3265**  **3291**  **3286**  **3271**  **3270**  **3298**  **3294** |
| **1**  **2** | **1**  **2**  **3**  **4**  **5**  **6**  **7** | **6/15/05**  **6/15/05**  **6/15/05**  **6/16/05**  **6/16/05**  **6/16/05**  **6/16/05** | **B_1-3**  **C_1-3**  **C10uL_1-3**  **0.25_10uL_1-2**  **0.5_10uL_1-2**  **0.5_10uL_1-2b**  **0.5_15uL_1-2** | **D17S250**  **D17S250**  **D17S250**  **D17S250**  **D17S250**  **D17S250**  **D17S250** | **195**  **195**  **195**  **195**  **195**  **195**  **195** | **202**  **202**  **202**  **202**  **202**  **202**  **202** | **195.5**  **195.7**  **195.7**  **195.8**  **195.7**  **195.7**  **195.8** | **201.9**  **202.1**  **201.9**  **202.1**  **202.1**  **202.0**  **202.1** | **1161**  **1152**  **461**  **1361**  **1173**  **1269**  **772** | **892**  **947**  **408**  **968**  **931**  **1043**  **673** | **9136**  **9440**  **3588**  **14718**  **12299**  **13503**  **7998** | **6865**  **7639**  **3076**  **10210**  **9925**  **10811**  **7134** | **4286**  **4384**  **4326**  **4945**  **4965**  **4925**  **4931** | **4362**  **4463**  **4403**  **5036**  **5057**  **5015**  **5021** |

| **Day** | **Count** | **Run Date** | **Sample Name** | **Marker** | **Allele 1** | **Allele 2** | **Size 1** | **Size 2** | **Height 1** | **Height 2** | **Peak Area 1** | **Peak Area 2** | **Data Point 1** | **Data Point 2** |
| --- | --- | --- | --- | --- | --- | --- | --- | --- | --- | --- | --- | --- | --- | --- |
| **3** | **8** | **6/20/05** | **0.25uM** | **D17S250** | **195** | **202** | **195.6** | **201.8** | **394** | **297** | **3363** | **2359** | **4269** | **4344** |
| **4**  **5**  **6**  **7**  **8**  **9**  **10** | **9**  **10**  **11**  **12**  **13**  **14**  **15**  **16**  **17**  **18** | **6/20/05**  **6/30/05**  **6/30/05**  **7/5/05**  **7/5/05**  **7/6/05**  **7/7/05**  **7/8/05**  **7/13/05**  **7/14/05** | **CTRL CTRL**  **CTRL6-19-05**  **CTRL CTRL_61905**  **CTRL CRTL CTRL CTRL CTLRL** | **D17S250**  **D17S250**  **D17S250**  **D17S250**  **D17S250**  **D17S250**  **D17S250**  **D17S250**  **D17S250**  **D17S250** | **195**  **195**  **195**  **195**  **195**  **195**  **195**  **195**  **195**  **195** | **202**  **202**  **202**  **202**  **202**  **202**  **202**  **202**  **202**  **202** | **195.6**  **195.5**  **195.4**  **195.5**  **195.5**  **195.5**  **195.5**  **195.5**  **195.7**  **195.6** | **201.8**  **201.7**  **201.7**  **201.7**  **201.8**  **201.8**  **201.8**  **201.8**  **202.1**  **201.8** | **544**  **1399**  **481**  **1027**  **697**  **1044**  **2559**  **1153**  **914**  **645** | **447**  **1070**  **323**  **843**  **483**  **738**  **1733**  **815**  **770**  **549** | **4676**  **11016**  **3949**  **8377**  **5947**  **8984**  **22726**  **9403**  **8508**  **5538** | **3757**  **8528**  **2545**  **6869**  **4004**  **6263**  **14915**  **6427**  **7196**  **4672** | **4293**  **4139**  **4050**  **4154**  **4182**  **4256**  **4249**  **4261**  **4621**  **4367** | **4369**  **4213**  **4124**  **4228**  **4258**  **4333**  **4325**  **4338**  **4703**  **4444** |
| **1**  **2**  **3**  **4** | **1**  **2**  **3**  **4**  **5**  **6**  **7**  **8**  **9**  **10** | **6/15/05**  **6/15/05**  **6/15/05**  **6/16/05**  **6/16/05**  **6/16/05**  **6/16/05**  **6/20/05**  **6/20/05**  **6/30/05** | **B_1-3**  **C_1-3**  **C10uL_1-3**  **0.25_10uL_1-2**  **0.5_10uL_1-2**  **0.5_10uL_1-2b**  **0.5_15uL_1-2**  **0.25uM CTRL CTRL** | **D18S61**  **D18S61**  **D18S61**  **D18S61**  **D18S61**  **D18S61**  **D18S61**  **D18S61**  **D18S61**  **D18S61** | **154**  **154**  **154**  **154**  **154**  **154**  **154**  **154**  **154**  **154** |  | **153.9**  **154.0**  **154.0**  **154.1**  **154.0**  **153.9**  **154.0**  **154.0**  **154.0**  **153.9** |  | **5334**  **7163**  **2490**  **7094**  **7369**  **6916**  **7169**  **976**  **645**  **1598** |  | **47180**  **71006**  **25397**  **71893**  **105395**  **103191**  **85540**  **8667**  **5107**  **13018** |  | **3774**  **3850**  **3798**  **4333**  **4350**  **4313**  **4320**  **3751**  **3775**  **3636** |  |
| **5**  **6**  **7**  **8**  **9**  **10** | **11**  **12**  **13**  **14**  **15**  **16**  **17**  **18** | **6/30/05**  **7/5/05**  **7/5/05**  **7/6/05**  **7/7/05**  **7/8/05**  **7/13/05**  **7/14/05** | **CTRL6-19-05**  **CTRL CTRL_61905**  **CTRL CRTL CTRL CTRL CTLRL** | **D18S61**  **D18S61**  **D18S61**  **D18S61**  **D18S61**  **D18S61**  **D18S61**  **D18S61** | **154**  **154**  **154**  **154**  **154**  **154**  **154**  **154** |  | **153.9**  **153.9**  **153.8**  **153.8**  **153.8**  **154.0**  **154.1**  **153.9** |  | **1227**  **450**  **1933**  **1961**  **4494**  **4298**  **6251**  **4361** |  | **10377**  **3420**  **15951**  **15979**  **40369**  **36536**  **59854**  **38240** |  | **3554**  **3644**  **3670**  **3736**  **3729**  **3744**  **4068**  **3838** |  |
| **1**  **2**  **3**  **4**  **5**  **6**  **7**  **8**  **9**  **10** | **1**  **2**  **3**  **4**  **5**  **6**  **7**  **8**  **9**  **10**  **11**  **12**  **13**  **14**  **15**  **16**  **17**  **18** | **6/15/05**  **6/15/05**  **6/15/05**  **6/16/05**  **6/16/05**  **6/16/05**  **6/16/05**  **6/20/05**  **6/20/05**  **6/30/05**  **6/30/05**  **7/5/05**  **7/5/05**  **7/6/05**  **7/7/05**  **7/8/05**  **7/13/05**  **7/14/05** | **B_1-3**  **C_1-3**  **C10uL_1-3**  **0.25_10uL_1-2**  **0.5_10uL_1-2**  **0.5_10uL_1-2b**  **0.5_15uL_1-2**  **0.25uM CTRL CTRL**  **CTRL6-19-05**  **CTRL CTRL_61905**  **CTRL CRTL CTRL CTRL CTLRL** | **D2S123**  **D2S123**  **D2S123**  **D2S123**  **D2S123**  **D2S123**  **D2S123**  **D2S123**  **D2S123**  **D2S123**  **D2S123**  **D2S123**  **D2S123**  **D2S123**  **D2S123**  **D2S123**  **D2S123**  **D2S123** | **219**  **219**  **219**  **219**  **219**  **219**  **219**  **219**  **219**  **219**  **219**  **219**  **219**  **219**  **219**  **219**  **219**  **219** |  | **218.5**  **218.8**  **218.7**  **218.9**  **218.9**  **218.8**  **218.8**  **218.4**  **218.6**  **218.3**  **218.2**  **218.4**  **218.5**  **218.5**  **218.5**  **218.4**  **218.9**  **218.5** |  | **857**  **3378**  **1720**  **3926**  **2779**  **4023**  **2134**  **470**  **487**  **1835**  **578**  **491**  **753**  **1307**  **3013**  **1645**  **1238**  **802** |  | **6875**  **30504**  **15426**  **41715**  **29096**  **43296**  **22167**  **3660**  **3938**  **14966**  **4470**  **4340**  **6440**  **12152**  **29054**  **14772**  **12563**  **7531** |  | **4548**  **4656**  **4594**  **5262**  **5284**  **5241**  **5245**  **4533**  **4560**  **4397**  **4305**  **4415**  **4445**  **4524**  **4516**  **4527**  **4906**  **4637** |  |
| **1**  **2** | **1**  **2**  **3**  **4**  **5**  **6**  **7** | **6/15/05**  **6/15/05**  **6/15/05**  **6/16/05**  **6/16/05**  **6/16/05**  **6/16/05** | **B_1-3**  **C_1-3**  **C10uL_1-3**  **0.25_10uL_1-2**  **0.5_10uL_1-2**  **0.5_10uL_1-2b**  **0.5_15uL_1-2** | **D3S1262**  **D3S1262**  **D3S1262**  **D3S1262**  **D3S1262**  **D3S1262**  **D3S1262** | **136**  **136**  **136**  **136**  **136**  **136**  **136** | **142**  **142**  **142**  **142**  **142**  **142**  **142** | **135.8**  **135.9**  **135.7**  **135.8**  **135.8**  **135.8**  **135.8** | **142.0**  **142.0**  **142.0**  **142.1**  **142.0**  **142.1**  **142.0** | **881**  **800**  **541**  **1889**  **1302**  **1880**  **980** | **666**  **624**  **379**  **1340**  **1028**  **1391**  **727** | **8898**  **8193**  **5569**  **26047**  **17911**  **27314**  **12845** | **6554**  **6472**  **4065**  **18868**  **14040**  **19925**  **9451** | **3563**  **3632**  **3582**  **4083**  **4099**  **4065**  **4073** | **3637**  **3708**  **3658**  **4171**  **4187**  **4153**  **4159** |

| **Day** | **Count** | **Run Date** | **Sample Name** | **Marker** | **Allele 1** | **Allele 2** | **Size 1** | **Size 2** | **Height 1** | **Height 2** | **Peak Area 1** | **Peak Area 2** | **Data Point 1** | **Data Point 2** |
| --- | --- | --- | --- | --- | --- | --- | --- | --- | --- | --- | --- | --- | --- | --- |
| **3**  **4**  **5**  **6**  **7**  **8**  **9**  **10** | **8**  **9**  **10**  **11**  **12**  **13**  **14**  **15**  **16**  **17**  **18** | **6/20/05**  **6/20/05**  **6/30/05**  **6/30/05**  **7/5/05**  **7/5/05**  **7/6/05**  **7/7/05**  **7/8/05**  **7/13/05**  **7/14/05** | **0.25uM CTRL CTRL**  **CTRL6-19-05**  **CTRL CTRL_61905**  **CTRL CRTL CTRL CTRL CTLRL** | **D3S1262**  **D3S1262**  **D3S1262**  **D3S1262**  **D3S1262**  **D3S1262**  **D3S1262**  **D3S1262**  **D3S1262**  **D3S1262**  **D3S1262** | **136**  **136**  **136**  **136**  **136**  **136**  **136**  **136**  **136**  **136**  **136** | **142**  **142**  **142**  **142**  **142**  **142**  **142**  **142**  **142**  **142**  **142** | **135.9**  **135.9**  **135.8**  **135.7**  **135.6**  **135.6**  **135.6**  **135.6**  **135.9**  **135.9**  **135.8** | **142.1**  **142.1**  **142.0**  **142.0**  **141.9**  **141.8**  **141.9**  **141.9**  **142.1**  **142.2**  **142.1** | **887**  **1092**  **1360**  **1110**  **647**  **1532**  **954**  **1854**  **1591**  **955**  **728** | **689**  **869**  **1095**  **899**  **504**  **1193**  **744**  **1366**  **1360**  **850**  **642** | **9195**  **11154**  **13689**  **10854**  **5690**  **15337**  **9919**  **23933**  **17461**  **11781**  **7681** | **7122**  **9103**  **11124**  **8516**  **4437**  **11912**  **7535**  **17839**  **15172**  **10444**  **6893** | **3538**  **3561**  **3428**  **3349**  **3434**  **3459**  **3522**  **3515**  **3531**  **3840**  **3621** | **3613**  **3636**  **3501**  **3421**  **3508**  **3533**  **3597**  **3590**  **3605**  **3920**  **3698** |
| **1** | **1**  **2**  **3** | **6/15/05**  **6/15/05**  **6/15/05** | **B_1-3**  **C_1-3**  **C10uL_1-3** | **D9S171**  **D9S171**  **D9S171** | **113**  **113**  **113** |  | **113.1**  **113.2**  **113.2** |  | **1043**  **3721**  **2512** |  | **8493**  **29591**  **20177** |  | **3272**  **3333**  **3288** |  |
| **2**  **3**  **4** | **4**  **5**  **6**  **7**  **8**  **9**  **10**  **11** | **6/16/05**  **6/16/05**  **6/16/05**  **6/16/05**  **6/20/05**  **6/20/05**  **6/30/05**  **6/30/05** | **0.25_10uL_1-2**  **0.5_10uL_1-2**  **0.5_10uL_1-2b**  **0.5_15uL_1-2**  **0.25uM CTRL CTRL**  **CTRL6-19-05** | **D9S171**  **D9S171**  **D9S171**  **D9S171**  **D9S171**  **D9S171**  **D9S171**  **D9S171** | **113**  **113**  **113**  **113**  **113**  **113**  **113**  **113** |  | **113.2**  **113.2**  **113.2**  **113.2**  **113.1**  **113.2**  **113.0**  **112.9** |  | **3288**  **1882**  **2534**  **1298**  **727**  **1254**  **2364**  **859** |  | **33567**  **18846**  **26283**  **13107**  **6757**  **11121**  **19779**  **8023** |  | **3742**  **3757**  **3726**  **3733**  **3245**  **3267**  **3142**  **3067** |  |
| **5**  **6**  **7**  **8**  **9**  **10** | **12**  **13**  **14**  **15**  **16**  **17**  **18** | **7/5/05**  **7/5/05**  **7/6/05**  **7/7/05**  **7/8/05**  **7/13/05**  **7/14/05** | **CTRL CTRL_61905**  **CTRL CRTL CTRL CTRL CTLRL** | **D9S171**  **D9S171**  **D9S171**  **D9S171**  **D9S171**  **D9S171**  **D9S171** | **113**  **113**  **113**  **113**  **113**  **113**  **113** |  | **112.9**  **112.9**  **113.0**  **112.9**  **113.2**  **113.3**  **113.0** |  | **559**  **1200**  **991**  **2470**  **4085**  **1826**  **1309** |  | **4560**  **11337**  **8841**  **21982**  **32862**  **16493**  **11242** |  | **3146**  **3169**  **3228**  **3221**  **3238**  **3529**  **3321** |  |
| **1**  **2**  **3** | **1**  **2**  **3**  **4**  **5**  **6** | **5/31/05**  **5/31/05**  **5/31/05**  **5/31/05**  **6/6/05**  **7/14/05** | **G2MF10_.5**  **G2MF15_.5**  **G2MF7_.5**  **G2MF7_1**  **G2MF10**  **MF** | **D17S250**  **D17S250**  **D17S250**  **D17S250**  **D17S250**  **D17S250** | **189**  **189**  **189**  **189**  **189**  **189** | **193**  **193**  **193**  **193**  **193**  **193** | **189.5**  **189.6**  **189.6**  **189.6**  **189.2**  **189.3** | **193.6**  **193.7**  **193.7**  **193.6**  **193.3**  **193.5** | **197**  **349**  **223**  **546**  **386**  **1790** | **162**  **259**  **172**  **436**  **289**  **1452** | **1607**  **2890**  **1783**  **4372**  **2985**  **14969** | **1230**  **2100**  **1417**  **3581**  **2241**  **11902** | **4336**  **4347**  **4341**  **4332**  **4074**  **4269** | **4389**  **4400**  **4394**  **4384**  **4124**  **4322** |
| **1**  **2**  **3** | **1**  **2**  **3**  **4**  **5**  **6** | **5/31/05**  **5/31/05**  **5/31/05**  **5/31/05**  **6/6/05**  **7/14/05** | **G2MF10_.5**  **G2MF15_.5**  **G2MF7_.5**  **G2MF7_1**  **G2MF10**  **MF** | **D18S61**  **D18S61**  **D18S61**  **D18S61**  **D18S61**  **D18S61** | **168**  **168**  **168**  **168**  **168**  **168** |  | **168.0**  **168.1**  **168.1**  **168.1**  **167.7**  **167.8** |  | **157**  **220**  **167**  **492**  **177**  **4583** |  | **1281**  **1741**  **1325**  **3763**  **1334**  **41796** |  | **4060**  **4069**  **4065**  **4056**  **3812**  **3996** |  |
| **1**  **2**  **3** | **1**  **2**  **3**  **4**  **5**  **6** | **5/31/05**  **5/31/05**  **5/31/05**  **5/31/05**  **6/6/05**  **7/14/05** | **G2MF10_.5**  **G2MF15_.5**  **G2MF7_.5**  **G2MF7_1**  **G2MF10**  **MF** | **D2S123**  **D2S123**  **D2S123**  **D2S123**  **D2S123**  **D2S123** | **219**  **219**  **219**  **219**  **219**  **219** |  | **218.7**  **218.7**  **218.8**  **218.7**  **218.3**  **218.5** |  | **1085**  **1353**  **999**  **229**  **908**  **2044** |  | **9569**  **11804**  **8485**  **1886**  **7341**  **19339** |  | **4688**  **4700**  **4694**  **4684**  **4408**  **4617** |  |
| **1**  **2**  **3** | **1**  **2**  **3**  **4**  **5**  **6** | **5/31/05**  **5/31/05**  **5/31/05**  **5/31/05**  **6/6/05**  **7/14/05** | **G2MF10_.5**  **G2MF15_.5**  **G2MF7_.5**  **G2MF7_1**  **G2MF10* MF** | **D3S1262**  **D3S1262**  **D3S1262**  **D3S1262**  **D3S1262* D3S1262** | **136**  **136**  **136**  **136**  **132**  **136** | **136** | **135.9**  **135.9**  **136.0**  **135.9**  **131.7**  **135.8** | **135.7** | **839**  **1165**  **858**  **1487**  **601**  **4123** | **1191** | **7937**  **10561**  **7881**  **13898**  **5143**  **51534** | **10713** | **3665**  **3672**  **3668**  **3661**  **3388**  **3605** | **3437** |

| **Day** | **Count** | **Run Date** | **Sample Name** | **Marker** | **Allele 1** | **Allele 2** | **Size 1** | **Size 2** | **Height 1** | **Height 2** | **Peak Area 1** | **Peak Area 2** | **Data Point 1** | **Data Point 2** |
| --- | --- | --- | --- | --- | --- | --- | --- | --- | --- | --- | --- | --- | --- | --- |
| **1**  **2**  **3** | **1**  **2**  **3**  **4**  **5**  **6** | **5/31/05**  **5/31/05**  **5/31/05**  **5/31/05**  **6/6/05**  **7/14/05** | **G2MF10_.5**  **G2MF15_.5**  **G2MF7_.5**  **G2MF7_1**  **G2MF10**  **MF** | **D9S171**  **D9S171**  **D9S171**  **D9S171**  **D9S171**  **D9S171** | **113**  **113**  **113**  **113**  **113**  **113** |  | **113.2**  **113.2**  **113.3**  **113.2**  **113.0**  **113.1** |  | **1171**  **1631**  **1132**  **2095**  **2738**  **2914** |  | **9759**  **13773**  **9454**  **17474**  **21897**  **24313** |  | **3365**  **3371**  **3367**  **3360**  **3151**  **3308** |  |
| **1** | **1**  **1**  **1**  **1**  **1**  **1**  **1**  **1**  **1**  **1** | **06-06-05-GS**  **06-06-05-GS**  **06-06-05-GS**  **06-06-05-GS**  **06-06-05-GS**  **06-06-05-GS**  **06-06-05-GS**  **06-06-05-GS**  **06-06-05-GS**  **06-06-05-GS** | **G2BF53M3**  **G2BF53M4**  **G2BF53M3**  **G2BF53M4**  **G2BF53M3**  **G2BF53M4**  **G2BF53M3**  **G2BF53M4**  **G2BF53M3**  **G2BF53M4** | **D17S250**  **D17S250**  **D18S61**  **D18S61**  **D2S123**  **D2S123**  **D3S1262**  **D3S1262**  **D9S171**  **D9S171** | **191**  **191**  **154**  **154**  **216**  **216**  **136**  **136**  **111**  **111** | **193**  **193**  **170**  **170**  **219(1)**  **219(1)**  **138**  **138**  **115**  **115** | **191.6**  **191.3**  **154.0**  **153.9**  **216.6**  **216.3**  **135.8**  **135.7**  **111.3**  **111.1** | **193.7**  **193.4**  **170.0**  **169.9**  **218.9**  **218.5**  **137.8**  **137.7**  **115.1**  **114.9** | **1112**  **1108**  **375**  **230**  **2447**  **2070**  **811**  **893**  **3851**  **4635** | **740**  **792**  **233**  **125**  **1791**  **1391**  **419**  **520**  **2388**  **3654** | **9918**  **8799**  **3233**  **1959**  **22901**  **18083**  **10603**  **10186**  **32777**  **37150** | **6210**  **6202**  **1708**  **894**  **15771**  **11339**  **5149**  **5809**  **18505**  **27899** | **4437**  **4196**  **3948**  **3733**  **4743**  **4486**  **3725**  **3520**  **3393**  **3203** | **4464**  **4221**  **4154**  **3929**  **4770**  **4511**  **3751**  **3545**  **3447**  **3254** |
|  | **1**  **1**  **1**  **1**  **1**  **1**  **1**  **1** | **06-06-05-GS**  **06-06-05-GS**  **06-06-05-GS**  **06-06-05-GS**  **06-06-05-GS**  **06-06-05-GS**  **06-06-05-GS**  **06-06-05-GS** | **G2CS72M2**  **G2CS72M6**  **G2CS72M2**  **G2CS72M6**  **G2CS72M2**  **G2CS72M6**  **G2CS72M2**  **G2CS72M6** | **D17S250**  **D17S250**  **D18S61**  **D18S61**  **D2S123**  **D2S123**  **D3S1262**  **D3S1262** | **191**  **193**  **170**  **166**  **219(1)**  **214**  **132**  **138** | **195**  **197**  **168**  **219(1)**  **136**  **144** | **191.4**  **193.4**  **169.8**  **165.8**  **218.4**  **214.0**  **131.8**  **137.6** | **195.5**  **197.5**  **167.9**  **218.4**  **135.7**  **144.0** | **718**  **1700**  **1645**  **562**  **2127**  **2309**  **516**  **1088** | **484**  **1341**  **317**  **1724**  **641**  **902** | **5800**  **13658**  **14161**  **4756**  **20343**  **21396**  **6157**  **13807** | **3630**  **10326**  **2325**  **14865**  **7925**  **11047** | **4155**  **4242**  **3888**  **3896**  **4467**  **4483**  **3435**  **3560** | **4205**  **4293**  **3922**  **4533**  **3483**  **3635** |
|  | **1**  **1** | **06-06-05-GS**  **06-06-05-GS** | **G2CS72M2**  **G2CS72M6** | **D9S171**  **D9S171** | **115**  **107** | **119**  **119** | **114.9**  **107.2** | **118.8**  **118.7** | **2595**  **6473** | **2011**  **3959** | **19998**  **62622** | **15215**  **31414** | **3219**  **3163** | **3270**  **3318** |
|  | **1**  **1** | **06-06-05-GS**  **06-06-05-GS** | **G2BF113WT2**  **G2BF113M1** | **D17S250**  **D17S250** | **195**  **195** | **207**  **207** | **195.5**  **195.5** | **206.1**  **206.2** | **483**  **1085** | **340**  **795** | **4136**  **8986** | **2992**  **6512** | **4232**  **4290** | **4357**  **4418** |
|  | **1**  **1**  **1**  **1**  **1**  **1**  **1**  **1**  **1**  **1**  **1**  **1**  **1** | **06-06-05-GS**  **06-06-05-GS**  **06-06-05-GS**  **06-06-05-GS**  **06-06-05-GS**  **06-06-05-GS**  **06-06-05-GS**  **06-06-05-GS**  **06-06-05-GS**  **06-06-05-GS**  **06-06-05-GS**  **06-06-05-GS**  **06-06-05-GS** | **G2BF113M4**  **G2BF113WT2**  **G2BF113M1**  **G2BF113M4**  **G2BF113WT2**  **G2BF113M1**  **G2BF113M4**  **G2BF113WT2**  **G2BF113M1**  **G2BF113M4**  **G2BF113WT2**  **G2BF113M1**  **G2BF113M4** | **D17S250**  **D18S61**  **D18S61**  **D18S61**  **D2S123**  **D2S123**  **D2S123**  **D3S1262**  **D3S1262**  **D3S1262**  **D9S171**  **D9S171**  **D9S171** | **197**  **172**  **172**  **168**  **216**  **216**  **219(1)**  **132**  **132**  **136**  **113**  **113**  **113** | **204**  **219(1)**  **219(1)**  **236**  **140**  **140**  **119**  **119**  **121** | **197.5**  **171.7**  **171.7**  **167.7**  **216.1**  **216.2**  **218.4**  **131.8**  **131.8**  **135.7**  **113.0**  **113.1**  **112.9** | **204.0**  **218.4**  **218.4**  **235.9**  **139.7**  **139.7**  **118.8**  **118.8**  **120.7** | **970**  **1617**  **3232**  **427**  **1711**  **2911**  **1769**  **658**  **1330**  **2643**  **3600**  **6484**  **6127** | **747**  **1093**  **1874**  **1452**  **691**  **1368**  **2404**  **5265**  **4357** | **8118**  **13934**  **29008**  **3585**  **15179**  **26028**  **16955**  **8101**  **18124**  **33442**  **29897**  **61633**  **49902** | **5933**  **9300**  **15366**  **13384**  **8146**  **16666**  **19206**  **42907**  **34470** | **4263**  **3937**  **3991**  **3894**  **4470**  **4532**  **4501**  **3457**  **3504**  **3512**  **3215**  **3260**  **3221** | **4339**  **4495**  **4557**  **4697**  **3555**  **3603**  **3291**  **3336**  **3323** |
|  | **1**  **1**  **1**  **1**  **1**  **1**  **1**  **1**  **1**  **1** | **06-06-05-GS**  **06-06-05-GS**  **06-06-05-GS**  **06-06-05-GS**  **06-06-05-GS**  **06-06-05-GS**  **06-06-05-GS**  **06-06-05-GS**  **06-06-05-GS**  **06-06-05-GS** | **G2CS143M3**  **G2CS143M6**  **G2CS143M3**  **G2CS143M6**  **G2CS143M3**  **G2CS143M6**  **G2CS143M3**  **G2CS143M6**  **G2CS143M3**  **G2CS143M6** | **D17S250**  **D17S250**  **D18S61**  **D18S61**  **D2S123**  **D2S123**  **D3S1262**  **D3S1262**  **D9S171**  **D9S171** | **189**  **191**  **168**  **168**  **219(1)**  **219(1)**  **132**  **134**  **107**  **105** | **193**  **193**  **174**  **172**  **223**  **138**  **113** | **189.3**  **191.4**  **167.8**  **167.8**  **218.3**  **218.3**  **131.6**  **133.6**  **107.4**  **105.1** | **193.4**  **193.3**  **173.7**  **171.6**  **222.7**  **137.7**  **112.9** | **1384**  **2019**  **286**  **450**  **2041**  **4147**  **3886**  **828**  **6580**  **6104** | **1070**  **1299**  **270**  **290**  **1632**  **703**  **6537** | **10944**  **16113**  **2219**  **3657**  **17825**  **38350**  **46801**  **10444**  **63820**  **68435** | **8376**  **10055**  **2271**  **2267**  **13562**  **8440**  **53190** | **4101**  **4189**  **3837**  **3895**  **4436**  **4503**  **3409**  **3486**  **3097**  **3114** | **4151**  **4213**  **3909**  **3943**  **4485**  **3537**  **3170** |
|  |  |  |  |  |  |  |  |  |  |  |  |  |  |  |

| **Sample Type** | **# of Patients** | **# of Markers** | **# of Alleles** |
| --- | --- | --- | --- |
| **UC** | **7** | **85** | **850** |
| **CD** | **6** | **710** | **7100** |
| **Control Sample** | **1** | **90** | **900** |
| **Cell Line** | **1** | **30** | **300** |
| **Other Samples** | **4** | **45** | **450** |
| **TOTAL** | **19** | **960** | **9600** |
