# Supplementary material for: Development of a cost-effective high-throughput process of microsatellite analysis involving miniaturized multiplexed PCR amplification and automated allele identification
Source: Hum Genomics. 2013 Mar 5;7(1):6. doi: 10.1186/1479-7364-7-6 (PMC3600708; doi:10.1186/1479-7364-7-6)
Supplement: Additional file 2 — Process reproducibility with group I markers. Summary of sizing values of identified alleles for each marker from the amplification of control samples in 23 independent runs over the course of 5 months. Averages, standard deviations, and %CVs have been calculated for each allele. Process variation includes sample preparation, PCR amplification, fragment separation, peak detection, and fragment sizing. [file 1479-7364-7-6-S2.doc]

**Additional file 2. Process reproducibility with group I markers**

**Allele 1**

**Allele 2**

**Allele 1**

**Allele 2**

**Allele 1**

**Allele 2**

**Allele 1**

**Allele 2**

**Allele 1**

**Allele 2**

**Allele 1**

**Allele 2**

**Bins**

**127**

**128**

**122**

**219**

**225**

**92**

**94**

**176**

**182**

**187**

**193**

**Run Date**

1

1

**5/31/2005**

127.1

122.0

219.1

225.4

91.9

94.2

176.5

182.3

187.6

193.4

2

2

6/6/2005

126.7

121.8

218.3

224.6

91.6

93.8

176.0

181.8

187.1

192.9

3

6/6/2005

126.6

121.6

218.2

224.7

91.6

93.8

176.0

181.8

187.1

192.7

3

4

6/8/2005

126.8

121.7

218.8

225.0

91.8

94.0

176.3

182.0

187.3

193.2

5

6/8/2005

126.9

121.8

218.9

225.1

91.8

94.0

176.3

182.1

187.4

193.2

4

6

6/15/2005

127.0

128.0

122.0

219.1

225.4

92.0

94.1

176.4

182.3

187.6

193.4

7

6/15/2005

126.9

128.0

121.9

219.0

225.3

91.9

94.1

176.4

182.2

187.4

193.3

5

8

6/16/2005

127.0

128.0

121.9

219.1

225.4

91.9

94.1

176.5

182.3

187.6

193.3

9

6/16/2005

126.9

127.9

121.8

218.7

224.9

91.7

94.0

176.2

182.1

187.4

193.2

10

6/16/2005

127.1

128.1

122.0

218.9

225.2

91.8

94.0

176.4

182.2

187.6

193.3

6

11

6/20/2005

126.9

121.8

218.5

224.8

91.7

93.9

176.2

182.0

187.3

193.0

7

12

6/30/2005

126.7

121.6

218.4

224.6

91.6

93.8

176.0

181.8

187.0

192.8

13

6/30/2005

126.7

121.6

218.4

224.6

91.7

93.9

176.1

181.9

187.2

192.9

8

14

7/6/2005

126.8

121.6

218.6

224.8

91.6

93.9

176.1

181.9

187.2

192.9

9

15

7/7/2005

126.7

121.7

218.5

224.8

91.6

93.8

176.1

181.9

187.2

192.9

16

7/7/2005

126.7

121.6

218.6

224.8

91.6

93.8

176.0

181.9

187.2

193.0

10

17

7/8/2005

126.8

121.7

218.4

224.6

91.7

93.9

176.1

181.8

187.2

192.9

18

7/8/2005

126.8

121.8

218.4

224.6

91.6

93.9

176.1

181.9

187.2

192.9

19

7/8/2005

127.0

121.8

218.7

224.9

91.8

94.0

176.3

182.1

187.4

193.1

11

20

7/11/2005

126.8

121.8

218.6

224.8

91.7

93.9

176.1

182.0

187.2

193.0

12

21

7/13/2005

126.9

121.9

218.9

225.1

91.8

94.1

176.3

182.1

187.5

193.2

13

22

7/15/2005

126.9

121.8

218.5

224.7

91.7

93.9

176.0

181.8

187.2

192.9

14

23

**11/14/2005**

126.8

121.6

218.5

224.8

91.6

93.8

176.1

181.9

187.2

192.9

**Average**

**126.8**

**128.0**

**121.8**

**218.6**

**224.9**

**91.7**

**93.9**

**176.2**

**182.0**

**187.3**

**193.0**

**SD**

**0.12**

**0.06**

**0.12**

**0.27**

**0.27**

**0.11**

**0.12**

**0.17**

**0.17**

**0.18**

**0.19**

**%CV**

**0.09%**

**0.05%**

**0.10%**

**0.12%**

**0.12%**

**0.12%**

**0.12%**

**0.09%**

**0.10%**

**0.10%**

**0.10%**

**Run**

**Count**

**Day**

**Count**

**Sizing Values**

**Microsatellite Markers**

**Bat25**

**Bat26**

**D3S3623**

**D5S346**

**D6S262**

**D7S481**
