# Supplementary material for: Development of a cost-effective high-throughput process of microsatellite analysis involving miniaturized multiplexed PCR amplification and automated allele identification
Source: Hum Genomics. 2013 Mar 5;7(1):6. doi: 10.1186/1479-7364-7-6 (PMC3600708; doi:10.1186/1479-7364-7-6)
Supplement: Additional file 3 — Process reproducibility with group II markers. Summary of sizing values of identified alleles for each marker from the amplification of control samples in 18 independent runs over the course of 1 month. Averages, standard deviations, and %CVs have been calculated for each allele. Process variation include sample preparation, PCR amplification, fragment separation, peak detection, and fragment sizing. [file 1479-7364-7-6-S3.doc]

**Additional file 3. Process reproducibility with group II markers**

**Allele 1**

**Allele 2**

**Allele 1**

**Allele 2**

**Allele 1**

**Allele 2**

**Allele 1**

**Allele 2**

**Allele 1**

**Allele 2**

**Bins**

**195**

**202**

**154**

**219**

**136**

**142**

**113**

**Run Date**

1

1

**6/15/2005**

195.5

201.9

153.9

218.5

135.8

142.0

113.1

2

6/15/2005

195.7

202.1

154.0

218.8

135.9

142.0

113.2

3

6/15/2005

195.7

201.9

154.0

218.7

135.7

142.0

113.2

2

4

6/16/2005

195.8

202.1

154.1

218.9

135.8

142.1

113.2

5

6/16/2005

195.7

202.1

154.0

218.9

135.8

142.0

113.2

6

6/16/2005

195.7

202.0

153.9

218.8

135.8

142.1

113.2

7

6/16/2005

195.8

202.1

154.0

218.8

135.8

142.0

113.2

3

8

6/20/2005

195.6

201.8

154.0

218.4

135.9

142.1

113.1

9

6/20/2005

195.6

201.8

154.0

218.6

135.9

142.1

113.2

4

10

6/30/2005

195.5

201.7

153.9

218.3

135.8

142.0

113.0

11

6/30/2005

195.4

201.7

153.9

218.2

135.7

142.0

112.9

5

12

7/5/2005

195.5

201.7

153.9

218.4

135.6

141.9

112.9

13

7/5/2005

195.5

201.8

153.8

218.5

135.6

141.8

112.9

6

14

7/6/2005

195.5

201.8

153.8

218.5

135.6

141.9

113.0

7

15

7/7/2005

195.5

201.8

153.8

218.5

135.6

141.9

112.9

8

16

7/8/2005

195.5

201.8

154.0

218.4

135.9

142.1

113.2

9

17

7/13/2005

195.7

202.1

154.1

218.9

135.9

142.2

113.3

10

18

**7/14/2005**

195.6

201.8

153.9

218.5

135.8

142.1

113.0

**Average**

**195.6**

**201.9**

**153.9**

**218.6**

**135.8**

**142.0**

**113.1**

**SD**

**0.12**

**0.15**

**0.11**

**0.22**

**0.10**

**0.10**

**0.12**

**%CV**

**0.06%**

**0.07%**

**0.07%**

**0.10%**

**0.08%**

**0.07%**

**0.11%**

**Run**

**Count**

**Day**

**Count**

**Sizing Values**

**Microsatellite Markers**

**D17S250**

**D18S61**

**D2S123**

**D3S1262**

**D9S171**
