# Supplementary material for: Development of a cost-effective high-throughput process of microsatellite analysis involving miniaturized multiplexed PCR amplification and automated allele identification
Source: Hum Genomics. 2013 Mar 5;7(1):6. doi: 10.1186/1479-7364-7-6 (PMC3600708; doi:10.1186/1479-7364-7-6)
Supplement: Additional file 4 — Process accuracy with group I markers. Summary of results from automated allele identification of 1,920 dinucleotide markers and 960 mononucleotide markers from 239 T cell clones from different subjects organized by dates of processing. [file 1479-7364-7-6-S4.doc]

**Additional file 4. Process accuracy with group I markers**

**Allele 1**

**Allele 2**

**Allele 1**

**Allele 2**

**Allele 1**

**Allele 2**

**Allele 1**

**Allele 2**

**Allele 1**

**Allele 2**

**Allele 1**

**Allele 2**

UC002B1

6/30/2005

4

128

122

219

225

103

105

182

201

32

m19

1

127

128

122

219

225

103

105

182

201

8

UC005B1

6/30/2005

2

126

127

123

225

227

92

103

172

182

199

203

16

m3

1

127

123

225

227

92

103

172

182

199

203

8

UC006B1

6/30/2005

3

127

122

219

223

92

105

179

184

193

201

24

UC009B1

6/30/2005

3

127

123

219

221

94

170

172

203

24

**Subtotal Count**

**6/30/2005**

**14**

**112**

**0.00%**

CD003B1_2

7/5/2005

32

127

122

219

92

105

182

185

197

201

256

m9

1

127

128

122

219

92

105

182

185

197

201

8

m5, m8

2

126

127

122

219

92

105

182

185

197

201

16

m40

1

127

121

122

219

92

105

182

185

197

201

8

**Subtotal Count**

**7/5/2005**

**36**

**288**

**0.00%**

CD006B1

7/6/2005

32

127

122

219

92

94

182

184

187

197

256

6

126

127

122

219

92

94

182

184

187

197

48

m15, m20

2

127

121

122

219

92

94

182

184

187

197

16

m25

1

127

122

219

*

92

94

182

184

187

197

8

**Subtotal Count**

**7/6/2005**

**41**

**328**

**0.30%**

CD006B1

7/7/2005

1

127

122

219

92

94

182

184

187

197

CD009B1

7/7/2005

5

127

123

219

221

94

170

172

203

40

m31

1

127

122

219

221

94

170

172

203

8

m51

1

127

128

123

219

221

94

170

172

203

8

11

127

122

123

219

221

94

170

172

203

88

CD010B1_2

7/7/2005

2

126

127

121

219

225

89

92

172

176

187

199

16

m18, m1

2

126

127

121

219

225

*

92

172

176

187

199

16

m15

1

126

127

121

219

225

89

92

*

176

187

199

8

**Subtotal Count**

**7/7/2005**

**24**

**192**

**1.04%**

CD013B1

7/8/2005

1

128

122

219

223

103

107

172

182

199

8

m5_1ul

1

128

122

219

*

*

107

172

182

199

8

2

128

129

122

219

223

103

107

172

182

199

16

m10_0.4ul

extra sample

128

129

122

*

223

103

107

172

182

199

8

3

129

122

219

103

107

172

182

199

24

**Subtotal Count**

**7/8/2005**

**8**

**64**

**4.69%**

**D5S346**

**D6S262**

**D7S481**

**# of Alleles with**

**Dinucleotide Repeats**

**Analyzed**

**% Deviation**

**from Expected**

**Subject**

**Run Date**

**# of**

**Clone(s)**

**Microsatellite Markers**

**Bat25**

**Bat26**

**D3S3623**

**Allele 1**

**Allele 2**

**Allele 1**

**Allele 2**

**Allele 1**

**Allele 2**

**Allele 1**

**Allele 2**

**Allele 1**

**Allele 2**

**Allele 1**

**Allele 2**

CD015B1

7/11/2005

8

126

122

221

92

101

184

186

187

197

64

m13

1

126

122

221

92

101

184

*

187

197

8

**Subtotal Count**

**7/11/2005**

**9**

**72**

**1.39%**

CD015B1

7/13/2005

24

126

122

221

92

101

184

186

187

197

192

4

126

127

122

221

92

101

184

186

187

197

32

m48

1

126

127

122

221

92*

101*

184

186

187

*

8

m50

1

126

127

122

123

221

92

101

184

186

187

197

8

m53, m56

2

127

122

221

92

101

184

186

187

197

16

CD009B1

7/13/2005

4

127

123

219

221

94

170

172

203

32

CD013B1

7/13/2005

1

128

129

122

219

103

107

172

182

199

8

**Subtotal Count**

**7/13/2005**

**37**

**296**

**1.01%**

CD003B1_2

7/15/2005

2

127

122

219

92

105

182

185

197

201

CD009B1

7/15/2005

6

127

123

219

221

94

170

172

203

48

m3, m44

2

127

122

123

219

221

94

170

172

203

16

m2

1

127

122

219

221

94

170

172

203

8

CD010B1_2

7/15/2005

5

127

121

219

225

89

92

172

176

187

199

40

**Subtotal Count**

**7/15/2005**

**16**

**128**

**0.00%**

CD010B1_2

7/18/2005

3

127

121

219

225

89

92

172

176

187

199

24

CD013B1

7/18/2005

10

128

129

122

219

103

107

172

182

199

80

m36

1

128

122

219

103

107

172

182

199

8

m17, m57

2

129

122

219

103

107

172

182

199

16

m58

1

129

122

123

219

103

107

172

182

199

8

CD014B1

7/18/2005

22

127

122

219

221

89

92

174

182

199

201

176

m22

1

127

122

219

221

89

92

174

182

199

*

8

m24

1

127

122

219

221

89

92

174

182

*

201

8

CD015B1

7/18/2005

2

126

122

221

92

101

184

186

187

197

16

**Subtotal Count**

**7/18/2005**

**43**

**344**

**0.58%**

CD005B1

11/14/2005

4

127

122

219

221

89

92

170

172

187

203

32

m5

1

127

122

123

219

221

89

92

170

172

187

203

8

CD008B1

11/14/2005

6

127

122

221

227

94

101

182

184

197

199

48

m2

1

127

128

122

221

227

94

101

182

184

197

199

8

**Subtotal Count**

**11/14/2005**

**12**

**96**

**0.00%**

**Total Count**

**All Days**

**240**

**1920**

**0.63%**

**# of Alleles with**

**Dinucleotide Repeats**

**Analyzed**

**% Deviation**

**from Expected**

**Bat25**

**Bat26**

**D3S3623**

**D5S346**

**D6S262**

**D7S481**

**Subject**

**Run Date**

**# of**

**Clone(s)**

**Microsatellite Markers**
