# Supplementary material for: Development of a cost-effective high-throughput process of microsatellite analysis involving miniaturized multiplexed PCR amplification and automated allele identification
Source: Hum Genomics. 2013 Mar 5;7(1):6. doi: 10.1186/1479-7364-7-6 (PMC3600708; doi:10.1186/1479-7364-7-6)
Supplement: Additional file 5 — Process accuracy with group II markers. Summary of results from automated allele identification of 1,560 dinucleotide markers from 156 T cell clones from different subjects organized by dates of processing. Percent deviation from expected values connotes the percent of alleles that have been miscalled, indicated by an asterisk. Misidentification can represent failure in any part of the process from sample preparation, PCR amplification, fragment separation, peak detection, fragment sizing, to automated allele calling. We manually analyzed all discordant samples and found failures to be related to either the interferences of nonspecific signals above threshold values or from the unequal amplification of alleles. These failures were likely related to poor sample quality since control samples were performed optimally in the same runs. [file 1479-7364-7-6-S5.doc]

**Additional table 5. Process accuracy with group II markers**

**Allele 1**

**Allele 2**

**Allele 1**

**Allele 2**

**Allele 1**

**Allele 2**

**Allele 1**

**Allele 2**

**Allele 1**

**Allele 2**

UC002B1

6/30/2005

5

193

200

152

168

216

219

136

138

113

50

UC005B1

6/30/2005

3

191

195

168

172

219

236

136

105

113

30

UC006B1

6/30/2005

3

191

204

166

172

219

132

142

113

115

30

UC009B1

6/30/2005

3

191

195

154

170

219

132

136

115

121

30

**Subtotal Count**

**6/30/2005**

**14**

**140**

**0.00%**

CD003B1_2

7/5/2005

32

181

202

164

172

223

236

136

113

320

**Subtotal Count**

**7/5/2005**

**32**

**320**

**0.00%**

CD006B1

7/6/2005

40

191

168

170

219

132

146

111

113

400

m51

1

191

168

170

219

132

146

*

113

10

**Subtotal Count**

**7/6/2005**

**41**

**410**

**0.24%**

CD009B1

7/7/2005

18

191

195

154

170

219

132

136

115

121

180

m103

1

*

195

154

170

219

132

136

115

121

10

m8

1

191

195

154

170

219

132

136

115

*

10

CD010B1_2

7/7/2005

8

195

197

168

170

221

236

136

105

119

80

**Subtotal Count**

**7/7/2005**

**28**

**280**

**0.71%**

CD009B1

7/13/2005

4

191

195

154

170

219

132

136

115

121

40

m37_1ul

1

191

*

154

*

219

132

136

115

121

10

CD013B1

7/13/2005

2

193

168

176

219

221

136

138

105

113

20

m5_1ul

1

193

168

*

219

221

136

*

105

113

10

CD015B1

7/13/2005

5

191

170

176

219

221

142

107

111

50

m12

1

191

170

176

219

221

142

*

107

111

10

**Subtotal Count**

**7/13/2005**

**14**

**140**

**3.57%**

CD015B1

7/14/2005

24

191

170

176

219

221

142

107

111

240

m31

1

*

170

176

219

221

142

107

111

10

m29

1

191

*

176

219

221

142

*

107

111

10

m58

1

191

170

176

219

221

142

*

107

111

10

**Subtotal Count**

**7/14/2005**

**27**

**270**

**1.48%**

**Total Count**

**All Days**

**156**

**1560**

**0.77%**

**# of**

**Clone(s)**

**# of Alleles**

**Analyzed**

**% Deviation**

**from Expected**

**Run Date**

**Subject**

**Microsatellite Markers**

**D17S250**

**D18S61**

**D2S123**

**D3S1262**

**D9S171**
